# Supplementary material for: Aqueous pyruvate partly dissociates under deep ultraviolet irradiation but is resilient to near ultraviolet excitation
Source: Nat Commun. 2024 Mar 4;15:1978. doi: 10.1038/s41467-024-46309-5 (PMC10912111; doi:10.1038/s41467-024-46309-5)
Supplement: Supplementary file 1 — Supplementary Information [file 41467_2024_46309_MOESM1_ESM.docx]

Aqueous pyruvate partly dissociates under deep
ultraviolet irradiation but is resilient to near ultraviolet excitation

Jan Thøgersen, Fani Madzharova, Tobias Weidner and Frank Jensen*

Department of Chemistry, Aarhus University, Langelandsgade 140, DK-8000 Aarhus C, Denmark

*Corresponding Author. Frank Jensen. E-mail: frj@chem.au.dk

Supplementary Information

Contains Supplementary Figures 1-3


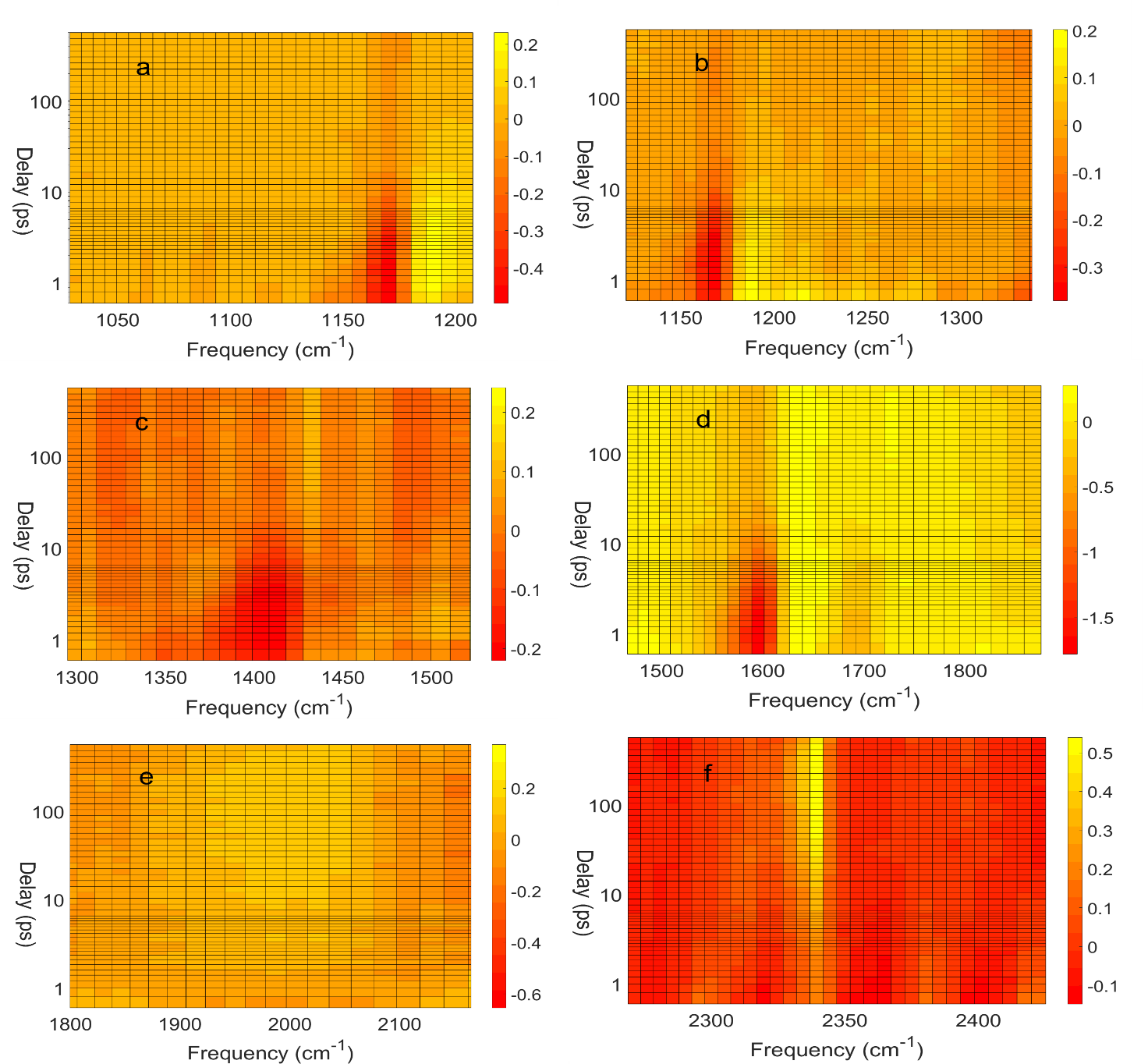


The infrared absorption dynamics of aqueous pyruvate recorded after the 200 nm excitation pulse. **a** The negative transient at 1176 cm^-1^ is associated with the C-CH_3_ stretch transition of ground state pyruvate, while the short-lived positive absorption at 1194 cm^-1^ is assigned to the C-CH_3_ stretch transition of excited state pyruvate. **b** The positive absorption appearing at 1295 cm^-1^ at long delays is assigned to acetic acid. **c** The negative absorption at 1354 cm^-1^ is assigned to the of the CH_3_ umbrella transition of ground state pyruvate. The excitation of the symmetric COO^−^ stretch and symmetric CH_2_ bend vibrations of ground state pyruvate merge with the C-COO^−^ stretch transition of ground state pyruvate to give the wide negative absorption with minimum at 1418 cm^-1^. The positive absorption appearing at 1438 cm^-1^ at long delays is assigned to acetic acid. **d** The negative absorption associated with the excitation of the asymmetric COO^−^ stretch and the C=O stretch vibrations of ground state pyruvate are observed at 1603 cm^-1^ at 1707 cm^-1^, respectively. The short-lived positive absorption observed around 1666 cm^-1^ is assigned to excitation of the COO^−^ stretch transition of excited state pyruvate. The positive absorption appearing at 1735 cm^-1^ at long delays is assigned to acetic acid. **e** The wide positive absorption with maximum around 2000 cm^-1^ reflects spectral shift of the H_2_O combination band induced by the excitation pulse. f) The absorption at 2341cm^-1^ is assigned to the formation of aqueous carbon dioxide. The transient spectra in **a**, **b**, **c**, **e** and **f** are recorded in H_2_O while the data in **d** is recorded in D_2_O. The transient spectra cannot be compared on a common scale. The figure are contour plot of the data shown in Fig. 2 of the main text. Source data are provided as a Source Data file.


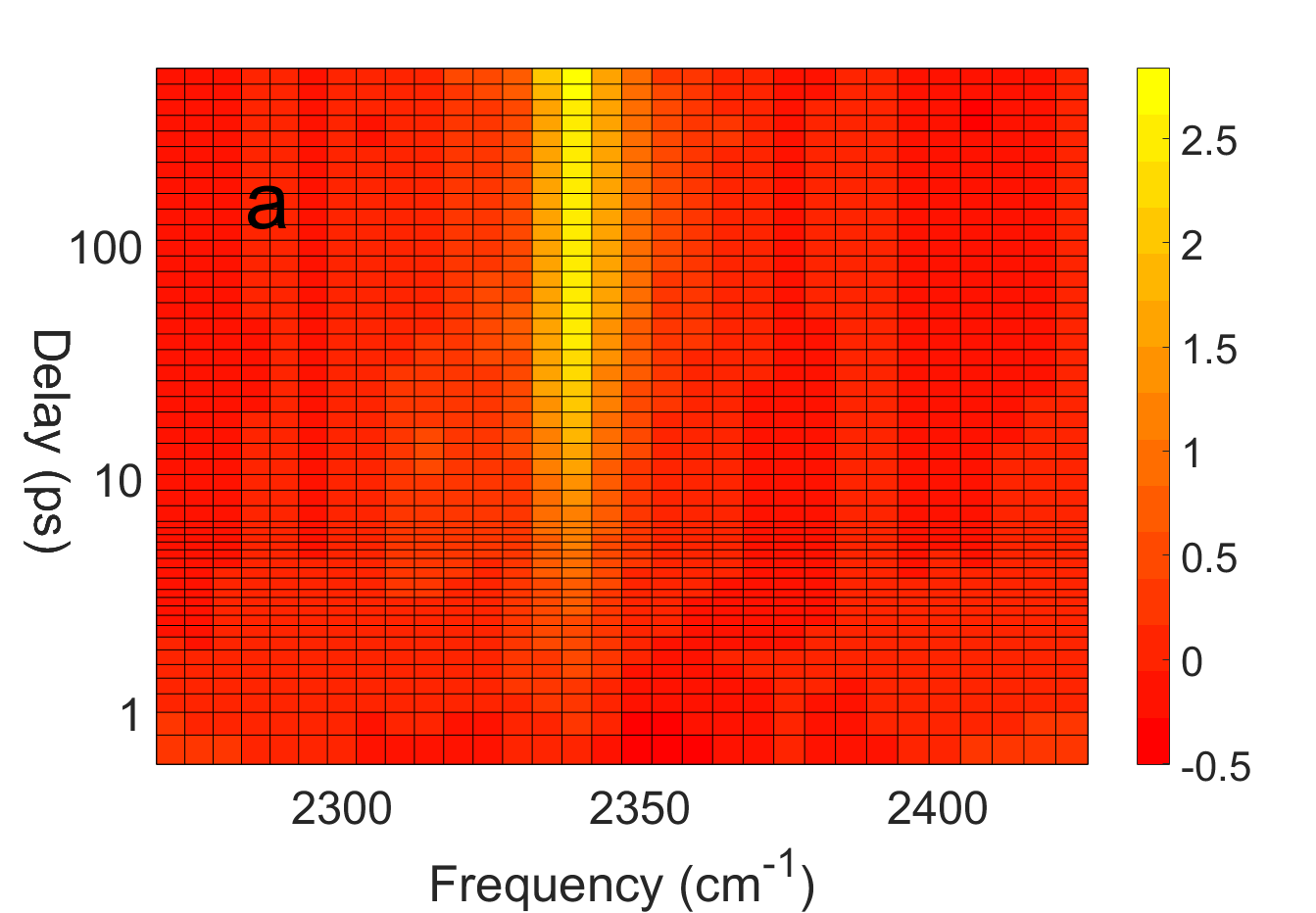


Figure 2. Transient absorption dynamics showing the formation of CO_2_(aq). The figure is a 2D contour plot of Fig. 7a in the main text. Source data are provided as a Source Data file.


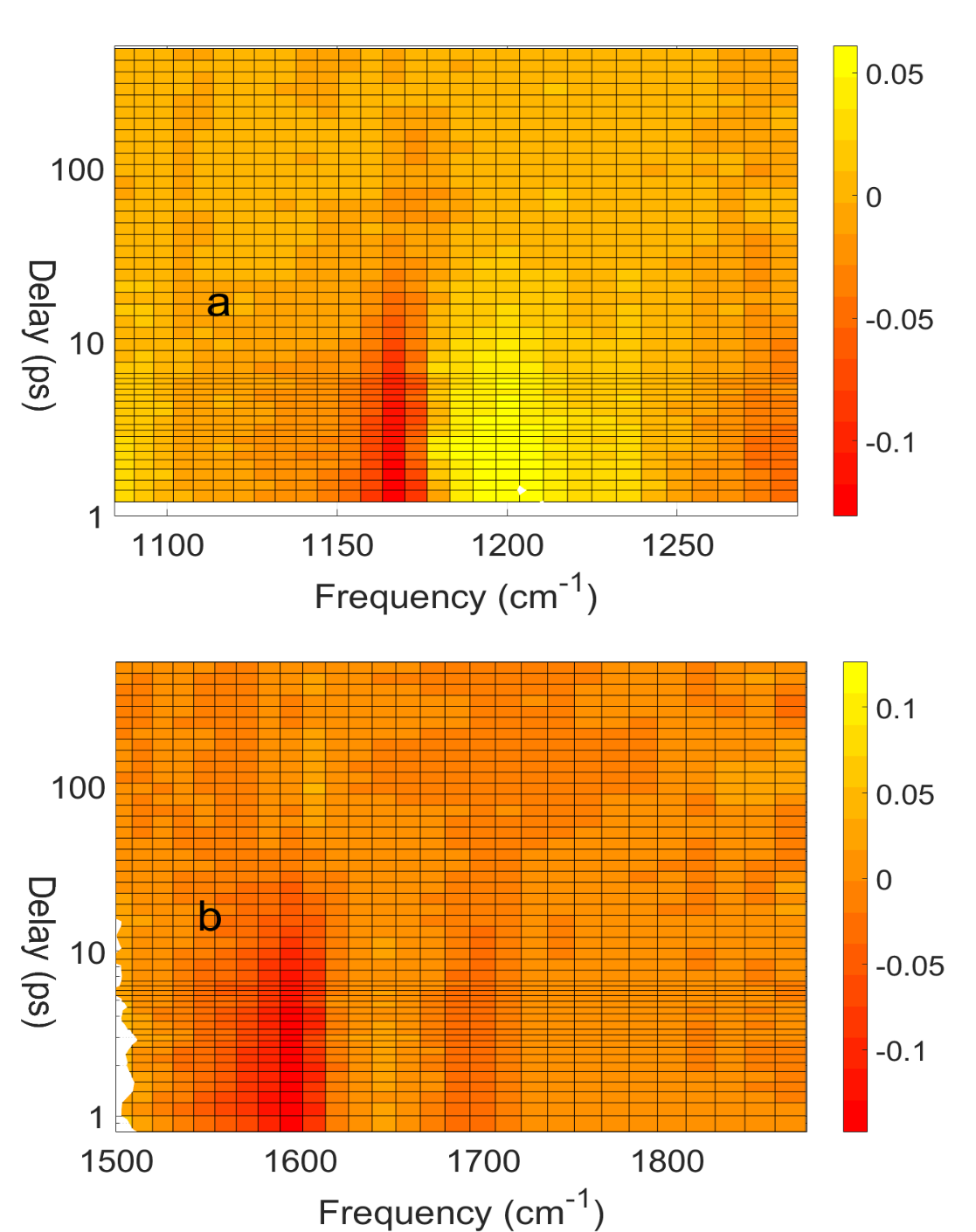


The infrared absorption dynamics following photoexcitation of aqueous pyruvate at 340 nm. **a** 0.3 M pyruvate in H_2_O. **b** 0.1 M pyruvate in D_2_O. The figures are 2D contour plots of Fig. 8a and Fig. 8b in the main text. Source data are provided as a Source Data file.
