# Supplementary material for: Aqueous pyruvate partly dissociates under deep ultraviolet irradiation but is resilient to near ultraviolet excitation
Source: Nat Commun. 2024 Mar 4;15:1978. doi: 10.1038/s41467-024-46309-5 (PMC10912111; doi:10.1038/s41467-024-46309-5)
Supplement: Supplementary file 3 — source data [file 41467_2024_46309_MOESM3_ESM.zip › Source Data/Source data.docx]

Source Data

Fig. 1a

200 1809.91379 174.689

201 1794.65517 165.613

202 1802.24138 154.748

203 1770.21552 142.218

204 1767.80172 129.539

205 1757.62931 116.561

206 1740.21552 104.425

207 1699.22414 93.161

208 1676.50862 83.162

209 1642.19828 74.089

210 1600.34483 65.849

211 1565.86207 58.415

212 1514.48276 51.591

213 1464.91379 45.63

214 1426.16379 40.071

215 1364.35345 35.282

216 1312.88793 30.888

217 1256.2069 26.897

218 1200.64655 23.377

219 1140.43103 20.287

220 1092.06897 17.624

221 1031.59483 15.228

222 970.60345 13.096

223 915.60345 11.193

224 863.44828 9.516

225 809.09483 8.067

226 758.53448 6.835

227 705.99138 5.744

228 654.65517 4.753

229 605.81897 3.877

230 563.10345 3.173

231 525.68966 2.613

232 483.7069 2.067

233 444.43966 1.736

234 403.7069 1.444

235 373.31897 1.184

236 343.66379 0.861

237 314.56897 0.656

238 285.21552 0.486

239 261.50862 0.363

240 240.56034 0.289

241 222.02586 0.221

242 202.93103 0.178

243 180.0431 0.122

244 167.71552 0.073

245 155.64655 0.047

246 139.13793 0.039

247 126.55172 -0.036

248 116.46552 -0.042

249 107.58621 -0.014

250 95.43103 1E-3

251 94.05172 0.01

252 80.12931 0.006

253 76.42241 -0.055

254 68.44828 0.016

255 64.31034 -0.002

256 60.30172 -0.035

257 58.14655 0.002

258 50.86207 0.018

259 46.68103 0.053

260 43.27586 0.059

261 39.61207 0.08

262 39.82759 0.093

263 35.77586 0.089

264 34.39655 0.087

265 33.23276 0.103

266 26.46552 0.061

267 26.25 0.035

268 23.53448 0.055

269 24.43966 0.068

270 23.31897 0.052

271 21.50862 0.03

272 22.97414 0.015

273 16.81034 0.057

274 17.19828 0.079

275 16.59483 0.071

276 14.52586 0.085

277 14.69828 0.099

278 11.55172 0.052

279 13.7069 0.026

280 14.35345 0.017

281 12.84483 0.038

282 12.84483 0.021

283 12.88793 0.063

284 12.5 0.086

285 12.19828 0.098

286 9.74138 0.112

287 10.25862 0.108

288 8.53448 0.119

289 13.10345 0.127

290 10.25862 0.124

291 10.68966 0.112

292 10.77586 0.109

293 10.94828 0.1

294 11.63793 0.134

295 10.81897 0.126

296 7.93103 0.137

297 12.62931 0.101

298 13.10345 0.12

299 12.02586 0.154

300 12.97414 0.168

301 11.16379 0.055

302 13.7931 0.058

303 10.08621 0.098

304 11.12069 0.165

305 11.55172 0.105

306 11.59483 0.011

307 13.87931 0.02

308 12.97414 0.035

309 14.65517 0.057

310 13.7931 0.11

311 1.03448 0.038

312 19.74138 0.022

313 13.75 0.003

314 15.86207 0.054

315 12.75862 0.054

316 16.85345 0.017

317 10.47414 0.046

318 21.07759 0.028

319 7.75862 0.041

320 8.10345 0.037

321 19.43966 0.039

322 11.16379 0.047

323 14.13793 0.032

324 19.05172 0.026

325 12.97414 0.021

326 18.96552 0.026

327 13.14655 0.063

328 13.49138 0.074

329 6.16379 0.077

330 17.28448 0.059

331 8.7069 0.042

332 11.63793 0.051

333 10.81897 0.059

334 17.06897 0.049

335 8.18966 0.055

336 6.07759 0.04

337 10.21552 0.029

338 15.86207 0.042

339 10.94828 0.035

340 12.62931 0.017

341 4.00862 0.033

342 7.19828 0.032

343 2.80172 0.044

344 5.60345 0.043

345 10.90517 0.035

346 6.68103 0.049

347 5.25862 0.029

348 13.49138 0.024

349 4.74138 0.036

350 4.9569 0.026

351 8.7069 0.031

352 -0.12931 0.034

353 6.03448 0.028

354 4.39655 0.036

355 2.19828 0.034

356 -0.68966 0.03

357 3.27586 0.055

358 -2.28448 0.054

359 -0.99138 0.056

360 6.55172 0.045

361 4.13793 0.077

362 -3.44828 0.108

363 4.35345 0.084

364 -2.37069 0.066

365 -4.52586 0.052

366 -2.02586 0.031

367 -0.34483 0.036

368 -3.06034 0.037

369 -1.07759 0.03

370 -5.17241 0.029

371 -3.10345 0.033

372 -6.33621 0.029

373 -2.58621 0.056

374 -4.00862 0.057

375 -5.34483 0.037

376 -3.96552 0.04

377 -7.06897 0.031

378 -5.56034 0.036

379 -5.34483 0.038

380 -8.36207 0.056

381 -7.67241 0.027

382 -4.82759 0.027

383 -4.18103 0.041

384 -3.92241 0.008

385 -4.48276 0.023

386 -7.24138 0.019

387 -2.19828 0.013

388 -5.17241 0.025

389 -7.15517 0.034

390 -3.44828 0.045

391 -4.39655 0.061

392 -1.93966 0.036

393 -5 0.025

394 -3.7931 0.02

395 -3.75 0.034

396 -5 0.037

397 -4.00862 0.028

398 -5.34483 0.045

399 -4.00862 0.016

400 -5.56034 0.029

401 -2.32759 0.022

402 -5.43103 0.027

403 -4.18103 0.008

404 -3.01724 0.01

405 -1.93966 0.034

406 -5.81897 0.057

407 -3.7069 0.067

408 -4.26724 0.065

409 -7.67241 0.053

410 -4.43966 0.05

411 -4.69828 0.061

412 -5.77586 0.044

413 -6.03448 0.023

414 -3.7931 0.044

415 -2.62931 0.028

416 -6.03448 0.025

417 -8.14655 0.03

418 -6.85345 0.026

419 -5.0431 0.028

420 -4.69828 0.027

421 -6.76724 0.054

422 -5.51724 0.042

423 -6.50862 0.034

424 -2.5 0.033

425 -6.37931 0.043

426 -6.33621 0.04

427 -4.05172 0.046

428 -3.75 0.02

429 -4.87069 0.015

430 -5.47414 0.005

431 -4.9569 0.008

432 -2.93103 0.027

433 -5.68966 0.027

434 -6.55172 0.038

435 -6.37931 0.053

436 -7.97414 0.057

437 -2.62931 0.045

438 -4.74138 0.045

439 -6.81034 0.052

440 -5.21552 0.051

441 -4.65517 0.051

442 -5.43103 0.046

443 -4.69828 0.062

444 -4.82759 0.048

445 -4.09483 0.052

446 -5.60345 0.046

447 -4.69828 0.038

448 -4.78448 0.035

449 -3.62069 0.049

450 -5.73276 0.051

451 -3.96552 0.045

452 -4.78448 0.054

453 -7.4569 0.046

454 -4.05172 0.035

455 -4.69828 0.04

456 -5.60345 0.02

457 -4.91379 0.026

458 -4.91379 0.034

459 -5.60345 0.048

460 -5 0.048

461 -4.74138 0.041

462 -5.43103 0.032

463 -5.51724 0.052

464 -5.08621 0.056

465 -5.77586 0.038

466 -4.39655 0.035

467 -5 0.036

468 -4.00862 0.027

469 -2.5 0.031

470 -4.82759 0.027

471 -5.99138 0.028

472 -4.56897 0.027

473 -4.48276 0.044

474 -4.74138 0.058

475 -3.31897 0.056

476 -2.62931 0.032

477 -4.56897 0.032

478 -4.39655 0.029

479 -4.74138 0.035

480 -5.99138 0.048

481 -5.64655 0.056

482 -5.64655 0.037

483 -5.21552 0.053

484 -3.66379 0.048

485 -5.30172 0.04

486 -4.87069 0.022

487 -4.43966 0.019

488 -3.83621 0.039

489 -4.00862 0.037

490 -4.39655 0.037

491 -3.92241 0.048

492 -6.07759 0.046

493 -4.18103 0.042

494 -6.07759 0.04

495 -4.56897 0.046

496 -5.38793 0.044

497 -4.82759 0.059

498 -5.73276 0.052

499 -3.44828 0.038

500 -4.00862 0.04

Fig. 1b

200 1809.91379 33.29487

201 1794.65517 20.92308

202 1802.24138 12.92308

203 1770.21552 8.01026

204 1767.80172 4.53846

205 1757.62931 3.13846

206 1740.21552 1.78718

207 1699.22414 1.66667

208 1676.50862 1.62821

209 1642.19828 1.74872

210 1600.34483 2.05641

211 1565.86207 2.37692

212 1514.48276 2.25641

213 1464.91379 2.70256

214 1426.16379 3.45128

215 1364.35345 3.9641

216 1312.88793 4.54615

217 1256.2069 5.25897

218 1200.64655 6.20513

219 1140.43103 7.29487

220 1092.06897 8.47179

221 1031.59483 9.91282

222 970.60345 11.38462

223 915.60345 13.05641

224 863.44828 14.80256

225 809.09483 16.7641

226 758.53448 19.1

227 705.99138 21.24359

228 654.65517 23.85897

229 605.81897 26.86154

230 563.10345 30.04615

231 525.68966 33.31538

232 483.7069 36.89231

233 444.43966 40.74615

234 403.7069 44.47949

235 373.31897 48.63846

236 343.66379 52.95897

237 314.56897 57.58718

238 285.21552 62.4359

239 261.50862 67.36923

240 240.56034 72.38462

241 222.02586 77.27436

242 202.93103 82.53846

243 180.0431 87.90256

244 167.71552 93.3

245 155.64655 98.64872

246 139.13793 104.24103

247 126.55172 109.95128

248 116.46552 115.54103

249 107.58621 121.04615

250 95.43103 126.46667

251 94.05172 131.54615

252 80.12931 136.53333

253 76.42241 140.97949

254 68.44828 145.52051

255 64.31034 149.80256

256 60.30172 153.6

257 58.14655 157.1641

258 50.86207 160.31795

259 46.68103 163.35128

260 43.27586 165.75385

261 39.61207 167.73846

262 39.82759 169.23333

263 35.77586 170.42564

264 34.39655 170.88718

265 33.23276 170.8641

266 26.46552 170.5359

267 26.25 169.59487

268 23.53448 168.21538

269 24.43966 166.38718

270 23.31897 164.17436

271 21.50862 161.39744

272 22.97414 158.17179

273 16.81034 154.48974

274 17.19828 150.69744

275 16.59483 146.13077

276 14.52586 141.23077

277 14.69828 136.19487

278 11.55172 130.74872

279 13.7069 124.91795

280 14.35345 119.22051

281 12.84483 113.48205

282 12.84483 107.52564

283 12.88793 101.54872

284 12.5 95.40513

285 12.19828 89.28718

286 9.74138 83.17436

287 10.25862 77.30256

288 8.53448 71.31538

289 13.10345 65.38462

290 10.25862 59.84872

291 10.68966 54.85897

292 10.77586 49.71282

293 10.94828 44.74615

294 11.63793 40.17692

295 10.81897 35.98718

296 7.93103 31.84615

297 12.62931 27.90769

298 13.10345 24.43077

299 12.02586 21.3641

300 12.97414 18.28718

301 11.16379 15.80256

302 13.7931 13.6359

303 10.08621 11.78205

304 11.12069 9.97179

305 11.55172 8.28974

306 11.59483 6.85385

307 13.87931 5.58205

308 12.97414 4.47179

309 14.65517 3.58462

310 13.7931 2.92821

311 1.03448 2.4641

312 19.74138 2.01026

313 13.75 1.63333

314 15.86207 1.32821

315 12.75862 0.97949

316 16.85345 0.81538

317 10.47414 0.68462

318 21.07759 0.52821

319 7.75862 0.39744

320 8.10345 0.30513

321 19.43966 0.1359

322 11.16379 0.0641

323 14.13793 0.14103

324 19.05172 0.14103

325 12.97414 0.09744

326 18.96552 0.08974

327 13.14655 0.14359

328 13.49138 0.08974

329 6.16379 0.02308

330 17.28448 0.02308

331 8.7069 0.04359

332 11.63793 0.06154

333 10.81897 0.14359

334 17.06897 0.11026

335 8.18966 0.13846

336 6.07759 0.05641

337 10.21552 0.05128

338 15.86207 0.01538

339 10.94828 -0.07436

340 12.62931 0.01282

341 4.00862 0.09487

342 7.19828 0.03846

343 2.80172 0.06923

344 5.60345 -0.02821

345 10.90517 -0.03077

346 6.68103 0.01538

347 5.25862 -0.07436

348 13.49138 -0.05641

349 4.74138 -0.00513

350 4.9569 0.01282

351 8.7069 0.02821

352 -0.12931 0.10513

353 6.03448 0.07436

354 4.39655 0.0359

355 2.19828 0.0359

356 -0.68966 0.08974

357 3.27586 0.12821

358 -2.28448 0.15641

359 -0.99138 0.1359

360 6.55172 0.14359

361 4.13793 0.09231

362 -3.44828 0.1

363 4.35345 0.10256

364 -2.37069 0.03333

365 -4.52586 0.02051

366 -2.02586 0.00513

367 -0.34483 0.04359

368 -3.06034 0.02564

369 -1.07759 0.00256

370 -5.17241 0.03077

371 -3.10345 0.03846

372 -6.33621 0.01282

373 -2.58621 0.00256

374 -4.00862 -0.02564

375 -5.34483 -0.01795

376 -3.96552 0.06667

377 -7.06897 0.08462

378 -5.56034 0.03077

379 -5.34483 0.01282

380 -8.36207 0.02564

381 -7.67241 -0.02821

382 -4.82759 -0.01795

383 -4.18103 0.03333

384 -3.92241 0.03077

385 -4.48276 0.07179

386 -7.24138 0.04872

387 -2.19828 -0.02821

388 -5.17241 -0.02308

389 -7.15517 0

390 -3.44828 0.06154

391 -4.39655 0.08462

392 -1.93966 0.0359

393 -5 0.02308

394 -3.7931 0.02564

395 -3.75 0.08462

396 -5 0.04615

397 -4.00862 0.01282

398 -5.34483 0.06667

399 -4.00862 0.03846

400 -5.56034 0.06667

401 -2.32759 0.07949

402 -5.43103 0.05385

403 -4.18103 0.02308

404 -3.01724 0.0641

405 -1.93966 0.17692

406 -5.81897 0.15641

407 -3.7069 0.14103

408 -4.26724 0.12564

409 -7.67241 0.08718

410 -4.43966 0.09231

411 -4.69828 0.14103

412 -5.77586 0.1

413 -6.03448 0.09487

414 -3.7931 0.09231

415 -2.62931 0.05641

416 -6.03448 0.04103

417 -8.14655 0.07436

418 -6.85345 0.0641

419 -5.0431 0.09487

420 -4.69828 0.08718

421 -6.76724 0.27436

422 -5.51724 0.22821

423 -6.50862 0.24359

424 -2.5 0.26154

425 -6.37931 0.28462

426 -6.33621 0.29744

427 -4.05172 0.2641

428 -3.75 0.25897

429 -4.87069 0.26923

430 -5.47414 0.22821

431 -4.9569 0.22564

432 -2.93103 0.31282

433 -5.68966 0.29744

434 -6.55172 0.31795

435 -6.37931 0.32821

436 -7.97414 0.31282

437 -2.62931 0.2641

438 -4.74138 0.29487

439 -6.81034 0.3

440 -5.21552 0.2641

441 -4.65517 0.2641

442 -5.43103 0.24103

443 -4.69828 0.2641

444 -4.82759 0.23077

445 -4.09483 0.25128

446 -5.60345 0.27949

447 -4.69828 0.22564

448 -4.78448 0.22564

449 -3.62069 0.3

450 -5.73276 0.30769

451 -3.96552 0.25641

452 -4.78448 0.26154

453 -7.4569 0.26154

454 -4.05172 0.27692

455 -4.69828 0.25897

456 -5.60345 0.22051

457 -4.91379 0.2359

458 -4.91379 0.24615

459 -5.60345 0.27436

460 -5 0.22821

461 -4.74138 0.24872

462 -5.43103 0.22821

463 -5.51724 0.26667

464 -5.08621 0.27949

465 -5.77586 0.25128

466 -4.39655 0.24872

467 -5 0.24872

468 -4.00862 0.22821

469 -2.5 0.27436

470 -4.82759 0.2641

471 -5.99138 0.27692

472 -4.56897 0.22051

473 -4.48276 0.27179

474 -4.74138 0.31795

475 -3.31897 0.32564

476 -2.62931 0.30769

477 -4.56897 0.26923

478 -4.39655 0.25897

479 -4.74138 0.3

480 -5.99138 0.3

481 -5.64655 0.33333

482 -5.64655 0.25385

483 -5.21552 0.25128

484 -3.66379 0.24359

485 -5.30172 0.21795

486 -4.87069 0.18974

487 -4.43966 0.19744

488 -3.83621 0.19487

489 -4.00862 0.21538

490 -4.39655 0.2359

491 -3.92241 0.28718

492 -6.07759 0.26667

493 -4.18103 0.23846

494 -6.07759 0.23846

495 -4.56897 0.24103

496 -5.38793 0.27179

497 -4.82759 0.28718

498 -5.73276 0.29231

499 -3.44828 0.26667

500 -4.00862 0.24359

Fig. 1c

1000.924 0.00257

1002.852 0.00253

1004.781 0.00246

1006.709 0.00258

1008.638 0.00261

1010.567 0.00266

1012.495 0.00291

1014.424 0.00302

1016.352 0.0029

1018.281 0.00286

1020.209 0.00294

1022.138 0.00307

1024.066 0.00309

1025.995 0.0029

1027.924 0.0027

1029.852 0.00262

1031.781 0.00261

1033.709 0.00262

1035.638 0.00273

1037.566 0.00277

1039.495 0.00278

1041.423 0.0027

1043.352 0.00253

1045.281 0.00262

1047.209 0.00272

1049.138 0.0027

1051.066 0.00273

1052.995 0.00287

1054.923 0.00295

1056.852 0.00286

1058.781 0.00285

1060.709 0.00288

1062.638 0.00292

1064.566 0.00271

1066.495 0.00266

1068.423 0.00298

1070.352 0.00298

1072.281 0.00289

1074.209 0.00289

1076.138 0.00295

1078.066 0.00307

1079.995 0.00317

1081.923 0.00328

1083.852 0.0034

1085.78 0.00329

1087.709 0.00347

1089.638 0.00355

1091.566 0.00344

1093.495 0.00347

1095.423 0.00352

1097.352 0.00362

1099.28 0.0035

1101.209 0.00337

1103.137 0.00347

1105.066 0.00353

1106.995 0.00348

1108.923 0.00369

1110.852 0.00384

1112.78 0.00369

1114.709 0.00348

1116.637 0.0036

1118.566 0.00371

1120.495 0.00375

1122.423 0.00375

1124.352 0.00381

1126.28 0.00416

1128.209 0.00428

1130.137 0.00423

1132.066 0.0042

1133.995 0.00417

1135.923 0.0042

1137.852 0.00435

1139.78 0.00453

1141.709 0.00472

1143.637 0.00471

1145.566 0.00471

1147.494 0.00494

1149.423 0.00519

1151.352 0.00536

1153.28 0.00543

1155.209 0.00556

1157.137 0.00589

1159.066 0.00624

1160.994 0.00677

1162.923 0.00741

1164.851 0.00821

1166.78 0.00965

1168.709 0.01161

1170.637 0.01414

1172.566 0.017

1174.494 0.01914

1176.423 0.01959

1178.351 0.01802

1180.28 0.01534

1182.208 0.0122

1184.137 0.00955

1186.066 0.00759

1187.994 0.00597

1189.923 0.00486

1191.851 0.00419

1193.78 0.00372

1195.708 0.00325

1197.637 0.00293

1199.566 0.00274

1201.494 0.00262

1203.423 0.00257

1205.351 0.00246

1207.28 0.0023

1209.208 0.00241

1211.137 0.0025

1213.066 0.00256

1214.994 0.00234

1216.923 0.00202

1218.851 0.00194

1220.78 0.00207

1222.708 0.00222

1224.637 0.0023

1226.565 0.00215

1228.494 0.00202

1230.423 0.00208

1232.351 0.00207

1234.28 0.00205

1236.208 0.00213

1238.137 0.00222

1240.065 0.00203

1241.994 0.00201

1243.922 0.0022

1245.851 0.00212

1247.78 0.00194

1249.708 0.00188

1251.637 0.00194

1253.565 0.00205

1255.494 0.00205

1257.422 0.00209

1259.351 0.00185

1261.28 0.00167

1263.208 0.00181

1265.137 0.0018

1267.065 0.00177

1268.994 0.00184

1270.922 0.00187

1272.851 0.00198

1274.78 0.00219

1276.708 0.00231

1278.637 0.00216

1280.565 0.00206

1282.494 0.00213

1284.422 0.00219

1286.351 0.00213

1288.279 0.00204

1290.208 0.0021

1292.137 0.00231

1294.065 0.00227

1295.994 0.00217

1297.922 0.0022

1299.851 0.00226

1301.779 0.00247

1303.708 0.00253

1305.636 0.00258

1307.565 0.00267

1309.494 0.0025

1311.422 0.00232

1313.351 0.00241

1315.279 0.00263

1317.208 0.00275

1319.136 0.00277

1321.065 0.00265

1322.994 0.00264

1324.922 0.00267

1326.851 0.00265

1328.779 0.00275

1330.708 0.00296

1332.636 0.00308

1334.565 0.00319

1336.494 0.00337

1338.422 0.00351

1340.351 0.00356

1342.279 0.00393

1344.208 0.00451

1346.136 0.00497

1348.065 0.00574

1349.993 0.00712

1351.922 0.00905

1353.851 0.01121

1355.779 0.01262

1357.708 0.01205

1359.636 0.01001

1361.565 0.00799

1363.493 0.00659

1365.422 0.00579

1367.35 0.00538

1369.279 0.00514

1371.208 0.00499

1373.136 0.00495

1375.065 0.00497

1376.993 0.00501

1378.922 0.0053

1380.85 0.00585

1382.779 0.00659

1384.708 0.00724

1386.636 0.00776

1388.565 0.00828

1390.493 0.00896

1392.422 0.00967

1394.35 0.01016

1396.279 0.01024

1398.208 0.0101

1400.136 0.01

1402.065 0.00977

1403.993 0.00941

1405.922 0.0091

1407.85 0.00889

1409.779 0.00879

1411.707 0.00859

1413.636 0.00836

1415.565 0.00839

1417.493 0.00881

1419.422 0.00928

1421.35 0.00923

1423.279 0.00897

1425.207 0.00898

1427.136 0.00886

1429.064 0.00821

1430.993 0.00735

1432.922 0.00651

1434.85 0.00576

1436.779 0.00526

1438.707 0.00472

1440.636 0.00437

1442.564 0.00412

1444.493 0.0038

1446.422 0.00362

1448.35 0.00336

1450.279 0.00313

1452.207 0.00308

1454.136 0.00309

1456.064 0.00301

1457.993 0.00262

1459.922 0.00204

1461.85 0.00148

1463.779 0.00122

1465.707 0.00147

1467.636 0.00177

1469.564 0.00163

1471.493 0.00119

1473.421 0.00108

1475.35 0.00148

1477.279 0.00203

1479.207 0.00224

1481.136 0.00238

1483.064 0.00248

1484.993 0.00242

1486.921 0.00253

1488.85 0.0028

1490.778 0.00286

1492.707 0.00273

1494.636 0.0027

1496.564 0.00298

1498.493 0.00312

1500.421 0.00309

1502.35 0.00312

1504.278 0.00312

1506.207 0.0033

1508.135 0.00343

1510.064 0.0035

1511.993 0.00338

1513.921 0.00335

1515.85 0.00375

1517.778 0.00408

1519.707 0.00409

1521.635 0.00425

1523.564 0.00431

1525.493 0.00429

1527.421 0.00426

1529.35 0.00424

1531.278 0.00448

1533.207 0.00479

1535.135 0.00497

1537.064 0.00492

1538.993 0.00487

1540.921 0.00517

1542.85 0.00547

1544.778 0.00591

1546.707 0.00643

1548.635 0.00689

1550.564 0.0075

1552.492 0.00797

1554.421 0.00851

1556.35 0.00907

1558.278 0.0099

1560.207 0.01074

1562.135 0.01121

1564.064 0.01197

1565.992 0.01299

1567.921 0.014

1569.849 0.0154

1571.778 0.01672

1573.707 0.01782

1575.635 0.01909

1577.564 0.02072

1579.492 0.02278

1581.421 0.02495

1583.349 0.02668

1585.278 0.02873

1587.207 0.03086

1589.135 0.03284

1591.064 0.0347

1592.992 0.03622

1594.921 0.03755

1596.849 0.03866

1598.778 0.03943

1600.707 0.03982

1602.635 0.04001

1604.564 0.03973

1606.492 0.03914

1608.421 0.03823

1610.349 0.0368

1612.278 0.03528

1614.206 0.03381

1616.135 0.03204

1618.064 0.03018

1619.992 0.02837

1621.921 0.02657

1623.849 0.02532

1625.778 0.02409

1627.706 0.02253

1629.635 0.02102

1631.563 0.01975

1633.492 0.01859

1635.421 0.0177

1637.349 0.01672

1639.278 0.01569

1641.206 0.01496

1643.135 0.01434

1645.063 0.01342

1646.992 0.01284

1648.921 0.01245

1650.849 0.0118

1652.778 0.01161

1654.706 0.01113

1656.635 0.01033

1658.563 0.00986

1660.492 0.0095

1662.421 0.00884

1664.349 0.00845

1666.278 0.00823

1668.206 0.00771

1670.135 0.00718

1672.063 0.00681

1673.992 0.00671

1675.92 0.00663

1677.849 0.00624

1679.778 0.00606

1681.706 0.00613

1683.635 0.00604

1685.563 0.00602

1687.492 0.00595

1689.42 0.00637

1691.349 0.00704

1693.277 0.00727

1695.206 0.00793

1697.135 0.00911

1699.063 0.0101

1700.992 0.01138

1702.92 0.01226

1704.849 0.01297

1706.777 0.01332

1708.706 0.01317

1710.635 0.01266

1712.563 0.01153

1714.492 0.01018

1716.42 0.0091

1718.349 0.00782

1720.277 0.00647

1722.206 0.00532

1724.135 0.00477

1726.063 0.00423

1727.992 0.00365

1729.92 0.00322

1731.849 0.00259

1733.777 0.00237

1735.706 0.00219

1737.634 0.00157

1739.563 0.00133

1741.492 0.00137

1743.42 0.00155

1745.349 0.00178

1747.277 0.00169

1749.206 0.00161

1751.134 0.00151

1753.063 0.00131

1754.991 0.00113

1756.92 0.00106

1758.849 8.60628E-4

1760.777 8.99147E-4

1762.706 9.45359E-4

1764.634 7.32724E-4

1766.563 7.53524E-4

1768.491 7.33641E-4

1770.42 6.06392E-4

1772.349 7.14256E-4

1774.277 6.44429E-4

1776.206 5.52098E-4

1778.134 4.99029E-4

1780.063 3.80026E-4

1781.991 3.63627E-4

1783.92 3.41156E-4

1785.848 4.33901E-4

1787.777 4.14321E-4

1789.706 2.20042E-4

1791.634 3.59868E-4

1793.563 5.02954E-4

1795.491 3.18387E-4

1797.42 3.04038E-4

1799.348 3.64911E-4

1801.277 4.5888E-4

1803.206 4.4547E-4

1805.134 2.18003E-4

1807.063 2.31326E-4

1808.991 1.38847E-4

1810.92 1.56287E-4

1812.848 2.60485E-4

1814.777 2.23027E-4

1816.705 2.14359E-4

1818.634 1.40942E-4

1820.563 3.04994E-4

1822.491 3.07108E-4

1824.42 3.05435E-4

1826.348 3.64618E-4

1828.277 2.137E-4

1830.205 2.07512E-4

1832.134 3.36941E-4

1834.063 4.5365E-4

1835.991 5.10534E-4

1837.92 3.78611E-4

1839.848 3.02565E-4

1841.777 3.2987E-4

1843.705 3.2442E-4

1845.634 2.28749E-4

1847.562 6.67718E-5

1849.491 3.1393E-5

1851.42 -1.74318E-5

1853.348 -4.801E-5

1855.277 1.96149E-4

1857.205 3.32588E-4

1859.134 3.67375E-4

1861.062 3.9676E-4

1862.991 3.98399E-4

1864.919 4.31426E-4

1866.848 3.92499E-4

1868.777 4.69036E-4

1870.705 3.4044E-4

1872.634 3.97148E-4

1874.562 5.89657E-4

1876.491 4.89952E-4

1878.419 3.03269E-4

1880.348 2.33036E-4

1882.277 2.52881E-4

1884.205 3.16262E-4

1886.134 4.63765E-4

1888.062 3.81384E-4

1889.991 2.74813E-4

1891.919 1.9835E-4

1893.848 3.26537E-4

1895.776 4.4987E-4

1897.705 3.59738E-4

1899.634 2.71592E-4

1901.562 2.30216E-4

1903.491 2.5841E-4

1905.419 3.47471E-4

1907.348 3.86305E-4

1909.276 4.25091E-4

1911.205 3.13564E-4

1913.134 2.48922E-4

1915.062 2.6141E-4

1916.991 3.80115E-4

1918.919 5.72661E-4

1920.848 6.18094E-4

1922.776 5.57391E-4

1924.705 5.12019E-4

1926.633 4.76553E-4

1928.562 5.4348E-4

1930.491 4.13468E-4

1932.419 3.21161E-4

1934.348 4.31798E-4

1936.276 3.70089E-4

1938.205 4.28533E-4

1940.133 4.27511E-4

1942.062 3.07977E-4

1943.99 3.84605E-4

1945.919 4.81311E-4

1947.848 4.01078E-4

1949.776 4.14101E-4

1951.705 5.27837E-4

1953.633 4.73811E-4

1955.562 4.55921E-4

1957.49 4.14748E-4

1959.419 4.98504E-4

1961.348 5.85056E-4

1963.276 5.51232E-4

1965.205 4.79793E-4

1967.133 4.48103E-4

1969.062 4.82156E-4

1970.99 4.68799E-4

1972.919 5.32775E-4

1974.848 6.60186E-4

1976.776 5.63143E-4

1978.705 3.98922E-4

1980.633 3.22265E-4

1982.562 4.18222E-4

1984.49 5.55083E-4

1986.419 4.41709E-4

1988.347 4.26377E-4

1990.276 4.32049E-4

1992.205 4.30868E-4

1994.133 4.55369E-4

1996.062 4.67424E-4

1997.99 3.95281E-4

1999.919 3.09084E-4

Fig. 1d

998.9951 5.161E-4

1000.924 4.47362E-4

1002.852 4.53235E-4

1004.781 5.58201E-4

1006.709 3.10781E-4

1008.638 2.14657E-4

1010.567 5.00028E-4

1012.495 5.70457E-4

1014.424 6.80817E-4

1016.352 8.26981E-4

1018.281 8.37956E-4

1020.209 9.25857E-4

1022.138 9.80158E-4

1024.066 7.1941E-4

1025.995 4.97883E-4

1027.924 3.44589E-4

1029.852 1.33748E-4

1031.781 4.67818E-5

1033.709 7.42546E-5

1035.638 1.57082E-4

1037.566 1.36426E-4

1039.495 1.27124E-4

1041.423 3.54937E-4

1043.352 2.72211E-4

1045.281 6.7701E-5

1047.209 -2.97779E-5

1049.138 7.71391E-5

1051.066 1.30352E-4

1052.995 3.64863E-5

1054.923 2.49936E-5

1056.852 3.39301E-5

1058.781 4.87211E-5

1060.709 1.1706E-4

1062.638 6.63348E-5

1064.566 -1.58322E-4

1066.495 -1.39145E-4

1068.423 -3.088E-5

1070.352 4.6615E-6

1072.281 1.16174E-4

1074.209 2.11218E-4

1076.138 2.49374E-4

1078.066 1.64731E-4

1079.995 2.0176E-4

1081.923 3.53471E-4

1083.852 2.9504E-4

1085.78 1.91384E-4

1087.709 -7.93795E-5

1089.638 -1.2021E-5

1091.566 1.53109E-4

1093.495 7.10921E-5

1095.423 1.44002E-4

1097.352 2.77229E-4

1099.28 2.31279E-4

1101.209 1.91775E-4

1103.137 2.80147E-5

1105.066 1.97543E-5

1106.995 2.16932E-4

1108.923 3.41042E-5

1110.852 -7.81436E-5

1112.78 1.04386E-4

1114.709 1.66038E-4

1116.637 1.98695E-4

1118.566 2.46548E-4

1120.495 1.7102E-4

1122.423 3.63927E-4

1124.352 6.13253E-4

1126.28 6.09176E-4

1128.209 7.71495E-4

1130.137 6.67213E-4

1132.066 5.77679E-4

1133.995 8.25858E-4

1135.923 0.00107

1137.852 0.00115

1139.78 0.00123

1141.709 0.00132

1143.637 0.00138

1145.566 0.00145

1147.494 0.00167

1149.423 0.00181

1151.352 0.00205

1153.28 0.00231

1155.209 0.00251

1157.137 0.00284

1159.066 0.00315

1160.994 0.00348

1162.923 0.00425

1164.851 0.00559

1166.78 0.0073

1168.709 0.00932

1170.637 0.01176

1172.566 0.01394

1174.494 0.01472

1176.423 0.01365

1178.351 0.01132

1180.28 0.00868

1182.208 0.00606

1184.137 0.00403

1186.066 0.00275

1187.994 0.00186

1189.923 0.00131

1191.851 0.00106

1193.78 8.52704E-4

1195.708 7.20812E-4

1197.637 7.85024E-4

1199.566 8.45072E-4

1201.494 9.36242E-4

1203.423 0.00109

1205.351 0.00124

1207.28 0.00124

1209.208 0.00134

1211.137 0.00141

1213.066 0.00143

1214.994 0.00124

1216.923 0.00121

1218.851 0.00111

1220.78 0.00108

1222.708 0.00107

1224.637 0.00107

1226.565 0.00102

1228.494 7.50096E-4

1230.423 7.57173E-4

1232.351 6.35698E-4

1234.28 3.87977E-4

1236.208 3.89678E-4

1238.137 2.33795E-4

1240.065 6.95454E-5

1241.994 8.59761E-5

1243.922 1.77231E-4

1245.851 1.48745E-4

1247.78 1.52654E-4

1249.708 1.95401E-4

1251.637 3.22495E-4

1253.565 3.7159E-4

1255.494 1.51832E-4

1257.422 8.31995E-5

1259.351 1.35304E-4

1261.28 -2.12612E-5

1263.208 7.7726E-5

1265.137 2.14791E-4

1267.065 2.30373E-4

1268.994 2.56471E-4

1270.922 2.39034E-4

1272.851 2.21714E-4

1274.78 2.7351E-4

1276.708 2.13647E-4

1278.637 1.85286E-4

1280.565 2.52447E-4

1282.494 2.22403E-4

1284.422 2.65447E-4

1286.351 4.57969E-4

1288.279 5.44126E-4

1290.208 3.88842E-4

1292.137 4.04881E-4

1294.065 5.35167E-4

1295.994 4.84215E-4

1297.922 3.67618E-4

1299.851 5.06219E-4

1301.779 5.92407E-4

1303.708 5.94636E-4

1305.636 6.24266E-4

1307.565 7.63222E-4

1309.494 7.39575E-4

1311.422 4.91171E-4

1313.351 5.44058E-4

1315.279 7.5926E-4

1317.208 7.71622E-4

1319.136 6.91128E-4

1321.065 7.78579E-4

1322.994 8.87489E-4

1324.922 9.59382E-4

1326.851 8.60618E-4

1328.779 8.88034E-4

1330.708 0.00103

1332.636 0.00111

1334.565 0.00133

1336.494 0.00141

1338.422 0.00155

1340.351 0.00159

1342.279 0.00171

1344.208 0.00197

1346.136 0.00217

1348.065 0.00274

1349.993 0.00367

1351.922 0.00498

1353.851 0.00697

1355.779 0.00878

1357.708 0.00887

1359.636 0.00747

1361.565 0.00582

1363.493 0.00471

1365.422 0.00393

1367.35 0.0035

1369.279 0.00338

1371.208 0.00332

1373.136 0.00332

1375.065 0.00337

1376.993 0.00352

1378.922 0.00382

1380.85 0.00404

1382.779 0.00437

1384.708 0.00482

1386.636 0.00539

1388.565 0.00598

1390.493 0.00648

1392.422 0.00703

1394.35 0.00766

1396.279 0.00792

1398.208 0.00809

1400.136 0.00818

1402.065 0.00806

1403.993 0.00796

1405.922 0.00783

1407.85 0.00767

1409.779 0.0078

1411.707 0.00822

1413.636 0.00824

1415.565 0.00808

1417.493 0.00839

1419.422 0.0087

1421.35 0.00864

1423.279 0.00852

1425.207 0.00834

1427.136 0.00809

1429.064 0.00772

1430.993 0.00722

1432.922 0.00666

1434.85 0.00635

1436.779 0.00592

1438.707 0.00525

1440.636 0.00485

1442.564 0.0045

1444.493 0.0042

1446.422 0.00442

1448.35 0.00472

1450.279 0.0046

1452.207 0.00462

1454.136 0.00475

1456.064 0.00504

1457.993 0.00513

1459.922 0.00494

1461.85 0.00474

1463.779 0.00456

1465.707 0.00432

1467.636 0.00416

1469.564 0.00408

1471.493 0.00411

1473.421 0.00398

1475.35 0.00358

1477.279 0.00334

1479.207 0.00308

1481.136 0.00295

1483.064 0.003

1484.993 0.00291

1486.921 0.0029

1488.85 0.00308

1490.778 0.003

1492.707 0.00261

1494.636 0.00252

1496.564 0.00272

1498.493 0.00264

1500.421 0.00249

1502.35 0.00239

1504.278 0.00254

1506.207 0.00293

1508.135 0.00271

1510.064 0.00246

1511.993 0.00256

1513.921 0.00245

1515.85 0.00242

1517.778 0.00248

1519.707 0.00264

1521.635 0.00277

1523.564 0.0027

1525.493 0.00268

1527.421 0.00249

1529.35 0.00243

1531.278 0.00268

1533.207 0.00295

1535.135 0.00291

1537.064 0.00307

1538.993 0.00378

1540.921 0.00382

1542.85 0.00358

1544.778 0.0038

1546.707 0.00352

1548.635 0.0035

1550.564 0.00388

1552.492 0.00407

1554.421 0.00448

1556.35 0.00502

1558.278 0.00559

1560.207 0.00583

1562.135 0.00557

1564.064 0.00563

1565.992 0.00597

1567.921 0.00655

1569.849 0.00724

1571.778 0.00759

1573.707 0.00809

1575.635 0.009

1577.564 0.00963

1579.492 0.01022

1581.421 0.01134

1583.349 0.01263

1585.278 0.01405

1587.207 0.01582

1589.135 0.01777

1591.064 0.01988

1592.992 0.02232

1594.921 0.0251

1596.849 0.02828

1598.778 0.03164

1600.707 0.03487

1602.635 0.03799

1604.564 0.04084

1606.492 0.0432

1608.421 0.04531

1610.349 0.04688

1612.278 0.04804

1614.206 0.04883

1616.135 0.04923

1618.064 0.04835

1619.992 0.04639

1621.921 0.04407

1623.849 0.04055

1625.778 0.03642

1627.706 0.0321

1629.635 0.02732

1631.563 0.023

1633.492 0.01954

1635.421 0.01633

1637.349 0.01326

1639.278 0.01089

1641.206 0.00907

1643.135 0.00771

1645.063 0.00671

1646.992 0.00568

1648.921 0.00478

1650.849 0.00449

1652.778 0.00462

1654.706 0.00376

1656.635 0.00291

1658.563 0.00265

1660.492 0.00254

1662.421 0.00264

1664.349 0.00237

1666.278 0.00211

1668.206 0.00234

1670.135 0.00238

1672.063 0.00195

1673.992 0.00224

1675.92 0.00246

1677.849 0.0022

1679.778 0.0025

1681.706 0.00289

1683.635 0.00338

1685.563 0.00367

1687.492 0.00366

1689.42 0.00409

1691.349 0.0049

1693.277 0.00586

1695.206 0.00766

1697.135 0.00943

1699.063 0.01132

1700.992 0.01354

1702.92 0.01473

1704.849 0.01604

1706.777 0.01676

1708.706 0.01671

1710.635 0.01597

1712.563 0.0145

1714.492 0.01272

1716.42 0.01139

1718.349 0.00972

1720.277 0.00784

1722.206 0.00664

1724.135 0.00572

1726.063 0.00495

1727.992 0.00433

1729.92 0.00395

1731.849 0.00394

1733.777 0.00403

1735.706 0.00343

1737.634 0.0031

1739.563 0.00314

1741.492 0.00304

1743.42 0.00297

1745.349 0.00273

1747.277 0.00262

1749.206 0.00271

1751.134 0.0027

1753.063 0.0022

1754.991 0.00184

1756.92 0.00159

1758.849 0.00123

1760.777 0.00117

1762.706 0.0011

1764.634 9.92291E-4

1766.563 0.0012

1768.491 0.00113

1770.42 9.91591E-4

1772.349 0.00116

1774.277 9.01045E-4

1776.206 6.61822E-4

1778.134 4.99399E-4

1780.063 4.72963E-4

1781.991 5.85895E-4

1783.92 6.47882E-4

1785.848 6.04704E-4

1787.777 4.65222E-4

1789.706 4.612E-4

1791.634 4.82402E-4

1793.563 4.4695E-4

1795.491 5.06795E-4

1797.42 4.33233E-4

1799.348 3.53438E-4

1801.277 4.41183E-4

1803.206 3.90679E-4

1805.134 3.34689E-4

1807.063 3.22705E-4

1808.991 3.71889E-4

1810.92 3.77319E-4

1812.848 2.70506E-4

1814.777 3.01271E-4

1816.705 2.15224E-4

1818.634 1.57496E-4

1820.563 3.35035E-4

1822.491 3.50303E-4

1824.42 4.24301E-4

1826.348 4.01634E-4

1828.277 4.37438E-4

1830.205 4.62464E-4

1832.134 2.78597E-4

1834.063 1.83263E-4

1835.991 2.80166E-4

1837.92 1.66613E-4

1839.848 1.03538E-4

1841.777 1.50811E-4

1843.705 2.81373E-4

1845.634 2.22308E-4

1847.562 1.65389E-4

1849.491 2.49324E-4

1851.42 2.43769E-4

1853.348 2.70683E-4

1855.277 1.91511E-4

1857.205 2.3153E-4

1859.134 2.6215E-4

1861.062 2.12685E-4

1862.991 2.53964E-4

1864.919 4.00556E-4

1866.848 5.24712E-4

1868.777 6.1997E-4

1870.705 5.62848E-4

1872.634 3.83495E-4

1874.562 4.21864E-4

1876.491 3.84083E-4

1878.419 1.64208E-4

1880.348 2.30739E-4

1882.277 4.22515E-4

1884.205 2.71339E-4

1886.134 9.33308E-5

1888.062 2.11093E-4

1889.991 4.26615E-4

1891.919 4.18179E-4

1893.848 3.03432E-4

1895.776 1.37665E-4

1897.705 9.73579E-5

1899.634 3.1434E-4

1901.562 4.80715E-4

1903.491 5.06659E-4

1905.419 4.28916E-4

1907.348 3.58796E-4

1909.276 3.0157E-4

1911.205 1.94406E-4

1913.134 1.04022E-4

1915.062 1.62971E-4

1916.991 4.28012E-4

1918.919 4.88192E-4

1920.848 3.37821E-4

1922.776 3.42855E-4

1924.705 2.91019E-4

1926.633 2.61293E-4

1928.562 3.06135E-4

1930.491 2.13499E-4

1932.419 2.16321E-4

1934.348 2.78938E-4

1936.276 2.91069E-4

1938.205 3.93E-4

1940.133 3.43753E-4

1942.062 2.59153E-4

1943.99 3.36492E-4

1945.919 4.44812E-4

1947.848 4.30593E-4

1949.776 4.22741E-4

1951.705 4.12804E-4

1953.633 3.26274E-4

1955.562 2.99443E-4

1957.49 3.05245E-4

1959.419 2.07394E-4

1961.348 3.26697E-4

1963.276 4.20296E-4

1965.205 3.62885E-4

1967.133 3.03475E-4

1969.062 3.02011E-4

1970.99 3.20591E-4

1972.919 2.3404E-4

1974.848 1.2705E-4

1976.776 2.43538E-4

1978.705 4.9212E-4

1980.633 4.3137E-4

1982.562 2.46036E-4

1984.49 2.17354E-4

1986.419 2.36392E-4

1988.347 3.11262E-4

1990.276 4.10485E-4

1992.205 3.47194E-4

1994.133 2.29877E-4

1996.062 2.9088E-4

1997.99 3.12618E-4

1999.919 3.08161E-4

Fig. 2a

2.0000000e-01 7.8503250e-02 1.6807128e-01 4.1602973e-01 3.4340043e-01 -7.2988277e-02 -4.3008103e-01 -2.1682327e-01 -5.6395399e-03 -6.6122359e-02 -1.5239634e-01 -7.0055209e-02 9.9891559e-02 1.0071989e-01 -1.7390018e-02 -7.2225569e-02 2.2612618e-02 1.1887536e-01 5.4414743e-02 -3.5694852e-02 -5.3513262e-02 -3.8396944e-03 5.7943109e-02 2.4251213e-02 -4.3977344e-02 -5.8669206e-02 -1.0960200e-02 2.9743006e-02 2.5726570e-02 -1.9631832e-03 -3.3420926e-03 2.3817177e-02

4.0000000e-01 8.7063227e-02 1.6408593e-01 3.2527699e-01 2.5982708e-01 -9.8042947e-02 -4.2865107e-01 -2.6379541e-01 -7.2455554e-02 -7.7925915e-02 -1.0733336e-01 -4.0877243e-02 7.1960982e-02 7.2096380e-02 -8.2809794e-04 -2.9560955e-02 3.1888189e-02 8.7328269e-02 4.0369109e-02 -1.8122571e-02 -2.5607161e-02 6.3821866e-03 4.0177883e-02 1.7523243e-02 -2.9479843e-02 -3.5990580e-02 -8.9925923e-04 2.0334071e-02 1.0269280e-02 -1.5145513e-02 -1.5984272e-02 3.9212957e-03

6.0000000e-01 2.4098011e-02 9.2309547e-02 1.9589030e-01 1.4006042e-01 -1.7279733e-01 -4.8387760e-01 -3.5317938e-01 -1.7308909e-01 -1.3056784e-01 -1.1526646e-01 -5.9957160e-02 1.4717798e-02 1.8188448e-02 -1.9062556e-02 -2.6906935e-02 1.2146549e-02 3.9417762e-02 9.1585293e-03 -2.1770028e-02 -1.9772825e-02 2.3148522e-03 2.2358685e-02 6.8486516e-03 -2.0594968e-02 -1.7411929e-02 9.3279862e-03 2.0988627e-02 1.1464147e-02 -7.3879955e-03 -7.4007040e-03 6.7687558e-03

8.0000000e-01 4.6396672e-02 1.0410742e-01 1.8940158e-01 1.3472554e-01 -1.6969500e-01 -4.7851936e-01 -3.6678120e-01 -1.8833621e-01 -1.2823076e-01 -9.6606205e-02 -4.9748948e-02 7.9616119e-03 9.0103929e-03 -1.6206366e-02 -2.1106106e-02 8.0393896e-03 2.1026323e-02 -5.6111295e-04 -2.0927737e-02 -1.8034713e-02 1.3777879e-03 1.5046248e-02 1.3784746e-03 -1.6793401e-02 -9.7865920e-03 1.2199682e-02 1.9329857e-02 1.1470361e-02 -1.7347192e-03 -5.4726392e-03 1.3296005e-02

1.0000000e+00 9.3869075e-02 1.4678121e-01 2.2287004e-01 1.6708921e-01 -1.3737669e-01 -4.4919869e-01 -3.4778849e-01 -1.6647343e-01 -9.5692543e-02 -6.0041827e-02 -2.1784952e-02 2.6335091e-02 2.3938982e-02 -5.8992278e-04 -5.8202634e-03 1.4595661e-02 2.2953226e-02 1.9844757e-03 -1.6025202e-02 -1.4013416e-02 4.6948973e-03 1.4846808e-02 -4.5360811e-04 -1.5809939e-02 -8.0614315e-03 8.3140038e-03 1.1794129e-02 5.1670440e-03 -4.6898136e-03 -1.1651001e-02 -1.1157394e-03

1.2000000e+00 9.5214990e-02 1.4994156e-01 2.2272427e-01 1.6314578e-01 -1.4262691e-01 -4.5150544e-01 -3.5137511e-01 -1.6335489e-01 -8.6635367e-02 -4.9536144e-02 -1.4617467e-02 2.7689432e-02 2.1211329e-02 -1.6836529e-03 -6.6077122e-03 1.1251963e-02 1.8135197e-02 -2.2241772e-03 -1.6492315e-02 -1.3624362e-02 3.4905459e-03 9.9419496e-03 -1.6472679e-03 -1.3551881e-02 -6.2483216e-03 8.8329866e-03 1.0975295e-02 6.9281978e-03 -2.4000380e-03 -9.7838032e-03 -7.5214752e-04

1.4000000e+00 9.5004980e-02 1.5319744e-01 2.2145621e-01 1.5993341e-01 -1.4642116e-01 -4.5446234e-01 -3.5052329e-01 -1.5440849e-01 -7.3717720e-02 -3.9016427e-02 -8.8166995e-03 2.8157057e-02 1.9958784e-02 -2.3498930e-03 -5.8686673e-03 8.4444486e-03 1.4802123e-02 -3.7839603e-03 -1.5457106e-02 -1.3070273e-02 2.1579303e-03 8.1272366e-03 -1.3706069e-03 -1.2274842e-02 -5.3613638e-03 7.5919411e-03 1.2795532e-02 8.6838801e-03 -3.1538689e-03 -5.6475574e-03 -3.6840181e-03

1.6000000e+00 1.0545137e-01 1.6384477e-01 2.2981113e-01 1.6223338e-01 -1.4685888e-01 -4.4872668e-01 -3.3602635e-01 -1.3587323e-01 -5.5438944e-02 -2.5284438e-02 5.9603011e-04 3.1619222e-02 2.0866253e-02 -4.0174300e-04 -3.9526151e-03 1.0691807e-02 1.2536044e-02 -4.1116990e-03 -1.3159536e-02 -1.2086895e-02 1.1308077e-03 7.0925085e-03 -1.0510538e-05 -1.2715285e-02 -4.4105043e-03 6.4674909e-03 1.2366706e-02 6.7224924e-03 -4.3367850e-03 -4.7430825e-03 -4.3896773e-03

1.8500000e+00 1.0866308e-01 1.7060322e-01 2.3102405e-01 1.6007174e-01 -1.4996590e-01 -4.4567632e-01 -3.2233064e-01 -1.1809294e-01 -4.0981625e-02 -1.5035060e-02 4.4060671e-03 3.1887429e-02 1.9186719e-02 -2.0753502e-03 -3.2640445e-03 9.2077228e-03 9.6163911e-03 -5.2455816e-03 -1.3595052e-02 -1.2176861e-02 6.4837264e-05 6.3952370e-03 -5.9111759e-04 -1.2261999e-02 -2.2241660e-03 8.3769738e-03 1.2557359e-02 4.9299436e-03 -4.6953051e-03 -4.3057388e-03 -2.4356960e-03

2.1000000e+00 1.1118462e-01 1.7284026e-01 2.3037213e-01 1.5490061e-01 -1.5443058e-01 -4.4057300e-01 -3.0475955e-01 -1.0065574e-01 -2.8466064e-02 -9.3229958e-03 6.9853553e-03 3.0726901e-02 1.7201577e-02 -2.6156581e-03 -4.1648240e-03 8.3110812e-03 8.3560806e-03 -5.4834492e-03 -1.4166778e-02 -1.0364491e-02 5.5318100e-04 6.9420241e-03 -1.5631574e-03 -1.2161875e-02 -1.5386229e-03 8.7564539e-03 1.1150227e-02 6.7162512e-03 -4.1322382e-03 -6.9939898e-03 -1.1880713e-03

2.3500000e+00 1.1162901e-01 1.7405838e-01 2.2958080e-01 1.4904058e-01 -1.5769496e-01 -4.3301280e-01 -2.8961387e-01 -8.5438438e-02 -2.0365720e-02 -6.7429145e-03 7.0938056e-03 2.8236628e-02 1.4133763e-02 -4.1002188e-03 -4.3300599e-03 7.0978549e-03 7.5038291e-03 -5.7851458e-03 -1.4143390e-02 -8.9258715e-03 1.4461301e-03 7.2853536e-03 -1.4614432e-03 -9.8271509e-03 -3.0389752e-03 7.4682287e-03 9.0659563e-03 9.0897196e-03 -1.6502867e-03 -8.1121851e-03 -2.0130828e-03

2.6000000e+00 1.1033939e-01 1.7135228e-01 2.2463536e-01 1.4048851e-01 -1.6165974e-01 -4.2440514e-01 -2.7295741e-01 -7.1941241e-02 -1.4845289e-02 -5.6568561e-03 6.2204557e-03 2.4307736e-02 1.1814770e-02 -5.2309673e-03 -5.7636400e-03 5.4312779e-03 5.6123367e-03 -5.4442233e-03 -1.4585806e-02 -9.1371261e-03 2.4047895e-03 8.7999349e-03 1.1026584e-03 -8.4030055e-03 -4.0783359e-03 6.1687402e-03 1.1351648e-02 8.1661629e-03 -2.2852538e-03 -6.6581248e-03 -3.5882889e-03

2.8500000e+00 1.1461302e-01 1.7242054e-01 2.2207567e-01 1.3564154e-01 -1.6286303e-01 -4.1095785e-01 -2.5356338e-01 -5.8871024e-02 -1.1531835e-02 -3.5061657e-03 6.6766587e-03 2.2430676e-02 9.9069514e-03 -6.2424224e-03 -7.2176601e-03 5.3193826e-03 6.0045418e-03 -6.6109890e-03 -1.4528659e-02 -8.9312281e-03 1.7662335e-03 8.4013436e-03 2.3110751e-03 -7.4879120e-03 -4.7816037e-03 6.8140334e-03 1.0897052e-02 6.0488576e-03 -9.6746040e-04 -6.5671273e-03 -2.3376845e-03

3.1000000e+00 1.1413705e-01 1.7202831e-01 2.1486698e-01 1.2911212e-01 -1.6248453e-01 -3.9815153e-01 -2.3455673e-01 -5.0914268e-02 -9.7119375e-03 -1.7774894e-03 5.4153540e-03 2.0345901e-02 9.4761537e-03 -6.2095213e-03 -7.1233980e-03 3.6006098e-03 5.2832814e-03 -6.2065785e-03 -1.4472140e-02 -7.9762167e-03 1.1254027e-03 7.2566182e-03 1.7986858e-03 -8.2194500e-03 -4.4094901e-03 7.7172185e-03 1.2832683e-02 6.5215951e-03 -3.2646650e-03 -9.1830333e-03 1.4762395e-03

3.3500000e+00 1.1644551e-01 1.7268945e-01 2.1101253e-01 1.2285471e-01 -1.5934566e-01 -3.8078790e-01 -2.1511924e-01 -4.3638926e-02 -8.3961361e-03 -1.8676952e-03 4.0149395e-03 1.8100342e-02 9.2904519e-03 -5.1537644e-03 -6.6056426e-03 4.3498422e-03 6.4344094e-03 -5.9431950e-03 -1.3535199e-02 -6.9634719e-03 2.0354751e-03 7.2031550e-03 2.5894374e-03 -9.1924518e-03 -4.3042643e-03 6.6966031e-03 9.7351083e-03 7.1612299e-03 -2.4853669e-03 -1.0714330e-02 1.7700439e-03

3.7500000e+00 1.1537195e-01 1.7243228e-01 2.0443723e-01 1.1618730e-01 -1.5450097e-01 -3.6111731e-01 -1.9809810e-01 -3.9426663e-02 -7.7546370e-03 -2.6509485e-03 3.5035092e-03 1.5734876e-02 8.7429920e-03 -4.1982721e-03 -5.6080138e-03 3.6856996e-03 6.1851633e-03 -5.0716322e-03 -1.2409002e-02 -6.2355664e-03 2.9967461e-03 6.7677001e-03 2.5767521e-03 -8.2346170e-03 -3.1625692e-03 4.7977568e-03 8.8879119e-03 6.4028023e-03 -4.1836442e-03 -8.2928121e-03 1.3812681e-03

4.1500000e+00 1.1818619e-01 1.7513010e-01 2.0138545e-01 1.1446694e-01 -1.4537233e-01 -3.3559225e-01 -1.7636198e-01 -3.1780313e-02 -5.7106737e-03 -2.5884411e-03 3.4613727e-03 1.5996459e-02 9.3562109e-03 -1.7916109e-03 -3.9735457e-03 6.3428998e-03 7.3965838e-03 -4.7879382e-03 -1.0759414e-02 -5.2558948e-03 2.7119288e-03 7.4720613e-03 3.0648677e-03 -9.0385943e-03 -3.5372412e-03 7.0300356e-03 8.6150357e-03 4.1035826e-03 -4.6814771e-03 -1.0712274e-02 2.7575022e-04

4.5500000e+00 1.1660455e-01 1.7157392e-01 1.9234954e-01 1.0871203e-01 -1.3860360e-01 -3.1380297e-01 -1.6137733e-01 -3.0217873e-02 -6.9540917e-03 -2.7661084e-03 3.6136630e-03 1.4997512e-02 8.0494326e-03 -1.2355470e-03 -2.4206962e-03 6.9197492e-03 7.2929196e-03 -4.1880227e-03 -9.1232170e-03 -4.8227727e-03 1.9128974e-03 5.7730761e-03 1.6230743e-03 -6.3724365e-03 -2.6674244e-03 7.9351683e-03 7.7327584e-03 4.3427871e-03 -5.0656133e-03 -9.1096201e-03 -2.1753880e-03

4.9500000e+00 1.1332810e-01 1.6358734e-01 1.8446667e-01 1.0355269e-01 -1.3306366e-01 -2.9517603e-01 -1.4902570e-01 -2.8667918e-02 -6.7485395e-03 -3.3980574e-03 1.4414104e-03 1.3695452e-02 7.6833583e-03 -1.3482083e-03 -1.7352177e-03 7.4713950e-03 6.4627283e-03 -3.3574200e-03 -7.2435533e-03 -3.4501441e-03 2.0593654e-03 6.6628958e-03 3.6913965e-03 -5.3081551e-03 -2.6660876e-03 8.2076303e-03 8.7807225e-03 6.0629384e-03 -5.4149480e-03 -9.1084721e-03 -8.8901456e-03

5.3500000e+00 1.0962209e-01 1.5290070e-01 1.7504463e-01 9.4258426e-02 -1.2896732e-01 -2.8000332e-01 -1.4325322e-01 -2.9793301e-02 -9.1844691e-03 -4.3002516e-03 1.0591974e-03 1.0881096e-02 5.1397004e-03 -2.5500291e-03 -1.1060963e-03 6.1605304e-03 5.0189168e-03 -1.6185043e-03 -6.8672678e-03 -3.6802101e-03 2.7158634e-03 5.7955104e-03 3.4549849e-03 -3.5887639e-03 -2.0190921e-03 5.3400043e-03 6.2445562e-03 6.9666202e-03 -4.6543885e-03 -4.8903636e-03 -1.0639175e-02

5.7500000e+00 1.1053882e-01 1.4817623e-01 1.7151564e-01 9.3064170e-02 -1.2071233e-01 -2.6227665e-01 -1.3352683e-01 -2.6349580e-02 -8.0301899e-03 -4.6032623e-03 1.4423145e-03 1.1007359e-02 5.0473969e-03 -8.0534216e-04 -6.4392360e-04 6.6052902e-03 4.9316393e-03 -1.5241686e-03 -7.3727250e-03 -3.2365887e-03 3.8457134e-03 7.9570193e-03 4.8038638e-03 -4.2011885e-03 -1.8302107e-03 5.5314187e-03 4.9920158e-03 5.6225897e-03 -5.2884908e-03 -7.1648314e-03 -9.0323462e-03

6.1500000e+00 1.0699235e-01 1.4466315e-01 1.6508286e-01 8.7270875e-02 -1.1557002e-01 -2.4704265e-01 -1.2766281e-01 -2.6941576e-02 -1.0678304e-02 -4.2159787e-03 3.2279364e-04 9.8621140e-03 2.9367912e-03 -1.9265970e-04 4.4398170e-04 6.2533066e-03 3.6016094e-03 -3.4521131e-03 -7.8564962e-03 -3.4884747e-03 4.0147161e-03 5.2398732e-03 2.5538246e-03 -4.4969043e-03 -5.2409200e-04 6.2258455e-03 1.6918537e-03 2.3365724e-03 -3.9929987e-03 -7.4475001e-03 -1.5487999e-04

6.5500000e+00 1.0752472e-01 1.4298097e-01 1.5952402e-01 8.6630890e-02 -1.0240799e-01 -2.2562715e-01 -1.1402206e-01 -2.3325487e-02 -9.9841505e-03 -2.1210550e-03 8.3717008e-04 1.0462938e-02 6.4863687e-03 1.9122905e-03 1.0452365e-03 8.2364444e-03 3.9471891e-03 -3.3485518e-03 -5.3310991e-03 -1.6620727e-03 4.0472742e-03 6.6530541e-03 3.8287446e-03 -3.5718127e-03 5.5925387e-04 6.2358453e-03 2.4415578e-03 1.6843488e-03 -6.1741304e-03 -8.4882021e-03 -5.4398294e-03

7.6380000e+00 9.7758689e-02 1.2946946e-01 1.4226893e-01 7.3596789e-02 -9.6496861e-02 -2.0732888e-01 -1.0757807e-01 -2.7852876e-02 -1.4971999e-02 -3.7621164e-03 -3.0695217e-03 7.1238659e-03 4.3018351e-03 -1.0111185e-03 1.7987296e-04 7.1907152e-03 3.4083509e-03 -2.1775744e-03 -4.3010470e-03 -2.2140190e-03 4.4039242e-03 4.0802296e-03 3.3673757e-03 -2.6937946e-03 -1.1104088e-03 3.4867685e-03 3.5273346e-03 4.1529146e-03 -4.3855062e-03 -3.5069155e-03 -7.9156090e-03

8.9070000e+00 9.3383141e-02 1.1872860e-01 1.2718669e-01 6.7979729e-02 -8.3692609e-02 -1.8262525e-01 -9.5092727e-02 -2.6825212e-02 -1.2905815e-02 -4.7786040e-03 -1.8329358e-03 6.1862101e-03 6.6592301e-03 -1.1767719e-03 1.3073488e-03 8.7534423e-03 4.2693968e-03 1.2013804e-04 -2.0513725e-03 -1.3914713e-03 4.1695396e-03 5.5040582e-03 3.1528482e-03 -2.1776278e-03 -1.9372100e-03 2.9702918e-03 3.5990974e-03 6.6741216e-03 -7.2306925e-03 -4.3773745e-03 -1.5501594e-02

1.0387000e+01 8.4239699e-02 1.0697332e-01 1.1304327e-01 6.0731756e-02 -7.3981976e-02 -1.5855929e-01 -8.5475494e-02 -2.5880183e-02 -1.2393567e-02 -3.9551418e-03 -9.9435500e-04 6.2690568e-03 4.8484016e-03 -1.4139693e-03 2.8860042e-03 7.8657563e-03 4.0934278e-03 4.6619822e-04 -1.4353816e-03 -2.3537241e-03 5.4551274e-03 4.9801649e-03 1.6444140e-03 -3.0353994e-03 -2.7098506e-03 2.1005168e-03 4.9551899e-03 4.9860192e-03 -7.6261155e-03 -1.9393059e-03 -1.1790283e-02

1.2113000e+01 7.7088332e-02 9.7276859e-02 1.0512492e-01 5.8365343e-02 -6.3514985e-02 -1.3803712e-01 -7.5094415e-02 -2.2968144e-02 -1.0241552e-02 -5.3244935e-03 -9.2644462e-04 5.6328322e-03 4.6556191e-03 -4.6424280e-05 4.5267372e-03 8.4478470e-03 3.9990223e-03 -6.1458050e-04 -1.7551000e-03 -8.4330427e-04 4.4527419e-03 4.7993421e-03 1.5801681e-03 -3.8594081e-03 -2.7626486e-03 4.7055501e-03 5.8299891e-03 3.7518335e-03 -1.0233722e-02 -4.5844545e-03 -7.3451116e-03

1.4125000e+01 7.2163133e-02 8.8630199e-02 9.5225872e-02 5.2068218e-02 -5.2924973e-02 -1.1638619e-01 -6.4244851e-02 -1.8672681e-02 -9.7309978e-03 -2.5204110e-03 -8.1145295e-05 5.9136223e-03 4.6194279e-03 1.5955200e-03 6.0498576e-03 6.8771098e-03 2.7126541e-03 -1.6375792e-04 -2.2433347e-03 -2.9949127e-03 2.9117094e-03 4.0964364e-03 1.8654213e-04 -3.5429978e-03 -3.4771738e-03 3.7807237e-03 7.8127698e-03 3.2950713e-03 -1.1514567e-02 -6.8343122e-03 -1.4469661e-03

1.6472000e+01 6.7138607e-02 7.9783824e-02 8.3677451e-02 4.5236157e-02 -4.7761997e-02 -1.0253956e-01 -5.5355016e-02 -1.6883474e-02 -8.3796477e-03 -3.0178680e-03 -1.3974147e-03 4.8994813e-03 4.2516368e-03 2.5675821e-03 6.3592258e-03 9.0591875e-03 2.9104632e-03 -5.8690955e-04 -1.4915188e-03 -3.9057987e-04 2.5940642e-03 3.1712747e-03 1.2359494e-03 -3.3949778e-03 -3.6258716e-03 2.8039374e-03 5.3058183e-03 2.2625062e-03 -1.0708566e-02 -9.6186009e-03 9.6156216e-04

1.9209000e+01 5.7164143e-02 6.8392470e-02 6.6629617e-02 3.5781144e-02 -4.7565994e-02 -9.1585114e-02 -5.1320141e-02 -1.5455942e-02 -8.3693320e-03 -1.6120678e-03 -1.5975228e-03 3.7573150e-03 4.5148405e-03 2.1454968e-03 4.8404413e-03 7.8415435e-03 2.5697180e-03 -2.9907735e-04 7.4382462e-04 -5.2153441e-04 2.7692609e-03 4.8156650e-03 1.7008373e-03 -2.7876921e-03 -3.0506902e-03 1.4211114e-03 5.3913490e-03 1.3843592e-03 -9.4028219e-03 -9.3847926e-03 -1.7878705e-04

2.2400000e+01 4.8193520e-02 6.0423831e-02 5.5340234e-02 2.7678308e-02 -4.9179642e-02 -8.6592581e-02 -4.7135752e-02 -1.4066340e-02 -9.2421414e-03 -3.3078990e-03 -3.3946370e-03 3.0313381e-03 3.6321026e-03 1.0024474e-03 4.5368770e-03 8.6909417e-03 3.1115693e-03 -1.8416023e-03 1.1971068e-03 6.2075113e-04 4.4727583e-03 4.4855949e-03 5.9307656e-03 -3.3925211e-03 -1.1550209e-03 2.5035458e-03 7.0048262e-03 -1.0551655e-03 -5.2017160e-03 -9.3424136e-03 -6.2381485e-03

2.6122000e+01 3.9208765e-02 4.8436563e-02 4.6178074e-02 1.9549556e-02 -5.1425672e-02 -8.1959567e-02 -4.5562692e-02 -1.4431220e-02 -1.0206606e-02 -5.4542472e-03 -5.5821580e-03 1.0433652e-03 1.9124085e-03 2.9439220e-05 3.5409713e-03 7.4942308e-03 2.6864880e-03 -1.3246104e-03 1.3329576e-03 -6.6745202e-05 4.2840506e-03 5.9031532e-03 5.2747418e-03 -3.2243008e-03 -1.2470558e-03 2.1064307e-03 8.1064797e-03 1.4581127e-03 -2.1989499e-03 -8.4642895e-03 -8.9217897e-03

3.0462000e+01 4.1806273e-02 4.8880704e-02 4.5014837e-02 1.8871583e-02 -4.6792083e-02 -7.4372128e-02 -3.9680792e-02 -1.1906926e-02 -1.0450660e-02 -3.6644011e-03 -5.2101734e-03 3.2943841e-03 1.8360151e-03 9.7117811e-04 4.5673784e-03 8.4081670e-03 1.1981390e-03 -1.8007335e-03 -1.3681032e-03 -2.9224839e-04 3.6012593e-03 4.2842205e-03 4.1681406e-03 -4.1184382e-03 -6.3193737e-04 3.0975899e-03 7.3517914e-03 2.8183485e-03 -1.6420249e-03 -8.2752498e-03 -9.5272159e-03

3.5523000e+01 3.8576381e-02 4.4300888e-02 4.0309502e-02 1.4946146e-02 -4.5621529e-02 -7.0998480e-02 -3.7901939e-02 -1.2878080e-02 -1.1090024e-02 -3.7485031e-03 -5.4311996e-03 2.7902562e-03 5.2261617e-04 2.0280135e-03 4.5429612e-03 7.8903515e-03 1.4531204e-03 -2.4730861e-03 -1.5569135e-03 -5.6663280e-04 4.8731912e-03 5.2400325e-03 8.3173662e-04 -4.1178144e-03 -2.9503579e-04 2.8010256e-03 5.3906905e-03 3.7159795e-03 -2.8950543e-03 -5.2176593e-03 -6.4380531e-03

4.1425000e+01 4.1243106e-02 4.4182692e-02 4.0340040e-02 1.4709096e-02 -4.2601135e-02 -6.7434586e-02 -3.4009468e-02 -1.1622269e-02 -1.0821422e-02 -3.2846398e-03 -5.6825849e-03 4.7544647e-03 1.2119461e-03 2.8940107e-03 3.4398561e-03 6.4657252e-03 2.3665711e-03 -4.4389434e-03 -2.2861140e-03 -2.1565292e-03 4.2985953e-03 4.9557491e-03 4.8612584e-04 -3.6877108e-03 -5.7916172e-04 4.6334072e-03 5.5179973e-03 2.9513093e-03 -5.6055902e-03 -3.6232699e-03 -3.8146012e-03

4.8307000e+01 3.8781570e-02 4.0597270e-02 3.5773939e-02 1.1122535e-02 -4.4658923e-02 -6.7708690e-02 -3.2505630e-02 -1.0017219e-02 -9.4688777e-03 -2.7739744e-03 -4.8321597e-03 2.5842995e-03 6.7018454e-04 1.6216577e-03 3.6559271e-03 5.8312459e-03 2.6889107e-03 -4.6930726e-03 1.4100993e-04 -2.6060873e-03 6.3540960e-03 4.3113918e-03 1.7466693e-03 -4.1941659e-03 -1.0083912e-04 2.3546252e-03 5.6889032e-03 1.6877038e-03 -4.3445651e-03 -4.3387980e-03 -3.3861919e-03

5.6333000e+01 3.7344502e-02 3.8074318e-02 3.3875821e-02 1.0767246e-02 -4.4918778e-02 -6.5491894e-02 -3.1916018e-02 -9.4300133e-03 -7.6380174e-03 -1.6077825e-03 -4.9906743e-03 1.5733253e-03 6.2261781e-04 9.2531944e-04 3.7932602e-03 5.2589825e-03 1.9050735e-03 -3.3448365e-03 3.7525861e-04 -2.5409272e-03 5.1566935e-03 2.1252471e-03 3.9168985e-03 -5.1258174e-03 -1.3713291e-03 1.5306367e-03 6.0813877e-03 3.3089321e-03 -3.1186895e-03 -4.8650710e-03 -2.9507751e-03

6.5692000e+01 3.3833186e-02 3.6788118e-02 3.0038697e-02 6.7551354e-03 -4.6537957e-02 -6.4857245e-02 -3.3545666e-02 -9.3664353e-03 -7.2500441e-03 -3.3426319e-04 -2.8108107e-03 9.5313423e-05 4.7382599e-04 5.9815910e-04 4.7567922e-03 5.5039178e-03 9.3277291e-04 -2.9813613e-03 4.0050546e-04 -1.9582617e-03 4.9700763e-03 1.3206312e-03 4.4368863e-03 -5.1543040e-03 2.8195098e-04 2.0879110e-03 5.2125372e-03 4.4641577e-03 -3.2515329e-03 -6.6971411e-03 -4.0812421e-03

7.6606000e+01 3.2014839e-02 3.3029077e-02 2.8312304e-02 4.9714458e-03 -4.7252846e-02 -6.6326192e-02 -3.3328823e-02 -1.0607911e-02 -7.6852408e-03 -6.9421140e-04 -2.9651688e-03 2.7559136e-04 5.9748926e-04 1.7999930e-03 3.6339507e-03 7.1129225e-03 2.2411793e-03 -2.7279646e-03 1.8884659e-04 8.7000989e-04 3.5799619e-03 2.4731782e-03 2.3878387e-03 -5.2055065e-03 1.2187286e-03 3.5388317e-03 3.0537915e-03 7.7136297e-03 -5.9945993e-03 -7.5748095e-03 -5.5092175e-03

8.9333000e+01 3.2342585e-02 3.1155154e-02 2.5551592e-02 1.5528419e-03 -5.0249092e-02 -6.7261480e-02 -3.2743200e-02 -1.0057074e-02 -7.5815380e-03 -7.9663960e-04 -2.3047089e-03 1.6897422e-04 9.9851217e-04 2.1720549e-03 4.1141320e-03 8.6029848e-03 2.2588987e-03 -2.4724651e-03 1.9826322e-04 1.6111605e-03 2.1020098e-03 4.1476329e-03 1.4048889e-03 -5.0999931e-03 1.6999184e-03 5.3962562e-03 3.2886872e-03 4.3625779e-03 -7.4243396e-03 -8.3793017e-03 -4.8498475e-03

1.0417500e+02 3.3170857e-02 3.1707880e-02 2.3896388e-02 2.7163401e-03 -5.1182907e-02 -6.7283940e-02 -2.9997621e-02 -8.4594219e-03 -7.8704293e-03 -8.0000910e-04 -3.2234760e-03 3.5157155e-04 4.4258394e-04 2.4257253e-03 4.5592059e-03 8.7082807e-03 1.2709150e-03 -2.0598835e-03 -1.7787323e-03 1.1214582e-03 1.5861499e-03 2.2545098e-03 1.0104090e-03 -6.3692453e-03 1.5721564e-03 5.7735914e-03 4.0010706e-03 4.3156379e-03 -6.0659640e-03 -5.5809615e-03 -3.6675502e-03

1.2148300e+02 3.3912772e-02 3.5532087e-02 2.4789078e-02 3.1386737e-03 -5.3251134e-02 -6.4314962e-02 -2.7546368e-02 -6.8988527e-03 -5.7105309e-03 2.6753764e-04 -2.5909889e-03 1.2993169e-04 9.9972389e-04 2.8445267e-03 5.5948591e-03 9.0916187e-03 -5.4558634e-04 -2.7667712e-03 -1.4542428e-03 -8.3126715e-04 1.2472628e-03 1.3061594e-03 1.2299396e-03 -5.8555099e-03 1.0677338e-03 5.7249616e-03 4.3411655e-03 4.9137038e-04 -5.3870330e-03 -2.1496222e-03 -2.1092773e-03

1.4166700e+02 3.5043509e-02 3.6507345e-02 2.5673597e-02 2.5285417e-03 -5.3786480e-02 -6.5038069e-02 -2.7629436e-02 -5.3117342e-03 -5.9073973e-03 6.5319517e-04 -2.7712852e-03 1.3458836e-03 7.5313878e-04 2.7962987e-03 4.5688946e-03 7.9035026e-03 2.1076314e-05 -4.0997215e-03 -1.7071864e-03 -4.8123993e-04 1.0249024e-03 1.2847302e-03 1.2354374e-03 -5.1024123e-03 2.4944726e-04 5.4006502e-03 1.6398645e-03 2.7684580e-03 -4.0171694e-03 -2.2022528e-03 -1.9689970e-03

1.6520400e+02 3.4803282e-02 3.4464462e-02 2.6891458e-02 1.5112151e-03 -5.5911263e-02 -6.9392910e-02 -2.9406938e-02 -6.2428361e-03 -4.9360371e-03 -1.4325559e-04 -2.6424875e-03 8.0765476e-04 1.1532743e-03 2.5965217e-03 5.1162607e-03 8.3964000e-03 5.5587381e-06 -4.3086009e-03 -1.3337088e-03 5.1364614e-04 1.7119142e-03 3.2735842e-03 4.8151609e-04 -3.2547272e-03 7.6711960e-04 4.9540234e-03 1.4666514e-03 3.1393707e-03 -5.3134193e-03 -1.7239545e-03 -5.8421070e-03

1.9265100e+02 3.3968795e-02 3.2935166e-02 2.6166312e-02 -2.6782743e-04 -5.6032585e-02 -7.2536569e-02 -2.8983993e-02 -3.6720178e-03 -5.1157483e-03 -3.0090309e-04 -1.3131489e-03 1.9675645e-03 4.3200262e-04 2.4849790e-03 5.2533533e-03 6.6903638e-03 5.5292687e-05 -3.8768683e-03 -3.5504190e-03 -2.5618848e-05 3.2529792e-03 2.3841941e-03 1.3045490e-03 -4.6550630e-03 1.8062024e-04 5.5928958e-03 3.9500947e-03 2.5802477e-03 -4.1192489e-03 -4.4666338e-03 -4.4907816e-03

2.2465800e+02 3.2560418e-02 3.2716227e-02 2.5498268e-02 -4.2386412e-04 -5.7471183e-02 -7.3384641e-02 -2.5575134e-02 -3.2305002e-03 -4.4635226e-03 6.0040151e-04 -9.7373947e-04 1.4480934e-03 1.4698817e-04 2.6590809e-03 6.2288874e-03 6.5189692e-03 -3.1573515e-04 -3.5675769e-03 -4.9435325e-03 -9.4353708e-04 4.2631803e-03 1.5247627e-03 -3.7594206e-04 -4.7440497e-03 1.5402690e-04 3.6182430e-03 6.7832548e-03 2.1097516e-03 -5.6992113e-03 -2.1552137e-03 -3.1351379e-03

2.6198300e+02 3.0524551e-02 3.1201888e-02 2.3312651e-02 -4.0525595e-03 -6.1529081e-02 -7.4359527e-02 -2.2811116e-02 -2.2456813e-03 -3.3186649e-03 2.5032096e-03 -4.6739570e-04 1.3838980e-03 3.9818545e-04 3.2566090e-03 5.8563576e-03 6.5343320e-03 -7.4652298e-04 -3.0585529e-03 -4.6703948e-03 -7.4764515e-04 5.1001393e-03 2.3597739e-03 -1.9082218e-03 -6.1836249e-03 -7.9509839e-05 3.3810837e-03 6.8934833e-03 2.2430501e-03 -5.6868229e-03 -2.9697108e-03 -2.6258247e-03

3.0551000e+02 3.0617039e-02 3.0899706e-02 2.5056684e-02 -4.4631268e-03 -6.2875094e-02 -7.4680528e-02 -2.0673012e-02 -7.8740187e-05 -1.9675166e-03 2.4572407e-03 -1.4298337e-03 1.9789458e-03 1.3079813e-03 2.9457229e-03 5.5953345e-03 6.8230258e-03 5.5471768e-04 -3.0986255e-03 -4.2843304e-03 -1.1800160e-03 4.3837390e-03 3.4053950e-03 -2.3935497e-03 -6.5582259e-03 -1.1983952e-03 3.2358828e-03 6.3832497e-03 3.9223806e-03 -5.1063499e-03 -4.0265354e-03 -2.9107381e-03

3.5626700e+02 2.7843722e-02 2.8555049e-02 2.4226054e-02 -4.8390847e-03 -6.5183125e-02 -7.7026432e-02 -2.1326974e-02 7.5066950e-04 -2.5402446e-04 3.0611436e-03 -2.0689070e-03 1.1179500e-04 9.4227172e-04 1.2965944e-03 3.2428822e-03 5.7949680e-03 -1.3860370e-04 -4.4692436e-03 -3.7829862e-03 -3.9615724e-04 3.2806253e-03 3.3967831e-03 -1.3872231e-03 -8.5892279e-03 1.1763754e-04 4.0379448e-03 3.8786340e-03 1.8640128e-03 -2.1975777e-03 -3.3830972e-03 -1.5941402e-03

4.1545800e+02 2.2711552e-02 2.4957988e-02 2.0387420e-02 -4.9584207e-03 -6.6948068e-02 -7.9421992e-02 -2.3554269e-02 1.3250864e-03 7.1578425e-04 4.7679257e-03 -2.3793327e-03 -9.0378029e-04 -6.6048725e-04 -2.7180564e-04 3.2466289e-03 5.8075628e-03 -5.9187707e-04 -3.8965176e-03 -3.6508077e-03 -2.6215233e-04 3.8204194e-03 1.6062531e-03 1.4174016e-03 -6.7703761e-03 -4.2628284e-04 3.6818496e-03 1.3082258e-03 3.9208612e-04 -1.0372945e-03 -2.9852738e-03 4.6156949e-05

4.8448300e+02 7.8470150e-03 1.1633036e-02 9.1117936e-03 -1.4052993e-02 -7.7827536e-02 -8.8343652e-02 -3.3774429e-02 -5.6688924e-03 -2.4050100e-03 1.5463083e-03 -5.8851252e-03 -7.1294435e-03 -4.5800603e-03 -3.2976711e-03 1.5537453e-03 4.3949257e-03 -3.2086791e-03 -3.7805719e-03 -2.6315438e-03 -1.8467549e-04 4.7393287e-03 2.6341392e-03 3.1728007e-03 -5.9849363e-03 2.4070511e-03 5.5802564e-03 -2.6412341e-04 1.7604779e-03 -3.3833886e-04 1.7370615e-03 1.3401378e-04

5.6497600e+02 1.6788791e-03 7.0847902e-03 6.7578089e-03 -1.6857927e-02 -8.2257711e-02 -9.1154438e-02 -3.8298272e-02 -4.5223152e-03 -1.3776081e-03 1.2933672e-03 -4.4876472e-03 -7.3523535e-03 -4.1992326e-03 -2.2635460e-03 3.9321629e-03 7.3130676e-03 -3.2909429e-03 3.1606790e-04 -4.2325037e-04 -1.5605364e-03 8.1184483e-03 4.4313187e-03 4.4095645e-03 -5.3761169e-03 1.4108447e-03 6.8943914e-03 -2.0973300e-03 4.9446972e-03 -5.3654744e-03 6.6242732e-05 -6.0868629e-03

Fig. 2b

2.0000000e-01 -3.0089316e-01 -2.1347355e-01 -1.5030565e-01 1.3071972e-02 -1.9662236e-01 -4.1443325e-01 -1.5127285e-01 2.3994633e-01 1.5418612e-01 -1.3876598e-01 3.6348105e-02 3.3813000e-01 2.3705393e-01 1.2152590e-02 1.6322262e-01 5.3424384e-01 4.3141223e-01 2.2190397e-01 1.8651398e-01 4.1586905e-01 4.5751039e-01 5.0427167e-02 -3.1055286e-01 -2.0454446e-01 2.4998079e-02 -5.0929037e-02 -2.1857043e-01 -2.5801919e-01 -1.7876523e-01 -1.9356256e-01 -3.3830514e-01

4.0000000e-01 -2.6330387e-01 -1.8098172e-01 -1.1787528e-01 -2.1925912e-02 -1.2876916e-01 -2.1880999e-01 -5.8848505e-02 1.5389877e-01 9.6493556e-02 -5.1186829e-02 6.2301872e-02 2.2341386e-01 1.5663732e-01 4.4155715e-02 1.3993385e-01 3.4825551e-01 2.8666749e-01 1.6773760e-01 1.7182567e-01 3.1526224e-01 2.9933154e-01 -2.3263480e-02 -3.0458447e-01 -2.2029434e-01 -3.8616304e-02 -5.6607783e-02 -1.3149980e-01 -1.4880862e-01 -9.8117061e-02 -1.1108885e-01 -1.9599293e-01

6.0000000e-01 -2.1616302e-01 -1.4651817e-01 -8.5087450e-02 -3.9250627e-02 -7.8674923e-02 -8.5479033e-02 1.1392193e-03 8.2816547e-02 4.8285541e-02 2.6493408e-03 6.9051634e-02 1.2838400e-01 8.8517899e-02 5.4864367e-02 1.1068199e-01 2.0281927e-01 1.7086593e-01 1.2195260e-01 1.5213316e-01 2.3327107e-01 1.7794437e-01 -7.9425008e-02 -3.0729184e-01 -2.3768468e-01 -8.8118145e-02 -6.1832925e-02 -7.0460122e-02 -6.8922574e-02 -3.2934185e-02 -4.3936597e-02 -9.3389848e-02

8.0000000e-01 -1.3244822e-01 -8.3206274e-02 -4.0260183e-02 -1.9000345e-02 -4.6939532e-02 -4.5380221e-02 7.1630761e-03 4.4891808e-02 1.5184722e-02 -4.2011354e-03 3.6424583e-02 6.4638524e-02 3.5507972e-02 2.5181845e-02 6.6927700e-02 1.1649940e-01 9.7459772e-02 7.5525963e-02 1.0992075e-01 1.6764522e-01 1.0727177e-01 -1.3116589e-01 -3.4418175e-01 -2.7628648e-01 -1.3493878e-01 -8.9163033e-02 -6.6376972e-02 -5.4224025e-02 -2.4482303e-02 -2.5307937e-02 -6.2963744e-02

1.0000000e+00 -8.8494320e-02 -5.1666198e-02 -1.9142335e-02 -7.5939151e-03 -3.3348458e-02 -3.2318488e-02 7.1727993e-03 2.6864207e-02 2.4138664e-03 -8.2364264e-03 2.2752121e-02 3.6387631e-02 1.6058224e-02 1.2119016e-02 4.9202455e-02 8.3361654e-02 6.9098720e-02 6.1553760e-02 9.5501211e-02 1.4858051e-01 8.6000420e-02 -1.4439863e-01 -3.5361520e-01 -2.8537755e-01 -1.3920791e-01 -8.4081853e-02 -5.4527971e-02 -3.4414841e-02 -9.8287128e-03 -6.8368395e-03 -3.9355474e-02

1.2000000e+00 -6.4823803e-02 -3.0639699e-02 -1.1564700e-02 -4.6723872e-03 -2.7391664e-02 -2.3665086e-02 4.8181405e-03 1.7562487e-02 -4.3318949e-03 -1.2642841e-02 1.2882159e-02 2.3528960e-02 7.2377603e-03 5.1314382e-03 4.0153881e-02 6.6172988e-02 5.3615001e-02 5.3278838e-02 8.9164760e-02 1.3803937e-01 7.2824950e-02 -1.5766021e-01 -3.6516749e-01 -2.9335591e-01 -1.4325248e-01 -8.3731569e-02 -4.9392500e-02 -2.9004939e-02 -1.2093211e-02 -9.4539658e-03 -3.1604179e-02

1.4000000e+00 -4.7108790e-02 -2.1624305e-02 -1.1047318e-02 -3.2561559e-03 -2.4736118e-02 -2.0069814e-02 3.9591699e-03 1.1906271e-02 -8.4500540e-03 -1.3475324e-02 8.9962940e-03 1.5944320e-02 3.4719608e-03 2.1227741e-03 3.4569337e-02 5.8147243e-02 4.6205438e-02 5.1349216e-02 8.7089222e-02 1.3399478e-01 6.5544241e-02 -1.6445471e-01 -3.6997804e-01 -2.9630836e-01 -1.4194994e-01 -7.9482688e-02 -4.6510858e-02 -2.8644341e-02 -1.2740021e-02 -1.2134769e-02 -2.9164110e-02

1.6000000e+00 -3.6231907e-02 -1.6322743e-02 -1.0649694e-02 -3.3996463e-03 -2.3988122e-02 -1.9348280e-02 1.2184150e-03 8.5128612e-03 -1.1360017e-02 -1.2659604e-02 6.5087440e-03 1.5999026e-02 3.2952674e-03 2.1621808e-03 3.1873836e-02 5.4012991e-02 4.2689092e-02 5.0563606e-02 8.9660351e-02 1.3333350e-01 6.1087245e-02 -1.6870054e-01 -3.7056462e-01 -2.9043143e-01 -1.3646370e-01 -7.4054154e-02 -4.1986498e-02 -2.4756757e-02 -1.0096940e-02 -1.4008042e-02 -3.1014376e-02

1.8500000e+00 -3.2870596e-02 -1.6484278e-02 -1.1167536e-02 -4.4881019e-03 -2.1058674e-02 -1.7984250e-02 1.7522075e-03 7.4100980e-03 -1.1570813e-02 -9.1213963e-03 7.3331715e-03 1.5252410e-02 5.0915319e-03 5.8311105e-03 3.4548744e-02 5.3297391e-02 4.4824364e-02 5.3034670e-02 9.3317002e-02 1.3456208e-01 6.0680967e-02 -1.6981390e-01 -3.6568042e-01 -2.8275411e-01 -1.2209017e-01 -6.2088078e-02 -3.3310657e-02 -1.6974364e-02 -9.1516394e-03 -1.4836910e-02 -3.4501919e-02

2.1000000e+00 -3.6195473e-02 -1.7893138e-02 -9.7730143e-03 -3.9582813e-03 -1.9137415e-02 -1.7424853e-02 2.6730003e-03 6.3801099e-03 -1.0140193e-02 -6.5968773e-03 9.5964514e-03 1.7779552e-02 7.1742142e-03 7.8062542e-03 3.5565517e-02 5.0602209e-02 4.4927229e-02 5.4886833e-02 9.7513969e-02 1.3431961e-01 5.7490090e-02 -1.7091424e-01 -3.6016235e-01 -2.7044083e-01 -1.0592827e-01 -4.8657237e-02 -3.0111267e-02 -1.2976221e-02 -9.5636041e-03 -1.3671904e-02 -3.1149257e-02

2.3500000e+00 -4.1770242e-02 -2.2638293e-02 -1.1370326e-02 -7.5810155e-03 -2.1138662e-02 -1.4099857e-02 3.9485115e-03 5.5615965e-03 -8.3004139e-03 -6.7049617e-03 8.6382123e-03 1.4757068e-02 6.7742641e-03 9.3594920e-03 3.7075473e-02 4.9093959e-02 4.3176731e-02 5.5829930e-02 9.8792394e-02 1.3042332e-01 5.2351259e-02 -1.7321254e-01 -3.5517313e-01 -2.6243723e-01 -9.3833844e-02 -4.1419636e-02 -2.7193908e-02 -1.6628943e-02 -1.1023670e-02 -1.5667996e-02 -3.6507146e-02

2.6000000e+00 -4.1313537e-02 -2.3238088e-02 -1.1280869e-02 -5.7770738e-03 -2.1162848e-02 -1.2992960e-02 3.9435341e-03 4.3722854e-03 -8.1633465e-03 -7.4049623e-03 8.9045425e-03 1.5064643e-02 5.6521853e-03 8.7480485e-03 3.4275481e-02 4.7057763e-02 4.0743543e-02 5.6421497e-02 9.8822001e-02 1.2730046e-01 4.8104640e-02 -1.7410966e-01 -3.4955796e-01 -2.4833908e-01 -8.3442469e-02 -3.7058900e-02 -2.7964452e-02 -2.0383176e-02 -9.0617712e-03 -1.5965912e-02 -3.2857252e-02

2.8500000e+00 -3.9985197e-02 -2.6112500e-02 -1.1129758e-02 -8.3212512e-03 -2.2239209e-02 -9.5304836e-03 4.1237884e-03 4.2675129e-03 -6.1556942e-03 -6.6995085e-03 9.2344348e-03 1.5939206e-02 7.0285813e-03 1.1471372e-02 3.5174427e-02 4.8883295e-02 4.2448893e-02 5.9594503e-02 1.0190477e-01 1.2792972e-01 4.7127044e-02 -1.7188218e-01 -3.3953051e-01 -2.3103417e-01 -7.5199654e-02 -3.0587459e-02 -2.1412377e-02 -1.8061843e-02 -9.0606051e-03 -1.9893050e-02 -3.5985212e-02

3.1000000e+00 -3.9507820e-02 -2.3206503e-02 -1.1522988e-02 -7.8288964e-03 -1.8549802e-02 -9.5825650e-03 1.3096695e-03 1.8677991e-03 -7.3329016e-03 -7.6917829e-03 8.3128456e-03 1.4519714e-02 4.3995893e-03 9.0679441e-03 3.2224619e-02 4.7584491e-02 4.1873802e-02 5.9075829e-02 1.0217355e-01 1.2439863e-01 4.4163774e-02 -1.7149161e-01 -3.3090378e-01 -2.1608426e-01 -7.0339245e-02 -2.8112561e-02 -2.1012338e-02 -1.7343020e-02 -1.2421689e-02 -2.4471406e-02 -3.3525209e-02

3.3500000e+00 -3.5748708e-02 -1.8452284e-02 -8.5491693e-03 -5.3389952e-03 -1.7548344e-02 -6.1864168e-03 1.0362976e-03 1.8120224e-03 -6.2863094e-03 -5.0957229e-03 1.0706465e-02 1.3954309e-02 6.7142362e-03 1.1801045e-02 3.4431903e-02 4.7405678e-02 4.1368941e-02 5.8588628e-02 1.0222204e-01 1.2428018e-01 4.2895738e-02 -1.6740356e-01 -3.1393995e-01 -1.9960932e-01 -6.1344193e-02 -2.3545738e-02 -1.3867757e-02 -1.2935364e-02 -7.7793140e-03 -1.5994006e-02 -2.3058484e-02

3.7500000e+00 -3.1764755e-02 -1.7187322e-02 -1.0917703e-02 -6.5415747e-03 -1.6505233e-02 -5.6669240e-03 7.4815369e-04 2.9415136e-03 -5.2262862e-03 -3.1026446e-03 1.1489081e-02 1.2744575e-02 7.4748932e-03 1.1935463e-02 3.3448415e-02 4.6282584e-02 4.0982172e-02 5.8999082e-02 1.0066625e-01 1.1975031e-01 3.8642924e-02 -1.6394387e-01 -2.9508736e-01 -1.8630879e-01 -5.3306363e-02 -2.5637621e-02 -1.2716392e-02 -1.4167814e-02 -8.0890050e-03 -1.1572055e-02 -1.9495031e-02

4.1500000e+00 -3.2518396e-02 -2.0055214e-02 -1.2835118e-02 -8.3382989e-03 -1.8149890e-02 -6.2536404e-03 1.2450873e-03 7.3931271e-04 -6.9895626e-03 -4.3134236e-03 9.1738902e-03 9.9552503e-03 7.6232373e-03 1.0981140e-02 3.0088288e-02 4.2257383e-02 3.8721498e-02 5.6870410e-02 9.4228597e-02 1.1361324e-01 3.1195886e-02 -1.6408488e-01 -2.8178045e-01 -1.7729285e-01 -5.0275739e-02 -3.1832073e-02 -1.7990793e-02 -2.1183805e-02 -1.2188656e-02 -1.6137535e-02 -2.2446268e-02

4.5500000e+00 -3.7970942e-02 -2.7442312e-02 -1.6403633e-02 -1.2002784e-02 -1.6591920e-02 -5.4081423e-03 3.2751652e-03 1.8660032e-03 -5.9535790e-03 -3.8321515e-03 9.1264284e-03 1.1133014e-02 7.7158365e-03 1.1149834e-02 2.9161319e-02 4.0377312e-02 4.0414203e-02 5.7623752e-02 9.2169092e-02 1.0842572e-01 2.8044174e-02 -1.5651232e-01 -2.6463915e-01 -1.6275071e-01 -4.8823978e-02 -3.2597948e-02 -2.1603562e-02 -2.2539073e-02 -1.5956041e-02 -2.0132332e-02 -3.0818451e-02

4.9500000e+00 -3.8732498e-02 -2.6064699e-02 -1.7528071e-02 -1.2387934e-02 -1.6709885e-02 -2.9151278e-03 1.7828522e-03 2.3306237e-03 -6.9081937e-03 -2.2869308e-03 1.1202130e-02 1.2682419e-02 8.9417472e-03 1.1943300e-02 2.9307308e-02 3.7910708e-02 3.8780478e-02 5.7358303e-02 9.0046903e-02 1.0431104e-01 2.5425167e-02 -1.5230288e-01 -2.4965657e-01 -1.5127334e-01 -4.8078608e-02 -3.3230735e-02 -2.2132736e-02 -2.1914259e-02 -1.4817256e-02 -2.1234987e-02 -2.6331015e-02

5.3500000e+00 -3.2535228e-02 -2.5414251e-02 -1.6755671e-02 -1.0342208e-02 -1.7887853e-02 -4.0872067e-03 6.2118544e-04 2.3508334e-03 -7.2773672e-03 -1.5784696e-03 1.2494522e-02 1.5139320e-02 9.4513463e-03 1.2517405e-02 3.0459010e-02 3.4137049e-02 3.3019788e-02 5.4141529e-02 8.6668950e-02 9.8185765e-02 2.0734392e-02 -1.4521772e-01 -2.3777440e-01 -1.4177615e-01 -4.6471392e-02 -3.2975219e-02 -2.2514342e-02 -2.0610114e-02 -1.3432522e-02 -2.1213527e-02 -1.8740395e-02

5.7500000e+00 -2.5980101e-02 -2.1594186e-02 -1.6191973e-02 -9.3065823e-03 -1.7717408e-02 -5.8071172e-03 -7.8319057e-05 1.2910994e-03 -7.0706944e-03 -1.7088140e-03 1.1977012e-02 1.3735769e-02 9.0454943e-03 1.3154935e-02 3.1805217e-02 3.3725178e-02 2.8732699e-02 5.4092700e-02 8.4131143e-02 9.1815361e-02 1.8594059e-02 -1.3891402e-01 -2.2764710e-01 -1.3421403e-01 -4.4155311e-02 -3.4846050e-02 -2.1261674e-02 -1.9708241e-02 -1.2959778e-02 -1.3915950e-02 -1.5776393e-02

6.1500000e+00 -2.7076287e-02 -2.6990973e-02 -1.4448643e-02 -7.1380934e-03 -1.4412391e-02 -7.3455421e-03 2.8238360e-03 1.3601834e-03 -4.8923388e-03 -4.4291836e-03 1.0841247e-02 1.1111601e-02 8.2739075e-03 1.3623242e-02 3.2054060e-02 3.2933525e-02 2.7299180e-02 5.0713273e-02 8.0368095e-02 8.7104640e-02 1.5764942e-02 -1.3222793e-01 -2.2131853e-01 -1.2622039e-01 -4.2076003e-02 -3.6069822e-02 -2.5246276e-02 -1.7741093e-02 -1.1397306e-02 -8.6385812e-03 -2.0586560e-02

6.5500000e+00 -3.2605086e-02 -3.3143389e-02 -1.6622538e-02 -7.9076646e-03 -1.0952504e-02 -4.4764005e-03 5.4290281e-03 3.0072191e-03 -2.7803356e-03 -3.1994245e-03 1.1069882e-02 1.0084562e-02 8.1599763e-03 1.4083532e-02 3.1560682e-02 3.2973256e-02 2.9389127e-02 5.1172875e-02 7.9176455e-02 8.3407860e-02 1.7506456e-02 -1.2150902e-01 -2.0334080e-01 -1.1470141e-01 -3.8236679e-02 -3.5572061e-02 -2.3037321e-02 -1.5276189e-02 -1.1581031e-02 -2.6431834e-03 -2.8481621e-02

7.6380000e+00 -3.9032487e-02 -3.9496097e-02 -1.6848278e-02 -9.4509215e-03 -1.0553521e-02 -8.6950854e-04 8.8924107e-03 5.7972566e-03 -1.1172280e-03 -5.8857855e-04 1.2459722e-02 1.1427910e-02 9.5912712e-03 1.3273473e-02 3.0030896e-02 3.1319301e-02 2.9610159e-02 4.7592985e-02 7.4548003e-02 7.9473474e-02 1.5522371e-02 -1.1187997e-01 -1.8186949e-01 -1.0184813e-01 -3.6037085e-02 -2.8997213e-02 -2.2891996e-02 -1.3562549e-02 -6.3582898e-03 -6.8749400e-03 -2.5476857e-02

8.9070000e+00 -3.8931815e-02 -3.6553296e-02 -1.5664893e-02 -9.4979321e-03 -1.1353342e-02 6.0601357e-04 9.7013348e-03 5.4618814e-03 -2.4669461e-04 2.7416490e-03 1.3438010e-02 1.1438873e-02 8.0214406e-03 1.0589300e-02 2.7460755e-02 2.7422488e-02 2.4760562e-02 4.1438752e-02 6.7144209e-02 6.9484461e-02 1.2267741e-02 -1.0075801e-01 -1.5885247e-01 -9.2686970e-02 -3.3809443e-02 -2.8907470e-02 -2.0002258e-02 -1.4611316e-02 -5.5191192e-03 -7.9141104e-03 -1.8930369e-02

1.0387000e+01 -4.3379856e-02 -3.3154771e-02 -1.5229632e-02 -9.6712186e-03 -1.1410571e-02 1.6226699e-03 1.1649929e-02 6.4636935e-03 -7.6348664e-04 2.6303303e-03 1.4721811e-02 1.0680344e-02 7.0243726e-03 9.0743932e-03 2.5094619e-02 2.2882314e-02 2.0799456e-02 3.5058737e-02 5.7841228e-02 5.6834375e-02 8.9830039e-03 -9.2225020e-02 -1.4142879e-01 -8.5889694e-02 -3.2076748e-02 -2.9110931e-02 -2.0443519e-02 -1.4627085e-02 -2.0728365e-03 -1.1127946e-02 -1.4883221e-02

1.2113000e+01 -4.3960765e-02 -2.9865219e-02 -1.6045642e-02 -6.6188930e-03 -8.0407604e-03 3.9073716e-03 1.0785519e-02 6.6220954e-03 -1.1180878e-03 4.0721745e-03 1.5385147e-02 9.7774847e-03 4.5688997e-03 8.5028241e-03 2.2891743e-02 1.7391525e-02 1.4935424e-02 3.0708405e-02 4.9150151e-02 4.7690719e-02 7.9342141e-03 -8.2037218e-02 -1.2516439e-01 -7.6645070e-02 -2.7206661e-02 -2.9997187e-02 -1.7993973e-02 -1.3607304e-02 -2.3077669e-03 -6.5822441e-03 -7.5052534e-03

1.4125000e+01 -4.8176662e-02 -3.3440167e-02 -1.7714559e-02 -6.7897409e-03 -5.1102124e-03 6.3572653e-03 1.2861666e-02 6.0600284e-03 -2.1528761e-03 3.0687849e-03 1.2587237e-02 6.5807666e-03 1.9837137e-03 5.6454756e-03 2.0386836e-02 1.2074296e-02 9.7714086e-03 2.4352442e-02 4.0729711e-02 3.8531245e-02 5.0194503e-03 -7.7012557e-02 -1.1245328e-01 -7.0193530e-02 -2.6299389e-02 -2.9110247e-02 -1.4128121e-02 -1.1516324e-02 -1.2591325e-03 -6.2611218e-03 -8.8618335e-03

1.6472000e+01 -5.3420216e-02 -3.9346408e-02 -1.8203921e-02 -9.3149735e-03 -6.8023762e-03 6.9144624e-03 1.2179202e-02 4.3615670e-03 -2.9863199e-03 2.5477252e-03 9.6012793e-03 3.8233110e-03 -2.1391550e-03 1.8296278e-03 1.7702459e-02 9.7415216e-03 5.5017177e-03 2.1344996e-02 3.5047354e-02 3.4412167e-02 1.0831892e-03 -6.9771783e-02 -1.0003824e-01 -6.2892647e-02 -2.5353073e-02 -2.4407229e-02 -1.0342120e-02 -1.1298047e-02 6.0472440e-04 -2.1720357e-03 -6.3468583e-03

1.9209000e+01 -5.7030140e-02 -4.1265306e-02 -1.7228389e-02 -1.2688257e-02 -8.4033698e-03 5.7015469e-03 1.2907169e-02 3.4352377e-03 -5.0487762e-03 1.3056597e-03 5.6161372e-03 -2.8802736e-04 -3.5934617e-03 -8.7373928e-04 1.4939286e-02 7.5714783e-03 3.2327511e-03 1.6820733e-02 3.1272433e-02 2.9447905e-02 -3.2750847e-03 -6.6952062e-02 -8.7498843e-02 -5.7576870e-02 -2.1133213e-02 -1.9089570e-02 -6.5979357e-03 -6.8092665e-03 5.2975147e-03 -1.0204799e-03 -3.5447714e-03

2.2400000e+01 -5.8770920e-02 -3.8714704e-02 -1.9606096e-02 -9.2884266e-03 -3.8082855e-03 1.1500144e-02 1.4300414e-02 5.8718153e-03 -4.6939657e-03 1.9271569e-03 4.9056643e-03 -9.9490201e-04 -4.5759419e-03 -1.3287983e-03 1.3755241e-02 7.8012747e-03 2.8487876e-03 1.8075648e-02 2.9939439e-02 2.6038794e-02 -2.1202775e-03 -6.0509385e-02 -7.9343096e-02 -4.9387600e-02 -1.9836381e-02 -1.7382549e-02 -9.8415060e-03 -7.4666185e-03 2.9747848e-03 1.1637446e-03 4.2400314e-04

2.6122000e+01 -4.9454742e-02 -3.8817698e-02 -2.2268076e-02 -7.2979908e-03 3.1818824e-04 1.3693833e-02 1.5923721e-02 6.4677033e-03 -4.3439696e-03 3.0160209e-03 5.3963910e-03 -2.8304134e-03 -4.7858859e-03 -1.0357940e-03 1.0785485e-02 5.2456304e-03 -8.6762568e-04 1.3228502e-02 2.4511517e-02 2.1402970e-02 -2.8312038e-03 -5.7325358e-02 -7.3786466e-02 -4.2759792e-02 -2.0018889e-02 -1.9243602e-02 -9.8814029e-03 -3.7253902e-03 7.8470928e-04 4.5922063e-03 4.3974477e-03

3.0462000e+01 -4.8886826e-02 -4.2861350e-02 -2.7658619e-02 -8.4470508e-03 -2.3698381e-04 1.3710983e-02 1.4504991e-02 6.4852510e-03 -2.9026245e-03 2.4517098e-03 6.3009966e-03 -3.3522929e-03 -4.9032936e-03 -2.0487487e-03 9.5503221e-03 4.0682872e-03 8.0356899e-04 1.4676641e-02 2.2471029e-02 1.9296639e-02 -4.3574514e-05 -5.1180944e-02 -6.8695090e-02 -3.8626257e-02 -1.9443646e-02 -1.8945418e-02 -8.6110245e-03 -1.8028807e-03 -3.4030293e-03 7.1285242e-03 6.6548097e-03

3.5523000e+01 -4.5739640e-02 -4.3623991e-02 -2.5830336e-02 -8.2924584e-03 -1.0197308e-03 1.2130884e-02 1.5735830e-02 5.2966211e-03 -2.2228256e-03 2.6998067e-03 5.5008993e-03 -3.6081034e-03 -5.0810297e-03 -2.5916485e-03 7.1903285e-03 1.1605786e-03 -1.6721668e-03 1.0522543e-02 1.7805876e-02 1.4734717e-02 -5.6046685e-03 -5.1458301e-02 -6.4722489e-02 -3.8473163e-02 -1.9247879e-02 -1.8255816e-02 -5.8917794e-03 -1.1518691e-03 3.5618734e-04 5.5066994e-03 8.8130131e-03

4.1425000e+01 -5.3468909e-02 -4.3356110e-02 -2.5167555e-02 -9.7743757e-03 -1.8629575e-03 1.3147723e-02 1.5213097e-02 6.3736654e-03 -1.9650007e-03 2.5734033e-03 3.8212470e-03 -2.9967037e-03 -5.4602882e-03 -2.9826199e-03 7.4389119e-03 1.1629741e-03 -2.0604560e-04 1.0675960e-02 1.7980483e-02 1.4582397e-02 -5.7827066e-03 -5.1360859e-02 -6.3105043e-02 -3.6123199e-02 -1.5454558e-02 -1.5415640e-02 -8.0569263e-03 1.3451425e-03 3.2468306e-03 7.5627734e-03 2.9306427e-03

4.8307000e+01 -5.4841477e-02 -4.5755863e-02 -2.5173500e-02 -9.9868565e-03 -1.2933258e-03 1.5853444e-02 1.8636210e-02 5.5054685e-03 -2.0905477e-03 3.9401268e-03 3.8121899e-03 -3.9002643e-03 -5.7090802e-03 -1.7417093e-03 6.5647877e-03 2.6980730e-03 -1.8301368e-03 8.8649278e-03 1.9562421e-02 1.3672546e-02 -7.6374433e-03 -5.1040582e-02 -6.3209858e-02 -3.3315215e-02 -1.3841219e-02 -1.6221063e-02 -8.0315643e-03 3.2354997e-03 5.6613899e-03 1.0477738e-02 1.9018796e-03

5.6333000e+01 -5.6062521e-02 -4.4193554e-02 -2.3240378e-02 -9.1238094e-03 -5.4992258e-04 1.7568286e-02 1.7784894e-02 6.6500513e-03 -1.6353577e-03 5.0439538e-03 4.5056710e-03 -4.8164577e-03 -6.5379552e-03 -3.0592849e-03 4.8448146e-03 3.0066608e-03 -2.3853738e-03 8.7034695e-03 1.7087001e-02 1.2656081e-02 -8.3033803e-03 -5.1014428e-02 -6.2216392e-02 -3.0754893e-02 -1.0341172e-02 -1.6075765e-02 -7.6631634e-03 3.0158460e-03 4.5588492e-03 1.1340470e-02 3.2887024e-03

6.5692000e+01 -4.7005525e-02 -4.1026086e-02 -2.0515683e-02 -5.9558038e-03 2.7434780e-03 1.9463164e-02 2.1156996e-02 7.4061233e-03 1.9442877e-04 5.1758644e-03 7.0774471e-03 -4.3804090e-03 -6.9528951e-03 -1.9721425e-03 6.1561089e-03 1.7117564e-04 -2.9935712e-03 8.7039593e-03 1.5250910e-02 1.1132473e-02 -8.8321104e-03 -5.2041218e-02 -6.1622847e-02 -3.1150954e-02 -1.2173781e-02 -1.7797530e-02 -4.4843611e-03 -1.7170622e-03 3.0972854e-03 8.6869713e-03 1.1806988e-02

7.6606000e+01 -4.5163215e-02 -3.6590729e-02 -2.0010423e-02 -4.2646054e-03 5.8371652e-03 2.4351410e-02 2.4023729e-02 1.0646462e-02 3.0670243e-03 4.7909005e-03 7.2866446e-03 -2.3124564e-03 -8.6927174e-03 -4.3060572e-03 6.6006662e-03 -1.7796794e-03 -4.3528354e-03 8.5005125e-03 1.2079399e-02 1.1302580e-02 -9.4168081e-03 -5.4800242e-02 -6.3011152e-02 -3.1178922e-02 -1.0621888e-02 -1.7134344e-02 -4.7032077e-03 -3.2235262e-04 3.2413868e-03 8.5178828e-03 1.1695713e-02

8.9333000e+01 -4.2586569e-02 -3.7698422e-02 -2.6311673e-02 -7.9429906e-03 6.8093727e-03 2.5922912e-02 2.4243869e-02 1.1876559e-02 2.1087064e-03 4.3861050e-03 6.7879527e-03 -3.1634857e-03 -8.5378806e-03 -3.8738387e-03 7.3074278e-03 -3.9475671e-03 -4.7499180e-03 7.7060032e-03 1.0083546e-02 1.1467446e-02 -8.5085092e-03 -5.5053149e-02 -6.5872255e-02 -3.4834893e-02 -1.1942750e-02 -1.5472803e-02 -4.7906514e-03 1.2221485e-03 3.9714137e-03 8.0683353e-03 7.4641429e-03

1.0417500e+02 -5.0456533e-02 -4.1960550e-02 -3.0501570e-02 -8.6112808e-03 5.0123788e-03 2.4191885e-02 2.2632460e-02 1.1559735e-02 1.0880829e-03 4.1132808e-03 4.6621762e-03 -5.4926495e-03 -9.0892157e-03 -8.1108118e-03 5.8256682e-03 -4.0159433e-03 -5.6407556e-03 6.8261786e-03 9.6818614e-03 9.4970149e-03 -9.8622535e-03 -5.5237453e-02 -6.7501710e-02 -3.4643771e-02 -7.3476681e-03 -1.2937057e-02 -2.3382137e-03 3.3979587e-03 5.0719338e-03 7.0585580e-03 7.1097173e-03

1.2148300e+02 -4.9258801e-02 -4.4684232e-02 -2.9100701e-02 -8.3932317e-03 6.8395726e-03 2.4018189e-02 2.2720839e-02 9.8591559e-03 -1.3261544e-03 2.9973531e-03 5.8361928e-03 -7.3850532e-03 -8.7970705e-03 -6.7184811e-03 6.7248213e-03 -5.0589969e-03 -6.9627111e-03 4.6289596e-03 9.3066846e-03 7.7688532e-03 -1.1572427e-02 -5.4522664e-02 -6.8960318e-02 -3.4697802e-02 -6.9798130e-03 -1.1515340e-02 -5.5899333e-04 9.1365927e-04 9.2260911e-03 6.6401492e-03 7.5267196e-03

1.4166700e+02 -6.2873047e-02 -5.0159433e-02 -3.1971485e-02 -8.9508663e-03 4.6535090e-03 2.4098474e-02 2.4607608e-02 9.6748988e-03 -6.3492660e-04 2.2819314e-03 6.0506745e-03 -6.9387617e-03 -9.3910435e-03 -6.8721071e-03 5.4375059e-03 -4.7946866e-03 -6.3131091e-03 5.7592037e-03 1.2695938e-02 8.7884994e-03 -1.1889654e-02 -5.4507350e-02 -6.9563697e-02 -3.2536761e-02 -3.8198913e-03 -1.0944062e-02 2.4981313e-03 -8.0199366e-05 9.0924330e-03 3.2595554e-03 9.9577336e-03

1.6520400e+02 -6.7027841e-02 -5.2496741e-02 -3.3539177e-02 -9.9991441e-03 8.5294486e-03 2.7518438e-02 2.5794740e-02 9.7440112e-03 7.7131755e-04 1.8864611e-03 4.3826467e-03 -7.5421868e-03 -1.0517036e-02 -6.5518339e-03 3.1495474e-03 -6.0613714e-03 -9.1550118e-03 4.3815940e-03 1.5509468e-02 9.0466863e-03 -1.2284959e-02 -5.4847957e-02 -6.6929084e-02 -2.9690401e-02 -4.0285890e-03 -1.0319286e-02 -7.0753650e-05 1.6057906e-03 1.1030835e-02 7.4005764e-03 9.1319149e-03

1.9265100e+02 -8.0089901e-02 -6.0476368e-02 -4.1373667e-02 -1.2544742e-02 7.7628793e-03 2.8523414e-02 2.5399156e-02 1.1249810e-02 9.4781689e-04 7.8235494e-04 8.2933775e-04 -8.6642553e-03 -1.1831714e-02 -8.5469334e-03 6.0158636e-04 -6.5815972e-03 -9.1258695e-03 4.3864393e-03 1.5560719e-02 9.4194128e-03 -1.1476321e-02 -5.7070940e-02 -6.5566854e-02 -2.8311761e-02 -2.8476462e-03 -9.5481169e-03 1.3559441e-04 2.6669640e-03 1.3129842e-02 5.8384505e-03 9.9147174e-03

2.2465800e+02 -7.6744394e-02 -6.3683962e-02 -3.7774690e-02 -1.2074177e-02 1.2909727e-02 3.1382254e-02 2.9359041e-02 1.2430804e-02 4.1599952e-03 1.1534704e-03 2.5524869e-03 -7.5839656e-03 -1.2329500e-02 -7.8733357e-03 5.0103755e-04 -5.7688138e-03 -9.7495993e-03 5.1236931e-03 1.4819462e-02 8.5984919e-03 -1.1550383e-02 -5.9995947e-02 -6.7694601e-02 -2.4107368e-02 -1.7975591e-03 -7.8031088e-03 -1.6908088e-05 4.1448595e-03 1.5623718e-02 1.2116229e-02 4.7326607e-03

2.6198300e+02 -8.4328163e-02 -6.6759708e-02 -4.3504336e-02 -1.5780953e-02 1.2964948e-02 3.5365284e-02 3.2742385e-02 1.3661986e-02 3.2222272e-03 2.4843186e-03 4.2178705e-03 -6.7137747e-03 -1.3091211e-02 -7.5976388e-03 1.0868030e-03 -4.2156345e-03 -8.3409770e-03 6.1164710e-03 1.2515125e-02 9.0677844e-03 -1.2385367e-02 -6.1499096e-02 -6.8497125e-02 -2.3597461e-02 -1.2860677e-03 -9.5101981e-03 1.7139365e-03 7.1311663e-03 1.4719028e-02 1.4816097e-02 2.3143201e-03

3.0551000e+02 -8.4497914e-02 -6.8797360e-02 -4.1800379e-02 -1.7376795e-02 1.1914317e-02 3.5443621e-02 3.3720859e-02 1.3717510e-02 1.6984543e-03 2.8719758e-03 4.4447560e-03 -7.4767932e-03 -1.5573531e-02 -9.9685919e-03 -2.9338318e-04 -6.1502572e-03 -1.1170178e-02 3.5202771e-03 1.0868249e-02 7.1645949e-03 -1.6515445e-02 -6.3196250e-02 -6.6347488e-02 -2.3619780e-02 -3.1503902e-04 -8.3608006e-03 2.4661065e-03 8.6205604e-03 1.0495478e-02 1.6907148e-02 1.3032277e-03

3.5626700e+02 -7.8360464e-02 -6.4529789e-02 -3.7450156e-02 -1.1939421e-02 1.5813636e-02 4.3790648e-02 3.8910991e-02 1.7489776e-02 2.7031895e-03 5.5261496e-03 6.6503084e-03 -5.4572843e-03 -1.4679183e-02 -9.4878819e-03 4.2751707e-04 -7.7916977e-03 -1.1773183e-02 1.8561267e-03 1.2431346e-02 7.5796437e-03 -1.8196765e-02 -6.1463282e-02 -6.1365264e-02 -2.1335021e-02 6.7418122e-04 -6.5242159e-03 2.9782726e-03 1.1477505e-02 1.3401045e-02 1.4996495e-02 6.2658285e-03

4.1545800e+02 -7.7087870e-02 -6.7686657e-02 -3.3481265e-02 -9.0441981e-03 1.9187120e-02 4.5815674e-02 4.0711193e-02 1.7448474e-02 3.1117223e-03 3.2202068e-03 4.0379310e-03 -9.0577048e-03 -1.6310238e-02 -1.1025147e-02 1.2781577e-04 -1.1119378e-02 -1.3684704e-02 5.9691747e-04 1.0373951e-02 4.7465778e-03 -2.2067639e-02 -6.3064420e-02 -5.9730443e-02 -2.0099005e-02 3.7909745e-03 -3.9673837e-03 4.2107505e-03 8.7785807e-03 1.2971683e-02 8.5640033e-03 1.0353194e-02

4.8448300e+02 -8.3266202e-02 -6.9333902e-02 -3.4840265e-02 -6.8800250e-03 2.4158496e-02 4.7257049e-02 4.1119457e-02 1.4558949e-02 3.4454841e-03 2.5750901e-03 3.2218573e-03 -1.0935395e-02 -1.7987456e-02 -1.0934387e-02 -3.4086144e-04 -1.0725498e-02 -1.3321196e-02 1.9431982e-03 9.4758882e-03 2.2974591e-03 -2.1787790e-02 -6.1806180e-02 -5.6766654e-02 -1.3786112e-02 6.5959209e-03 -5.0540373e-03 4.1565699e-03 6.5843382e-03 1.7240568e-02 5.4905955e-03 1.2506461e-02

5.6497600e+02 -9.9792674e-02 -7.5493103e-02 -3.9732136e-02 -7.3902881e-03 2.7530827e-02 4.4892667e-02 3.7718756e-02 1.1605016e-02 2.3345984e-03 3.0497169e-03 5.7114313e-04 -1.4789627e-02 -2.0607707e-02 -1.1460493e-02 1.7300076e-03 -7.3361623e-03 -1.1771744e-02 4.6725685e-03 7.6006294e-03 4.9417037e-04 -1.9428366e-02 -5.9279661e-02 -4.7474520e-02 -7.0932105e-03 1.2149375e-02 -5.4846181e-03 5.5234913e-03 4.5909219e-03 1.7663093e-02 6.2149601e-03 1.3640676e-02

Fig. 2c

2.0000000e-01 -3.0089316e-01 -2.1347355e-01 -1.5030565e-01 1.3071972e-02 -1.9662236e-01 -4.1443325e-01 -1.5127285e-01 2.3994633e-01 1.5418612e-01 -1.3876598e-01 3.6348105e-02 3.3813000e-01 2.3705393e-01 1.2152590e-02 1.6322262e-01 5.3424384e-01 4.3141223e-01 2.2190397e-01 1.8651398e-01 4.1586905e-01 4.5751039e-01 5.0427167e-02 -3.1055286e-01 -2.0454446e-01 2.4998079e-02 -5.0929037e-02 -2.1857043e-01 -2.5801919e-01 -1.7876523e-01 -1.9356256e-01 -3.3830514e-01

4.0000000e-01 -2.6330387e-01 -1.8098172e-01 -1.1787528e-01 -2.1925912e-02 -1.2876916e-01 -2.1880999e-01 -5.8848505e-02 1.5389877e-01 9.6493556e-02 -5.1186829e-02 6.2301872e-02 2.2341386e-01 1.5663732e-01 4.4155715e-02 1.3993385e-01 3.4825551e-01 2.8666749e-01 1.6773760e-01 1.7182567e-01 3.1526224e-01 2.9933154e-01 -2.3263480e-02 -3.0458447e-01 -2.2029434e-01 -3.8616304e-02 -5.6607783e-02 -1.3149980e-01 -1.4880862e-01 -9.8117061e-02 -1.1108885e-01 -1.9599293e-01

6.0000000e-01 -2.1616302e-01 -1.4651817e-01 -8.5087450e-02 -3.9250627e-02 -7.8674923e-02 -8.5479033e-02 1.1392193e-03 8.2816547e-02 4.8285541e-02 2.6493408e-03 6.9051634e-02 1.2838400e-01 8.8517899e-02 5.4864367e-02 1.1068199e-01 2.0281927e-01 1.7086593e-01 1.2195260e-01 1.5213316e-01 2.3327107e-01 1.7794437e-01 -7.9425008e-02 -3.0729184e-01 -2.3768468e-01 -8.8118145e-02 -6.1832925e-02 -7.0460122e-02 -6.8922574e-02 -3.2934185e-02 -4.3936597e-02 -9.3389848e-02

8.0000000e-01 -1.3244822e-01 -8.3206274e-02 -4.0260183e-02 -1.9000345e-02 -4.6939532e-02 -4.5380221e-02 7.1630761e-03 4.4891808e-02 1.5184722e-02 -4.2011354e-03 3.6424583e-02 6.4638524e-02 3.5507972e-02 2.5181845e-02 6.6927700e-02 1.1649940e-01 9.7459772e-02 7.5525963e-02 1.0992075e-01 1.6764522e-01 1.0727177e-01 -1.3116589e-01 -3.4418175e-01 -2.7628648e-01 -1.3493878e-01 -8.9163033e-02 -6.6376972e-02 -5.4224025e-02 -2.4482303e-02 -2.5307937e-02 -6.2963744e-02

1.0000000e+00 -8.8494320e-02 -5.1666198e-02 -1.9142335e-02 -7.5939151e-03 -3.3348458e-02 -3.2318488e-02 7.1727993e-03 2.6864207e-02 2.4138664e-03 -8.2364264e-03 2.2752121e-02 3.6387631e-02 1.6058224e-02 1.2119016e-02 4.9202455e-02 8.3361654e-02 6.9098720e-02 6.1553760e-02 9.5501211e-02 1.4858051e-01 8.6000420e-02 -1.4439863e-01 -3.5361520e-01 -2.8537755e-01 -1.3920791e-01 -8.4081853e-02 -5.4527971e-02 -3.4414841e-02 -9.8287128e-03 -6.8368395e-03 -3.9355474e-02

1.2000000e+00 -6.4823803e-02 -3.0639699e-02 -1.1564700e-02 -4.6723872e-03 -2.7391664e-02 -2.3665086e-02 4.8181405e-03 1.7562487e-02 -4.3318949e-03 -1.2642841e-02 1.2882159e-02 2.3528960e-02 7.2377603e-03 5.1314382e-03 4.0153881e-02 6.6172988e-02 5.3615001e-02 5.3278838e-02 8.9164760e-02 1.3803937e-01 7.2824950e-02 -1.5766021e-01 -3.6516749e-01 -2.9335591e-01 -1.4325248e-01 -8.3731569e-02 -4.9392500e-02 -2.9004939e-02 -1.2093211e-02 -9.4539658e-03 -3.1604179e-02

1.4000000e+00 -4.7108790e-02 -2.1624305e-02 -1.1047318e-02 -3.2561559e-03 -2.4736118e-02 -2.0069814e-02 3.9591699e-03 1.1906271e-02 -8.4500540e-03 -1.3475324e-02 8.9962940e-03 1.5944320e-02 3.4719608e-03 2.1227741e-03 3.4569337e-02 5.8147243e-02 4.6205438e-02 5.1349216e-02 8.7089222e-02 1.3399478e-01 6.5544241e-02 -1.6445471e-01 -3.6997804e-01 -2.9630836e-01 -1.4194994e-01 -7.9482688e-02 -4.6510858e-02 -2.8644341e-02 -1.2740021e-02 -1.2134769e-02 -2.9164110e-02

1.6000000e+00 -3.6231907e-02 -1.6322743e-02 -1.0649694e-02 -3.3996463e-03 -2.3988122e-02 -1.9348280e-02 1.2184150e-03 8.5128612e-03 -1.1360017e-02 -1.2659604e-02 6.5087440e-03 1.5999026e-02 3.2952674e-03 2.1621808e-03 3.1873836e-02 5.4012991e-02 4.2689092e-02 5.0563606e-02 8.9660351e-02 1.3333350e-01 6.1087245e-02 -1.6870054e-01 -3.7056462e-01 -2.9043143e-01 -1.3646370e-01 -7.4054154e-02 -4.1986498e-02 -2.4756757e-02 -1.0096940e-02 -1.4008042e-02 -3.1014376e-02

1.8500000e+00 -3.2870596e-02 -1.6484278e-02 -1.1167536e-02 -4.4881019e-03 -2.1058674e-02 -1.7984250e-02 1.7522075e-03 7.4100980e-03 -1.1570813e-02 -9.1213963e-03 7.3331715e-03 1.5252410e-02 5.0915319e-03 5.8311105e-03 3.4548744e-02 5.3297391e-02 4.4824364e-02 5.3034670e-02 9.3317002e-02 1.3456208e-01 6.0680967e-02 -1.6981390e-01 -3.6568042e-01 -2.8275411e-01 -1.2209017e-01 -6.2088078e-02 -3.3310657e-02 -1.6974364e-02 -9.1516394e-03 -1.4836910e-02 -3.4501919e-02

2.1000000e+00 -3.6195473e-02 -1.7893138e-02 -9.7730143e-03 -3.9582813e-03 -1.9137415e-02 -1.7424853e-02 2.6730003e-03 6.3801099e-03 -1.0140193e-02 -6.5968773e-03 9.5964514e-03 1.7779552e-02 7.1742142e-03 7.8062542e-03 3.5565517e-02 5.0602209e-02 4.4927229e-02 5.4886833e-02 9.7513969e-02 1.3431961e-01 5.7490090e-02 -1.7091424e-01 -3.6016235e-01 -2.7044083e-01 -1.0592827e-01 -4.8657237e-02 -3.0111267e-02 -1.2976221e-02 -9.5636041e-03 -1.3671904e-02 -3.1149257e-02

2.3500000e+00 -4.1770242e-02 -2.2638293e-02 -1.1370326e-02 -7.5810155e-03 -2.1138662e-02 -1.4099857e-02 3.9485115e-03 5.5615965e-03 -8.3004139e-03 -6.7049617e-03 8.6382123e-03 1.4757068e-02 6.7742641e-03 9.3594920e-03 3.7075473e-02 4.9093959e-02 4.3176731e-02 5.5829930e-02 9.8792394e-02 1.3042332e-01 5.2351259e-02 -1.7321254e-01 -3.5517313e-01 -2.6243723e-01 -9.3833844e-02 -4.1419636e-02 -2.7193908e-02 -1.6628943e-02 -1.1023670e-02 -1.5667996e-02 -3.6507146e-02

2.6000000e+00 -4.1313537e-02 -2.3238088e-02 -1.1280869e-02 -5.7770738e-03 -2.1162848e-02 -1.2992960e-02 3.9435341e-03 4.3722854e-03 -8.1633465e-03 -7.4049623e-03 8.9045425e-03 1.5064643e-02 5.6521853e-03 8.7480485e-03 3.4275481e-02 4.7057763e-02 4.0743543e-02 5.6421497e-02 9.8822001e-02 1.2730046e-01 4.8104640e-02 -1.7410966e-01 -3.4955796e-01 -2.4833908e-01 -8.3442469e-02 -3.7058900e-02 -2.7964452e-02 -2.0383176e-02 -9.0617712e-03 -1.5965912e-02 -3.2857252e-02

2.8500000e+00 -3.9985197e-02 -2.6112500e-02 -1.1129758e-02 -8.3212512e-03 -2.2239209e-02 -9.5304836e-03 4.1237884e-03 4.2675129e-03 -6.1556942e-03 -6.6995085e-03 9.2344348e-03 1.5939206e-02 7.0285813e-03 1.1471372e-02 3.5174427e-02 4.8883295e-02 4.2448893e-02 5.9594503e-02 1.0190477e-01 1.2792972e-01 4.7127044e-02 -1.7188218e-01 -3.3953051e-01 -2.3103417e-01 -7.5199654e-02 -3.0587459e-02 -2.1412377e-02 -1.8061843e-02 -9.0606051e-03 -1.9893050e-02 -3.5985212e-02

3.1000000e+00 -3.9507820e-02 -2.3206503e-02 -1.1522988e-02 -7.8288964e-03 -1.8549802e-02 -9.5825650e-03 1.3096695e-03 1.8677991e-03 -7.3329016e-03 -7.6917829e-03 8.3128456e-03 1.4519714e-02 4.3995893e-03 9.0679441e-03 3.2224619e-02 4.7584491e-02 4.1873802e-02 5.9075829e-02 1.0217355e-01 1.2439863e-01 4.4163774e-02 -1.7149161e-01 -3.3090378e-01 -2.1608426e-01 -7.0339245e-02 -2.8112561e-02 -2.1012338e-02 -1.7343020e-02 -1.2421689e-02 -2.4471406e-02 -3.3525209e-02

3.3500000e+00 -3.5748708e-02 -1.8452284e-02 -8.5491693e-03 -5.3389952e-03 -1.7548344e-02 -6.1864168e-03 1.0362976e-03 1.8120224e-03 -6.2863094e-03 -5.0957229e-03 1.0706465e-02 1.3954309e-02 6.7142362e-03 1.1801045e-02 3.4431903e-02 4.7405678e-02 4.1368941e-02 5.8588628e-02 1.0222204e-01 1.2428018e-01 4.2895738e-02 -1.6740356e-01 -3.1393995e-01 -1.9960932e-01 -6.1344193e-02 -2.3545738e-02 -1.3867757e-02 -1.2935364e-02 -7.7793140e-03 -1.5994006e-02 -2.3058484e-02

3.7500000e+00 -3.1764755e-02 -1.7187322e-02 -1.0917703e-02 -6.5415747e-03 -1.6505233e-02 -5.6669240e-03 7.4815369e-04 2.9415136e-03 -5.2262862e-03 -3.1026446e-03 1.1489081e-02 1.2744575e-02 7.4748932e-03 1.1935463e-02 3.3448415e-02 4.6282584e-02 4.0982172e-02 5.8999082e-02 1.0066625e-01 1.1975031e-01 3.8642924e-02 -1.6394387e-01 -2.9508736e-01 -1.8630879e-01 -5.3306363e-02 -2.5637621e-02 -1.2716392e-02 -1.4167814e-02 -8.0890050e-03 -1.1572055e-02 -1.9495031e-02

4.1500000e+00 -3.2518396e-02 -2.0055214e-02 -1.2835118e-02 -8.3382989e-03 -1.8149890e-02 -6.2536404e-03 1.2450873e-03 7.3931271e-04 -6.9895626e-03 -4.3134236e-03 9.1738902e-03 9.9552503e-03 7.6232373e-03 1.0981140e-02 3.0088288e-02 4.2257383e-02 3.8721498e-02 5.6870410e-02 9.4228597e-02 1.1361324e-01 3.1195886e-02 -1.6408488e-01 -2.8178045e-01 -1.7729285e-01 -5.0275739e-02 -3.1832073e-02 -1.7990793e-02 -2.1183805e-02 -1.2188656e-02 -1.6137535e-02 -2.2446268e-02

4.5500000e+00 -3.7970942e-02 -2.7442312e-02 -1.6403633e-02 -1.2002784e-02 -1.6591920e-02 -5.4081423e-03 3.2751652e-03 1.8660032e-03 -5.9535790e-03 -3.8321515e-03 9.1264284e-03 1.1133014e-02 7.7158365e-03 1.1149834e-02 2.9161319e-02 4.0377312e-02 4.0414203e-02 5.7623752e-02 9.2169092e-02 1.0842572e-01 2.8044174e-02 -1.5651232e-01 -2.6463915e-01 -1.6275071e-01 -4.8823978e-02 -3.2597948e-02 -2.1603562e-02 -2.2539073e-02 -1.5956041e-02 -2.0132332e-02 -3.0818451e-02

4.9500000e+00 -3.8732498e-02 -2.6064699e-02 -1.7528071e-02 -1.2387934e-02 -1.6709885e-02 -2.9151278e-03 1.7828522e-03 2.3306237e-03 -6.9081937e-03 -2.2869308e-03 1.1202130e-02 1.2682419e-02 8.9417472e-03 1.1943300e-02 2.9307308e-02 3.7910708e-02 3.8780478e-02 5.7358303e-02 9.0046903e-02 1.0431104e-01 2.5425167e-02 -1.5230288e-01 -2.4965657e-01 -1.5127334e-01 -4.8078608e-02 -3.3230735e-02 -2.2132736e-02 -2.1914259e-02 -1.4817256e-02 -2.1234987e-02 -2.6331015e-02

5.3500000e+00 -3.2535228e-02 -2.5414251e-02 -1.6755671e-02 -1.0342208e-02 -1.7887853e-02 -4.0872067e-03 6.2118544e-04 2.3508334e-03 -7.2773672e-03 -1.5784696e-03 1.2494522e-02 1.5139320e-02 9.4513463e-03 1.2517405e-02 3.0459010e-02 3.4137049e-02 3.3019788e-02 5.4141529e-02 8.6668950e-02 9.8185765e-02 2.0734392e-02 -1.4521772e-01 -2.3777440e-01 -1.4177615e-01 -4.6471392e-02 -3.2975219e-02 -2.2514342e-02 -2.0610114e-02 -1.3432522e-02 -2.1213527e-02 -1.8740395e-02

5.7500000e+00 -2.5980101e-02 -2.1594186e-02 -1.6191973e-02 -9.3065823e-03 -1.7717408e-02 -5.8071172e-03 -7.8319057e-05 1.2910994e-03 -7.0706944e-03 -1.7088140e-03 1.1977012e-02 1.3735769e-02 9.0454943e-03 1.3154935e-02 3.1805217e-02 3.3725178e-02 2.8732699e-02 5.4092700e-02 8.4131143e-02 9.1815361e-02 1.8594059e-02 -1.3891402e-01 -2.2764710e-01 -1.3421403e-01 -4.4155311e-02 -3.4846050e-02 -2.1261674e-02 -1.9708241e-02 -1.2959778e-02 -1.3915950e-02 -1.5776393e-02

6.1500000e+00 -2.7076287e-02 -2.6990973e-02 -1.4448643e-02 -7.1380934e-03 -1.4412391e-02 -7.3455421e-03 2.8238360e-03 1.3601834e-03 -4.8923388e-03 -4.4291836e-03 1.0841247e-02 1.1111601e-02 8.2739075e-03 1.3623242e-02 3.2054060e-02 3.2933525e-02 2.7299180e-02 5.0713273e-02 8.0368095e-02 8.7104640e-02 1.5764942e-02 -1.3222793e-01 -2.2131853e-01 -1.2622039e-01 -4.2076003e-02 -3.6069822e-02 -2.5246276e-02 -1.7741093e-02 -1.1397306e-02 -8.6385812e-03 -2.0586560e-02

6.5500000e+00 -3.2605086e-02 -3.3143389e-02 -1.6622538e-02 -7.9076646e-03 -1.0952504e-02 -4.4764005e-03 5.4290281e-03 3.0072191e-03 -2.7803356e-03 -3.1994245e-03 1.1069882e-02 1.0084562e-02 8.1599763e-03 1.4083532e-02 3.1560682e-02 3.2973256e-02 2.9389127e-02 5.1172875e-02 7.9176455e-02 8.3407860e-02 1.7506456e-02 -1.2150902e-01 -2.0334080e-01 -1.1470141e-01 -3.8236679e-02 -3.5572061e-02 -2.3037321e-02 -1.5276189e-02 -1.1581031e-02 -2.6431834e-03 -2.8481621e-02

7.6380000e+00 -3.9032487e-02 -3.9496097e-02 -1.6848278e-02 -9.4509215e-03 -1.0553521e-02 -8.6950854e-04 8.8924107e-03 5.7972566e-03 -1.1172280e-03 -5.8857855e-04 1.2459722e-02 1.1427910e-02 9.5912712e-03 1.3273473e-02 3.0030896e-02 3.1319301e-02 2.9610159e-02 4.7592985e-02 7.4548003e-02 7.9473474e-02 1.5522371e-02 -1.1187997e-01 -1.8186949e-01 -1.0184813e-01 -3.6037085e-02 -2.8997213e-02 -2.2891996e-02 -1.3562549e-02 -6.3582898e-03 -6.8749400e-03 -2.5476857e-02

8.9070000e+00 -3.8931815e-02 -3.6553296e-02 -1.5664893e-02 -9.4979321e-03 -1.1353342e-02 6.0601357e-04 9.7013348e-03 5.4618814e-03 -2.4669461e-04 2.7416490e-03 1.3438010e-02 1.1438873e-02 8.0214406e-03 1.0589300e-02 2.7460755e-02 2.7422488e-02 2.4760562e-02 4.1438752e-02 6.7144209e-02 6.9484461e-02 1.2267741e-02 -1.0075801e-01 -1.5885247e-01 -9.2686970e-02 -3.3809443e-02 -2.8907470e-02 -2.0002258e-02 -1.4611316e-02 -5.5191192e-03 -7.9141104e-03 -1.8930369e-02

1.0387000e+01 -4.3379856e-02 -3.3154771e-02 -1.5229632e-02 -9.6712186e-03 -1.1410571e-02 1.6226699e-03 1.1649929e-02 6.4636935e-03 -7.6348664e-04 2.6303303e-03 1.4721811e-02 1.0680344e-02 7.0243726e-03 9.0743932e-03 2.5094619e-02 2.2882314e-02 2.0799456e-02 3.5058737e-02 5.7841228e-02 5.6834375e-02 8.9830039e-03 -9.2225020e-02 -1.4142879e-01 -8.5889694e-02 -3.2076748e-02 -2.9110931e-02 -2.0443519e-02 -1.4627085e-02 -2.0728365e-03 -1.1127946e-02 -1.4883221e-02

1.2113000e+01 -4.3960765e-02 -2.9865219e-02 -1.6045642e-02 -6.6188930e-03 -8.0407604e-03 3.9073716e-03 1.0785519e-02 6.6220954e-03 -1.1180878e-03 4.0721745e-03 1.5385147e-02 9.7774847e-03 4.5688997e-03 8.5028241e-03 2.2891743e-02 1.7391525e-02 1.4935424e-02 3.0708405e-02 4.9150151e-02 4.7690719e-02 7.9342141e-03 -8.2037218e-02 -1.2516439e-01 -7.6645070e-02 -2.7206661e-02 -2.9997187e-02 -1.7993973e-02 -1.3607304e-02 -2.3077669e-03 -6.5822441e-03 -7.5052534e-03

1.4125000e+01 -4.8176662e-02 -3.3440167e-02 -1.7714559e-02 -6.7897409e-03 -5.1102124e-03 6.3572653e-03 1.2861666e-02 6.0600284e-03 -2.1528761e-03 3.0687849e-03 1.2587237e-02 6.5807666e-03 1.9837137e-03 5.6454756e-03 2.0386836e-02 1.2074296e-02 9.7714086e-03 2.4352442e-02 4.0729711e-02 3.8531245e-02 5.0194503e-03 -7.7012557e-02 -1.1245328e-01 -7.0193530e-02 -2.6299389e-02 -2.9110247e-02 -1.4128121e-02 -1.1516324e-02 -1.2591325e-03 -6.2611218e-03 -8.8618335e-03

1.6472000e+01 -5.3420216e-02 -3.9346408e-02 -1.8203921e-02 -9.3149735e-03 -6.8023762e-03 6.9144624e-03 1.2179202e-02 4.3615670e-03 -2.9863199e-03 2.5477252e-03 9.6012793e-03 3.8233110e-03 -2.1391550e-03 1.8296278e-03 1.7702459e-02 9.7415216e-03 5.5017177e-03 2.1344996e-02 3.5047354e-02 3.4412167e-02 1.0831892e-03 -6.9771783e-02 -1.0003824e-01 -6.2892647e-02 -2.5353073e-02 -2.4407229e-02 -1.0342120e-02 -1.1298047e-02 6.0472440e-04 -2.1720357e-03 -6.3468583e-03

1.9209000e+01 -5.7030140e-02 -4.1265306e-02 -1.7228389e-02 -1.2688257e-02 -8.4033698e-03 5.7015469e-03 1.2907169e-02 3.4352377e-03 -5.0487762e-03 1.3056597e-03 5.6161372e-03 -2.8802736e-04 -3.5934617e-03 -8.7373928e-04 1.4939286e-02 7.5714783e-03 3.2327511e-03 1.6820733e-02 3.1272433e-02 2.9447905e-02 -3.2750847e-03 -6.6952062e-02 -8.7498843e-02 -5.7576870e-02 -2.1133213e-02 -1.9089570e-02 -6.5979357e-03 -6.8092665e-03 5.2975147e-03 -1.0204799e-03 -3.5447714e-03

2.2400000e+01 -5.8770920e-02 -3.8714704e-02 -1.9606096e-02 -9.2884266e-03 -3.8082855e-03 1.1500144e-02 1.4300414e-02 5.8718153e-03 -4.6939657e-03 1.9271569e-03 4.9056643e-03 -9.9490201e-04 -4.5759419e-03 -1.3287983e-03 1.3755241e-02 7.8012747e-03 2.8487876e-03 1.8075648e-02 2.9939439e-02 2.6038794e-02 -2.1202775e-03 -6.0509385e-02 -7.9343096e-02 -4.9387600e-02 -1.9836381e-02 -1.7382549e-02 -9.8415060e-03 -7.4666185e-03 2.9747848e-03 1.1637446e-03 4.2400314e-04

2.6122000e+01 -4.9454742e-02 -3.8817698e-02 -2.2268076e-02 -7.2979908e-03 3.1818824e-04 1.3693833e-02 1.5923721e-02 6.4677033e-03 -4.3439696e-03 3.0160209e-03 5.3963910e-03 -2.8304134e-03 -4.7858859e-03 -1.0357940e-03 1.0785485e-02 5.2456304e-03 -8.6762568e-04 1.3228502e-02 2.4511517e-02 2.1402970e-02 -2.8312038e-03 -5.7325358e-02 -7.3786466e-02 -4.2759792e-02 -2.0018889e-02 -1.9243602e-02 -9.8814029e-03 -3.7253902e-03 7.8470928e-04 4.5922063e-03 4.3974477e-03

3.0462000e+01 -4.8886826e-02 -4.2861350e-02 -2.7658619e-02 -8.4470508e-03 -2.3698381e-04 1.3710983e-02 1.4504991e-02 6.4852510e-03 -2.9026245e-03 2.4517098e-03 6.3009966e-03 -3.3522929e-03 -4.9032936e-03 -2.0487487e-03 9.5503221e-03 4.0682872e-03 8.0356899e-04 1.4676641e-02 2.2471029e-02 1.9296639e-02 -4.3574514e-05 -5.1180944e-02 -6.8695090e-02 -3.8626257e-02 -1.9443646e-02 -1.8945418e-02 -8.6110245e-03 -1.8028807e-03 -3.4030293e-03 7.1285242e-03 6.6548097e-03

3.5523000e+01 -4.5739640e-02 -4.3623991e-02 -2.5830336e-02 -8.2924584e-03 -1.0197308e-03 1.2130884e-02 1.5735830e-02 5.2966211e-03 -2.2228256e-03 2.6998067e-03 5.5008993e-03 -3.6081034e-03 -5.0810297e-03 -2.5916485e-03 7.1903285e-03 1.1605786e-03 -1.6721668e-03 1.0522543e-02 1.7805876e-02 1.4734717e-02 -5.6046685e-03 -5.1458301e-02 -6.4722489e-02 -3.8473163e-02 -1.9247879e-02 -1.8255816e-02 -5.8917794e-03 -1.1518691e-03 3.5618734e-04 5.5066994e-03 8.8130131e-03

4.1425000e+01 -5.3468909e-02 -4.3356110e-02 -2.5167555e-02 -9.7743757e-03 -1.8629575e-03 1.3147723e-02 1.5213097e-02 6.3736654e-03 -1.9650007e-03 2.5734033e-03 3.8212470e-03 -2.9967037e-03 -5.4602882e-03 -2.9826199e-03 7.4389119e-03 1.1629741e-03 -2.0604560e-04 1.0675960e-02 1.7980483e-02 1.4582397e-02 -5.7827066e-03 -5.1360859e-02 -6.3105043e-02 -3.6123199e-02 -1.5454558e-02 -1.5415640e-02 -8.0569263e-03 1.3451425e-03 3.2468306e-03 7.5627734e-03 2.9306427e-03

4.8307000e+01 -5.4841477e-02 -4.5755863e-02 -2.5173500e-02 -9.9868565e-03 -1.2933258e-03 1.5853444e-02 1.8636210e-02 5.5054685e-03 -2.0905477e-03 3.9401268e-03 3.8121899e-03 -3.9002643e-03 -5.7090802e-03 -1.7417093e-03 6.5647877e-03 2.6980730e-03 -1.8301368e-03 8.8649278e-03 1.9562421e-02 1.3672546e-02 -7.6374433e-03 -5.1040582e-02 -6.3209858e-02 -3.3315215e-02 -1.3841219e-02 -1.6221063e-02 -8.0315643e-03 3.2354997e-03 5.6613899e-03 1.0477738e-02 1.9018796e-03

5.6333000e+01 -5.6062521e-02 -4.4193554e-02 -2.3240378e-02 -9.1238094e-03 -5.4992258e-04 1.7568286e-02 1.7784894e-02 6.6500513e-03 -1.6353577e-03 5.0439538e-03 4.5056710e-03 -4.8164577e-03 -6.5379552e-03 -3.0592849e-03 4.8448146e-03 3.0066608e-03 -2.3853738e-03 8.7034695e-03 1.7087001e-02 1.2656081e-02 -8.3033803e-03 -5.1014428e-02 -6.2216392e-02 -3.0754893e-02 -1.0341172e-02 -1.6075765e-02 -7.6631634e-03 3.0158460e-03 4.5588492e-03 1.1340470e-02 3.2887024e-03

6.5692000e+01 -4.7005525e-02 -4.1026086e-02 -2.0515683e-02 -5.9558038e-03 2.7434780e-03 1.9463164e-02 2.1156996e-02 7.4061233e-03 1.9442877e-04 5.1758644e-03 7.0774471e-03 -4.3804090e-03 -6.9528951e-03 -1.9721425e-03 6.1561089e-03 1.7117564e-04 -2.9935712e-03 8.7039593e-03 1.5250910e-02 1.1132473e-02 -8.8321104e-03 -5.2041218e-02 -6.1622847e-02 -3.1150954e-02 -1.2173781e-02 -1.7797530e-02 -4.4843611e-03 -1.7170622e-03 3.0972854e-03 8.6869713e-03 1.1806988e-02

7.6606000e+01 -4.5163215e-02 -3.6590729e-02 -2.0010423e-02 -4.2646054e-03 5.8371652e-03 2.4351410e-02 2.4023729e-02 1.0646462e-02 3.0670243e-03 4.7909005e-03 7.2866446e-03 -2.3124564e-03 -8.6927174e-03 -4.3060572e-03 6.6006662e-03 -1.7796794e-03 -4.3528354e-03 8.5005125e-03 1.2079399e-02 1.1302580e-02 -9.4168081e-03 -5.4800242e-02 -6.3011152e-02 -3.1178922e-02 -1.0621888e-02 -1.7134344e-02 -4.7032077e-03 -3.2235262e-04 3.2413868e-03 8.5178828e-03 1.1695713e-02

8.9333000e+01 -4.2586569e-02 -3.7698422e-02 -2.6311673e-02 -7.9429906e-03 6.8093727e-03 2.5922912e-02 2.4243869e-02 1.1876559e-02 2.1087064e-03 4.3861050e-03 6.7879527e-03 -3.1634857e-03 -8.5378806e-03 -3.8738387e-03 7.3074278e-03 -3.9475671e-03 -4.7499180e-03 7.7060032e-03 1.0083546e-02 1.1467446e-02 -8.5085092e-03 -5.5053149e-02 -6.5872255e-02 -3.4834893e-02 -1.1942750e-02 -1.5472803e-02 -4.7906514e-03 1.2221485e-03 3.9714137e-03 8.0683353e-03 7.4641429e-03

1.0417500e+02 -5.0456533e-02 -4.1960550e-02 -3.0501570e-02 -8.6112808e-03 5.0123788e-03 2.4191885e-02 2.2632460e-02 1.1559735e-02 1.0880829e-03 4.1132808e-03 4.6621762e-03 -5.4926495e-03 -9.0892157e-03 -8.1108118e-03 5.8256682e-03 -4.0159433e-03 -5.6407556e-03 6.8261786e-03 9.6818614e-03 9.4970149e-03 -9.8622535e-03 -5.5237453e-02 -6.7501710e-02 -3.4643771e-02 -7.3476681e-03 -1.2937057e-02 -2.3382137e-03 3.3979587e-03 5.0719338e-03 7.0585580e-03 7.1097173e-03

1.2148300e+02 -4.9258801e-02 -4.4684232e-02 -2.9100701e-02 -8.3932317e-03 6.8395726e-03 2.4018189e-02 2.2720839e-02 9.8591559e-03 -1.3261544e-03 2.9973531e-03 5.8361928e-03 -7.3850532e-03 -8.7970705e-03 -6.7184811e-03 6.7248213e-03 -5.0589969e-03 -6.9627111e-03 4.6289596e-03 9.3066846e-03 7.7688532e-03 -1.1572427e-02 -5.4522664e-02 -6.8960318e-02 -3.4697802e-02 -6.9798130e-03 -1.1515340e-02 -5.5899333e-04 9.1365927e-04 9.2260911e-03 6.6401492e-03 7.5267196e-03

1.4166700e+02 -6.2873047e-02 -5.0159433e-02 -3.1971485e-02 -8.9508663e-03 4.6535090e-03 2.4098474e-02 2.4607608e-02 9.6748988e-03 -6.3492660e-04 2.2819314e-03 6.0506745e-03 -6.9387617e-03 -9.3910435e-03 -6.8721071e-03 5.4375059e-03 -4.7946866e-03 -6.3131091e-03 5.7592037e-03 1.2695938e-02 8.7884994e-03 -1.1889654e-02 -5.4507350e-02 -6.9563697e-02 -3.2536761e-02 -3.8198913e-03 -1.0944062e-02 2.4981313e-03 -8.0199366e-05 9.0924330e-03 3.2595554e-03 9.9577336e-03

1.6520400e+02 -6.7027841e-02 -5.2496741e-02 -3.3539177e-02 -9.9991441e-03 8.5294486e-03 2.7518438e-02 2.5794740e-02 9.7440112e-03 7.7131755e-04 1.8864611e-03 4.3826467e-03 -7.5421868e-03 -1.0517036e-02 -6.5518339e-03 3.1495474e-03 -6.0613714e-03 -9.1550118e-03 4.3815940e-03 1.5509468e-02 9.0466863e-03 -1.2284959e-02 -5.4847957e-02 -6.6929084e-02 -2.9690401e-02 -4.0285890e-03 -1.0319286e-02 -7.0753650e-05 1.6057906e-03 1.1030835e-02 7.4005764e-03 9.1319149e-03

1.9265100e+02 -8.0089901e-02 -6.0476368e-02 -4.1373667e-02 -1.2544742e-02 7.7628793e-03 2.8523414e-02 2.5399156e-02 1.1249810e-02 9.4781689e-04 7.8235494e-04 8.2933775e-04 -8.6642553e-03 -1.1831714e-02 -8.5469334e-03 6.0158636e-04 -6.5815972e-03 -9.1258695e-03 4.3864393e-03 1.5560719e-02 9.4194128e-03 -1.1476321e-02 -5.7070940e-02 -6.5566854e-02 -2.8311761e-02 -2.8476462e-03 -9.5481169e-03 1.3559441e-04 2.6669640e-03 1.3129842e-02 5.8384505e-03 9.9147174e-03

2.2465800e+02 -7.6744394e-02 -6.3683962e-02 -3.7774690e-02 -1.2074177e-02 1.2909727e-02 3.1382254e-02 2.9359041e-02 1.2430804e-02 4.1599952e-03 1.1534704e-03 2.5524869e-03 -7.5839656e-03 -1.2329500e-02 -7.8733357e-03 5.0103755e-04 -5.7688138e-03 -9.7495993e-03 5.1236931e-03 1.4819462e-02 8.5984919e-03 -1.1550383e-02 -5.9995947e-02 -6.7694601e-02 -2.4107368e-02 -1.7975591e-03 -7.8031088e-03 -1.6908088e-05 4.1448595e-03 1.5623718e-02 1.2116229e-02 4.7326607e-03

2.6198300e+02 -8.4328163e-02 -6.6759708e-02 -4.3504336e-02 -1.5780953e-02 1.2964948e-02 3.5365284e-02 3.2742385e-02 1.3661986e-02 3.2222272e-03 2.4843186e-03 4.2178705e-03 -6.7137747e-03 -1.3091211e-02 -7.5976388e-03 1.0868030e-03 -4.2156345e-03 -8.3409770e-03 6.1164710e-03 1.2515125e-02 9.0677844e-03 -1.2385367e-02 -6.1499096e-02 -6.8497125e-02 -2.3597461e-02 -1.2860677e-03 -9.5101981e-03 1.7139365e-03 7.1311663e-03 1.4719028e-02 1.4816097e-02 2.3143201e-03

3.0551000e+02 -8.4497914e-02 -6.8797360e-02 -4.1800379e-02 -1.7376795e-02 1.1914317e-02 3.5443621e-02 3.3720859e-02 1.3717510e-02 1.6984543e-03 2.8719758e-03 4.4447560e-03 -7.4767932e-03 -1.5573531e-02 -9.9685919e-03 -2.9338318e-04 -6.1502572e-03 -1.1170178e-02 3.5202771e-03 1.0868249e-02 7.1645949e-03 -1.6515445e-02 -6.3196250e-02 -6.6347488e-02 -2.3619780e-02 -3.1503902e-04 -8.3608006e-03 2.4661065e-03 8.6205604e-03 1.0495478e-02 1.6907148e-02 1.3032277e-03

3.5626700e+02 -7.8360464e-02 -6.4529789e-02 -3.7450156e-02 -1.1939421e-02 1.5813636e-02 4.3790648e-02 3.8910991e-02 1.7489776e-02 2.7031895e-03 5.5261496e-03 6.6503084e-03 -5.4572843e-03 -1.4679183e-02 -9.4878819e-03 4.2751707e-04 -7.7916977e-03 -1.1773183e-02 1.8561267e-03 1.2431346e-02 7.5796437e-03 -1.8196765e-02 -6.1463282e-02 -6.1365264e-02 -2.1335021e-02 6.7418122e-04 -6.5242159e-03 2.9782726e-03 1.1477505e-02 1.3401045e-02 1.4996495e-02 6.2658285e-03

4.1545800e+02 -7.7087870e-02 -6.7686657e-02 -3.3481265e-02 -9.0441981e-03 1.9187120e-02 4.5815674e-02 4.0711193e-02 1.7448474e-02 3.1117223e-03 3.2202068e-03 4.0379310e-03 -9.0577048e-03 -1.6310238e-02 -1.1025147e-02 1.2781577e-04 -1.1119378e-02 -1.3684704e-02 5.9691747e-04 1.0373951e-02 4.7465778e-03 -2.2067639e-02 -6.3064420e-02 -5.9730443e-02 -2.0099005e-02 3.7909745e-03 -3.9673837e-03 4.2107505e-03 8.7785807e-03 1.2971683e-02 8.5640033e-03 1.0353194e-02

4.8448300e+02 -8.3266202e-02 -6.9333902e-02 -3.4840265e-02 -6.8800250e-03 2.4158496e-02 4.7257049e-02 4.1119457e-02 1.4558949e-02 3.4454841e-03 2.5750901e-03 3.2218573e-03 -1.0935395e-02 -1.7987456e-02 -1.0934387e-02 -3.4086144e-04 -1.0725498e-02 -1.3321196e-02 1.9431982e-03 9.4758882e-03 2.2974591e-03 -2.1787790e-02 -6.1806180e-02 -5.6766654e-02 -1.3786112e-02 6.5959209e-03 -5.0540373e-03 4.1565699e-03 6.5843382e-03 1.7240568e-02 5.4905955e-03 1.2506461e-02

5.6497600e+02 -9.9792674e-02 -7.5493103e-02 -3.9732136e-02 -7.3902881e-03 2.7530827e-02 4.4892667e-02 3.7718756e-02 1.1605016e-02 2.3345984e-03 3.0497169e-03 5.7114313e-04 -1.4789627e-02 -2.0607707e-02 -1.1460493e-02 1.7300076e-03 -7.3361623e-03 -1.1771744e-02 4.6725685e-03 7.6006294e-03 4.9417037e-04 -1.9428366e-02 -5.9279661e-02 -4.7474520e-02 -7.0932105e-03 1.2149375e-02 -5.4846181e-03 5.5234913e-03 4.5909219e-03 1.7663093e-02 6.2149601e-03 1.3640676e-02

Fig. 2d

6.0000000e-01 6.6580497e-02 4.8831993e-02 9.8370302e-02 1.4267097e-01 9.3682251e-02 7.7849736e-02 6.4220224e-02 3.1449033e-02 4.9544170e-02 2.1963831e-02 -1.5592893e-01 -3.6410471e-01 -3.7112093e-01 -1.8065246e-01 -3.1318526e-03 1.6740337e-01 1.7410104e-01 -2.1840385e-01 -1.2208289e+00 -1.7219938e+00 -1.3692406e+00 -9.3548263e-01 -6.0416743e-01 -3.6104942e-01 -2.0407166e-01 -6.7407533e-02 1.5685081e-02 1.0553716e-01 1.8111134e-01 2.0473563e-01 2.3537252e-01 2.5240420e-01

8.0000000e-01 5.5450280e-02 2.7880947e-02 7.1914606e-02 9.9546536e-02 6.5351652e-02 6.6244168e-02 5.8609939e-02 4.1388786e-02 6.4931173e-02 3.8355202e-02 -1.2858672e-01 -3.4416786e-01 -3.5216587e-01 -1.5977041e-01 1.6615184e-02 1.8074254e-01 1.7469459e-01 -2.3094406e-01 -1.2535185e+00 -1.7670909e+00 -1.3941542e+00 -9.4229746e-01 -6.0360469e-01 -3.6351444e-01 -2.1643688e-01 -9.3823553e-02 -8.8450148e-03 7.4110508e-02 1.4201244e-01 1.8418153e-01 1.9404450e-01 2.6153976e-01

1.0000000e+00 1.6598778e-02 3.1963457e-03 4.2981423e-02 6.0921821e-02 4.8398940e-02 5.6026778e-02 5.8098263e-02 5.5308926e-02 8.3710235e-02 6.4024930e-02 -9.7613638e-02 -3.1890817e-01 -3.2609644e-01 -1.3226657e-01 4.6538241e-02 2.0355242e-01 1.8745569e-01 -2.2979395e-01 -1.2694672e+00 -1.7823428e+00 -1.3926212e+00 -9.2516650e-01 -5.8497262e-01 -3.5761208e-01 -2.2216581e-01 -1.0902876e-01 -3.2354233e-02 4.1846083e-02 1.1175549e-01 1.5633457e-01 1.5495626e-01 2.2530475e-01

1.2000000e+00 -5.8185374e-03 -1.9109237e-02 1.7814171e-02 4.0345901e-02 4.2673068e-02 5.5708357e-02 6.5026360e-02 6.7797584e-02 9.7568116e-02 7.5562297e-02 -8.4873276e-02 -3.0769175e-01 -3.1381867e-01 -1.1728532e-01 6.1593288e-02 2.1578440e-01 1.9109121e-01 -2.3082013e-01 -1.2756599e+00 -1.7751774e+00 -1.3653999e+00 -8.9217966e-01 -5.5044233e-01 -3.3975110e-01 -2.2017588e-01 -1.2367456e-01 -4.5419796e-02 2.5199421e-02 9.2466379e-02 1.3890600e-01 1.3550784e-01 1.9366141e-01

1.4000000e+00 -3.1919663e-02 -3.2856913e-02 -1.1194916e-03 3.2210222e-02 4.1373734e-02 6.0936107e-02 7.2615634e-02 8.2128840e-02 1.1499915e-01 9.6414911e-02 -6.3928970e-02 -2.8556433e-01 -2.8619041e-01 -8.6877877e-02 9.2753198e-02 2.4227201e-01 2.1576168e-01 -2.0927441e-01 -1.2495242e+00 -1.7256126e+00 -1.3047185e+00 -8.2817396e-01 -4.9510910e-01 -2.9880424e-01 -1.9520739e-01 -1.0649827e-01 -4.4647655e-02 3.0853638e-02 1.1325675e-01 1.4612812e-01 1.3560479e-01 1.5095646e-01

1.6000000e+00 -3.4993891e-02 -4.2796346e-02 -7.5020576e-03 3.2523401e-02 3.5408001e-02 6.1286603e-02 7.6572290e-02 8.6892040e-02 1.2025940e-01 9.9432816e-02 -6.0229018e-02 -2.8159483e-01 -2.7584044e-01 -7.5031749e-02 1.0427428e-01 2.4894893e-01 2.1720940e-01 -2.1645948e-01 -1.2403407e+00 -1.6895564e+00 -1.2542659e+00 -7.8445404e-01 -4.6274782e-01 -2.7885708e-01 -1.8721675e-01 -1.0627349e-01 -5.0986344e-02 3.2976939e-02 1.1025490e-01 1.4744135e-01 1.2935542e-01 1.5200118e-01

1.8500000e+00 -8.1560040e-02 -4.5601177e-02 -2.0603791e-02 2.9463978e-02 3.5812881e-02 6.4983562e-02 8.1614563e-02 9.3590661e-02 1.2722188e-01 1.1279113e-01 -4.8742386e-02 -2.6605592e-01 -2.5183147e-01 -4.9299740e-02 1.2789146e-01 2.6773125e-01 2.3181005e-01 -2.0382417e-01 -1.2159628e+00 -1.6385219e+00 -1.1994694e+00 -7.2812621e-01 -4.2024955e-01 -2.5317480e-01 -1.7486433e-01 -9.8505249e-02 -5.0732377e-02 3.9643098e-02 1.1882227e-01 1.3732421e-01 1.0388359e-01 1.2102439e-01

2.1000000e+00 -9.9733505e-02 -3.7754095e-02 -1.9015782e-02 3.0909136e-02 3.3150936e-02 5.9669801e-02 7.9653831e-02 9.3107710e-02 1.2928904e-01 1.1515069e-01 -4.6093894e-02 -2.5967698e-01 -2.3812526e-01 -3.7606141e-02 1.3847724e-01 2.7257569e-01 2.3248361e-01 -2.0409696e-01 -1.1967541e+00 -1.5942324e+00 -1.1456782e+00 -6.8396657e-01 -3.9532238e-01 -2.4977125e-01 -1.7587747e-01 -1.0727540e-01 -5.0492401e-02 2.9964090e-02 1.1118462e-01 1.3016371e-01 1.0391516e-01 1.4355275e-01

2.3500000e+00 -9.9510069e-02 -3.5703677e-02 -2.2662457e-02 2.3301974e-02 2.8263589e-02 5.9756297e-02 7.4150274e-02 9.0844632e-02 1.3058043e-01 1.1647332e-01 -4.5634082e-02 -2.5208055e-01 -2.2559459e-01 -2.6334742e-02 1.4560874e-01 2.7467555e-01 2.3022769e-01 -1.9148558e-01 -1.1658443e+00 -1.5401114e+00 -1.0965906e+00 -6.4328738e-01 -3.7315844e-01 -2.3958266e-01 -1.7190268e-01 -1.1363249e-01 -4.8936462e-02 2.7085688e-02 1.1137188e-01 1.1170133e-01 1.0595858e-01 1.4699281e-01

2.6000000e+00 -9.8205405e-02 -3.5861986e-02 -1.5683479e-02 2.1762297e-02 2.0663125e-02 5.9553394e-02 7.1905099e-02 9.0565848e-02 1.3009052e-01 1.1195496e-01 -4.5938383e-02 -2.4534478e-01 -2.1746253e-01 -1.8788329e-02 1.4965610e-01 2.7052533e-01 2.2260654e-01 -1.9228511e-01 -1.1451025e+00 -1.4938642e+00 -1.0469822e+00 -6.1578737e-01 -3.5915017e-01 -2.3721127e-01 -1.7429340e-01 -1.2160631e-01 -4.9163148e-02 2.9036291e-02 1.0348083e-01 1.1464461e-01 1.1182407e-01 1.5131598e-01

2.8500000e+00 -9.4932678e-02 -5.6104076e-02 -2.4921299e-02 1.7876874e-02 2.3681280e-02 6.1551367e-02 7.9135536e-02 9.1272140e-02 1.3294749e-01 1.1674151e-01 -4.3357580e-02 -2.3831559e-01 -2.0511704e-01 -8.7541691e-03 1.5442444e-01 2.6817615e-01 2.1741763e-01 -1.8881247e-01 -1.1233679e+00 -1.4484584e+00 -1.0070630e+00 -5.8894930e-01 -3.4194374e-01 -2.2698652e-01 -1.7715286e-01 -1.2091399e-01 -5.0877508e-02 2.9240306e-02 9.4037149e-02 1.0964870e-01 1.1582895e-01 1.4263820e-01

3.1000000e+00 -1.1293418e-01 -6.6980022e-02 -3.5019472e-02 1.7234630e-02 2.8756053e-02 6.1920184e-02 8.3555363e-02 9.7492886e-02 1.3746567e-01 1.1876425e-01 -4.0926854e-02 -2.2567753e-01 -1.9089267e-01 5.8704496e-03 1.6002685e-01 2.6867585e-01 2.1672676e-01 -1.8894183e-01 -1.1004523e+00 -1.4042539e+00 -9.6401938e-01 -5.6468154e-01 -3.2610797e-01 -2.2345787e-01 -1.7750350e-01 -1.1748847e-01 -5.6490057e-02 3.0296412e-02 9.1133944e-02 1.1465985e-01 1.0563943e-01 1.2661430e-01

3.3500000e+00 -1.2138449e-01 -8.9705226e-02 -4.2434492e-02 9.6480658e-03 3.1251424e-02 5.8215511e-02 8.5886213e-02 1.0039504e-01 1.3976381e-01 1.2580260e-01 -3.5069502e-02 -2.1263548e-01 -1.7094800e-01 2.0341904e-02 1.6802383e-01 2.6956668e-01 2.1821833e-01 -1.7615638e-01 -1.0592163e+00 -1.3439492e+00 -9.1868184e-01 -5.3163564e-01 -3.0800727e-01 -2.1703368e-01 -1.7126819e-01 -1.1210847e-01 -6.0443206e-02 2.6759560e-02 9.0500767e-02 9.9214007e-02 9.1228185e-02 1.0950919e-01

3.7500000e+00 -1.3598685e-01 -9.8877550e-02 -4.3716458e-02 2.8043449e-03 2.9130732e-02 5.7159867e-02 8.2563544e-02 1.0446652e-01 1.3892850e-01 1.2811751e-01 -2.9104801e-02 -1.9633982e-01 -1.5592063e-01 3.5399002e-02 1.7215593e-01 2.7023665e-01 2.1868197e-01 -1.6613132e-01 -1.0099158e+00 -1.2776977e+00 -8.7247574e-01 -5.0735714e-01 -2.9520991e-01 -2.1368609e-01 -1.6659903e-01 -1.1034013e-01 -6.3556456e-02 2.7750233e-02 8.5625831e-02 9.1148883e-02 6.5794584e-02 1.1284812e-01

4.1500000e+00 -1.2530353e-01 -9.5249665e-02 -3.4663977e-02 2.8889714e-03 2.0666587e-02 5.3167918e-02 7.7604989e-02 9.7218466e-02 1.3171266e-01 1.2339586e-01 -2.7368321e-02 -1.8834131e-01 -1.4548021e-01 3.8746856e-02 1.6991509e-01 2.5910800e-01 2.0964575e-01 -1.6076351e-01 -9.6446599e-01 -1.2191232e+00 -8.3647248e-01 -4.8846729e-01 -2.8802792e-01 -2.1129232e-01 -1.7290776e-01 -1.1358853e-01 -6.4842262e-02 1.9527390e-02 8.2346439e-02 8.7430888e-02 7.5631727e-02 1.2996294e-01

4.5500000e+00 -1.0273121e-01 -8.8660042e-02 -3.8421640e-02 4.2469687e-03 1.7157406e-02 4.6455286e-02 7.0403213e-02 9.0303781e-02 1.2502058e-01 1.2023251e-01 -2.7227519e-02 -1.7918594e-01 -1.3690788e-01 3.9479890e-02 1.6253657e-01 2.4889036e-01 2.0089779e-01 -1.5844996e-01 -9.2520236e-01 -1.1660026e+00 -8.0591444e-01 -4.7833211e-01 -2.8652543e-01 -2.1268091e-01 -1.7943840e-01 -1.1537442e-01 -6.8620485e-02 1.9634532e-02 8.0189105e-02 8.8843030e-02 7.8090435e-02 1.4156078e-01

4.9500000e+00 -1.1337503e-01 -8.3784968e-02 -3.9591830e-02 9.8153814e-03 2.0281308e-02 4.4305236e-02 7.0392428e-02 9.0834464e-02 1.2721290e-01 1.2175728e-01 -1.9098882e-02 -1.5929607e-01 -1.1679581e-01 4.9923711e-02 1.7112462e-01 2.5128269e-01 2.0407402e-01 -1.3733431e-01 -8.7767924e-01 -1.1041297e+00 -7.6322398e-01 -4.4995204e-01 -2.6796488e-01 -1.9802314e-01 -1.6208181e-01 -9.9198581e-02 -5.0905462e-02 3.7119518e-02 9.9830659e-02 1.0054358e-01 1.0840423e-01 1.3973589e-01

5.3500000e+00 -1.1718381e-01 -8.2252327e-02 -4.0498651e-02 1.2261630e-02 3.1875897e-02 4.7029839e-02 6.7835788e-02 8.9084722e-02 1.2226726e-01 1.2101301e-01 -2.0188122e-02 -1.5013057e-01 -1.0967079e-01 4.8101747e-02 1.6053855e-01 2.4003610e-01 1.9399723e-01 -1.3362628e-01 -8.4860931e-01 -1.0596688e+00 -7.4146587e-01 -4.4081424e-01 -2.7192013e-01 -2.0321716e-01 -1.6397963e-01 -9.9380502e-02 -5.2890426e-02 4.1006605e-02 9.6326922e-02 9.9076849e-02 1.1813703e-01 1.5069777e-01

5.7500000e+00 -1.3048098e-01 -8.7954615e-02 -3.7668302e-02 1.1453911e-02 2.9480959e-02 5.0024092e-02 6.9049536e-02 9.0841835e-02 1.2395851e-01 1.1817108e-01 -1.5520650e-02 -1.3795553e-01 -1.0163643e-01 5.2503089e-02 1.5489212e-01 2.3387109e-01 1.8994138e-01 -1.2726959e-01 -8.1698547e-01 -1.0249899e+00 -7.2116345e-01 -4.3598853e-01 -2.7015177e-01 -2.0204051e-01 -1.6317211e-01 -1.0444660e-01 -6.0169672e-02 3.7405953e-02 9.4089891e-02 9.7056414e-02 1.1723112e-01 1.5000559e-01

6.1500000e+00 -1.1422538e-01 -9.5565304e-02 -3.2948152e-02 -2.8742647e-04 2.6461056e-02 4.8593495e-02 6.8083439e-02 8.7435784e-02 1.2509344e-01 1.1911701e-01 -1.4271918e-02 -1.2616838e-01 -9.6924744e-02 5.3854295e-02 1.4684906e-01 2.2457855e-01 1.8634878e-01 -1.2018291e-01 -7.8085515e-01 -9.8710257e-01 -7.0072545e-01 -4.3072541e-01 -2.7064904e-01 -1.9811162e-01 -1.6278105e-01 -1.0428858e-01 -6.6781123e-02 4.0284096e-02 8.6279516e-02 9.4431252e-02 1.1432809e-01 1.6678883e-01

6.5500000e+00 -1.1773417e-01 -1.0774337e-01 -3.8877363e-02 -2.1684868e-03 1.5610287e-02 4.4647355e-02 6.8383147e-02 8.6550456e-02 1.2832277e-01 1.1608861e-01 -5.8511564e-03 -1.0955595e-01 -8.4787178e-02 5.6424443e-02 1.4636289e-01 2.2358182e-01 1.9044426e-01 -1.0229909e-01 -7.2881350e-01 -9.3540391e-01 -6.6987021e-01 -4.1874862e-01 -2.6337036e-01 -1.9361761e-01 -1.5792487e-01 -1.0734198e-01 -6.4568744e-02 3.7791376e-02 8.2185928e-02 8.9244809e-02 9.5099258e-02 1.4336786e-01

7.6380000e+00 -1.2955809e-01 -1.1789062e-01 -5.3235465e-02 -4.6631467e-03 1.5222883e-02 3.7513900e-02 6.7566905e-02 8.5095291e-02 1.2888117e-01 1.1880037e-01 3.5717714e-03 -8.9713696e-02 -6.9736393e-02 5.7026697e-02 1.4071997e-01 2.1580418e-01 1.8728282e-01 -8.2688952e-02 -6.6727492e-01 -8.6790850e-01 -6.3548908e-01 -4.0298802e-01 -2.5933097e-01 -1.9198557e-01 -1.5808408e-01 -1.0855974e-01 -6.6052091e-02 2.0173672e-02 6.4285390e-02 7.0251257e-02 7.9531156e-02 1.2328933e-01

8.9070000e+00 -1.5575931e-01 -1.1284778e-01 -6.5017197e-02 -7.8302568e-03 8.7703848e-03 3.5633800e-02 6.2865448e-02 8.6763175e-02 1.2326854e-01 1.1546649e-01 1.4558352e-02 -6.9172850e-02 -5.1177037e-02 5.8185574e-02 1.3462514e-01 2.0691018e-01 1.8205660e-01 -6.1316351e-02 -5.9808970e-01 -7.8958946e-01 -5.9272059e-01 -3.8385239e-01 -2.4729743e-01 -1.8493060e-01 -1.5438877e-01 -1.0679844e-01 -6.5047759e-02 1.4364154e-02 5.1876861e-02 6.7425953e-02 6.0916705e-02 9.2438413e-02

1.0387000e+01 -1.6414285e-01 -1.1732214e-01 -6.7340850e-02 -1.7568192e-02 5.8070292e-03 2.7682081e-02 5.7606970e-02 8.4994769e-02 1.1973395e-01 1.1987371e-01 2.8598980e-02 -4.7069259e-02 -3.7057637e-02 6.1247605e-02 1.2949461e-01 1.9660523e-01 1.7875603e-01 -3.7964370e-02 -5.2754531e-01 -7.1459910e-01 -5.5268933e-01 -3.6594856e-01 -2.3536465e-01 -1.7736240e-01 -1.4708395e-01 -1.0465437e-01 -7.0617050e-02 8.1636519e-03 5.2618410e-02 5.8615261e-02 5.4074598e-02 9.3553845e-02

1.2113000e+01 -1.6956310e-01 -1.0954066e-01 -5.9158952e-02 -1.7435826e-02 2.8919479e-03 3.0657018e-02 5.1884781e-02 8.0152708e-02 1.0986701e-01 1.1296431e-01 2.9535294e-02 -3.5985297e-02 -3.3501647e-02 5.2250783e-02 1.1788192e-01 1.8053242e-01 1.6629698e-01 -2.5977639e-02 -4.6816864e-01 -6.5259750e-01 -5.2394999e-01 -3.5584750e-01 -2.3099140e-01 -1.7526398e-01 -1.4463752e-01 -1.0277827e-01 -6.7637071e-02 6.3868968e-03 4.9009172e-02 5.2491324e-02 4.5666616e-02 1.0094235e-01

1.4125000e+01 -1.7396159e-01 -1.2679551e-01 -6.1333943e-02 -1.7430090e-02 4.3705987e-03 3.1558091e-02 5.1752435e-02 7.8544093e-02 1.0899493e-01 1.1281322e-01 3.8060304e-02 -2.2006416e-02 -2.6174803e-02 4.7796670e-02 1.1268892e-01 1.7259226e-01 1.6196405e-01 -1.0629295e-02 -4.1256899e-01 -5.9436852e-01 -4.9148233e-01 -3.4047748e-01 -2.2425835e-01 -1.7123958e-01 -1.3802411e-01 -9.8702532e-02 -6.9077041e-02 -1.5581140e-04 5.0533686e-02 3.3095680e-02 5.1107429e-02 1.0739956e-01

1.6472000e+01 -1.9911902e-01 -1.3595022e-01 -7.6911992e-02 -1.8888832e-02 6.0041664e-03 4.1105998e-02 5.7443071e-02 8.1067535e-02 1.1399228e-01 1.2357227e-01 5.1416200e-02 2.3758271e-03 -6.8776933e-03 5.9058854e-02 1.1854041e-01 1.7753229e-01 1.6748655e-01 1.3573291e-02 -3.5482502e-01 -5.3598685e-01 -4.4688364e-01 -3.1166510e-01 -2.0241957e-01 -1.4574870e-01 -1.2306375e-01 -7.7142955e-02 -5.4391833e-02 9.1784531e-03 5.1567446e-02 3.5019385e-02 4.2769542e-02 1.1014822e-01

1.9209000e+01 -2.0985652e-01 -1.5464426e-01 -9.3783984e-02 -3.0342607e-02 -1.0159587e-03 3.1545564e-02 5.6746992e-02 7.7550446e-02 1.1574513e-01 1.2932200e-01 6.5633089e-02 2.2648836e-02 1.1271757e-02 7.0730768e-02 1.2518685e-01 1.8090872e-01 1.7681449e-01 3.8794986e-02 -3.0671990e-01 -4.8792555e-01 -4.1061296e-01 -2.9000977e-01 -1.8670253e-01 -1.3434609e-01 -1.1028129e-01 -6.4930867e-02 -5.6944896e-02 2.5992757e-03 4.2270002e-02 3.3203340e-02 3.0008384e-02 8.0475930e-02

2.2400000e+01 -2.0893698e-01 -1.5152616e-01 -9.3851442e-02 -3.2969364e-02 -9.7280317e-03 2.6044892e-02 5.3218846e-02 6.6785703e-02 1.0750043e-01 1.2483974e-01 6.6881453e-02 2.7853120e-02 1.2357711e-02 6.8253607e-02 1.1740126e-01 1.6998886e-01 1.6670233e-01 4.2115333e-02 -2.7620342e-01 -4.5703083e-01 -3.9492870e-01 -2.8464283e-01 -1.8800727e-01 -1.3368035e-01 -1.1733437e-01 -6.4910899e-02 -6.3539128e-02 -2.3922107e-03 3.5344079e-02 4.2971674e-02 1.9225880e-02 7.4749710e-02

2.6122000e+01 -2.1491258e-01 -1.4677300e-01 -8.8803225e-02 -3.1514363e-02 -3.9484029e-03 2.5706781e-02 5.2837758e-02 6.7927870e-02 1.0563389e-01 1.2117236e-01 7.1839898e-02 3.2234901e-02 1.8218502e-02 6.5873920e-02 1.1409212e-01 1.6256747e-01 1.6303741e-01 5.2953464e-02 -2.4515379e-01 -4.2637472e-01 -3.8341427e-01 -2.7821235e-01 -1.8099080e-01 -1.3719774e-01 -1.1243525e-01 -6.1123677e-02 -6.4048320e-02 -4.0794372e-03 2.6325429e-02 5.5351448e-02 2.9445161e-02 7.7345488e-02

3.0462000e+01 -2.1721380e-01 -1.4132908e-01 -8.1735061e-02 -2.5896328e-02 7.5586454e-03 3.0958164e-02 5.5588634e-02 7.3887229e-02 1.0895755e-01 1.2337962e-01 7.7828555e-02 4.2375550e-02 2.5761547e-02 7.0100235e-02 1.1693104e-01 1.6427079e-01 1.6508340e-01 6.2852485e-02 -2.1822725e-01 -3.9550472e-01 -3.6098044e-01 -2.6410921e-01 -1.7200110e-01 -1.2358814e-01 -1.0267963e-01 -5.2526170e-02 -5.1702300e-02 9.6601448e-03 2.8011040e-02 5.6926303e-02 4.3441557e-02 1.1677580e-01

3.5523000e+01 -2.1913116e-01 -1.5701229e-01 -9.3837329e-02 -3.1053525e-02 5.2780130e-03 3.1655957e-02 5.7085428e-02 7.9871320e-02 1.1322864e-01 1.2987880e-01 8.5508358e-02 5.5902250e-02 3.8950522e-02 7.7584302e-02 1.2206946e-01 1.7153663e-01 1.7609215e-01 7.8860475e-02 -1.9724491e-01 -3.7023775e-01 -3.4504110e-01 -2.5351925e-01 -1.6176446e-01 -1.1540690e-01 -9.2377726e-02 -5.1024672e-02 -4.6937772e-02 1.1641326e-02 1.9337689e-02 4.7121762e-02 4.1690033e-02 1.0967337e-01

4.1425000e+01 -1.9732881e-01 -1.4581597e-01 -9.3367455e-02 -2.4314613e-02 2.9503481e-03 2.6916004e-02 6.0051383e-02 8.1105957e-02 1.1435097e-01 1.3283945e-01 9.2592492e-02 6.5246986e-02 4.7144895e-02 8.4365938e-02 1.2844325e-01 1.8011439e-01 1.8806665e-01 9.1371055e-02 -1.7373394e-01 -3.4368084e-01 -3.2273974e-01 -2.3711932e-01 -1.5070755e-01 -9.6877021e-02 -7.7557576e-02 -4.5445014e-02 -3.6111081e-02 2.3026192e-02 3.5702827e-02 5.0664224e-02 5.6019494e-02 1.1855680e-01

4.8307000e+01 -1.9116243e-01 -1.4802394e-01 -9.6056508e-02 -2.8702547e-02 -2.4896768e-03 2.5717607e-02 6.1528594e-02 8.0623025e-02 1.1458741e-01 1.3606138e-01 9.7278310e-02 7.0274356e-02 5.0658093e-02 8.5508187e-02 1.2826939e-01 1.7875253e-01 1.8646085e-01 9.2509349e-02 -1.6089126e-01 -3.3450917e-01 -3.2025253e-01 -2.3317240e-01 -1.4496680e-01 -9.3952344e-02 -7.5672918e-02 -4.3859695e-02 -3.6443458e-02 2.5578395e-02 3.9257850e-02 4.7614606e-02 6.7421409e-02 9.3077718e-02

5.6333000e+01 -1.8748413e-01 -1.4186324e-01 -9.6771175e-02 -3.1750916e-02 -2.1452988e-03 2.2442653e-02 5.9527386e-02 7.7979147e-02 1.1313664e-01 1.3485302e-01 1.0021591e-01 7.2016341e-02 4.8812155e-02 8.6397609e-02 1.2817506e-01 1.7548730e-01 1.8588788e-01 9.2131085e-02 -1.5342303e-01 -3.3349767e-01 -3.1771362e-01 -2.3526528e-01 -1.4480328e-01 -9.2908547e-02 -7.6345584e-02 -4.2021172e-02 -3.5954031e-02 2.2088512e-02 3.9918190e-02 4.2492787e-02 6.0399811e-02 1.0232649e-01

6.5692000e+01 -1.8247562e-01 -1.5689988e-01 -1.0505390e-01 -4.0214448e-02 -8.4361882e-03 2.2037705e-02 5.8963183e-02 8.0344324e-02 1.1746599e-01 1.3932350e-01 1.0463762e-01 7.8990861e-02 5.4339537e-02 9.4213041e-02 1.3316587e-01 1.7920526e-01 1.8818722e-01 9.7737838e-02 -1.4975873e-01 -3.3175556e-01 -3.1712169e-01 -2.3209705e-01 -1.3879286e-01 -9.2324602e-02 -7.4279016e-02 -3.3701792e-02 -2.5026892e-02 2.0518112e-02 3.4180370e-02 3.1633418e-02 4.8267511e-02 9.0698283e-02

7.6606000e+01 -1.7168292e-01 -1.4862759e-01 -9.5647262e-02 -3.8186714e-02 -5.0222593e-03 2.3196595e-02 5.6133140e-02 7.8784460e-02 1.1571543e-01 1.3578302e-01 1.0348135e-01 7.6659277e-02 4.9743772e-02 9.1966572e-02 1.2919851e-01 1.7570316e-01 1.8709187e-01 9.8621401e-02 -1.4986842e-01 -3.3307001e-01 -3.1940939e-01 -2.3836816e-01 -1.4504720e-01 -9.6550990e-02 -8.0491598e-02 -3.1010046e-02 -2.4774959e-02 1.7597427e-02 3.9998266e-02 3.9145430e-02 5.1271176e-02 1.1377714e-01

8.9333000e+01 -1.7615065e-01 -1.5031790e-01 -9.4736664e-02 -3.6548325e-02 -6.9477964e-03 2.0451796e-02 5.4199112e-02 7.7928255e-02 1.1576611e-01 1.3809432e-01 1.0494597e-01 7.6625252e-02 4.8431758e-02 9.1167518e-02 1.3094631e-01 1.7821599e-01 1.8914082e-01 1.0349650e-01 -1.4255358e-01 -3.3208692e-01 -3.2199564e-01 -2.3603079e-01 -1.4643202e-01 -1.0066859e-01 -7.9240726e-02 -3.3029562e-02 -1.8553201e-02 2.2128312e-02 4.6608645e-02 4.1705182e-02 4.5818018e-02 9.4561294e-02

1.0417500e+02 -2.0181609e-01 -1.5830370e-01 -9.9159769e-02 -3.7146428e-02 -1.9376912e-03 2.5181891e-02 5.6069555e-02 8.0414322e-02 1.1738099e-01 1.4251713e-01 1.1037041e-01 8.1667946e-02 5.3491930e-02 9.4944985e-02 1.3507015e-01 1.8258590e-01 1.9439209e-01 1.0787334e-01 -1.3916499e-01 -3.3507380e-01 -3.1869994e-01 -2.3724884e-01 -1.5015154e-01 -1.0058737e-01 -8.2000896e-02 -3.3907664e-02 -3.0423149e-02 2.6929557e-02 5.1039746e-02 4.4132758e-02 5.0250649e-02 8.4114517e-02

1.2148300e+02 -2.0934677e-01 -1.6193440e-01 -1.1285723e-01 -4.7707323e-02 -1.3985925e-02 2.0831173e-02 5.5982316e-02 8.0781638e-02 1.2176972e-01 1.4901294e-01 1.1713877e-01 8.9473112e-02 6.4691991e-02 1.0403185e-01 1.4650155e-01 1.9442172e-01 2.0428075e-01 1.1977841e-01 -1.3006176e-01 -3.2952780e-01 -3.1688537e-01 -2.3105937e-01 -1.4248632e-01 -9.6908327e-02 -7.5839775e-02 -3.6170653e-02 -2.5745892e-02 2.8497300e-02 4.8756768e-02 4.3606076e-02 2.8741506e-02 6.1702054e-02

1.4166700e+02 -2.1460306e-01 -1.6297063e-01 -1.1769821e-01 -4.9621014e-02 -1.0824766e-02 2.1913279e-02 5.7996002e-02 8.3416077e-02 1.2162497e-01 1.5270026e-01 1.2147331e-01 9.2918917e-02 6.9025331e-02 1.0742789e-01 1.4824069e-01 1.9804927e-01 2.0750657e-01 1.1983796e-01 -1.3683811e-01 -3.3476978e-01 -3.1620911e-01 -2.3145543e-01 -1.4438966e-01 -9.6677648e-02 -7.8025857e-02 -3.6324677e-02 -2.8740776e-02 1.3285904e-02 4.3441478e-02 4.5642113e-02 3.5106362e-02 7.9442161e-02

1.6520400e+02 -2.1757018e-01 -1.5774919e-01 -1.1361827e-01 -5.4335296e-02 -1.6048025e-02 1.5745279e-02 5.8247147e-02 8.1665906e-02 1.2231134e-01 1.5328535e-01 1.2036529e-01 9.6690280e-02 6.8437548e-02 1.0801076e-01 1.5193600e-01 2.0265912e-01 2.1271207e-01 1.2195719e-01 -1.4154640e-01 -3.3544552e-01 -3.1776424e-01 -2.2865890e-01 -1.3879976e-01 -8.8866343e-02 -6.9747122e-02 -3.3757162e-02 -2.0878470e-02 8.6506712e-03 4.2419368e-02 4.0708781e-02 2.2322456e-02 7.5334990e-02

1.9265100e+02 -2.2864787e-01 -1.6909940e-01 -1.1583139e-01 -4.7281896e-02 -9.0141781e-03 1.7171962e-02 6.0412175e-02 8.0926556e-02 1.1848631e-01 1.5361408e-01 1.2198161e-01 9.5878076e-02 6.7368066e-02 1.0481627e-01 1.5125879e-01 2.0371714e-01 2.1419326e-01 1.2396415e-01 -1.4895446e-01 -3.4143873e-01 -3.2050581e-01 -2.3153573e-01 -1.4515718e-01 -8.7382398e-02 -6.9140615e-02 -3.2955981e-02 -2.4053266e-02 8.5548129e-03 4.5502253e-02 5.2930432e-02 2.0810777e-02 6.1875423e-02

2.2465800e+02 -2.3004864e-01 -1.6158976e-01 -1.1073257e-01 -4.8219548e-02 -9.2580939e-03 1.8618194e-02 5.7655162e-02 7.7777092e-02 1.1886037e-01 1.5242115e-01 1.1789235e-01 9.3775628e-02 6.5831412e-02 1.0113687e-01 1.5003860e-01 2.0336492e-01 2.1249556e-01 1.2204700e-01 -1.5064921e-01 -3.5028515e-01 -3.3090180e-01 -2.4358010e-01 -1.4899794e-01 -8.9917799e-02 -7.4986116e-02 -3.4975274e-02 -2.9551607e-02 1.5296601e-02 4.4426445e-02 4.3587729e-02 3.3416639e-02 6.8004731e-02

2.6198300e+02 -1.9934692e-01 -1.4021248e-01 -1.0658055e-01 -4.1880882e-02 -9.5627373e-03 1.7594397e-02 5.4154156e-02 7.6981289e-02 1.1597065e-01 1.5294921e-01 1.1886503e-01 8.8016599e-02 6.2154171e-02 9.8716420e-02 1.4499544e-01 1.9918773e-01 2.0883589e-01 1.2113556e-01 -1.5318217e-01 -3.6189051e-01 -3.4614125e-01 -2.5736813e-01 -1.5936815e-01 -1.0006259e-01 -8.4718990e-02 -3.8439858e-02 -2.9787635e-02 2.0281286e-02 4.7060230e-02 5.8704569e-02 4.3786720e-02 1.0603181e-01

3.0551000e+02 -2.1449767e-01 -1.4656044e-01 -1.0399557e-01 -4.0710810e-02 -1.4124050e-02 1.9212282e-02 5.3874233e-02 7.7945319e-02 1.2179052e-01 1.5766926e-01 1.2291476e-01 9.1305691e-02 6.1774152e-02 1.0132269e-01 1.4474027e-01 1.9865365e-01 2.1277831e-01 1.1821052e-01 -1.6010240e-01 -3.7294402e-01 -3.5532285e-01 -2.5975230e-01 -1.5494607e-01 -1.0043410e-01 -8.7217283e-02 -3.9304425e-02 -3.2526950e-02 2.0087115e-02 4.9204801e-02 3.3948864e-02 5.7039779e-02 1.0748774e-01

3.5626700e+02 -2.0656707e-01 -1.5591808e-01 -1.1210329e-01 -3.6266507e-02 -1.1327357e-02 1.5587006e-02 5.6738904e-02 7.6278461e-02 1.2389360e-01 1.6071136e-01 1.2614926e-01 9.0264223e-02 6.1984987e-02 9.9842379e-02 1.4180866e-01 1.9461787e-01 2.0819278e-01 1.1329228e-01 -1.6970701e-01 -3.7934935e-01 -3.5771198e-01 -2.5539100e-01 -1.5074614e-01 -9.7950725e-02 -8.3178995e-02 -3.6005801e-02 -3.6105594e-02 2.0984902e-02 4.5684688e-02 4.0649492e-02 4.8110093e-02 1.0102766e-01

4.1545800e+02 -2.7081363e-01 -2.0443424e-01 -1.5135705e-01 -6.7562558e-02 -3.2802105e-02 -3.7509307e-03 3.1901503e-02 5.3823842e-02 1.0819250e-01 1.4275899e-01 1.0805824e-01 7.3496620e-02 4.5313572e-02 8.0959624e-02 1.2781972e-01 1.8240587e-01 1.9603417e-01 9.6358102e-02 -1.8501143e-01 -3.7957847e-01 -3.5689366e-01 -2.4349026e-01 -1.3844347e-01 -8.8999748e-02 -7.3728858e-02 -3.0981497e-02 -3.9817318e-02 2.1754076e-02 4.9686794e-02 3.9460322e-02 4.3407857e-02 8.1742970e-02

4.8448300e+02 -2.6547427e-01 -1.8694712e-01 -1.4384811e-01 -6.5285573e-02 -2.6455715e-02 1.8001243e-04 2.5222256e-02 4.8135419e-02 1.0050066e-01 1.3116155e-01 9.0179420e-02 5.7194993e-02 2.8782078e-02 6.4248882e-02 1.0814769e-01 1.6039116e-01 1.6909605e-01 6.4344819e-02 -2.1032735e-01 -3.8305620e-01 -3.5451743e-01 -2.3629604e-01 -1.3551728e-01 -8.9785047e-02 -7.6322838e-02 -3.8264784e-02 -4.5126972e-02 6.8905539e-03 2.7832122e-02 4.1104767e-02 2.4688890e-02 8.3829931e-02

5.6497600e+02 -2.8942435e-01 -1.7423266e-01 -1.2669094e-01 -6.2904771e-02 -2.4988145e-02 1.0847706e-02 1.6801636e-02 4.7112751e-02 9.4953904e-02 1.1636636e-01 7.2877115e-02 4.3832585e-02 1.2560243e-02 5.2429464e-02 8.9653684e-02 1.4025423e-01 1.4923050e-01 3.2852103e-02 -2.3855614e-01 -3.7861734e-01 -3.4652457e-01 -2.1653267e-01 -1.1985780e-01 -8.2121724e-02 -7.2255321e-02 -4.3192610e-02 -3.8378066e-02 -6.0871276e-03 2.3838638e-02 3.5361864e-02 8.0510537e-03 6.9361240e-02

Fig. 2e

6.0000000e-01 6.6454549e-01 3.4661612e-01 1.3573960e-01 8.3792332e-02 5.2593928e-02 2.9749571e-02 -2.7044515e-02 -2.8676136e-02 -5.7881349e-02 -5.4034183e-02 -4.0231855e-02 -6.3511983e-02 -4.3045302e-02 -5.7765865e-02 -3.8145924e-02 -4.7108962e-02 -1.7203232e-02 -1.1288786e-02 1.1209488e-02 3.0555507e-02 7.4214628e-02 4.2850958e-02 8.2883492e-02 2.7965258e-02 3.0174959e-02 -3.8615716e-02 -7.6919404e-03

8.0000000e-01 5.6608103e-01 3.6804381e-01 2.2491316e-01 9.3758459e-02 5.0939520e-02 2.6217548e-02 -2.9252928e-02 -2.1794099e-02 -3.2228592e-02 -2.6818180e-02 -3.2404926e-03 -2.3478717e-02 -1.3823067e-03 -2.0697680e-02 -4.2155896e-03 -1.8035148e-02 3.7413589e-04 -4.1756643e-03 8.1820768e-03 1.1505180e-02 3.7528304e-02 4.3332567e-03 3.3112642e-02 -1.6049496e-02 -2.3799023e-02 -2.2764400e-02 5.0539964e-02

1.0000000e+00 3.6506962e-01 2.8434068e-01 1.6429803e-01 9.5113058e-02 -2.4379266e-03 7.9745584e-03 -4.0062195e-02 -3.0149127e-02 -1.4737452e-02 -9.0876892e-03 1.6241966e-02 2.0092802e-03 2.5361281e-02 5.4872338e-03 2.1964610e-02 6.1724004e-03 1.7593086e-02 5.9409512e-03 1.1286398e-02 7.3352565e-03 1.6798432e-02 -1.1964778e-02 3.3106314e-03 -3.8817642e-02 -2.9386560e-02 -1.9243087e-02 1.9095792e-02

1.2000000e+00 5.2358121e-01 3.2715589e-01 1.9428367e-01 1.0140937e-01 1.9312567e-02 -4.1421129e-03 -5.1331334e-02 -3.7857773e-02 -1.9078486e-02 -1.4520596e-02 1.6227001e-02 6.9572834e-03 2.7305407e-02 8.2790159e-03 2.4379218e-02 9.3935658e-03 1.4509700e-02 2.7259980e-03 6.4002233e-03 2.3268399e-03 4.7639963e-03 -1.9476489e-02 -2.8801440e-03 -3.4098522e-02 -2.5177548e-02 1.9611466e-02 1.1321415e-01

1.4000000e+00 3.8043189e-01 2.1657774e-01 5.4257815e-02 3.4571297e-02 -1.3512973e-02 -2.9691850e-02 -5.9337916e-02 -5.2621847e-02 -1.7355772e-02 -1.3421637e-02 1.5963439e-02 9.0161392e-03 2.9156927e-02 1.1698692e-02 2.5155394e-02 1.0856381e-02 1.4613369e-02 1.2307600e-03 4.1716243e-03 -1.7383134e-03 -1.2776884e-03 -2.0071176e-02 -1.1441173e-02 -2.9758472e-02 -1.5331944e-02 3.5711178e-03 3.6532658e-02

1.6000000e+00 1.3699593e-01 1.8782579e-02 -2.9406010e-04 -9.4001436e-02 -3.1581050e-02 -5.4432821e-02 -6.4285447e-02 -4.2224436e-02 -4.6177976e-03 3.3292669e-03 3.6579340e-02 3.0580766e-02 5.3053633e-02 3.6568082e-02 4.6140628e-02 3.3058136e-02 3.3537145e-02 1.8756513e-02 2.0390074e-02 7.4182872e-03 5.1518936e-03 -1.2317432e-02 -5.8164259e-03 -1.5460911e-02 -3.9546477e-02 -1.9856154e-02 -4.7424758e-02

1.8500000e+00 -1.6139413e-01 -7.3790316e-02 -8.1463692e-02 -1.1005093e-01 -5.2791613e-02 -1.5757962e-02 -2.5994849e-02 8.8661556e-03 4.3287425e-02 5.7749909e-02 8.0970438e-02 7.2700034e-02 9.1822217e-02 7.3103978e-02 7.5566430e-02 5.8870779e-02 5.4303980e-02 3.5715710e-02 3.2599020e-02 8.1123213e-03 1.4836970e-03 -1.2000775e-02 -2.1990039e-02 -3.8214739e-02 -7.6385235e-02 -9.1237280e-02 -2.0374303e-01

2.1000000e+00 -1.2812910e-01 -1.0813149e-01 -4.1086495e-02 -1.1836167e-01 -6.2916120e-02 -1.2162229e-02 -4.7903681e-02 5.1211624e-03 3.8565566e-02 6.1927722e-02 8.3907724e-02 7.6756533e-02 9.6565961e-02 8.1622305e-02 8.4349328e-02 6.9068904e-02 6.2475661e-02 4.2197968e-02 3.9088243e-02 1.3203940e-02 1.4631984e-03 -1.1931507e-02 -2.0176711e-02 -4.1842311e-02 -8.3810241e-02 -1.0598882e-01 -1.7591410e-01

2.3500000e+00 1.1648012e-01 -3.5382379e-02 -7.1478925e-02 -9.1771145e-02 -6.0567984e-02 -1.9734747e-02 -7.4845462e-02 -3.1439244e-02 6.6323372e-03 3.9686905e-02 5.7717578e-02 5.4497281e-02 7.4517722e-02 6.2889039e-02 6.6645111e-02 5.3329206e-02 4.5742632e-02 2.8506383e-02 2.7451460e-02 5.0538532e-03 -2.1198053e-03 -1.9949254e-02 -1.7359616e-02 -4.7598745e-02 -6.6550234e-02 -6.7052431e-02 -2.3239445e-02

2.6000000e+00 6.4513791e-02 -1.2740124e-01 -2.4227752e-02 -6.6504716e-02 -2.7298398e-02 -2.3679043e-02 -6.6872250e-02 -2.9653336e-02 1.5381965e-02 4.4042141e-02 6.0102877e-02 5.4783357e-02 7.4240913e-02 6.3255999e-02 6.5574169e-02 5.1768058e-02 4.3017364e-02 2.3356826e-02 2.2836213e-02 3.5839684e-03 -3.9611356e-03 -2.8027679e-02 -2.5233608e-02 -4.8509250e-02 -6.2566988e-02 -4.7844279e-02 -3.1594657e-02

2.8500000e+00 -5.3420236e-02 -2.3936307e-01 -9.3899932e-02 -7.1897231e-02 -2.5234610e-02 -2.3134655e-02 -3.7644929e-02 -1.4649677e-02 3.6878813e-02 6.5057508e-02 7.8053604e-02 7.1697499e-02 8.6033764e-02 7.3303789e-02 7.1803428e-02 5.5821305e-02 4.5419133e-02 2.3928692e-02 2.2200145e-02 9.1601771e-04 -9.8251444e-03 -2.4943464e-02 -2.7688746e-02 -4.3363153e-02 -6.6294226e-02 -4.4953891e-02 -1.0027147e-01

3.1000000e+00 -2.8728885e-01 -3.2896302e-01 -1.1476736e-01 -9.4413047e-02 -4.6147878e-02 -4.1674929e-02 -4.7608280e-02 -1.2375042e-02 3.8047609e-02 6.4428069e-02 8.0036947e-02 7.1734369e-02 8.5610289e-02 7.3316130e-02 7.0583098e-02 5.5454157e-02 4.5681681e-02 2.3829098e-02 2.4676207e-02 1.6548293e-03 -1.3137876e-02 -1.8762093e-02 -2.6312694e-02 -4.3199925e-02 -6.1481412e-02 -3.2185846e-02 -1.2647599e-01

3.3500000e+00 -3.4841561e-01 -3.5991503e-01 -1.8942368e-01 -1.0297875e-01 -7.8556988e-02 -3.5116743e-02 -4.2594009e-02 -4.1127889e-03 4.5355873e-02 7.9551194e-02 9.6867286e-02 8.6180802e-02 9.8818573e-02 8.5442249e-02 8.1921612e-02 6.8836555e-02 5.6592391e-02 3.2008504e-02 2.9466330e-02 5.7376721e-03 -1.1000982e-02 -1.8826475e-02 -2.3760997e-02 -4.5550429e-02 -6.9308358e-02 -6.8229425e-02 -1.4908100e-01

3.7500000e+00 -5.0152761e-01 -4.0676374e-01 -2.3959884e-01 -1.1575955e-01 -1.0023940e-01 -2.2594605e-02 -4.2022990e-02 1.9345674e-03 4.5060469e-02 8.0166023e-02 9.9750089e-02 8.8754379e-02 1.0385010e-01 8.9749945e-02 8.5328704e-02 7.4931944e-02 6.0410304e-02 3.6411816e-02 3.2623218e-02 1.1021272e-02 -7.9545870e-03 -1.9021264e-02 -2.5675687e-02 -5.3092150e-02 -7.0569665e-02 -8.3251968e-02 -1.5299720e-01

4.1500000e+00 -4.2059790e-01 -3.1435193e-01 -1.9755531e-01 -1.2005635e-01 -8.4936640e-02 -2.6372004e-02 -3.2836938e-02 -3.4412794e-03 3.8574749e-02 7.5413513e-02 9.7447273e-02 8.5223818e-02 9.8622445e-02 8.6762600e-02 8.1743189e-02 7.1380222e-02 5.5885462e-02 3.2097070e-02 2.4714587e-02 5.1655855e-03 -1.5913681e-02 -2.3457960e-02 -3.0815349e-02 -4.0789641e-02 -5.5691004e-02 -9.2915662e-02 -1.1196357e-01

4.5500000e+00 -3.4669875e-01 -2.2492971e-01 -1.7138642e-01 -1.1381293e-01 -5.9698269e-02 -2.4796447e-02 -3.8031180e-02 -9.2929079e-03 3.5185173e-02 6.8722746e-02 9.0308681e-02 7.9975012e-02 9.3900241e-02 8.4160655e-02 7.6772303e-02 6.4379308e-02 5.1115847e-02 2.8236140e-02 2.3051756e-02 8.0160634e-04 -2.3684408e-02 -2.0316077e-02 -3.3929894e-02 -4.3675405e-02 -5.3916548e-02 -7.0566672e-02 -8.9138542e-02

4.9500000e+00 -1.7593747e-01 -7.6300062e-02 -6.7024578e-02 -9.4020692e-02 -1.6183703e-02 -1.6441242e-02 -3.9756968e-02 -3.7179388e-03 4.0377362e-02 7.1883663e-02 8.6770880e-02 7.5324085e-02 8.5918158e-02 7.6421090e-02 6.8061634e-02 5.3634615e-02 3.8680913e-02 1.7228112e-02 1.1151483e-02 -1.5354033e-02 -3.7483506e-02 -3.2384362e-02 -3.9559734e-02 -4.4159141e-02 -5.7008829e-02 -3.4509121e-02 -3.2612092e-02

5.3500000e+00 -3.7728781e-01 -2.1243484e-01 -1.4167581e-01 -8.1633511e-02 -5.1816228e-02 4.0865038e-03 -3.8541788e-02 1.4474780e-02 5.7657749e-02 9.1851949e-02 1.0644547e-01 9.6228965e-02 1.0619263e-01 9.6546157e-02 8.5322748e-02 7.1206389e-02 5.4391510e-02 2.9572302e-02 2.4223358e-02 -8.1796774e-03 -2.9453122e-02 -3.0406610e-02 -4.4952105e-02 -6.0314912e-02 -8.5685365e-02 -4.1445499e-02 -1.1770907e-01

5.7500000e+00 -4.8613009e-01 -2.6985616e-01 -9.9058990e-02 -7.1830573e-02 -6.6339806e-02 -1.3611807e-03 -3.1880823e-02 7.4138019e-03 4.8184861e-02 8.4306452e-02 9.7703575e-02 9.2862440e-02 1.0039844e-01 9.1960454e-02 8.2636505e-02 7.0220722e-02 5.1567331e-02 2.8782960e-02 2.2933630e-02 -6.0412712e-03 -2.8018333e-02 -2.9998808e-02 -3.8895624e-02 -5.2799732e-02 -6.6974120e-02 -3.6605828e-02 -1.4405829e-01

6.1500000e+00 -6.1130816e-01 -3.8107926e-01 -1.7955939e-01 -8.7544116e-02 -1.0793494e-01 -1.7167690e-02 -4.3659842e-02 -1.0026269e-02 2.9926088e-02 6.9880187e-02 8.8899518e-02 8.7474084e-02 9.6197038e-02 9.0575664e-02 8.0451686e-02 6.9944247e-02 5.1776894e-02 2.9990553e-02 2.4060185e-02 1.4150958e-03 -2.3477405e-02 -2.6053024e-02 -3.7173831e-02 -5.6951602e-02 -4.5997170e-02 -3.8054100e-02 -1.4344637e-01

6.5500000e+00 -4.7862319e-01 -3.6150683e-01 -1.4491530e-01 -7.7579962e-02 -1.0179054e-01 -2.0322144e-02 -4.9030286e-02 -8.0519256e-03 2.9740174e-02 7.3760087e-02 8.7239368e-02 8.4628360e-02 9.5034506e-02 8.6231331e-02 7.7253752e-02 6.6580065e-02 4.6325920e-02 2.4548646e-02 1.7269230e-02 3.9730394e-04 -2.7482534e-02 -2.8028069e-02 -4.0759147e-02 -4.9288352e-02 -3.9845354e-02 -3.5270917e-02 -1.1040291e-01

7.6380000e+00 -5.1341599e-01 -4.4071492e-01 -2.3068460e-01 -1.2199303e-01 -1.0596081e-01 -2.3863575e-02 -5.9678962e-02 -8.7960166e-03 3.8023124e-02 8.4531533e-02 1.0054247e-01 9.3690356e-02 1.0507808e-01 9.5455304e-02 8.3618465e-02 7.3010395e-02 5.1806317e-02 2.9226824e-02 2.0526528e-02 4.8537907e-04 -2.7998684e-02 -2.9640081e-02 -5.3818605e-02 -4.5547765e-02 -4.5822135e-02 -4.0378698e-02 -1.3121852e-01

8.9070000e+00 -6.1095860e-01 -5.6617196e-01 -3.3315366e-01 -1.7641829e-01 -1.6789609e-01 -6.4686664e-02 -8.0698995e-02 -1.6318503e-02 3.0250611e-02 8.6080002e-02 1.0256482e-01 9.4960014e-02 1.0912133e-01 1.0153925e-01 9.1004034e-02 8.3165627e-02 6.3809699e-02 4.2048601e-02 3.5871689e-02 1.4598642e-02 -1.5339554e-02 -1.5588951e-02 -4.7274984e-02 -3.0860823e-02 -5.4748376e-02 -8.6944972e-02 -1.6521337e-01

1.0387000e+01 -6.0715075e-01 -5.6871948e-01 -3.5661733e-01 -2.1700610e-01 -1.5143291e-01 -7.5828174e-02 -8.1025677e-02 -2.6811932e-02 3.4374133e-02 9.0847134e-02 1.0701055e-01 1.0214535e-01 1.1195298e-01 1.0656768e-01 9.3564431e-02 8.7196317e-02 6.7129797e-02 4.4431373e-02 3.9987906e-02 1.6053929e-02 -1.7692034e-02 -1.4051480e-02 -4.4190610e-02 -3.1271671e-02 -3.8023309e-02 -1.0251624e-01 -2.3188593e-01

1.2113000e+01 -8.0918513e-01 -4.8687860e-01 -3.8808522e-01 -2.3727485e-01 -1.7145129e-01 -7.1986668e-02 -8.4980101e-02 -2.7036430e-02 4.0985434e-02 9.4599141e-02 1.0819621e-01 1.0456900e-01 1.1399035e-01 1.0882491e-01 9.7689094e-02 9.1529394e-02 6.9164712e-02 4.6217662e-02 4.4583223e-02 2.1309389e-02 -1.4400514e-02 -8.9608423e-03 -3.2865981e-02 -3.9389692e-02 -3.9170791e-02 -1.3191549e-01 -2.2631476e-01

1.4125000e+01 -7.1848575e-01 -3.0399577e-01 -2.3866317e-01 -1.4122119e-01 -4.7441131e-02 2.7009166e-03 -2.6248311e-02 2.9291378e-02 9.1249594e-02 1.3847333e-01 1.4470557e-01 1.3671665e-01 1.3839014e-01 1.2597190e-01 1.0712133e-01 9.6967024e-02 6.5093426e-02 3.5711128e-02 2.8038517e-02 -6.7358123e-04 -4.3260916e-02 -3.7918501e-02 -5.7422967e-02 -7.4168906e-02 -5.6993892e-02 -1.2627250e-01 -1.7021930e-01

1.6472000e+01 -9.7032863e-01 -4.3695354e-01 -3.7615481e-01 -1.8745000e-01 -1.6388966e-01 -7.0342619e-02 -8.2466383e-02 -2.1330837e-02 4.1726058e-02 9.4290242e-02 1.1217686e-01 1.0437456e-01 1.1139349e-01 1.0423357e-01 8.8930525e-02 8.3251147e-02 5.5836636e-02 3.1736240e-02 2.7212573e-02 6.4626489e-04 -3.5409053e-02 -2.5275099e-02 -3.6826618e-02 -4.9247731e-02 -3.4268947e-02 -7.5142505e-02 -1.3169439e-01

1.9209000e+01 -8.1091695e-01 -4.3397810e-01 -3.1702039e-01 -1.6520352e-01 -1.4904120e-01 -7.6675822e-02 -8.4906150e-02 -2.3592385e-02 3.7135832e-02 9.8329385e-02 1.1269899e-01 1.0448817e-01 1.1000424e-01 1.0583661e-01 8.6515102e-02 8.2950920e-02 5.5795655e-02 3.0421249e-02 2.5603316e-02 -1.7505330e-03 -4.0706048e-02 -2.6886346e-02 -3.9084260e-02 -4.8991020e-02 -4.2581928e-02 -5.3379369e-02 -1.2992216e-01

2.2400000e+01 -8.5077773e-01 -4.7728272e-01 -3.4764525e-01 -1.7384736e-01 -1.6196938e-01 -7.5560973e-02 -7.2865870e-02 -4.0974858e-02 3.7722834e-02 9.8662512e-02 1.1147733e-01 1.0478157e-01 1.1004136e-01 1.0790620e-01 8.9454398e-02 8.6476967e-02 5.8556973e-02 3.2333181e-02 3.0095699e-02 -6.8069516e-04 -3.8654912e-02 -2.3685556e-02 -4.2296277e-02 -3.7561115e-02 -4.8307844e-02 -3.9938651e-02 -1.9550648e-01

2.6122000e+01 -8.1948713e-01 -4.9143435e-01 -3.2574289e-01 -2.2042834e-01 -1.4053889e-01 -6.5132019e-02 -6.6454188e-02 -2.8871241e-02 5.3000593e-02 1.0978499e-01 1.1579517e-01 1.1478122e-01 1.2051970e-01 1.1774266e-01 9.7006262e-02 9.4789095e-02 6.5887825e-02 3.8196841e-02 3.4748903e-02 2.5747971e-03 -4.0618687e-02 -2.4927433e-02 -4.9575682e-02 -5.2996375e-02 -6.6131170e-02 -5.8283454e-02 -2.2091902e-01

3.0462000e+01 -9.8050153e-01 -6.5414259e-01 -4.2893747e-01 -2.5763582e-01 -1.8322886e-01 -9.7882698e-02 -7.7775752e-02 -3.9145424e-02 4.8684521e-02 1.0443860e-01 1.1679390e-01 1.1663539e-01 1.2732959e-01 1.2304149e-01 1.0594556e-01 1.0190641e-01 7.5398076e-02 4.7767992e-02 4.4732669e-02 7.5199904e-03 -2.8423133e-02 -1.5514780e-02 -4.7926699e-02 -4.4658659e-02 -5.9674457e-02 -8.4865939e-02 -2.6390401e-01

3.5523000e+01 -8.9502352e-01 -5.5632863e-01 -3.6535472e-01 -2.0541916e-01 -1.6094313e-01 -7.4743290e-02 -6.7329869e-02 -1.5833673e-02 6.4353871e-02 1.1622267e-01 1.2867729e-01 1.2612588e-01 1.3372287e-01 1.2916887e-01 1.0752606e-01 1.0173711e-01 7.3179807e-02 4.3975773e-02 3.6992375e-02 -3.6009277e-03 -4.2520612e-02 -3.5003105e-02 -6.2302805e-02 -6.3580425e-02 -7.1134679e-02 -7.4729627e-02 -1.3971169e-01

4.1425000e+01 -8.0370951e-01 -4.7749449e-01 -3.2286755e-01 -1.5258697e-01 -1.1686336e-01 -5.9543113e-02 -6.3409695e-02 -1.9797555e-03 6.6915191e-02 1.2745105e-01 1.3953113e-01 1.3377006e-01 1.3879948e-01 1.3042099e-01 1.1023875e-01 1.0021352e-01 7.0011587e-02 3.9116738e-02 2.9781599e-02 -1.3575072e-02 -5.0540071e-02 -4.3985764e-02 -7.6279775e-02 -6.7059670e-02 -9.7374974e-02 -1.0352485e-01 -5.8722460e-02

4.8307000e+01 -5.5744569e-01 -3.2379135e-01 -2.6819333e-01 -7.8561190e-02 -6.7082422e-02 -1.8919843e-02 -3.5311317e-02 2.2717201e-02 7.5352950e-02 1.3816066e-01 1.4401416e-01 1.3610170e-01 1.3762557e-01 1.3049306e-01 1.0414911e-01 9.2599252e-02 5.6938288e-02 2.4098902e-02 1.1278640e-02 -3.0299108e-02 -7.8134469e-02 -6.6993439e-02 -9.5424239e-02 -9.5771998e-02 -1.1964477e-01 -5.0206568e-02 3.1549886e-03

5.6333000e+01 -5.3797409e-01 -2.7807081e-01 -2.3703821e-01 -9.8646856e-02 -6.0414591e-02 -3.0658238e-02 -4.0194669e-02 2.8663820e-02 8.4157460e-02 1.5015364e-01 1.5979584e-01 1.5357499e-01 1.5645436e-01 1.4875924e-01 1.2487588e-01 1.1396982e-01 7.8183526e-02 4.4975337e-02 3.3925128e-02 -5.0203820e-03 -5.4703325e-02 -4.5780036e-02 -7.3234406e-02 -9.5006569e-02 -1.1778792e-01 -9.4963800e-02 -1.4070351e-01

6.5692000e+01 -4.5364053e-01 -1.7740602e-01 -2.0773461e-01 -1.0560732e-01 -6.9458829e-02 -3.0211521e-02 -2.2711199e-02 2.0798194e-02 7.9878872e-02 1.3814916e-01 1.5194681e-01 1.4355625e-01 1.4608160e-01 1.4208230e-01 1.1647136e-01 1.0707343e-01 7.0224993e-02 3.8431766e-02 2.7609957e-02 -1.0354073e-02 -6.4106168e-02 -5.4773396e-02 -7.4479405e-02 -9.8346770e-02 -1.1165668e-01 -6.4359957e-02 -1.0637806e-01

7.6606000e+01 -4.8466636e-01 -1.7274303e-01 -2.0533601e-01 -1.4025646e-01 -8.0825903e-02 -3.6945901e-02 -2.6754469e-02 1.7407225e-02 8.0318818e-02 1.4434704e-01 1.6009036e-01 1.5405660e-01 1.5538189e-01 1.5151335e-01 1.2740968e-01 1.1799482e-01 8.0759248e-02 4.9214310e-02 3.6292168e-02 -3.2935411e-03 -5.7786393e-02 -5.3795531e-02 -7.3914688e-02 -8.9765531e-02 -1.0835702e-01 -8.9872414e-02 -1.5074430e-01

8.9333000e+01 -4.0638754e-01 -1.4604539e-01 -2.2801287e-01 -1.1349968e-01 -7.5697200e-02 -2.8014823e-02 -2.6847049e-02 1.5282825e-02 7.5000993e-02 1.3697210e-01 1.5274552e-01 1.4612902e-01 1.4941504e-01 1.4544698e-01 1.2049986e-01 1.1047995e-01 7.2899748e-02 4.0774937e-02 2.5991717e-02 -1.6195231e-02 -7.3583765e-02 -6.3639966e-02 -8.8916492e-02 -1.0012193e-01 -1.1931749e-01 -5.9745989e-02 -6.9134576e-02

1.0417500e+02 -4.7559498e-01 -1.4000575e-01 -1.9227973e-01 -9.6047146e-02 -7.5136857e-02 -2.7230716e-02 -4.0739302e-02 -3.1002986e-03 6.7835127e-02 1.3557062e-01 1.4507559e-01 1.4169619e-01 1.4949425e-01 1.4426895e-01 1.2053277e-01 1.1041877e-01 7.3941619e-02 3.9890176e-02 2.7065836e-02 -1.4401508e-02 -6.8203817e-02 -5.9400595e-02 -8.5527575e-02 -9.6887072e-02 -1.1268800e-01 -6.3954556e-02 -7.0283903e-02

1.2148300e+02 -4.5296292e-01 -1.8488318e-01 -1.6450752e-01 -7.4309728e-02 -7.3404453e-02 -1.6460282e-02 -3.4239120e-02 9.4975721e-04 7.1467988e-02 1.3236941e-01 1.4421570e-01 1.3915764e-01 1.4637378e-01 1.3900435e-01 1.1390497e-01 1.0309654e-01 6.7391265e-02 3.0594269e-02 1.7362902e-02 -2.3819609e-02 -7.7417860e-02 -7.0418620e-02 -1.0058919e-01 -1.1472175e-01 -1.2359739e-01 -8.0101447e-02 2.2025462e-02

1.4166700e+02 -6.7888854e-01 -3.3032629e-01 -1.7753732e-01 -1.2114787e-01 -1.0235806e-01 -7.3869263e-03 -1.5390818e-02 1.1970210e-02 8.8550024e-02 1.4828236e-01 1.6030580e-01 1.5481369e-01 1.6145849e-01 1.5293603e-01 1.2770449e-01 1.1509104e-01 8.0096122e-02 4.2368799e-02 2.9885013e-02 -1.4688080e-02 -6.3817142e-02 -6.1161674e-02 -9.4040703e-02 -9.2312474e-02 -1.1632146e-01 -9.4725735e-02 -9.5488289e-02

1.6520400e+02 -8.8742341e-01 -5.4215238e-01 -3.6031416e-01 -2.1960664e-01 -1.8084839e-01 -5.2535727e-02 -4.9933378e-02 -6.1679074e-03 6.7262267e-02 1.3070483e-01 1.5074651e-01 1.4840877e-01 1.5658935e-01 1.5177465e-01 1.2784201e-01 1.1919798e-01 8.7371340e-02 5.3905056e-02 4.1623588e-02 -2.3214624e-03 -4.8975374e-02 -3.5730323e-02 -7.0023409e-02 -6.9436999e-02 -8.7776746e-02 -9.4709609e-02 -1.9429587e-01

1.9265100e+02 -9.0248918e-01 -5.0676488e-01 -3.2089218e-01 -2.2042457e-01 -1.6486723e-01 -5.4922081e-02 -3.9288169e-02 -1.0338367e-02 7.0819575e-02 1.2807866e-01 1.4781761e-01 1.4780882e-01 1.5407169e-01 1.5036621e-01 1.2630030e-01 1.1677379e-01 8.5724706e-02 5.1919574e-02 4.2717086e-02 -2.2576034e-03 -4.7974559e-02 -3.6190422e-02 -5.5542757e-02 -6.1555408e-02 -8.1503845e-02 -9.7067183e-02 -2.6360619e-01

2.2465800e+02 -6.2324283e-01 -3.9609862e-01 -2.2468914e-01 -1.2518049e-01 -9.6778971e-02 -8.7731723e-03 -1.1586653e-02 2.9606652e-02 1.0035239e-01 1.5649741e-01 1.7089438e-01 1.6688645e-01 1.7090514e-01 1.6403739e-01 1.3676547e-01 1.2285896e-01 9.0298743e-02 5.1228604e-02 3.6099904e-02 -9.3987930e-03 -6.1193077e-02 -4.5851380e-02 -6.3098996e-02 -7.8203437e-02 -1.1843679e-01 -1.2142638e-01 -2.1089116e-01

2.6198300e+02 -1.8125040e-01 -1.8084304e-01 -5.8309944e-02 -8.1194302e-02 -7.1730565e-02 -3.3431848e-02 -4.6149978e-02 -1.1897544e-02 6.2958059e-02 1.1460226e-01 1.3284558e-01 1.2803898e-01 1.3366909e-01 1.2788891e-01 1.0404006e-01 9.1565447e-02 5.9987216e-02 2.1460742e-02 1.0806931e-02 -3.2402475e-02 -8.4508913e-02 -6.8490695e-02 -8.0346022e-02 -8.8916670e-02 -1.0580535e-01 -7.4807035e-02 3.6493649e-02

3.0551000e+02 -9.8784851e-02 -1.4201301e-01 -4.6891849e-02 -7.6604818e-02 -6.6426575e-02 -1.2879694e-02 -4.5215719e-02 7.7067766e-03 7.4000150e-02 1.3268564e-01 1.4971131e-01 1.3995637e-01 1.4457179e-01 1.3460892e-01 1.0848213e-01 9.6371451e-02 6.1472880e-02 2.1392671e-02 9.7229837e-03 -3.7739475e-02 -9.0706147e-02 -7.2885973e-02 -9.6927753e-02 -1.0639283e-01 -1.2581812e-01 -7.8647103e-02 4.5902458e-02

3.5626700e+02 5.6125839e-03 -7.7964491e-02 -4.5660187e-02 -1.0507038e-01 -5.3691224e-02 -2.6197524e-02 -5.4111137e-02 -1.8828676e-02 5.6271437e-02 1.1200627e-01 1.2573367e-01 1.1267355e-01 1.1603021e-01 1.0581928e-01 7.9696029e-02 6.9314679e-02 3.6151361e-02 -6.3690225e-04 -5.9922241e-03 -5.1076877e-02 -9.8204317e-02 -8.3467536e-02 -1.0586208e-01 -1.0192708e-01 -1.0625992e-01 -2.4520596e-02 1.9325593e-01

4.1545800e+02 -2.3186320e-01 -1.5817291e-01 -9.1902302e-02 -1.0663775e-01 -3.9199906e-02 2.9205752e-03 -2.3048011e-02 4.0291984e-03 7.4547587e-02 1.3553302e-01 1.3861514e-01 1.2208132e-01 1.1829230e-01 1.0640583e-01 7.7879623e-02 6.5616337e-02 3.1628870e-02 -2.8276577e-03 -4.5832653e-03 -4.8360802e-02 -9.2158969e-02 -8.2359675e-02 -1.0103239e-01 -9.6470818e-02 -1.3132961e-01 -2.0026698e-02 1.2217304e-01

4.8448300e+02 -1.4744699e-01 -1.0660815e-01 -3.1024842e-02 -4.0029792e-02 1.8977728e-02 9.3305295e-03 -1.0330863e-02 -2.0435805e-03 6.6335554e-02 1.1686233e-01 1.1649179e-01 9.1959515e-02 8.7188965e-02 7.3623089e-02 4.8004320e-02 3.7384211e-02 7.2060844e-03 -1.6711322e-02 -1.0003542e-02 -4.4221397e-02 -8.1543680e-02 -6.0003171e-02 -7.0323322e-02 -4.9387932e-02 -8.6803167e-02 7.0056765e-02 2.4285460e-01

5.6497600e+02 -2.9175118e-01 -1.9025194e-01 -7.2293350e-02 -2.3550155e-02 3.7009892e-03 -2.6385844e-02 -2.9890206e-02 -2.7717622e-02 2.7653388e-02 7.8225628e-02 8.4759377e-02 5.6089703e-02 5.1690289e-02 3.8372582e-02 1.8782283e-02 1.3525998e-02 -1.3202101e-02 -2.1831123e-02 -3.9841372e-03 -2.7675176e-02 -5.8273915e-02 -1.8698681e-02 -2.1618626e-02 -4.2164020e-03 -2.9800666e-02 1.2305224e-01 1.9428930e-01

Fig. 2f

6.0000000e-01 1.3595315e-01 1.3168214e-01 -3.8509087e-02 -6.6178711e-02 -1.2888991e-01 -1.1173169e-01 2.8187713e-03 5.9031812e-02 1.3313743e-01 2.4767802e-02 -1.5449572e-02 -1.4832235e-01 -1.3915717e-01 -1.2957070e-01 -7.5765757e-02 1.2510169e-01 1.7719100e-01 1.0751752e-01 4.5792959e-02 -4.7485493e-02 -1.3739663e-01 -2.8737425e-02 -1.7182329e-02 8.0942695e-02 1.1311353e-01 1.3690938e-01 1.6109964e-02 -1.8382330e-02 -9.3864784e-02 -8.4630009e-02 -2.3997279e-02 3.8486965e-02

8.0000000e-01 1.0893341e-01 1.0034749e-01 -4.7840455e-02 -6.9772986e-02 -1.1388966e-01 -8.8933351e-02 8.8673801e-03 6.4957989e-02 1.1404860e-01 1.7793991e-02 -2.4685703e-02 -1.3553903e-01 -1.1825652e-01 -1.0124072e-01 -4.4121821e-02 1.2305930e-01 1.7973111e-01 9.6474089e-02 3.3929034e-02 -4.7610248e-02 -1.0533195e-01 -1.1679921e-02 5.6519752e-03 8.0048887e-02 1.0010930e-01 1.0396202e-01 -2.4821268e-03 -3.3927656e-02 -9.4249749e-02 -8.0568915e-02 -2.4214332e-02 3.3719159e-02

1.0000000e+00 9.0949551e-02 7.6190611e-02 -5.6278134e-02 -7.3696152e-02 -1.0301058e-01 -7.1358215e-02 1.7092476e-02 6.9026028e-02 1.0353531e-01 1.2325199e-02 -3.2181773e-02 -1.2896073e-01 -1.0634313e-01 -8.1913097e-02 -2.2878423e-02 1.2186477e-01 1.8488436e-01 9.4354567e-02 3.0419930e-02 -4.6561332e-02 -8.2922004e-02 -5.4120184e-04 1.8087293e-02 7.8708033e-02 8.7631905e-02 8.0973129e-02 -1.6703405e-02 -4.2442474e-02 -8.9757189e-02 -7.4284657e-02 -2.5925319e-02 2.5471383e-02

1.2000000e+00 7.3956445e-02 6.2512796e-02 -5.2019514e-02 -6.5249589e-02 -9.0234528e-02 -6.0262764e-02 1.3928271e-02 6.1931478e-02 8.7516094e-02 9.6480221e-03 -3.3508499e-02 -1.1723823e-01 -9.4168266e-02 -7.2648033e-02 -1.5754956e-02 1.1320421e-01 1.8570570e-01 9.2345429e-02 3.1079975e-02 -3.9033750e-02 -6.5986835e-02 9.0419677e-03 2.6947571e-02 7.5269891e-02 7.8700508e-02 6.6724339e-02 -2.2570032e-02 -4.6993355e-02 -8.8754379e-02 -6.8590850e-02 -2.7786516e-02 2.1150210e-02

1.4000000e+00 7.2622998e-02 5.8709338e-02 -5.0517383e-02 -6.1293592e-02 -8.1975050e-02 -4.9856515e-02 1.9079919e-02 6.4948162e-02 8.9442791e-02 1.5221381e-02 -3.0118487e-02 -1.0920224e-01 -8.8025623e-02 -6.7172729e-02 -7.9376431e-03 1.1632788e-01 1.9513043e-01 1.0547393e-01 3.7157939e-02 -2.8994938e-02 -5.3807122e-02 1.7019973e-02 3.4531601e-02 7.6670682e-02 7.8585804e-02 6.7371556e-02 -2.0468139e-02 -4.0963026e-02 -8.0736513e-02 -5.9473676e-02 -2.8052590e-02 1.9202671e-02

1.6000000e+00 6.8386515e-02 5.1852565e-02 -5.2665053e-02 -6.1144778e-02 -7.7922048e-02 -4.3459301e-02 1.8837361e-02 6.5280502e-02 8.4629355e-02 1.2168818e-02 -3.1291874e-02 -1.0588149e-01 -8.5116786e-02 -6.6596533e-02 -6.8278251e-03 1.1449191e-01 2.0063058e-01 1.1033908e-01 4.0564330e-02 -2.3887767e-02 -4.7256635e-02 2.2548636e-02 3.9531287e-02 7.4020232e-02 7.1915381e-02 6.0739314e-02 -2.0706389e-02 -4.2812815e-02 -7.9036382e-02 -5.7198508e-02 -3.0056944e-02 1.2903561e-02

1.8500000e+00 5.7257736e-02 4.5706979e-02 -5.5345083e-02 -6.2359909e-02 -7.2852954e-02 -3.6816339e-02 2.0412482e-02 6.3920722e-02 8.1201597e-02 8.8959892e-03 -3.1586884e-02 -1.0293514e-01 -8.2609553e-02 -6.2757244e-02 -3.2616551e-03 1.1452010e-01 2.0596792e-01 1.1448501e-01 4.7607281e-02 -1.9675718e-02 -4.0521758e-02 2.7871388e-02 4.4021949e-02 7.2000487e-02 6.5487233e-02 5.6324751e-02 -2.3930060e-02 -4.4095301e-02 -7.6983405e-02 -5.8300470e-02 -3.4942262e-02 6.4994552e-03

2.1000000e+00 4.9988358e-02 3.7273632e-02 -5.6831116e-02 -6.0919034e-02 -6.5837267e-02 -2.9070960e-02 2.4540742e-02 6.3135697e-02 7.7843431e-02 3.2238038e-03 -3.5321323e-02 -1.0169142e-01 -8.1431961e-02 -6.0742076e-02 -4.7985670e-03 1.1323477e-01 2.1494346e-01 1.1910204e-01 5.7512060e-02 -1.6756619e-02 -3.4574121e-02 3.2001093e-02 4.6764899e-02 7.1867838e-02 5.6958122e-02 5.2763629e-02 -2.1407097e-02 -4.3075842e-02 -7.2529057e-02 -5.8584529e-02 -3.8698706e-02 -2.7027055e-03

2.3500000e+00 3.6581636e-02 2.7408452e-02 -5.3707761e-02 -5.3454483e-02 -5.6921194e-02 -2.0623706e-02 2.2304505e-02 5.7462962e-02 7.0328192e-02 3.0010534e-03 -3.7589591e-02 -9.6664880e-02 -7.7239950e-02 -5.7742601e-02 -1.9433023e-03 1.0972748e-01 2.2006295e-01 1.2258497e-01 5.6768757e-02 -1.3098495e-02 -2.6601244e-02 3.7459717e-02 5.1776264e-02 7.1384606e-02 5.3962620e-02 4.8035516e-02 -2.1558825e-02 -4.1317858e-02 -7.1198367e-02 -6.0754138e-02 -4.4604620e-02 -8.6449305e-03

2.6000000e+00 3.1104757e-02 1.6909846e-02 -5.7551212e-02 -5.2468911e-02 -5.1145075e-02 -1.1261195e-02 2.6605572e-02 5.7311740e-02 6.7412488e-02 2.7429986e-03 -4.3124546e-02 -9.6794628e-02 -7.6165729e-02 -5.4351328e-02 2.8914283e-03 1.1209140e-01 2.2954877e-01 1.3132773e-01 5.6256487e-02 -1.2596445e-02 -2.3656201e-02 3.9852083e-02 5.2094272e-02 7.2840563e-02 5.1748824e-02 4.4949922e-02 -2.2085273e-02 -4.0036893e-02 -6.9655065e-02 -6.1503252e-02 -4.6798247e-02 -1.4327508e-02

2.8500000e+00 2.5936114e-02 1.8221712e-02 -5.0616061e-02 -4.7546370e-02 -4.9493714e-02 -1.4927586e-02 1.7600851e-02 4.8085773e-02 6.1707353e-02 6.5634368e-03 -3.4733466e-02 -9.0649508e-02 -7.2246843e-02 -5.3534289e-02 -2.7166113e-04 1.0582822e-01 2.3034322e-01 1.3457618e-01 5.8807958e-02 -4.9629211e-03 -1.6692600e-02 4.7743446e-02 5.4453077e-02 7.0350821e-02 4.5403873e-02 3.6112432e-02 -2.6369974e-02 -4.0868476e-02 -6.8992210e-02 -5.7772972e-02 -4.5441228e-02 -1.5493441e-02

3.1000000e+00 2.3837195e-02 2.4716660e-02 -4.5033132e-02 -4.4092706e-02 -4.5265586e-02 -2.0306295e-02 1.0725981e-02 3.8038721e-02 5.5182409e-02 4.9318630e-03 -2.9636099e-02 -8.2513322e-02 -7.1053319e-02 -5.2979263e-02 -8.8339983e-03 9.9735637e-02 2.3292147e-01 1.3854300e-01 6.9409052e-02 2.7334650e-03 -9.7852629e-03 5.5635371e-02 5.5495203e-02 6.7656405e-02 3.5051816e-02 3.0821945e-02 -2.7516366e-02 -4.2135275e-02 -6.5593935e-02 -5.2908167e-02 -4.5791125e-02 -1.7227292e-02

3.3500000e+00 2.1783196e-02 2.5946937e-02 -3.9502150e-02 -4.0291930e-02 -4.4892597e-02 -2.3229012e-02 4.7626318e-03 3.2082145e-02 5.0786004e-02 5.6566897e-03 -2.4259984e-02 -7.9112202e-02 -6.8736145e-02 -5.2802995e-02 -1.4182597e-02 9.5664204e-02 2.4091823e-01 1.4643864e-01 7.8084602e-02 1.0890934e-02 -2.3118633e-03 6.3772374e-02 5.9503479e-02 6.6011285e-02 2.8262294e-02 2.6543344e-02 -2.4733728e-02 -4.0962082e-02 -6.3818544e-02 -5.1115271e-02 -4.8655754e-02 -2.1841814e-02

3.7500000e+00 1.8723824e-02 2.3166867e-02 -3.9314460e-02 -4.0646696e-02 -4.1935715e-02 -1.7936385e-02 3.7047571e-03 2.9577846e-02 4.6224351e-02 5.5934075e-03 -2.4907512e-02 -7.4844266e-02 -6.7831714e-02 -5.2512712e-02 -1.5435781e-02 9.6437559e-02 2.5201712e-01 1.5689273e-01 8.3492193e-02 1.4800338e-02 4.4146492e-03 6.9374951e-02 6.2283734e-02 6.2886491e-02 2.4233383e-02 2.5546905e-02 -2.5312779e-02 -3.9358171e-02 -6.2705772e-02 -4.9901412e-02 -5.1826924e-02 -2.6832136e-02

4.1500000e+00 1.6446762e-02 1.8012930e-02 -4.0871870e-02 -4.2861839e-02 -4.4667846e-02 -1.6336757e-02 3.0778971e-03 3.0861253e-02 4.5218451e-02 8.0362967e-03 -2.3921066e-02 -7.3228828e-02 -6.5298175e-02 -5.4639627e-02 -1.3467370e-02 9.9780416e-02 2.6568455e-01 1.6534684e-01 8.8437463e-02 1.8650894e-02 9.8422938e-03 7.3900823e-02 6.6363643e-02 6.0299582e-02 2.2832138e-02 2.2459477e-02 -2.7341235e-02 -3.8257830e-02 -6.2255804e-02 -5.3496988e-02 -5.5227540e-02 -3.3438956e-02

4.5500000e+00 9.4346284e-03 1.5689869e-02 -4.0911484e-02 -3.9737317e-02 -3.8662845e-02 -1.1439952e-02 3.4624836e-03 2.8194917e-02 4.1453473e-02 6.8192763e-03 -2.3589878e-02 -6.8872195e-02 -6.1130542e-02 -5.1821787e-02 -1.2299462e-02 1.0195990e-01 2.7370078e-01 1.6865764e-01 9.3045293e-02 1.8682881e-02 1.4226549e-02 8.0277110e-02 7.2143711e-02 5.9659503e-02 2.1551635e-02 1.8426260e-02 -2.9327467e-02 -3.8435058e-02 -5.9273690e-02 -5.2646870e-02 -5.3971772e-02 -3.5869235e-02

4.9500000e+00 -2.1826926e-03 1.2767803e-02 -3.7412940e-02 -2.9593810e-02 -3.0836358e-02 -8.2381363e-03 1.0316409e-03 2.2640427e-02 3.5865676e-02 4.1271787e-03 -2.2643273e-02 -6.2183217e-02 -5.1480527e-02 -4.6307070e-02 -8.6693815e-03 1.0085634e-01 2.8140756e-01 1.6999109e-01 9.4676070e-02 2.1418299e-02 1.9402924e-02 8.9149089e-02 7.8884607e-02 6.3503958e-02 2.0132292e-02 1.3493758e-02 -3.1225824e-02 -4.0887968e-02 -5.7289185e-02 -5.2156319e-02 -5.3130545e-02 -3.5637036e-02

5.3500000e+00 -7.9543207e-03 1.0809289e-02 -3.4082402e-02 -2.4893954e-02 -2.4721927e-02 -1.2542092e-03 8.6643566e-05 1.8858745e-02 3.1971879e-02 4.8278283e-03 -2.2216620e-02 -5.8570678e-02 -4.6689889e-02 -4.0832795e-02 -6.1106017e-03 9.9817819e-02 2.8760960e-01 1.7626453e-01 9.6506092e-02 2.6958077e-02 2.5802959e-02 9.6668210e-02 8.1737970e-02 6.5968715e-02 2.0163589e-02 1.2049404e-02 -3.1593027e-02 -3.8130888e-02 -5.8386030e-02 -4.8810967e-02 -5.1801213e-02 -3.1692281e-02

5.7500000e+00 -5.5473968e-03 9.2863541e-03 -3.4737273e-02 -2.6049842e-02 -2.5789911e-02 -4.6303989e-04 -1.6047844e-03 1.7270968e-02 3.0191997e-02 6.3734198e-03 -2.0814979e-02 -5.5099747e-02 -4.7571428e-02 -4.2486566e-02 -7.3800360e-03 9.9037290e-02 2.9524721e-01 1.8006551e-01 1.0022434e-01 3.3012822e-02 3.0309427e-02 9.8044628e-02 8.0857896e-02 6.1250372e-02 1.5437198e-02 7.8358524e-03 -3.1972187e-02 -3.7053106e-02 -5.6168422e-02 -4.5681319e-02 -5.4645978e-02 -3.2229580e-02

6.1500000e+00 3.0960937e-04 7.3911518e-03 -3.7248250e-02 -3.7180486e-02 -3.4218777e-02 -1.9940084e-03 -3.9810143e-07 2.3000505e-02 3.7695822e-02 1.4633340e-02 -2.0369150e-02 -6.1369522e-02 -5.6229984e-02 -4.7687406e-02 -1.0158990e-02 1.0406806e-01 3.0740622e-01 1.9150228e-01 1.0536572e-01 3.3103347e-02 2.6994629e-02 9.2680045e-02 7.5511405e-02 5.6723189e-02 1.3641877e-02 6.9119512e-03 -2.8283771e-02 -3.3990471e-02 -5.8217995e-02 -4.7917189e-02 -6.0202895e-02 -3.4925623e-02

6.5500000e+00 -1.2856748e-03 5.0700331e-03 -3.8184417e-02 -4.0531914e-02 -3.9579682e-02 -4.2809390e-03 -1.2123744e-04 2.7074540e-02 3.9864846e-02 1.9571802e-02 -1.8734137e-02 -6.1449845e-02 -5.8800196e-02 -5.0895062e-02 -1.0255647e-02 1.0565318e-01 3.1845256e-01 1.9817862e-01 1.1105649e-01 3.3593995e-02 2.7836594e-02 8.9829767e-02 7.5485323e-02 5.2791740e-02 1.0317499e-02 4.0500811e-03 -2.6540030e-02 -3.4666351e-02 -5.7331394e-02 -4.6984006e-02 -6.2392697e-02 -4.1373310e-02

7.6380000e+00 -9.5496309e-03 5.5161926e-04 -3.7310164e-02 -3.7426875e-02 -3.5683737e-02 4.9169159e-04 7.3685812e-04 2.6878210e-02 3.7978529e-02 1.8602935e-02 -1.7548120e-02 -5.9282846e-02 -5.3086788e-02 -4.6397486e-02 -5.2841468e-03 1.0831636e-01 3.2829850e-01 2.0754340e-01 1.1501953e-01 3.7712027e-02 3.2272363e-02 9.4841343e-02 8.0514441e-02 5.5563800e-02 1.0794563e-02 5.3018769e-03 -2.7675445e-02 -3.6665587e-02 -5.8359065e-02 -4.8559061e-02 -6.2287422e-02 -4.3416991e-02

8.9070000e+00 -1.9969589e-02 -5.6277054e-03 -3.8134458e-02 -3.1398434e-02 -2.6935884e-02 4.4269424e-03 -1.4852024e-03 1.9694770e-02 2.6740617e-02 1.2561098e-02 -2.0467025e-02 -5.2411324e-02 -4.7762698e-02 -4.3252713e-02 -2.2399347e-03 1.0724508e-01 3.3289443e-01 2.0835723e-01 1.2079025e-01 4.4014615e-02 4.0962764e-02 1.0174015e-01 8.5188885e-02 5.3075763e-02 6.8742363e-03 7.1376144e-04 -3.3015203e-02 -4.0581529e-02 -5.7276090e-02 -4.9909753e-02 -6.5327054e-02 -5.4383525e-02

1.0387000e+01 -2.8544180e-02 -1.2198367e-02 -3.3216258e-02 -2.4354784e-02 -1.7601874e-02 9.5012130e-03 -4.0914474e-03 1.1340890e-02 2.0079638e-02 1.1014213e-02 -1.8556967e-02 -4.4398814e-02 -4.0271574e-02 -3.7780155e-02 8.7620341e-04 1.1087593e-01 3.4190579e-01 2.1771421e-01 1.2595763e-01 5.0714150e-02 4.9631880e-02 1.0939493e-01 8.9715394e-02 5.5844731e-02 5.5993830e-03 -6.3320272e-04 -3.6411909e-02 -4.1788285e-02 -5.7474209e-02 -4.7441560e-02 -6.6285547e-02 -5.3815379e-02

1.2113000e+01 -2.8818265e-02 -1.6710518e-02 -3.4156453e-02 -2.6582007e-02 -1.7532116e-02 1.0170622e-02 -5.3530592e-03 1.1920580e-02 1.9674947e-02 1.4915784e-02 -2.1365497e-02 -4.4982816e-02 -4.2118422e-02 -4.3118565e-02 -1.9224904e-03 1.1545089e-01 3.5753200e-01 2.2673294e-01 1.3239746e-01 5.3060006e-02 5.1518998e-02 1.0902288e-01 8.4654408e-02 5.4201701e-02 3.3345281e-03 5.7699915e-04 -3.7707108e-02 -4.3015015e-02 -6.0081273e-02 -4.6715098e-02 -7.0888373e-02 -5.9545976e-02

1.4125000e+01 -3.7814087e-02 -2.3231414e-02 -3.4635694e-02 -2.3279526e-02 -1.4014837e-02 1.7897445e-02 -6.2760531e-04 1.4822757e-02 2.0163612e-02 1.4518042e-02 -2.0948204e-02 -4.4953037e-02 -3.7992037e-02 -4.0128112e-02 5.8018298e-03 1.2621188e-01 3.8016515e-01 2.4028572e-01 1.3684203e-01 5.7423850e-02 5.6636623e-02 1.1085812e-01 8.8072778e-02 5.7724714e-02 5.7067037e-03 1.7055293e-03 -3.9301360e-02 -4.8395513e-02 -6.3682477e-02 -4.4537130e-02 -7.0388348e-02 -5.4043674e-02

1.6472000e+01 -4.6089812e-02 -2.6886983e-02 -3.6578218e-02 -2.3353463e-02 -1.3051991e-02 2.0321924e-02 -2.0123210e-03 1.3844340e-02 1.7357641e-02 1.1027320e-02 -2.1906886e-02 -4.6268233e-02 -3.9559979e-02 -4.1704378e-02 3.1736520e-03 1.3022853e-01 3.9753867e-01 2.4823349e-01 1.4256943e-01 6.1223564e-02 5.7794662e-02 1.0859882e-01 8.4333462e-02 5.3012029e-02 5.9287046e-05 -2.4959926e-03 -4.5292704e-02 -5.5704741e-02 -6.3951325e-02 -4.6954012e-02 -7.0421689e-02 -5.8442574e-02

1.9209000e+01 -5.6954447e-02 -2.5538294e-02 -3.0973152e-02 -1.9103307e-02 -7.1533767e-03 2.3327691e-02 -2.9783829e-03 9.6471249e-03 1.7008273e-02 1.1713457e-02 -1.3771259e-02 -3.5240476e-02 -3.1424126e-02 -3.3071805e-02 8.9085618e-03 1.3960571e-01 4.2022607e-01 2.6735003e-01 1.5810568e-01 7.6595444e-02 7.0963736e-02 1.1380065e-01 9.0619930e-02 5.5952014e-02 2.5109930e-03 -3.8453709e-03 -4.6333979e-02 -5.6953731e-02 -5.9112191e-02 -4.1412255e-02 -6.4385816e-02 -5.2607435e-02

2.2400000e+01 -5.8452378e-02 -2.2819632e-02 -2.3632218e-02 -1.0536755e-02 2.3471419e-03 2.9313194e-02 -4.2065434e-03 6.1786560e-03 1.5588853e-02 1.5476778e-02 -2.7111701e-03 -1.8744256e-02 -1.8651410e-02 -2.1485892e-02 1.1530040e-02 1.4439180e-01 4.4125712e-01 2.8873677e-01 1.7884748e-01 9.6639012e-02 9.1352455e-02 1.2874756e-01 1.0015043e-01 6.7714818e-02 9.9781522e-03 3.4485331e-03 -3.6454273e-02 -4.4926497e-02 -4.1465701e-02 -2.5013447e-02 -4.8302293e-02 -3.6483130e-02

2.6122000e+01 -5.9961745e-02 -2.0129246e-02 -1.9074083e-02 -6.9912050e-03 4.5204425e-03 2.8257248e-02 -6.1123956e-03 1.1622384e-03 1.6495880e-02 2.0264262e-02 3.9717855e-03 -9.8921462e-03 -1.1927460e-02 -1.8085082e-02 1.1730732e-02 1.4761339e-01 4.6148865e-01 3.0901759e-01 1.9489197e-01 1.1064152e-01 1.0490436e-01 1.3363990e-01 1.0134723e-01 7.0773459e-02 1.1155009e-02 5.8336488e-03 -3.3009186e-02 -4.1003725e-02 -3.0834195e-02 -1.1795823e-02 -3.8350164e-02 -2.9360442e-02

3.0462000e+01 -6.4841836e-02 -2.6049370e-02 -1.7258576e-02 1.5512489e-03 1.0597480e-02 3.0129159e-02 -1.2985000e-02 -8.4999505e-03 3.4510257e-03 1.0603315e-02 -2.7602771e-04 -8.7789437e-03 -9.8293730e-03 -1.8226919e-02 5.4068152e-03 1.3759723e-01 4.6959107e-01 3.1580506e-01 1.9728328e-01 1.1546014e-01 1.1067039e-01 1.3005610e-01 9.4770032e-02 6.4240595e-02 1.2115462e-03 -3.7010265e-03 -4.0352032e-02 -4.9186376e-02 -3.8058964e-02 -1.5210699e-02 -4.2838482e-02 -3.6559544e-02

3.5523000e+01 -5.9657675e-02 -2.4723942e-02 -1.5600855e-02 -1.5030464e-03 8.5468526e-03 2.8411881e-02 -1.1910921e-02 -3.5678418e-03 8.5418310e-03 1.4605813e-02 3.9064883e-03 -7.2967555e-03 -1.1116196e-02 -2.0209441e-02 5.7545696e-03 1.4167823e-01 4.9295595e-01 3.3705245e-01 2.1278147e-01 1.2943142e-01 1.1616238e-01 1.2588075e-01 9.1999938e-02 6.2262370e-02 3.0164580e-03 3.0662043e-04 -3.4825055e-02 -4.3515813e-02 -3.2788561e-02 -1.0243873e-02 -4.1036986e-02 -3.6397803e-02

4.1425000e+01 -6.0367241e-02 -3.3788850e-02 -2.8591463e-02 -1.1135388e-02 6.7997342e-03 3.3234268e-02 -4.3550864e-03 3.5650385e-03 7.8393477e-03 8.4178818e-03 -6.4066494e-03 -1.9905534e-02 -1.9735390e-02 -2.3824834e-02 5.2665212e-03 1.4107473e-01 5.0788385e-01 3.4786261e-01 2.1058607e-01 1.2115930e-01 1.0678002e-01 1.1065298e-01 8.0636250e-02 5.6403232e-02 8.2633757e-04 -2.6063552e-03 -4.0078766e-02 -5.2273813e-02 -5.2141077e-02 -2.6947140e-02 -5.7446579e-02 -5.0687970e-02

4.8307000e+01 -5.9404562e-02 -3.6028651e-02 -3.2705865e-02 -1.9084522e-02 3.6410605e-03 3.4537237e-02 1.2870302e-03 7.6207986e-03 1.1326571e-02 9.6003771e-03 -7.8750819e-03 -2.3135770e-02 -2.3321649e-02 -2.4191345e-02 6.3375208e-03 1.4529833e-01 5.1408779e-01 3.5366096e-01 2.1109989e-01 1.1976863e-01 1.0224692e-01 1.0558247e-01 7.4475355e-02 5.5282304e-02 -1.0056176e-03 -3.6560054e-03 -3.9511121e-02 -5.1702891e-02 -5.1983238e-02 -3.0530752e-02 -6.1918350e-02 -5.4970952e-02

5.6333000e+01 -6.4984729e-02 -3.7717482e-02 -3.0236926e-02 -1.6129050e-02 5.5208270e-03 3.6286722e-02 -1.8767777e-04 2.8553167e-03 5.7243976e-03 6.8105932e-03 -9.3563992e-03 -2.3816879e-02 -2.1751145e-02 -2.1505824e-02 5.9714589e-03 1.4428308e-01 5.1115875e-01 3.5349752e-01 2.0962511e-01 1.1809523e-01 1.0416095e-01 1.0561532e-01 7.2459735e-02 5.4237256e-02 -3.7260506e-03 -6.1967836e-03 -4.1765540e-02 -5.6179916e-02 -5.4943837e-02 -3.0533893e-02 -6.1734340e-02 -5.5466764e-02

6.5692000e+01 -6.9108811e-02 -3.3690269e-02 -2.1883118e-02 -7.0560151e-03 8.2807189e-03 3.5013099e-02 -6.2185225e-03 -5.0004590e-03 -1.7770200e-03 6.3064873e-03 -5.3749080e-03 -1.5736841e-02 -1.6228278e-02 -2.1093181e-02 6.4478207e-03 1.4150410e-01 5.0783905e-01 3.5104103e-01 2.1035209e-01 1.2373297e-01 1.0996037e-01 1.1190635e-01 7.7334647e-02 5.4307311e-02 -3.5509257e-03 -8.1513300e-03 -4.1170501e-02 -5.3948701e-02 -4.7169859e-02 -2.5086900e-02 -5.9470414e-02 -4.9803322e-02

7.6606000e+01 -7.2135727e-02 -3.1870052e-02 -1.8041567e-02 -4.4722479e-03 8.5132651e-03 3.5535001e-02 -1.0460009e-02 -6.8250626e-03 -4.5844570e-03 9.5955144e-03 -3.2151063e-03 -1.4070274e-02 -1.4400609e-02 -2.3278061e-02 5.4274214e-03 1.3883956e-01 5.1160008e-01 3.5494504e-01 2.1322349e-01 1.2589685e-01 1.1275317e-01 1.1246852e-01 7.9738313e-02 5.3076612e-02 -3.5645048e-03 -7.4779592e-03 -4.0803005e-02 -5.3349229e-02 -4.6262545e-02 -2.3642536e-02 -5.6634779e-02 -4.7546186e-02

8.9333000e+01 -7.0884688e-02 -3.3956270e-02 -2.2216724e-02 -7.5560250e-03 7.6768526e-03 3.6262605e-02 -8.8914027e-03 -1.5198025e-03 -1.4362240e-03 9.1580392e-03 -9.2573040e-03 -1.5765542e-02 -1.5267294e-02 -2.5330178e-02 5.6998594e-03 1.3725587e-01 5.2063758e-01 3.6301006e-01 2.1531401e-01 1.2603452e-01 1.1099198e-01 1.0954546e-01 8.0010363e-02 5.2505095e-02 -3.6656009e-03 -6.0039815e-03 -4.1044634e-02 -5.4317809e-02 -4.8776423e-02 -2.8314391e-02 -6.0666958e-02 -4.9602700e-02

1.0417500e+02 -7.3622342e-02 -3.4588002e-02 -2.4831259e-02 -1.1226433e-02 1.0553161e-02 3.7293545e-02 -9.0972577e-03 -1.3175544e-03 -1.0598244e-04 5.7651566e-03 -9.2637697e-03 -1.5444058e-02 -1.2405334e-02 -2.1382891e-02 5.5936494e-03 1.3780400e-01 5.2364788e-01 3.6486834e-01 2.1916877e-01 1.3005788e-01 1.1441775e-01 1.1027810e-01 8.2331981e-02 5.3180140e-02 -6.5870249e-03 -9.1387776e-03 -4.1872045e-02 -5.6537753e-02 -4.8893490e-02 -3.0797178e-02 -5.8944402e-02 -4.9785412e-02

1.2148300e+02 -6.5104012e-02 -3.2201379e-02 -2.7442873e-02 -1.5708488e-02 6.2261222e-03 3.4900970e-02 -3.0595950e-03 6.5654468e-03 9.6034505e-03 8.3131073e-03 -7.4588888e-03 -1.7642508e-02 -1.6464051e-02 -2.2422149e-02 5.0452356e-03 1.3789881e-01 5.3342362e-01 3.7575803e-01 2.2564211e-01 1.3301989e-01 1.1611810e-01 1.0969513e-01 8.2899362e-02 5.4893196e-02 -3.1179757e-03 -4.1284215e-03 -3.5852742e-02 -5.1313534e-02 -4.4129395e-02 -2.8206251e-02 -5.9806360e-02 -4.9361978e-02

1.4166700e+02 -6.0614371e-02 -2.5001564e-02 -2.5987274e-02 -1.7325825e-02 4.7041308e-03 3.3989276e-02 -9.7048344e-06 9.7405444e-03 1.9015381e-02 1.6874499e-02 5.2912603e-03 -9.0623016e-03 -1.0002152e-02 -1.8906254e-02 1.0056434e-02 1.4395095e-01 5.4233835e-01 3.8686367e-01 2.3704829e-01 1.4335528e-01 1.2653135e-01 1.2088055e-01 9.0324625e-02 6.4035622e-02 5.6469428e-03 5.3573203e-03 -2.2779844e-02 -3.7761145e-02 -2.9988844e-02 -1.4603198e-02 -4.7706464e-02 -3.8303896e-02

1.6520400e+02 -5.3815789e-02 -2.7809328e-02 -2.9865583e-02 -2.2135549e-02 -1.9118837e-03 3.1423089e-02 -1.5582825e-03 1.2916351e-02 2.0734675e-02 1.9888379e-02 1.6031538e-03 -1.4985640e-02 -1.7394638e-02 -2.5810475e-02 8.6462178e-03 1.4325714e-01 5.3907635e-01 3.8244320e-01 2.3276541e-01 1.3585837e-01 1.1796146e-01 1.1428206e-01 8.0544222e-02 5.8026682e-02 1.3721596e-03 3.0930514e-03 -2.7202246e-02 -4.4601664e-02 -3.8216137e-02 -2.2067925e-02 -5.9167622e-02 -4.6707154e-02

1.9265100e+02 -5.8686052e-02 -3.5318755e-02 -3.6930104e-02 -2.5392779e-02 -1.5145664e-04 3.4049856e-02 9.2489045e-05 1.1071108e-02 1.5453380e-02 1.2863979e-02 -4.5617553e-03 -2.0210479e-02 -1.9935179e-02 -2.7439505e-02 9.0262276e-03 1.4260537e-01 5.3218562e-01 3.7459034e-01 2.2347088e-01 1.2753729e-01 1.0971225e-01 1.0755848e-01 7.3862010e-02 5.4202222e-02 -2.1358420e-03 -1.8618013e-03 -3.6285607e-02 -5.6828873e-02 -4.8588546e-02 -3.0635570e-02 -6.6897885e-02 -5.6601764e-02

2.2465800e+02 -6.0178441e-02 -3.7093436e-02 -3.8080276e-02 -2.6953976e-02 -1.0795468e-03 3.5225338e-02 -6.3266049e-04 1.0819123e-02 1.3871356e-02 1.3489043e-02 -5.8861428e-03 -2.3304525e-02 -2.2731758e-02 -2.7757827e-02 9.1469544e-03 1.4431694e-01 5.3309748e-01 3.7336161e-01 2.2015903e-01 1.2452408e-01 1.0541815e-01 1.0453394e-01 7.2560150e-02 5.4056822e-02 -1.5266651e-03 -2.0575995e-03 -3.9618599e-02 -5.7837945e-02 -5.3069585e-02 -3.3525796e-02 -7.1175187e-02 -5.9765364e-02

2.6198300e+02 -6.8239902e-02 -3.7167705e-02 -3.6047827e-02 -2.5681953e-02 1.1895671e-03 3.7438720e-02 8.0816068e-04 8.9218833e-03 1.0541216e-02 9.8048253e-03 -4.2556219e-03 -2.0518787e-02 -1.8954331e-02 -2.3887242e-02 8.7371586e-03 1.4181922e-01 5.3617063e-01 3.7886007e-01 2.1999856e-01 1.2669781e-01 1.1033250e-01 1.0761934e-01 7.7268020e-02 5.7260581e-02 -2.2860373e-04 -3.3468273e-03 -4.1142712e-02 -5.9614545e-02 -5.5416610e-02 -3.3564856e-02 -7.0793231e-02 -5.7226010e-02

3.0551000e+02 -6.2749925e-02 -3.3235300e-02 -3.5649976e-02 -2.7164291e-02 -6.8475786e-03 3.6582829e-02 7.0426924e-04 1.4411084e-02 1.5466282e-02 1.8379212e-02 -6.4997786e-03 -2.2916004e-02 -2.2583775e-02 -2.7341070e-02 1.1662473e-02 1.4946255e-01 5.3951919e-01 3.7860210e-01 2.2154198e-01 1.2413654e-01 1.0870215e-01 1.0922185e-01 7.6979369e-02 5.7841079e-02 7.6736777e-04 -1.5510442e-03 -4.1183433e-02 -5.6421408e-02 -5.6054906e-02 -3.3675531e-02 -6.9677551e-02 -5.1032898e-02

3.5626700e+02 -6.2663786e-02 -3.6303738e-02 -4.0120507e-02 -2.7636427e-02 -8.8351672e-03 3.7726944e-02 4.5562440e-03 1.6688409e-02 1.4625785e-02 2.0735050e-02 -1.2466109e-02 -2.9978342e-02 -2.7026957e-02 -2.9700931e-02 1.3694432e-02 1.5621383e-01 5.2987129e-01 3.5999352e-01 2.0974656e-01 1.1053211e-01 9.8775224e-02 1.0364894e-01 7.3059635e-02 5.7193368e-02 -2.5114409e-04 -1.8705135e-03 -4.8919682e-02 -6.4518924e-02 -6.1147177e-02 -3.6338042e-02 -6.9012454e-02 -4.9666343e-02

4.1545800e+02 -6.4749375e-02 -4.3197097e-02 -4.6357063e-02 -2.7401598e-02 -1.0256685e-02 3.9243356e-02 6.3615459e-03 1.8699573e-02 1.8401463e-02 2.9169575e-02 -1.8823394e-02 -3.9686410e-02 -3.5273105e-02 -3.4138441e-02 1.9322622e-02 1.7328813e-01 5.1399212e-01 3.3162131e-01 1.9257009e-01 9.3111513e-02 7.7964959e-02 9.3105156e-02 6.2288164e-02 5.4309712e-02 -1.2484700e-03 -2.5259807e-03 -5.7371752e-02 -6.9454045e-02 -6.7567121e-02 -3.6455904e-02 -6.7588316e-02 -4.8329944e-02

4.8448300e+02 -7.2414614e-02 -5.3202406e-02 -4.8005230e-02 -2.4748374e-02 -8.6971208e-03 4.3104248e-02 8.9984529e-03 1.7623218e-02 1.9511269e-02 4.1492334e-02 -1.7613855e-02 -4.5804234e-02 -3.3631732e-02 -3.1473280e-02 2.8099863e-02 1.9273544e-01 4.8805453e-01 2.9166620e-01 1.6767022e-01 7.3032990e-02 5.5935938e-02 8.2677816e-02 5.4679050e-02 5.3159064e-02 -8.8737976e-04 -4.9808523e-03 -6.6701588e-02 -7.4937487e-02 -6.9610779e-02 -3.1294694e-02 -6.1033459e-02 -3.9221966e-02

5.6497600e+02 -7.9459581e-02 -6.8758485e-02 -5.0108839e-02 -2.2290371e-02 -1.0707807e-02 5.0049131e-02 1.4272721e-02 2.1101743e-02 2.3003979e-02 5.8461215e-02 -2.0239765e-02 -5.6195838e-02 -3.4832882e-02 -2.9650771e-02 4.1494287e-02 2.1918709e-01 4.5877926e-01 2.4793695e-01 1.3886293e-01 4.9816193e-02 2.9353699e-02 6.9799756e-02 4.2447837e-02 4.9383918e-02 -6.2669029e-04 -9.0775103e-03 -7.5924728e-02 -7.8510195e-02 -7.1736595e-02 -2.5428109e-02 -5.2598418e-02 -2.7264367e-02

Fig. 3a

0 1772.8 1767.2 1761.6 1756 1750.5 1745 1739.6 1734.2 1728.8 1723.5 1718.2 1712.9 1707.7 1702.5 1697.4 1692.3 1687.2 1682.2 1677.2 1672.2 1667.3 1662.4 1657.5 1652.7 1647.9 1643.1 1638.4 1633.7 1629 1624.3 1619.7 1615.2 1610.6 1606.1 1601.6 1597.1 1592.7 1588.3 1583.9 1579.5 1575.2 1570.9 1566.7 1562.4 1558.2 1554 1549.9 1545.7 1541.6 1537.5 1533.5 1529.5 1525.4 1521.5 1517.5 1513.6 1509.7 1505.8 1501.9 1498.1 1494.3 1490.5

1402.1 -3.3221e-05 -0.00012829 -0.00012967 -0.00016675 -0.0002009 -0.00021449 -0.00020164 -0.0002222 -0.00014906 -0.00013268 -0.0001452 -0.00017026 -0.00014878 -4.7889e-05 -0.00012212 -8.1866e-05 -4.1454e-05 -4.3809e-05 -3.5653e-05 -5.4304e-05 -4.8184e-05 -1.1422e-05 -4.0656e-05 -5.9014e-05 -2.3556e-05 -3.1634e-05 -6.4898e-05 -9.5084e-05 -0.00011983 9.1193e-05 6.043e-05 -6.8215e-05 4.832e-06 -5.8418e-05 -9.7174e-05 7.5413e-05 0.00024186 0.00028756 0.00016961 0.00016428 0.00012335 0.00023441 0.00027838 6.3357e-05 0.00012684 0.00041186 0.00030406 0.00032088 0.00027737 0.00028788 0.00034351 0.00013691 -1.243e-05 -0.00013837 0.00015849 -6.7572e-05 1.793e-05 0.00019461 1.8546e-05 7.6009e-05 -0.00028194 -3.0782e-05

1409.6 -2.3905e-05 -7.9699e-05 -0.00010961 -0.00013777 -0.000185 -0.00019517 -0.000196 -0.00023124 -0.00017027 -0.00015306 -0.00016273 -0.00020215 -0.00017912 -7.3923e-05 -0.0001134 -0.00010214 -5.471e-05 -4.4149e-05 -3.6801e-05 -3.9915e-05 -1.6843e-05 8.642e-06 -5.3341e-06 -9.0298e-06 1.1188e-05 2.3149e-06 -1.7891e-05 -7.0793e-05 -4.7831e-05 4.6398e-05 1.2986e-06 -6.5817e-05 -7.9975e-05 -3.7153e-05 -4.7558e-05 5.6968e-05 0.00026466 0.00027264 0.00013058 0.00012099 0.00010956 0.00013402 0.00024036 6.0405e-06 0.00011904 0.00034259 0.00018381 0.00015325 0.00025438 0.00025502 0.00028609 9.7315e-05 1.6525e-05 -8.8726e-05 0.00016515 -3.6111e-05 -1.499e-05 0.00014464 -8.7546e-05 -4.9532e-06 -0.00017774 -1.5681e-05

1417.2 -7.1438e-05 -0.00010294 -0.00015026 -0.00017921 -0.00023497 -0.00025175 -0.00024526 -0.00028219 -0.00025348 -0.00021964 -0.00023236 -0.00027548 -0.00026407 -0.00016957 -0.00014395 -0.00014483 -9.7743e-05 -8.6634e-05 -7.6309e-05 -6.8322e-05 -1.9778e-05 1.0863e-05 1.227e-05 5.3157e-05 3.5377e-05 2.3224e-05 3.5545e-05 -1.6208e-05 8.6715e-06 6.4711e-05 -5.2145e-05 -4.2146e-05 -8.1121e-05 -3.7448e-05 3.42e-05 9.8652e-05 0.00020718 0.00020433 5.3129e-05 6.4688e-05 9.04e-05 7.5759e-05 0.00014565 -6.628e-05 0.00011805 0.00021755 0.00010914 7.8608e-05 0.00021336 0.00020744 0.00027134 6.0898e-05 -1.8213e-05 -4.2301e-05 5.2311e-05 6.3569e-05 -0.00013763 0.00012027 -9.6647e-05 -6.9725e-05 -2.4619e-05 -9.619e-05

1424.8 -9.6685e-05 -0.0001446 -0.00020178 -0.00022589 -0.00028552 -0.00030256 -0.00027089 -0.0003042 -0.00028951 -0.00025268 -0.00028091 -0.00028846 -0.0003286 -0.00022144 -0.00015267 -0.00014437 -0.00012182 -9.872e-05 -9.9118e-05 -5.9395e-05 -3.0915e-05 -2.7618e-06 2.3012e-05 8.6648e-05 6.9473e-05 8.9726e-05 7.2621e-05 0.00010986 0.00013301 0.00016528 0.00011179 3.0311e-06 -5.6774e-05 -8.661e-05 -4.46e-05 -5.648e-06 7.7959e-05 6.2393e-05 -4.1177e-06 5.1157e-05 5.5817e-05 7.709e-05 4.6904e-05 -5.9443e-05 4.6014e-05 0.00013389 8.9704e-05 0.00015096 0.00015837 0.00018569 0.00019792 -4.8286e-05 -9.2015e-05 -0.00011989 -0.00010074 5.0009e-05 -0.00022364 0.00010426 -6.5046e-05 -0.00014164 -6.782e-05 -8.9836e-05

1432.4 -9.0067e-05 -0.00014555 -0.00019494 -0.00021643 -0.00028914 -0.00028756 -0.00025943 -0.00030039 -0.00028721 -0.00026297 -0.0002864 -0.00029121 -0.00033701 -0.00021805 -0.00014071 -0.00013629 -0.00013621 -0.00011108 -9.0057e-05 -5.3895e-05 -2.1233e-05 6.7153e-06 1.7062e-05 0.00012524 0.00013293 0.00012174 0.00010986 0.00016276 7.7024e-05 5.7884e-05 0.00019712 2.9727e-05 1.0403e-05 -0.00017177 -0.00010474 -6.4008e-06 5.8895e-05 4.1293e-05 8.4182e-05 7.9621e-05 1.1597e-05 4.8111e-05 4.029e-05 -4.0226e-05 1.6783e-05 0.00013068 3.7609e-06 0.00020301 0.0001479 0.00017676 0.00011419 -8.9949e-05 -7.8571e-05 -0.00018038 -1.224e-05 -1.233e-05 -0.0001834 0.00010011 -7.1775e-05 -0.00026365 -9.5472e-05 5.1064e-05

1439.9 -6.2465e-05 -0.00012592 -0.00015815 -0.00018568 -0.00025312 -0.00024309 -0.00025988 -0.00029775 -0.00026475 -0.00026696 -0.00028926 -0.00028705 -0.00033032 -0.0002332 -0.00016425 -0.00012472 -0.00013469 -0.00013651 -0.00010238 -6.9227e-05 -2.6521e-05 -8.1926e-06 -1.3374e-05 0.00010073 0.00011903 0.00011292 0.00011406 9.354e-05 3.2323e-05 -2.463e-05 0.00010084 5.2307e-05 -9.2664e-05 -0.00012609 -5.8066e-05 3.6121e-05 0.00010133 6.5577e-05 0.00010465 4.1311e-05 3.8692e-05 3.7594e-05 8.4132e-05 -8.0807e-06 6.5348e-05 0.00014979 -1.7124e-05 0.0001966 0.00011763 6.3086e-05 7.1369e-05 -1.1902e-05 -1.5129e-05 -7.8083e-05 -1.3642e-05 -8.2043e-05 -0.00015589 5.2556e-05 -4.3492e-05 -0.00024848 -0.00012314 -4.5003e-05

1447.4 -6.8068e-05 -0.00013653 -0.00017477 -0.00019597 -0.00026986 -0.00025647 -0.00029733 -0.00032842 -0.00029292 -0.00030598 -0.00033128 -0.00030969 -0.00034809 -0.00028833 -0.00022981 -0.00016067 -0.00014462 -0.00016534 -0.00013315 -7.4334e-05 -5.3748e-05 -3.1652e-05 -1.6441e-05 5.5041e-05 7.5357e-05 7.4002e-05 9.2469e-05 4.0045e-05 -2.0385e-05 2.0573e-05 -5.2224e-05 -1.659e-05 -0.00010207 -7.3977e-05 1.5632e-05 0.00017886 8.0698e-05 7.3042e-05 0.00014428 3.4506e-05 8.87e-05 8.8184e-05 0.00012922 8.2389e-05 9.8195e-05 9.9463e-05 3.6512e-05 0.00016989 0.00011731 7.4735e-05 9.0084e-05 8.4234e-05 0.00016068 5.8699e-05 -5.8278e-05 9.1647e-06 0.00011287 0.00012099 1.7125e-05 -0.00018142 -0.00024479 -0.00022498

1455 -6.6837e-05 -0.00012438 -0.00016982 -0.00019445 -0.00028447 -0.00027483 -0.00028535 -0.00034275 -0.00032756 -0.0003203 -0.0003598 -0.00033652 -0.00032958 -0.00031966 -0.00026673 -0.0001718 -0.00013134 -0.00013276 -0.00012179 -6.1117e-05 -4.0703e-05 -9.0353e-06 3.7229e-05 8.2427e-05 7.1257e-05 9.3873e-05 0.00010082 5.6419e-05 2.3858e-05 1.5114e-05 -5.6138e-05 -1.3694e-05 -6.6168e-05 2.7977e-05 6.7127e-05 0.00016972 5.9302e-05 9.0199e-05 0.00016839 0.00014091 0.00016683 0.00020988 0.00014965 0.00010034 0.00010761 9.0088e-05 0.00012551 0.00017369 0.00021866 0.0002008 0.00017026 0.0001665 0.000135 0.00020606 3.8028e-05 0.0001666 0.00027781 0.000134 5.2671e-05 -0.00021799 -0.000223 -0.0001347

1462.5 -0.000124 -0.00015814 -0.00022751 -0.000256 -0.00035319 -0.00035995 -0.00034651 -0.00043372 -0.00041344 -0.00042134 -0.00045274 -0.00045153 -0.0004093 -0.00043043 -0.00037945 -0.00026391 -0.00019473 -0.00017829 -0.00014275 -0.00010985 -7.9021e-05 -2.3175e-05 1.0554e-05 9.2685e-05 6.3606e-05 9.7813e-05 0.00010635 5.7535e-05 4.3513e-05 7.0605e-05 -7.3088e-05 7.4715e-05 0.00012817 3.2479e-05 1.178e-05 5.6549e-05 -1.9778e-06 8.5253e-05 0.00015669 0.00019683 0.00016704 0.00026524 0.00013358 0.0001105 0.00013787 0.00016729 0.00016083 0.0001566 0.00028562 0.00023735 0.00028987 0.00023665 0.0001293 0.00026653 6.4642e-05 0.00021789 8.258e-05 0.0002085 8.3127e-05 -0.00013962 -6.0476e-05 -5.6865e-05

1470 -4.4077e-05 -0.00012154 -0.00020357 -0.00027022 -0.0003644 -0.00040096 -0.00041697 -0.0005254 -0.00049653 -0.00052467 -0.00056268 -0.00056011 -0.00055535 -0.00054883 -0.00050995 -0.00039979 -0.00029841 -0.00027266 -0.00021675 -0.00018476 -0.00014088 -7.4025e-05 -2.4484e-05 4.6625e-05 7.95e-05 0.00013062 0.00010038 6.1229e-05 7.7198e-05 0.00014038 3.2287e-05 0.00016713 0.00017297 5.1025e-06 -0.00013214 -0.00018275 -8.9144e-05 -5.0073e-06 6.2504e-05 0.00017613 0.00016762 0.00019939 0.00013533 0.00011529 0.000194 0.0002477 0.00019998 0.00016056 0.00038052 0.0003433 0.00041737 0.00030746 0.00028523 0.00040059 0.00027186 0.00014946 0.00026656 0.00019937 0.00013599 -0.00021087 6.828e-05 -0.00011554

1477.5 0.00025474 0.00013561 3.179e-05 -7.5122e-05 -0.00021571 -0.0002547 -0.00034069 -0.00051559 -0.00050382 -0.00053432 -0.00063255 -0.00065262 -0.00069195 -0.00067701 -0.00061962 -0.00054671 -0.000427 -0.00039176 -0.00032872 -0.00029142 -0.00021742 -0.00014202 -7.5254e-05 -3.1313e-05 4.1067e-05 9.4941e-05 9.6678e-05 5.4445e-05 1.358e-05 7.864e-05 8.2478e-06 -7.065e-05 6.4208e-05 -0.00014191 -0.00029589 -0.0002971 -0.00021718 -0.00020045 -5.9732e-05 5.087e-05 0.00010247 0.00010219 9.2023e-05 6.7507e-05 7.2472e-05 0.00023446 0.00018367 0.00022502 0.00047745 0.00041682 0.00051996 0.00042028 0.00041022 0.00055614 0.00044193 0.00024592 0.00068404 0.00030057 0.00017974 -0.00010773 4.5233e-05 -5.0267e-05

1485 0.00040905 0.00033187 0.00022879 0.00012262 -2.1503e-05 -5.2899e-05 -0.00014211 -0.00037711 -0.00038281 -0.00043217 -0.00056882 -0.00061791 -0.00068393 -0.00069069 -0.00064135 -0.00058747 -0.00051943 -0.00044985 -0.00038784 -0.00035153 -0.00027143 -0.00018978 -0.0001054 -4.0354e-05 1.1054e-05 8.4465e-05 0.00012497 0.00011838 0.00010748 1.8093e-05 -1.5345e-05 -0.00020898 -2.8689e-05 -0.00010769 -0.00029304 -0.00038457 -0.00030553 -0.0003201 -0.00022545 -7.7907e-05 -3.314e-05 -4.9725e-05 -0.00010149 -9.4955e-05 -6.5268e-05 0.00012266 6.4637e-05 0.00013044 0.00034775 0.00030844 0.00046505 0.00044519 0.0002836 0.00047557 0.00034841 0.00032477 0.0004554 0.00025321 6.7128e-05 4.8453e-05 -9.4632e-05 -5.3974e-05

1492.5 0.00033105 0.00028871 0.00022563 0.00013353 2.5167e-05 -3.1585e-08 -5.3441e-05 -0.00026875 -0.00028051 -0.00032685 -0.00048142 -0.00053748 -0.00061245 -0.00060167 -0.00058546 -0.00053118 -0.00048763 -0.00044225 -0.00036083 -0.00033082 -0.00026102 -0.00017302 -0.00010634 2.506e-05 9.7129e-05 0.00013252 0.00020842 0.00027078 0.00024006 0.00010417 -2.3434e-06 -7.3092e-05 0.00014573 5.861e-05 -3.366e-05 -0.00026751 -0.00032169 -0.0003858 -0.00024722 -0.0001446 -0.00012886 -0.00027124 -0.00028864 -0.00030756 -3.2879e-06 6.2354e-05 -4.0071e-05 3.8095e-05 0.00014278 0.00033396 0.00044701 0.00044789 0.00020594 0.00032822 0.00032446 0.00025968 -2.8104e-05 6.1959e-05 -3.5631e-05 -1.3186e-05 -0.00015986 -5.9187e-05

1499.9 0.00030336 0.0002421 0.00021532 0.00013863 1.9745e-05 9.7858e-06 -3.6327e-05 -0.00024054 -0.00025712 -0.00029378 -0.00047007 -0.0005436 -0.00062148 -0.00058923 -0.00061568 -0.00057693 -0.00051214 -0.00051175 -0.00045136 -0.00039601 -0.00035386 -0.00026324 -0.00020053 4.4742e-06 0.00010409 0.00015255 0.00020692 0.00034544 0.00037149 0.00018715 0.0001395 0.00010698 0.00013947 0.00011452 5.7485e-05 -0.00019616 -0.00037702 -0.00041527 -0.00034154 -0.00034673 -0.00026619 -0.0004346 -0.00033859 -0.00039929 7.7817e-05 8.0931e-05 7.0443e-05 0.00016943 0.00011995 0.00058255 0.00065127 0.00057211 0.00047195 0.00042177 0.00058069 0.00039833 -6.6413e-05 0.00020802 0.00021444 0.00012132 4.0936e-05 -5.3844e-06

1507.4 0.00041831 0.0003705 0.0003143 0.00026922 0.00012521 0.00010297 5.3935e-05 -0.00019095 -0.00018908 -0.00023274 -0.00044627 -0.0005355 -0.00061651 -0.00060421 -0.00068122 -0.00067242 -0.00059174 -0.00062901 -0.00060493 -0.00054077 -0.00052973 -0.00042275 -0.00036825 -0.00011583 -3.2604e-05 6.9733e-05 0.00015809 0.00038616 0.00046614 0.00046859 0.00040724 0.00031961 0.00031058 8.2735e-05 1.095e-05 -0.00016324 -0.00049786 -0.00057348 -0.0005754 -0.00065336 -0.00052051 -0.00056868 -0.00044155 -0.00048653 0.00012067 0.00026892 0.0002659 0.00038227 0.00040188 0.00092484 0.00084313 0.00083137 0.00092517 0.00085842 0.00089733 0.0006692 0.00050093 0.00050911 0.00054028 0.00031294 0.00034152 0.00016137

1514.8 0.00063789 0.0005846 0.00051358 0.00046412 0.00031111 0.00027857 0.00019943 -0.00011558 -8.1758e-05 -0.00013999 -0.00041848 -0.00053528 -0.00063432 -0.00059215 -0.00078662 -0.00078844 -0.00073043 -0.00083094 -0.00082281 -0.00082027 -0.00078511 -0.00069832 -0.00064203 -0.00041873 -0.00038604 -0.00016303 -7.3949e-05 0.00021379 0.00047156 0.00062804 0.00065995 0.0006186 0.00051028 0.00019323 8.8822e-07 -0.00041046 -0.00073278 -0.00085576 -0.00091806 -0.00089298 -0.00073511 -0.00066387 -0.00059412 -0.00049874 0.00031994 0.00052703 0.00053707 0.00069692 0.00087301 0.0013156 0.0012252 0.0013831 0.0014776 0.0014924 0.0012701 0.0010252 0.00096405 0.00080039 0.00060111 0.0005153 0.00046367 0.00038707

1522.3 0.00082787 0.00078128 0.0007416 0.0006761 0.00051915 0.00048928 0.00038197 -1.2935e-05 5.2558e-05 -1.044e-05 -0.00033872 -0.0005285 -0.00060301 -0.00053565 -0.00085102 -0.00089353 -0.00082758 -0.0011111 -0.0010616 -0.0011457 -0.0011186 -0.0010643 -0.00098201 -0.00090675 -0.00088573 -0.00062749 -0.0005048 -0.00020556 0.00011982 0.00051696 0.00065366 0.00075785 0.00067623 0.00031222 -9.7853e-06 -0.00055218 -0.0009506 -0.0011077 -0.0011294 -0.001119 -0.00089559 -0.00076771 -0.00060416 -0.00042153 0.00040189 0.00079237 0.00086843 0.0010734 0.0014298 0.0019029 0.0019723 0.0021607 0.002091 0.0018723 0.0016062 0.0011021 0.00099363 0.00089744 0.00069021 0.00068686 0.00040036 0.00043574

1529.7 0.00097918 0.00097758 0.00096688 0.00088486 0.00070805 0.00069967 0.00059211 0.00011835 0.00022927 0.00015262 -0.00024647 -0.00044928 -0.00049315 -0.00037461 -0.00086981 -0.00097391 -0.00084157 -0.0013952 -0.0012526 -0.0014025 -0.0014532 -0.0014618 -0.0013323 -0.001587 -0.001514 -0.001257 -0.0011106 -0.00094503 -0.00057636 -8.8326e-05 0.0003005 0.00065467 0.00076262 0.00053729 0.00015568 -0.00047077 -0.00083113 -0.0011196 -0.0012041 -0.0012735 -0.00099662 -0.00076359 -0.00047505 -0.00021846 0.00063686 0.001276 0.0014348 0.0018047 0.0023581 0.002955 0.0029146 0.0028208 0.002375 0.0017857 0.0014416 0.00096443 0.00050162 0.00075214 0.00066751 0.0006903 0.00042407 0.00035302

1537.1 0.0011694 0.001209 0.0012395 0.0011521 0.00096105 0.00099169 0.00090148 0.00036802 0.00052232 0.00042852 -5.8664e-05 -0.00025959 -0.00026455 -7.3211e-05 -0.00074169 -0.00089607 -0.00069041 -0.0015404 -0.0013501 -0.001564 -0.0017238 -0.0017951 -0.0016397 -0.0023266 -0.0022055 -0.0020099 -0.0019125 -0.0019602 -0.0016892 -0.0010357 -0.00038145 0.00037939 0.00089458 0.00073881 0.00051854 -5.8892e-07 -0.00046018 -0.00083345 -0.0010346 -0.0011335 -0.00087695 -0.00041268 5.4324e-05 0.00045596 0.0015484 0.002397 0.0029123 0.0034432 0.0037751 0.0038937 0.0032767 0.0026456 0.0019921 0.0013943 0.0010519 0.00097424 0.00051524 0.00066371 0.00060317 0.00076677 0.00061248 0.00055006

1544.5 0.0013142 0.0013971 0.0014687 0.0014063 0.0012408 0.0013092 0.0012358 0.00068342 0.00084434 0.00076566 0.0002344 -1.8723e-05 2.9601e-05 0.00031407 -0.00049651 -0.00067622 -0.00043285 -0.0015735 -0.0013505 -0.0016286 -0.0019034 -0.00207 -0.0018837 -0.0030877 -0.0029919 -0.0029131 -0.0030472 -0.0034623 -0.00328 -0.0026794 -0.001688 -0.0003293 0.00059377 0.0010525 0.0011371 0.00076909 0.00032722 -0.00014021 -0.00049936 -0.00045466 -3.1271e-05 0.00063205 0.0013759 0.0021344 0.0036974 0.0048173 0.0056067 0.0057937 0.0049631 0.0035809 0.0026528 0.0019645 0.0015654 0.00097148 0.0010422 0.0010293 0.0011836 0.00065077 0.00083719 0.0011046 0.0008006 0.00084158

1551.9 0.001459 0.0015652 0.0016808 0.0016579 0.0015442 0.0016559 0.0016054 0.0010393 0.0012133 0.0011615 0.00062051 0.00031585 0.00043722 0.00083343 -0.00010859 -0.00031521 -9.4125e-06 -0.0014012 -0.0011317 -0.0015035 -0.0018811 -0.0021547 -0.0019658 -0.0038261 -0.003732 -0.0039698 -0.0043814 -0.0054364 -0.0056439 -0.0053468 -0.0041914 -0.002101 -0.00035137 0.0011209 0.001865 0.0019909 0.0015921 0.001202 0.0009631 0.0011721 0.0019935 0.0030305 0.0043123 0.0055894 0.0071167 0.0077799 0.0076245 0.0067021 0.0045854 0.0021891 0.0016841 0.0015423 0.0015183 0.00067037 0.0010423 0.0010897 0.0015082 0.00072379 0.0011688 0.001317 0.00093497 0.0012219

1559.3 0.0015924 0.00172 0.0018655 0.0018997 0.0018333 0.0019915 0.001966 0.0013991 0.0015979 0.0015682 0.0010194 0.00070532 0.00092863 0.0014074 0.00040512 0.0001408 0.00053402 -0.00099914 -0.00070012 -0.0011355 -0.0016059 -0.0019793 -0.0017874 -0.0043176 -0.0043049 -0.0048215 -0.0055634 -0.0074388 -0.0081609 -0.0085509 -0.0074279 -0.0046282 -0.0021139 0.00056131 0.0021766 0.0030659 0.0030496 0.0031814 0.0033344 0.0041509 0.0058728 0.0077301 0.0094692 0.010803 0.0094439 0.0082879 0.0068724 0.0053204 0.0034148 0.0010848 0.0010692 0.0012707 0.001327 0.00054907 0.00089815 0.0012571 0.0012109 0.00062797 0.0014301 0.0015872 0.0011951 0.0015795

1566.7 0.0016427 0.0017884 0.0019587 0.0020147 0.0020053 0.0021963 0.002193 0.0016587 0.0019217 0.0018667 0.001356 0.0010288 0.0013151 0.001901 0.00089706 0.00061589 0.0010635 -0.00057591 -0.00020386 -0.00067899 -0.0012036 -0.0016443 -0.001468 -0.0045658 -0.0046385 -0.0054544 -0.0064534 -0.009229 -0.010671 -0.011769 -0.010956 -0.0078402 -0.0047781 -0.0011572 0.0015541 0.0037784 0.0046534 0.0058613 0.007258 0.0095941 0.012593 0.014722 0.01489 0.014399 0.0082752 0.00605 0.0047317 0.0037619 0.0025938 0.00049781 0.00070065 0.0010698 0.0011132 0.00038994 0.00076787 0.0014587 0.0013725 0.0005097 0.00159 0.0018703 0.0014882 0.001866

1574 0.0017228 0.0018526 0.0020565 0.0021072 0.0021272 0.0023674 0.0024026 0.0019239 0.0022439 0.0021759 0.001645 0.001228 0.001654 0.0024728 0.001431 0.0011668 0.001646 -0.00016577 0.00029309 -0.00018494 -0.00075723 -0.0012539 -0.0010119 -0.0048385 -0.0049605 -0.006143 -0.0075252 -0.011614 -0.01391 -0.016247 -0.016131 -0.013041 -0.0098528 -0.0046324 4.0429e-05 0.004975 0.0083626 0.01251 0.016471 0.020556 0.021668 0.02088 0.016737 0.01435 0.0060193 0.0042522 0.0036212 0.0033241 0.0023383 0.00033941 0.00065422 0.0012128 0.0012901 0.00065653 0.0010383 0.0017625 0.0023017 0.00095766 0.0020947 0.0023761 0.0021264 0.002405

1581.4 0.0018835 0.0019725 0.0021678 0.0022508 0.0022653 0.0025533 0.0026183 0.0022123 0.0026006 0.0025222 0.0018717 0.0013379 0.0019958 0.0031482 0.0021313 0.0018262 0.0023319 0.00032293 0.00088757 0.00040732 -0.00016602 -0.0006474 -0.00029873 -0.0048079 -0.0048825 -0.0066047 -0.0083959 -0.014079 -0.01765 -0.021661 -0.022769 -0.020493 -0.017278 -0.010145 -0.0020708 0.0085757 0.017631 0.026967 0.031831 0.032846 0.025649 0.021281 0.014992 0.012867 0.0047582 0.0034947 0.0034813 0.0033333 0.0023567 0.00035475 0.00077959 0.0014844 0.0015575 0.0011072 0.0016275 0.0020702 0.0027182 0.001539 0.0025516 0.0029077 0.002789 0.0028967

1588.8 0.001993 0.0020677 0.0022157 0.0022942 0.0023187 0.0026054 0.002714 0.0023832 0.0028397 0.0027767 0.0020186 0.0012861 0.0021444 0.0037681 0.0028603 0.0025027 0.0029378 0.00082471 0.0014694 0.0010445 0.00048833 8.7612e-05 0.0006101 -0.0042751 -0.0042491 -0.006344 -0.0084871 -0.015871 -0.020875 -0.027257 -0.03016 -0.029352 -0.026439 -0.016303 -0.001794 0.019232 0.035954 0.04501 0.042513 0.03603 0.022902 0.018434 0.013023 0.011743 0.0043221 0.0032859 0.0036253 0.0035068 0.002648 0.00053507 0.001061 0.0018088 0.0019202 0.0012305 0.0019415 0.0024073 0.0026636 0.0017527 0.002751 0.0034884 0.0031574 0.0031878

1596.1 0.0020368 0.0020649 0.0021989 0.0022346 0.002259 0.0025241 0.0026664 0.0024169 0.0029066 0.0028824 0.0021228 0.0010665 0.0020422 0.0041915 0.0034916 0.0030526 0.0033422 0.0012762 0.0019594 0.0016091 0.0011199 0.00081832 0.0014728 -0.0033367 -0.003099 -0.0053855 -0.0076739 -0.016706 -0.023692 -0.033053 -0.038758 -0.03955 -0.03501 -0.017183 0.0092802 0.03962 0.053126 0.051351 0.041066 0.032012 0.019388 0.016192 0.011893 0.011069 0.0042782 0.0034092 0.0038816 0.003822 0.0031318 0.00091737 0.001358 0.0022102 0.0022411 0.0012898 0.0019125 0.0025883 0.0026623 0.0017955 0.0028343 0.0038316 0.0032418 0.0033648

1603.4 0.0020598 0.0020518 0.0021511 0.0021517 0.0021395 0.0023764 0.0024856 0.0023546 0.0028266 0.0028515 0.0021498 0.00085476 0.0017994 0.0043055 0.0038551 0.0033851 0.0035316 0.0016483 0.002344 0.0020558 0.0016775 0.0014313 0.0021756 -0.0021268 -0.0015826 -0.0038691 -0.0060806 -0.016509 -0.025732 -0.039567 -0.049057 -0.04795 -0.034538 -0.0017702 0.030812 0.052178 0.052699 0.044362 0.034314 0.027047 0.016811 0.014694 0.011182 0.010579 0.0042409 0.0037878 0.0041782 0.0041699 0.0035736 0.0013662 0.0017213 0.0025554 0.0024004 0.0013285 0.0019096 0.0024727 0.0029686 0.001824 0.0026662 0.0034048 0.0030929 0.0032175

1610.7 0.0019553 0.0019353 0.0020077 0.0019868 0.0019208 0.0021164 0.0021653 0.0021077 0.0025723 0.0026146 0.0019719 0.00065895 0.0015109 0.0039496 0.0038061 0.0034345 0.0035212 0.0018801 0.0025268 0.0023339 0.0020221 0.0018895 0.0026693 -0.00085335 -1.1052e-05 -0.0021797 -0.0041907 -0.015637 -0.027885 -0.047412 -0.05889 -0.043609 -0.01455 0.021393 0.040178 0.045563 0.041008 0.03408 0.027127 0.022249 0.014588 0.013223 0.010365 0.010068 0.0043479 0.0040962 0.0044056 0.0044178 0.0037996 0.0017036 0.0020459 0.0027724 0.0023875 0.0014579 0.0019031 0.0021226 0.0028848 0.0014971 0.0023129 0.0027916 0.0026665 0.0027127

1618 0.0017625 0.0017627 0.001831 0.0017856 0.0017042 0.0018533 0.0018566 0.0017903 0.0022294 0.0022793 0.001678 0.0005272 0.0013112 0.0033879 0.0034348 0.0032638 0.0033818 0.0020322 0.0025817 0.0024822 0.0022506 0.0022072 0.0030141 0.00022515 0.0012949 -0.00071473 -0.0028461 -0.016247 -0.033158 -0.055327 -0.056464 -0.020532 0.0085917 0.028391 0.033423 0.03342 0.029804 0.025285 0.020908 0.017944 0.012532 0.011724 0.009464 0.0094178 0.0045325 0.0042026 0.0045028 0.0045247 0.0039029 0.0019088 0.0022616 0.0028149 0.0024459 0.0015009 0.0016384 0.0019494 0.0025007 0.00098736 0.0019127 0.0022513 0.0018471 0.0023197

1625.3 0.0016665 0.0016913 0.001747 0.0016948 0.0016057 0.0017238 0.0017158 0.0015639 0.0019551 0.0019926 0.001428 0.00048518 0.0011892 0.0028959 0.0029922 0.0030574 0.0032738 0.0021719 0.0026658 0.0026221 0.0024949 0.0025039 0.0033015 0.00084912 0.0020057 -0.00042825 -0.0040555 -0.022507 -0.042549 -0.052611 -0.036035 -0.0010278 0.014727 0.023556 0.025306 0.025045 0.022741 0.019857 0.01693 0.015114 0.011218 0.010907 0.0091403 0.0090749 0.0048651 0.004468 0.0047271 0.0047742 0.004095 0.0022251 0.0024599 0.0029369 0.002522 0.001434 0.0016281 0.0018841 0.0021534 0.00057305 0.0015572 0.0018009 0.0012449 0.0018904

1632.6 0.0014948 0.0015349 0.0015555 0.0015346 0.001421 0.0015104 0.0015285 0.0012674 0.0015981 0.0016241 0.0010993 0.00039189 0.00093513 0.0023342 0.002513 0.0027338 0.003075 0.0022001 0.0026769 0.0026873 0.0026449 0.0027055 0.003558 0.00080278 0.0014912 -0.0025903 -0.010271 -0.031143 -0.041794 -0.035143 -0.017391 0.0037176 0.012265 0.018028 0.019517 0.019882 0.018342 0.016325 0.01432 0.013252 0.01032 0.01027 0.0089194 0.0087987 0.0051571 0.0048017 0.0049702 0.0050118 0.0043355 0.0025366 0.0026837 0.0031244 0.0025864 0.0014042 0.0017431 0.0017069 0.0018286 0.00035217 0.0011655 0.0015567 0.0009106 0.0011552

1639.9 0.0012777 0.0012922 0.0013164 0.0012942 0.0012117 0.0012805 0.0012715 0.0010002 0.0012255 0.0012139 0.00079041 0.00028556 0.00061584 0.0016982 0.0020241 0.0023319 0.0027825 0.002123 0.0026219 0.0027054 0.0027146 0.0027997 0.0035872 -0.00021073 -0.0013214 -0.0082275 -0.017432 -0.028372 -0.027813 -0.01955 -0.0094973 0.0035427 0.0091635 0.013427 0.014941 0.015509 0.014463 0.013163 0.011763 0.011183 0.0090668 0.009097 0.0080297 0.007932 0.0050153 0.0047819 0.0048633 0.0048689 0.0042554 0.0026742 0.0026289 0.0030586 0.0026158 0.0013632 0.0016824 0.0015904 0.0017965 0.00026379 0.00084013 0.0012219 0.00061632 0.00067886

1647.2 0.0010979 0.001089 0.0011305 0.0010983 0.0010578 0.0011223 0.0010933 0.00085366 0.00098459 0.00094752 0.000605 0.00023094 0.00035585 0.0011612 0.0016103 0.0020149 0.0024941 0.0020793 0.0025997 0.0027456 0.0027428 0.0026897 0.0029305 -0.0030595 -0.006274 -0.01207 -0.016092 -0.017507 -0.015956 -0.011631 -0.0060707 0.0026138 0.006732 0.0098103 0.010948 0.011645 0.011023 0.010168 0.0093407 0.0089763 0.0074387 0.0074644 0.0066592 0.0066639 0.0044522 0.0043102 0.0044366 0.0044135 0.0038605 0.0025528 0.0023708 0.0028075 0.0025082 0.0013873 0.0015229 0.001579 0.0018644 0.00022548 0.00076018 0.00086038 0.00051368 0.00055082

1654.4 0.00085989 0.00084828 0.00087874 0.00085662 0.00084112 0.00091162 0.00089413 0.0007126 0.00079716 0.00074532 0.00042197 7.3599e-05 4.7613e-05 0.00068425 0.0012172 0.0017168 0.0022048 0.0020538 0.0025872 0.0027367 0.002514 0.0017617 0.00062344 -0.0061127 -0.0082397 -0.0094689 -0.0099426 -0.0099465 -0.0095779 -0.0074955 -0.0042552 0.0018413 0.0048259 0.0070767 0.0079028 0.0084276 0.0081891 0.0076942 0.0070382 0.0068276 0.0056913 0.0057044 0.0051634 0.0052733 0.0036983 0.0035433 0.003756 0.0037414 0.0033189 0.0022412 0.0022074 0.0024946 0.0022942 0.0014116 0.0014012 0.0014937 0.0017311 0.0003204 0.00074356 0.00082785 0.00060483 0.0005138

1661.7 0.00064686 0.00063358 0.00065773 0.00063373 0.00062799 0.00072166 0.00070268 0.00057425 0.0006574 0.00062015 0.00028985 -8.5715e-05 -0.00027654 0.00025446 0.00089738 0.0015206 0.0020266 0.0021375 0.0026226 0.002529 0.0014431 -0.00060243 -0.0022443 -0.0057462 -0.0059047 -0.0054889 -0.0056425 -0.0059865 -0.0062712 -0.0050946 -0.0028578 0.0013738 0.0036186 0.0052353 0.0058858 0.0062836 0.0060923 0.0056323 0.0050784 0.0049312 0.0040783 0.0041108 0.0038345 0.0039359 0.0027755 0.0027385 0.0029835 0.0029305 0.0026927 0.001849 0.001893 0.0020602 0.0018628 0.0012889 0.0012741 0.0013485 0.0016298 0.00049596 0.0006923 0.00081379 0.00059901 0.00042608

1668.9 0.00054191 0.00051341 0.00051749 0.00049192 0.00047779 0.00056169 0.00059083 0.00048739 0.00058708 0.00059897 0.00030425 -0.00016363 -0.00049244 -0.00012586 0.00067628 0.0014219 0.0020908 0.0023932 0.0025929 0.0014406 -0.00075683 -0.0024573 -0.0028393 -0.003615 -0.0034123 -0.0030344 -0.0032207 -0.0038842 -0.0042806 -0.003596 -0.001945 0.00119 0.0028086 0.0040961 0.0047573 0.0049606 0.0046326 0.0041059 0.0036605 0.0034646 0.0029175 0.002918 0.0027882 0.0029007 0.0020235 0.0020868 0.0022268 0.0022162 0.0020417 0.0015204 0.0015078 0.001674 0.0014329 0.0011219 0.0011372 0.0012648 0.0013226 0.0005752 0.00069149 0.00065602 0.00040696 0.00017072

1676.1 0.00040728 0.00035628 0.0003488 0.00031759 0.00027598 0.00035708 0.00041514 0.00033641 0.00049228 0.00053689 0.00032497 -0.0001628 -0.00066966 -0.00044105 0.00041106 0.0013877 0.0023925 0.0025208 0.0015834 -0.00060827 -0.002082 -0.0022783 -0.0020218 -0.0020718 -0.0018649 -0.0016454 -0.001857 -0.0024286 -0.0029148 -0.0025998 -0.0013839 0.00095881 0.0023607 0.0034406 0.0038269 0.0039101 0.0034403 0.0029694 0.0026058 0.0024405 0.001983 0.0020243 0.0018997 0.0019885 0.0015083 0.0015028 0.0015577 0.0016189 0.0015504 0.001172 0.0011924 0.0013215 0.0011581 0.001112 0.00094051 0.0010849 0.0010718 0.00064204 0.00062671 0.00052841 0.00022963 1.1151e-05

1683.4 0.00023334 0.00017878 0.00018049 0.00012235 8.3716e-05 0.00013623 0.00020192 0.00018295 0.00036581 0.00044324 0.00035348 -0.00013145 -0.00080163 -0.00073635 0.00014807 0.0016947 0.0028904 0.001444 -0.00025532 -0.0016539 -0.0018346 -0.001587 -0.0013144 -0.0011226 -0.00097713 -0.00082069 -0.00099884 -0.0014018 -0.0018074 -0.0018875 -0.0010691 0.00098823 0.0020944 0.0028064 0.0028999 0.0027273 0.0023597 0.0020427 0.0016899 0.0015256 0.0011915 0.00118 0.0010196 0.0011044 0.0009539 0.00090995 0.0009988 0.0010001 0.0010925 0.00080653 0.00085843 0.00093232 0.00083446 0.00089107 0.00067305 0.00075459 0.00091749 0.0004073 0.00034878 0.00034179 0.00010737 -3.0497e-05

1690.6 0.00011685 7.4416e-05 8.6389e-05 1.0552e-05 -2.7246e-05 8.3555e-06 7.2828e-05 0.0001297 0.0002979 0.00041791 0.00044682 7.32e-06 -0.00083464 -0.00096404 0.00038624 0.0024488 0.0025564 -0.00016367 -0.0010327 -0.0013923 -0.0012688 -0.0010506 -0.00082223 -0.0004695 -0.00035921 -0.00027654 -0.00042989 -0.00062118 -0.0010476 -0.0012667 -0.00063602 0.00096463 0.0018673 0.0021312 0.0020724 0.0018827 0.0015543 0.0012581 0.00095631 0.00075365 0.00058335 0.00048159 0.00042326 0.00047633 0.00036277 0.0004704 0.00049136 0.00051237 0.00055663 0.00046987 0.00047522 0.00050662 0.00045885 0.00049254 0.00037947 0.00032266 0.00038865 2.4858e-05 -2.9219e-05 8.8183e-05 -0.00017223 -0.00011195

1697.8 2.8385e-05 5.1652e-06 1.7588e-05 -4.5849e-05 -0.00010893 -6.8185e-05 -1.3793e-05 7.2772e-05 0.00022974 0.00039132 0.0005649 0.00013363 -0.0011126 -0.0011259 0.0012839 0.0023083 0.0011814 -0.00077672 -0.00096486 -0.0010434 -0.00090978 -0.00071737 -0.00055568 -9.9577e-05 -5.7789e-05 3.9009e-05 -6.2409e-05 -0.00029233 -0.00053727 -0.00068578 -0.00031533 0.00076929 0.0013383 0.0015603 0.001574 0.0014287 0.0011298 0.00080552 0.00053502 0.00036684 0.00029832 0.00018685 0.00017624 0.00012601 7.8345e-05 0.00023798 0.0001901 0.00021982 0.00028274 0.00027234 0.0001894 0.00017884 0.00019066 0.00019735 0.00014283 5.8222e-05 -7.7422e-05 -7.24e-05 -8.4104e-05 4.5215e-05 -0.00016099 -0.00017796

1704.9 -1.0159e-05 -2.8995e-05 -4.3853e-05 -7.0133e-05 -0.00015392 -0.00014603 -0.00010779 -4.1322e-05 0.00013814 0.00035907 0.00075419 2.1881e-05 -0.0015759 -0.00038689 0.0014543 0.0011182 0.00020544 -0.00078738 -0.00081495 -0.00081294 -0.00066227 -0.00046184 -0.00038701 6.3528e-05 9.3081e-05 0.00014842 8.0941e-05 -6.2935e-05 -0.00030021 -0.00023758 -0.00018052 0.00041946 0.00094173 0.0012435 0.0013219 0.0012301 0.00099273 0.00060252 0.00041871 0.00026427 0.00020784 0.00015295 8.7921e-05 -3.6053e-05 0.00011374 0.0001688 0.00010306 5.2799e-06 6.5108e-05 0.00017189 0.00012519 7.8514e-05 6.6888e-05 0.00011016 6.568e-05 -1.1323e-05 -0.00019399 -6.3204e-05 2.0361e-05 1.4027e-06 -6.1244e-05 -0.00013233

1712.1 4.5115e-05 1.5501e-05 -3.5565e-05 -4.66e-05 -0.00013736 -0.00014163 -0.00012518 -6.8241e-05 0.00015704 0.00053969 0.00085841 -0.00060282 -0.0011242 0.00051735 0.00059575 0.00026391 -9.9672e-05 -0.00054505 -0.0005007 -0.00049168 -0.00034041 -0.00019916 -0.00016669 0.0001469 0.00020129 0.00020554 0.0001617 7.7501e-05 -0.00015159 -0.00015922 -0.00023942 0.00030357 0.00075478 0.0011102 0.0013314 0.0012132 0.0010311 0.00074517 0.00053753 0.00036499 0.00029775 0.00020981 0.0001253 4.7886e-05 0.00023354 0.00022794 0.00015929 -4.8009e-05 4.7676e-05 0.00011732 0.00017323 0.00011191 8.2394e-05 7.9774e-05 1.3683e-05 -8.7755e-05 -5.6562e-05 -4.0074e-05 6.3111e-05 -8.9963e-05 -8.0739e-05 -0.00023301

1719.3 7.8854e-05 5.9343e-05 -1.0995e-05 -4.5362e-05 -0.00010972 -0.00011997 -9.3355e-05 -2.1645e-06 0.00032795 0.00077979 0.00029171 -0.00097903 -0.00036171 0.00030888 -2.6378e-05 -4.269e-05 -7.2047e-05 -0.00028096 -0.00025214 -0.0002224 -0.00012769 -6.0903e-05 -1.9689e-06 0.00010717 0.00015968 0.00016441 0.00011793 8.0733e-05 -0.00018066 -0.0003607 -0.00031861 0.0001209 0.00057697 0.00093865 0.0012113 0.0012923 0.0011884 0.00094411 0.0007054 0.00046533 0.00039053 0.00026233 0.00017571 0.00020131 0.00033249 0.00025621 0.00021189 4.9394e-05 0.00018376 0.00021594 0.00013889 0.00017701 0.0001361 3.0132e-05 -3.8379e-05 -8.1526e-05 -0.00031176 -1.0345e-05 8.5478e-05 -0.00017112 -3.2636e-05 -0.00012448

1726.4 0.00015164 0.00012817 5.6775e-05 -6.3078e-06 -4.3051e-05 -5.9498e-05 -2.0806e-06 0.00017556 0.00056411 0.00058508 -0.00026426 -0.00062293 -0.00021349 -5.3957e-05 -0.00019076 -0.00011338 -2.9798e-05 -0.00014594 -0.00013997 -8.0799e-05 -1.8979e-05 -3.2831e-05 5.2268e-05 3.609e-05 0.00012526 8.351e-05 7.9941e-05 -7.7879e-06 -0.00016334 -0.00026819 -0.00021817 1.0483e-05 0.00034178 0.00064825 0.0010392 0.0011753 0.0011344 0.00093896 0.00069789 0.00043268 0.00036006 0.00037996 0.00021506 0.00028016 0.00039094 0.00028695 0.00027913 0.00012374 0.00028462 0.00029379 0.00017235 0.00016266 0.00023951 0.00016416 3.4476e-05 -3.8237e-05 -0.00051483 0.00011975 3.4063e-05 -0.00017689 2.3716e-05 -7.5769e-05

1733.6 0.00026644 0.00022075 0.00014666 8.245e-05 2.8086e-05 3.202e-05 0.00015833 0.00039319 0.0005341 0.00020476 -0.00025978 -0.00034556 -0.00022919 -0.00018843 -0.00020443 -0.00014676 -3.4736e-05 -9.5961e-05 -6.02e-05 -1.3228e-05 2.863e-05 -1.1371e-05 5.8038e-05 4.6449e-06 9.8977e-05 1.5187e-05 1.1848e-05 -3.9307e-05 -0.00017245 -0.00011954 3.4666e-05 -5.9953e-05 0.0002784 0.0005554 0.00092646 0.0010691 0.00098834 0.00081481 0.00062311 0.00045133 0.00030114 0.00040969 0.00030731 0.00036423 0.00045002 0.00033305 0.00034904 0.00027921 0.0002565 0.00027227 0.0003812 0.00021167 0.00033288 0.0002803 0.00020101 4.627e-05 -0.00020283 0.00035415 3.259e-05 -4.3481e-05 4.2749e-05 -0.00022384

1740.7 0.00029882 0.00022676 0.00014433 6.1434e-05 9.5112e-06 9.462e-05 0.00030018 0.00033636 0.00026489 6.4119e-05 -0.0001924 -0.00026795 -0.00026776 -0.00025921 -0.00023829 -0.00018544 -8.5812e-05 -9.7423e-05 -3.2836e-05 -3.6319e-05 -5.7882e-06 -3.6121e-05 -7.1689e-06 -4.6564e-05 -4.7129e-05 -6.9083e-05 -7.6968e-05 -0.00014438 -0.00016636 -0.00013055 2.4417e-05 -6.7859e-05 0.00012422 0.00038224 0.00073211 0.0007897 0.00073814 0.00068207 0.00058518 0.00042503 0.00031025 0.00035583 0.00036367 0.00039464 0.00051298 0.0003465 0.00035829 0.0004297 0.00036977 0.00029831 0.00045521 0.00030608 0.00028117 0.00030633 0.00039079 0.00016172 4.8551e-05 0.00032952 1.2602e-05 0.00012528 -2.3011e-05 -0.00014647

1747.8 0.00030099 0.00021189 0.00012821 4.0358e-05 6.3834e-05 0.00023553 0.00034324 0.00022519 0.0001344 6.0042e-05 -0.00013787 -0.00021994 -0.00025389 -0.00026337 -0.00023238 -0.00017065 -9.1627e-05 -7.3659e-05 -1.7888e-05 -3.157e-05 -3.8911e-05 -3.2502e-05 -2.4633e-05 -6.1132e-05 -6.6282e-05 -7.9093e-05 -9.0264e-05 -0.00013467 -0.00014295 -0.00011279 -2.3258e-05 -9.463e-05 2.4941e-05 0.00015097 0.00034764 0.00048606 0.00055615 0.00051689 0.00048251 0.00029701 0.00026656 0.00024994 0.00033762 0.00038496 0.00044758 0.00030868 0.00031435 0.00042533 0.00049089 0.00037521 0.00043227 0.00028958 0.0003553 0.00028378 0.00041711 0.00023617 0.00023879 0.00040716 0.00015005 0.00020385 0.00010119 4.9196e-06

1755 0.0002525 0.00017465 8.8039e-05 4.8993e-05 0.00017755 0.00028993 0.0002692 0.00016881 0.00010425 7.2417e-05 -0.00010118 -0.00017504 -0.00021698 -0.00021356 -0.00022099 -0.00015088 -7.1814e-05 -3.8252e-05 -1.4972e-05 -1.6452e-05 -2.156e-05 -3.1332e-05 -5.0653e-06 -4.0459e-05 -1.2433e-05 -7.7631e-06 1.7125e-06 -6.9738e-05 -4.9002e-05 2.1475e-06 -2.8175e-05 1.3311e-05 -4.8604e-05 -9.2764e-05 -7.6003e-06 0.0002126 0.00038863 0.00036978 0.00025713 0.00020041 0.00023069 0.00024204 0.00024081 0.00032028 0.00029675 0.00027069 0.00032642 0.00033333 0.00040988 0.00032036 0.00049956 0.00029354 0.00044869 0.0003106 0.00035222 0.00031971 0.00031963 0.0004777 0.00026126 9.1363e-05 0.00022674 -8.4548e-05

1762.1 0.00017311 9.7411e-05 5.4223e-05 8.8187e-05 0.0002029 0.00022811 0.00017937 0.00010947 9.4176e-05 5.5753e-05 -7.6372e-05 -0.00016279 -0.00018273 -0.0002029 -0.00020761 -0.00014066 -5.6754e-05 -2.853e-07 -7.9593e-07 3.0006e-06 -1.2334e-05 -1.5118e-05 2.7939e-06 -4.9441e-05 1.0045e-05 1.9513e-05 2.403e-05 -2.3422e-05 -9.6543e-05 -1.3986e-05 1.3234e-05 -2.9713e-05 -8.8739e-05 -0.00022754 -0.00011415 4.6254e-05 0.00027226 0.00021814 0.00010915 0.00015073 0.00012621 0.00015458 0.00014818 0.00018288 0.00018271 0.0002061 0.00026504 0.00020496 0.00014885 0.00018138 0.00034774 0.00032998 0.00037541 0.00037759 0.00033656 0.00028214 0.0003357 0.00028043 0.00010754 -6.7459e-05 0.00025375 -5.9684e-05

1769.2 0.00011595 6.5809e-05 9.2261e-05 0.0001583 0.00018874 0.00018979 0.00014203 8.964e-05 8.9371e-05 4.2192e-05 -6.2768e-05 -0.00013671 -0.00016121 -0.00015935 -0.00016283 -8.2956e-05 -1.8146e-05 4.5638e-05 3.8898e-05 1.7427e-05 -6.9706e-07 4.7014e-06 2.8748e-05 -1.0193e-05 -6.0799e-06 -5.5221e-06 2.4464e-05 -8.1255e-05 -0.00014165 -0.00012322 -0.00010218 -0.00014232 -0.00013415 -0.00028212 -6.5161e-05 -5.7754e-06 0.00018646 0.0001588 0.00013812 0.00012227 8.3889e-05 9.5924e-05 0.00012708 7.9983e-05 0.00018124 0.00011666 0.00016083 0.00010107 5.5826e-05 9.7665e-05 0.00019043 0.00030887 0.00019933 0.00027157 0.00030769 0.00022559 0.00019246 1.6295e-05 -1.3898e-05 8.3158e-05 0.00019497 7.7214e-05

1776.3 0.00010465 0.00010635 0.00016506 0.00020166 0.00018583 0.00019179 0.00015946 0.00011062 0.00011544 6.3705e-05 -2.6167e-05 -8.2098e-05 -0.00010142 -8.6205e-05 -7.3345e-05 4.1981e-06 6.6611e-05 9.1827e-05 9.8196e-05 7.2805e-05 4.6695e-05 5.4187e-05 8.5339e-05 4.7739e-05 3.7922e-05 -1.3628e-05 -2.4778e-05 -8.2084e-05 -0.00015144 -0.00023575 -0.00020587 -0.00023057 -4.1154e-05 -0.00015392 -5.7902e-05 5.1065e-05 0.00018955 0.00022197 0.00027527 0.00025008 0.00015674 0.00017753 0.00020404 9.4146e-05 0.00017028 4.2633e-05 7.5421e-05 6.1636e-05 5.5643e-05 4.7692e-05 0.00015319 0.00016033 0.00012405 9.3715e-05 0.00015116 0.0002166 2.5891e-05 1.0998e-05 2.4468e-05 0.00026728 0.00012001 0.00019068

1783.3 0.00016517 0.00020718 0.0002447 0.00026184 0.00022931 0.00023996 0.00022822 0.00018556 0.0001759 0.00014308 3.7377e-05 2.4339e-05 2.913e-05 3.8618e-05 3.192e-05 0.00010791 0.0001593 0.00016526 0.00017489 0.00015031 0.00013986 0.00014566 0.00016091 0.00013562 0.00011637 3.581e-05 4.7971e-05 -3.7974e-05 -2.6526e-05 -0.00015945 -0.00016567 -0.00013943 2.9296e-05 -1.1254e-06 1.3201e-05 8.7383e-05 0.00019263 0.00026888 0.00036679 0.00029892 0.00020421 0.00023721 0.00023844 0.0001498 0.00014087 2.5919e-05 1.0409e-05 6.2325e-06 -8.04e-05 4.4883e-05 0.00011476 -1.7069e-05 0.0001202 6.1422e-05 -3.0083e-05 0.00013881 0.00015215 3.0489e-05 6.8984e-05 7.0152e-05 0.00013206 0.00012403

1790.4 0.00015957 0.00019677 0.00021344 0.00021591 0.00020042 0.00020277 0.00018203 0.00014845 0.00012754 0.0001131 2.4292e-05 1.8487e-05 3.8524e-05 3.3958e-05 1.8903e-05 7.064e-05 0.0001153 0.00012878 0.00013723 0.00014019 0.00012125 0.00015075 0.00015132 8.6208e-05 6.976e-05 4.6158e-05 4.5369e-05 4.1457e-05 9.152e-06 -5.5746e-05 5.1938e-05 -1.8835e-05 3.6454e-05 2.2663e-05 7.4967e-05 0.00011996 0.00016656 0.00017924 0.00029681 0.00020586 0.00020145 0.00021269 0.00017982 9.1374e-05 0.00012267 6.8051e-05 1.2694e-05 -7.3185e-05 -0.00022117 7.5034e-05 1.4784e-05 3.4935e-05 0.00010531 0.00011486 -1.9119e-05 2.5336e-05 0.00024547 -5.1966e-05 8.2871e-05 -9.0658e-05 0.0001582 -0.00013923

1797.4 0.00011031 0.00013306 0.00015105 0.00014296 0.0001437 0.00014403 0.00011211 7.5664e-05 5.5771e-05 6.1985e-05 -1.559e-05 -1.5542e-05 -2.4361e-05 -1.2032e-05 -2.775e-05 4.8775e-06 3.5205e-05 5.817e-05 7.5037e-05 8.7768e-05 7.8631e-05 9.6577e-05 7.318e-05 -4.4376e-06 -1.8269e-05 1.809e-05 2.7014e-05 4.4081e-05 2.9978e-05 9.4097e-05 0.00019485 0.00015167 1.1516e-05 -1.8722e-06 0.00012976 0.00015139 0.00010527 8.3177e-05 0.00014901 8.6803e-05 0.00020433 0.00014271 0.00011659 4.5907e-05 0.00011585 0.00013043 3.514e-05 -5.4323e-06 -8.6447e-05 0.00012108 -1.5328e-05 7.4291e-05 9.4319e-05 3.771e-05 -1.6125e-05 5.5899e-05 2.6779e-05 -0.00022163 7.0922e-06 -8.077e-05 9.2255e-05 -0.00028559

1804.5 8.8551e-05 9.7375e-05 0.00011955 0.00011819 0.00010008 0.00011002 9.3596e-05 6.4918e-05 4.6402e-05 5.6758e-05 -1.79e-05 -2.2806e-05 -4.9244e-05 -4.6647e-05 -4.0382e-05 -1.1319e-05 1.1564e-05 3.9153e-05 4.6487e-05 5.4924e-05 6.0405e-05 6.812e-05 4.2006e-05 -1.5558e-05 -1.0126e-05 4.7488e-06 1.2182e-05 8.217e-05 7.7309e-05 0.00013162 0.00019045 8.8645e-05 6.2407e-05 0.00012865 0.0001263 0.00014305 6.7613e-05 -5.8642e-05 2.6088e-05 4.1361e-05 0.00015255 2.1502e-06 2.8262e-05 0.00013551 0.00013142 0.00016662 7.9657e-05 0.00011974 0.00017496 0.00011878 7.5524e-05 -6.0331e-05 3.7011e-05 -0.00014094 5.685e-05 0.00015415 -0.00026859 -0.00025329 -0.00011509 -3.5414e-05 -2.4441e-05 -0.00014812

1811.5 0.00011908 0.00013398 0.00014619 0.00014354 0.00010277 0.00011697 0.00011097 0.00010509 7.2738e-05 7.0753e-05 1.1756e-05 3.0728e-06 -9.2191e-06 -1.0803e-06 -1.8693e-05 9.3763e-06 3.9808e-05 5.8534e-05 6.3832e-05 6.769e-05 6.8325e-05 5.2904e-05 4.1838e-05 3.2509e-05 2.634e-05 1.0521e-05 4.564e-06 8.112e-05 0.00011409 0.00010877 5.0238e-05 4.9503e-06 3.2554e-05 0.00019 0.00021296 8.2319e-05 -7.8731e-06 -5.5764e-05 -9.9373e-06 2.7974e-05 0.00012803 4.0551e-05 3.6696e-05 0.00015814 0.00025174 0.00016509 0.00011379 0.00025905 0.00017373 0.00019767 0.00013994 -1.4623e-05 -1.5334e-05 -4.6974e-05 0.00026367 0.0001765 -0.00016052 1.5919e-05 -7.4858e-06 1.2408e-05 2.0339e-05 0.00019825

1818.6 0.00017717 0.00015565 0.00016037 0.00017136 0.0001358 0.00012499 0.00014463 0.00010823 8.4212e-05 7.4337e-05 5.2392e-05 2.6769e-05 3.6361e-05 5.8239e-05 2.094e-05 3.7471e-05 5.77e-05 7.1675e-05 7.362e-05 7.5419e-05 6.8397e-05 6.3425e-05 4.7198e-05 5.188e-05 8.8975e-05 6.4256e-05 5.4032e-05 8.6437e-05 0.00012802 4.9172e-05 9.881e-05 5.8914e-05 4.6925e-05 0.0002027 0.00022161 2.0353e-05 -5.0896e-05 -9.4763e-05 -0.00011118 -3.1212e-05 6.6159e-05 6.5651e-05 8.5074e-05 3.307e-05 0.00022994 0.00015191 0.00013035 0.00030557 7.9704e-05 0.00029246 0.00012084 0.00017718 0.00010274 0.00011148 0.00031361 0.00018038 0.00038831 0.00013432 7.9074e-05 5.3293e-05 0.00012962 0.00026611

1825.6 0.00012698 9.1953e-05 0.00010544 0.00012419 0.00010949 8.194e-05 0.00011846 5.9385e-05 3.9767e-05 2.7448e-05 3.0085e-05 1.8302e-05 1.4856e-06 2.0674e-05 -3.1543e-05 5.5459e-06 1.5682e-05 2.9547e-05 2.6478e-05 2.8489e-05 3.7518e-05 4.4467e-05 1.4527e-05 2.5742e-05 8.7803e-05 0.00011666 0.00012242 4.4597e-05 0.00012061 8.2614e-05 0.00029694 0.00024369 0.00012097 0.00017799 0.0001418 -8.0012e-05 -0.0001424 -0.00016975 -0.00022386 -0.0001346 -1.7298e-05 3.8256e-05 0.00016234 2.1909e-05 0.00017541 0.00019236 0.0001823 0.00029548 8.842e-05 0.00024048 8.5731e-05 0.00026554 0.00029571 0.00021851 0.00016419 0.00012624 0.00048704 8.072e-05 7.7784e-05 6.5155e-05 0.00013186 2.0534e-05

1832.6 4.6612e-05 6.2713e-05 5.97e-05 6.5288e-05 6.5097e-05 3.5488e-05 5.8136e-05 4.8644e-06 -1.0358e-05 -2.6517e-05 -7.3168e-07 -3.9894e-05 -2.4891e-05 -4.3228e-05 -9.4421e-05 -5.1092e-05 -2.2364e-05 -1.9134e-05 -1.0848e-05 -1.564e-05 -6.5612e-06 8.9648e-06 -1.5582e-05 -1.4249e-06 0.00010249 0.0001244 9.661e-05 6.5597e-05 6.3033e-05 6.6572e-05 0.00035068 0.00022158 9.1506e-05 4.7818e-05 6.4411e-05 -0.00010662 -0.00018284 -0.00020572 -0.00026969 -0.00014083 -4.0847e-05 1.4014e-05 0.00014492 8.3368e-05 0.00016968 0.00016494 0.00015223 0.00024386 0.00013658 8.9175e-05 -2.2013e-05 0.00014761 0.00023746 0.00018722 2.4334e-05 3.5444e-05 0.0001811 -1.0316e-05 0.00011383 4.1089e-05 4.2087e-05 -0.00021541

1839.6 6.0517e-05 6.8308e-05 5.2044e-05 4.8476e-05 4.3811e-05 3.8791e-05 2.5233e-05 -2.3998e-05 -3.23e-05 -3.056e-05 -2.5797e-05 -5.8383e-05 -4.8491e-05 -4.6172e-05 -7.5693e-05 -4.269e-05 -2.8983e-05 -3.3332e-05 -1.3515e-05 -3.493e-05 -8.112e-06 -4.0497e-06 -4.143e-06 1.2245e-05 0.0001102 8.0819e-05 7.0848e-05 7.327e-05 7.887e-05 0.00011584 0.00024101 0.00021246 7.8656e-05 4.1007e-05 0.00010073 -0.00019498 -0.0002747 -0.00024286 -0.00026093 -0.0001163 -7.628e-05 -2.9858e-05 9.3763e-05 7.9636e-05 0.00020439 0.00015327 9.8422e-05 0.00019155 0.00019672 3.1131e-05 -6.9377e-05 -4.6712e-05 0.00013369 0.00015542 1.9196e-05 1.5539e-05 0.0002307 7.2014e-05 0.00017175 5.7616e-05 2.7261e-05 -6.193e-05

1846.6 4.7948e-05 3.6934e-05 3.5454e-05 2.5646e-05 1.7152e-05 1.3054e-05 -4.4054e-06 -4.9846e-05 -5.7694e-05 -4.8236e-05 -4.4055e-05 -8.1375e-05 -6.7424e-05 -6.4711e-05 -6.3019e-05 -4.6745e-05 -4.6146e-05 -4.7222e-05 -2.3008e-05 -3.4839e-05 -9.565e-06 1.2878e-07 5.1413e-06 4.3328e-06 8.5978e-05 3.359e-05 2.4153e-05 9.7361e-05 0.0001089 0.00021927 0.00016845 0.00020192 0.00019905 0.00015888 -3.7911e-05 -0.00028941 -0.00033562 -0.00032254 -0.00024324 -0.00015136 -0.00011508 -0.00013174 2.9214e-05 0.00011322 0.0001989 0.00011734 0.00010341 0.00015066 0.00019829 0.00010424 -1.1702e-05 -1.8542e-05 9.5092e-05 0.00011399 -4.2838e-05 2.3564e-05 0.00030544 4.3288e-05 0.00016878 0.00017611 2.8299e-05 -4.2572e-05

1853.5 2.779e-05 3.9799e-05 4.2443e-05 3.2308e-05 9.179e-06 5.7614e-06 -1.2663e-05 -5.4085e-05 -6.2137e-05 -5.6666e-05 -4.8676e-05 -8.5006e-05 -4.9538e-05 -6.4717e-05 -9.4311e-05 -7.3226e-05 -6.9384e-05 -4.9303e-05 -3.6017e-05 -3.8388e-05 -2.0159e-05 -1.3478e-05 1.2628e-06 7.6948e-08 1.6984e-05 1.7991e-05 3.43e-05 0.00010504 0.00017202 0.00021838 0.0001284 0.00020369 0.0002783 7.5683e-05 -0.00015503 -0.00028709 -0.00032235 -0.00028683 -0.0002274 -0.00021269 -7.6951e-05 -0.00013711 -1.5333e-06 0.00016303 0.00014029 0.00018064 0.00014369 0.00016876 0.00013601 0.00014549 0.00014951 0.0001277 0.00011599 0.00015507 -0.00011754 1.469e-05 0.00025992 -3.478e-05 8.9656e-05 0.00013673 4.2838e-06 -8.3537e-05

1860.5 3.3131e-05 4.8172e-05 5.5886e-05 5.8957e-05 2.1487e-05 4.5844e-05 3.9329e-06 -3.8845e-05 -4.1906e-05 -3.3091e-05 -1.0655e-05 -4.3193e-05 -4.4374e-06 -1.5884e-05 -9.7115e-05 -6.5018e-05 -6.9595e-05 -3.374e-05 -2.0707e-05 7.4156e-06 1.163e-05 3.7946e-06 1.8502e-05 -8.5666e-06 -7.2693e-06 -4.812e-06 4.122e-05 6.6561e-05 4.8162e-05 3.4938e-05 -1.1667e-05 7.0443e-05 0.00018594 3.2025e-05 -8.0218e-05 -2.7947e-05 -7.6514e-05 -1.0262e-05 -8.1518e-05 -5.1336e-05 -6.4539e-05 -6.8926e-05 8.3063e-05 0.00022849 0.00012809 0.0002732 0.00021609 0.00026129 6.1999e-07 0.0001539 0.0002342 0.00016283 0.00024462 0.0001985 1.7959e-05 3.0725e-05 0.00039829 -4.0133e-05 7.1198e-05 7.5069e-05 4.5051e-05 4.1493e-05

Fig 3b

0 1772.8 1767.2 1761.6 1756 1750.5 1745 1739.6 1734.2 1728.8 1723.5 1718.2 1712.9 1707.7 1702.5 1697.4 1692.3 1687.2 1682.2 1677.2 1672.2 1667.3 1662.4 1657.5 1652.7 1647.9 1643.1 1638.4 1633.7 1629 1624.3 1619.7 1615.2 1610.6 1606.1 1601.6 1597.1 1592.7 1588.3 1583.9 1579.5 1575.2 1570.9 1566.7 1562.4 1558.2 1554 1549.9 1545.7 1541.6 1537.5 1533.5 1529.5 1525.4 1521.5 1517.5 1513.6 1509.7 1505.8 1501.9 1498.1 1494.3 1490.5

1402.1 -0.00010483 -9.1546e-05 -5.1956e-05 -9.3901e-07 9.0091e-06 3.6301e-05 5.123e-05 5.1543e-05 2.3552e-05 5.5154e-05 6.274e-06 -4.5294e-05 1.0054e-05 1.1115e-05 7.6555e-05 9.9906e-05 0.00010633 0.00012603 0.00012783 0.00010955 8.6678e-05 4.2286e-05 3.4074e-05 3.8574e-05 9.4036e-06 -4.307e-05 -3.8479e-05 -4.5177e-05 4.9449e-05 0.00015225 4.3072e-05 -7.3894e-05 -1.4158e-05 -0.00024516 -0.0002876 -0.00028036 -0.00028522 -0.00023982 -0.00023913 -0.00011317 -0.00017488 -0.00021128 -0.00024785 -0.00015901 5.709e-05 -0.00012064 -0.00031446 -0.00017993 -0.0001594 -0.00045329 -4.3644e-05 -0.00028485 -0.00022412 -0.00035143 -0.00020732 -5.8886e-05 -0.00021608 -0.00034749 5.6387e-05 1.5646e-05 2.5759e-05 -3.7199e-05

1409.6 -1.8187e-05 -1.0755e-05 2.327e-05 5.3079e-05 7.0142e-05 8.107e-05 0.00010153 0.00010396 8.6632e-05 0.00010438 4.9571e-05 -2.1468e-06 2.7687e-05 6.4657e-05 0.00010581 0.00013907 0.00015589 0.00014121 0.00015479 0.00013236 9.4872e-05 7.5732e-05 7.0156e-05 5.3695e-05 4.2196e-05 5.2828e-06 -1.259e-05 -2.0353e-05 4.958e-05 0.00011186 3.2696e-05 -0.00012208 -7.5897e-05 -0.00021922 -0.00021117 -0.00024298 -0.00023266 -0.00020033 -0.00016826 -0.00015357 -0.00016496 -0.0002109 -0.00024219 -0.00016511 -2.9989e-05 -0.0001497 -0.00027442 -0.00017683 -0.00035336 -0.00035542 -9.3297e-05 -0.00021427 -0.00017197 -0.0002889 -0.00022375 -9.7642e-05 -0.00013585 -0.00036227 -1.6824e-05 3.2012e-06 -9.7274e-06 -7.3266e-05

1417.2 5.8978e-06 2.582e-05 4.5162e-05 2.9194e-05 6.7995e-05 6.9847e-05 8.4051e-05 8.1467e-05 8.0458e-05 7.9534e-05 8.7034e-06 -1.3716e-05 -4.0122e-05 2.0603e-05 8.8815e-05 0.00014139 0.00013471 0.00011506 0.00012397 0.00010936 6.9716e-05 5.5889e-05 8.1879e-05 4.6415e-05 2.8467e-05 1.8201e-05 4.4701e-06 -1.8403e-05 1.085e-05 0.0001038 1.3219e-05 -0.00013964 -0.00010155 -0.00022704 -0.00014153 -0.00016807 -9.6594e-05 -9.7239e-05 -6.2275e-05 -0.00010963 -0.00011932 -0.00015102 -0.00014081 -0.00011146 -5.1022e-05 -0.00011831 -0.00019407 -9.4111e-05 -0.00029952 -0.00017418 -0.00015904 -0.00010485 -0.00010392 -0.0001304 -3.4253e-05 -6.5596e-05 0.00011279 -0.00020102 -1.1152e-05 -1.8982e-05 5.1202e-05 -3.5849e-05

1424.8 -6.6873e-05 -4.5386e-05 -4.2939e-05 -8.2285e-05 -4.9374e-05 -3.1699e-05 -3.5747e-05 -3.2259e-05 -2.4004e-05 -5.6956e-05 -9.6316e-05 -0.00011974 -0.00017537 -0.00012919 -1.9035e-05 5.6081e-05 2.6682e-05 5.0269e-05 3.3548e-05 6.5036e-06 -1.171e-06 -1.4611e-05 1.7949e-05 7.0106e-06 -5.307e-06 2.8192e-06 -7.1276e-06 8.363e-06 -5.7686e-06 7.8066e-05 6.6714e-05 3.789e-05 -4.3983e-05 -0.00019669 -8.6428e-05 -0.00013916 -2.5405e-05 -3.1931e-06 1.3754e-05 1.9847e-05 6.5834e-06 1.7775e-05 1.2284e-05 -5.6914e-05 6.6465e-05 -4.1086e-05 -3.7975e-05 4.6619e-05 3.4978e-06 -4.2544e-05 -7.2476e-05 -2.6704e-05 3.9478e-05 0.00010253 0.00018311 8.891e-05 0.0002844 0.00011715 2.6538e-05 9.804e-06 0.00015994 8.1302e-05

1432.4 -1.3096e-05 -1.4591e-05 -1.9053e-05 -5.4292e-05 -5.0906e-05 -3.5256e-05 -3.3664e-05 -3.7542e-05 -2.1524e-05 -7.6221e-05 -6.8409e-05 -0.00011286 -0.00017943 -0.00016177 -4.4197e-05 5.0292e-05 3.6621e-05 6.5652e-05 3.1587e-05 4.4162e-06 -7.6825e-07 3.0942e-06 -6.1219e-07 1.5702e-05 2.7618e-05 2.5906e-05 -1.0549e-05 -1.8039e-05 -7.6312e-05 7.915e-06 4.6916e-05 0.00013834 0.00014664 -8.3785e-05 -7.4563e-05 -8.7507e-05 3.9053e-05 6.5513e-05 8.9742e-05 0.00010783 0.00010843 0.00014233 0.00010478 5.4263e-05 0.00017859 -1.201e-05 2.046e-05 0.00014644 0.00018077 5.0616e-05 0.00011513 3.8425e-05 1.2551e-05 0.00016803 6.0706e-05 0.00015158 -7.0563e-05 0.00017282 -4.9446e-05 -1.3327e-05 5.4721e-05 -0.00013036

1439.9 0.00010205 8.9374e-05 7.7215e-05 5.1227e-05 3.863e-05 4.6555e-05 4.3621e-05 1.7522e-05 3.6832e-05 -7.3382e-07 2.7216e-05 -3.7697e-05 -9.4556e-05 -0.00010357 -3.3666e-05 8.0856e-05 9.4825e-05 9.8797e-05 7.9004e-05 6.7755e-05 3.6126e-05 4.9147e-05 1.6339e-05 1.7074e-05 6.8322e-05 4.9842e-05 2.7675e-06 -1.6146e-05 -4.7194e-05 -1.0902e-05 7.3076e-05 9.38e-05 0.0002216 -3.2333e-05 -5.4458e-05 -5.6271e-05 6.3075e-05 9.5211e-05 0.00012181 8.8365e-05 0.00011139 0.000122 0.00016664 0.00013078 0.00019319 7.1185e-05 2.8228e-05 0.00019586 0.00019179 0.00011632 0.00020113 3.5531e-05 -2.703e-05 0.00010099 -0.0001644 7.3763e-05 -7.1281e-05 0.00010783 -6.8139e-05 -9.1319e-05 -9.2878e-05 -0.00023767

1447.4 0.00012302 9.7558e-05 9.2044e-05 7.4819e-05 6.2455e-05 5.8196e-05 4.593e-05 1.0975e-05 5.9859e-06 1.5922e-06 2.8223e-05 -4.6621e-05 -8.4035e-05 -9.2017e-05 -5.915e-05 2.6561e-05 6.1365e-05 6.8992e-05 6.4483e-05 5.6105e-05 1.3776e-05 2.6527e-05 -2.2959e-05 -3.6831e-05 2.3157e-05 2.9473e-05 -2.1236e-05 -6.066e-06 -4.4407e-05 1.2638e-05 -1.0465e-05 -8.4449e-05 0.00015545 -5.6216e-05 -5.542e-05 7.1958e-05 0.0001326 0.00014382 0.00012748 5.3937e-05 0.00010619 0.00010529 0.00014801 0.00012553 0.0001981 0.00019815 0.00013861 0.00013052 0.00015549 0.00013714 0.00013608 0.00011294 6.154e-05 7.2815e-05 -0.00016264 -7.5789e-05 4.2272e-05 3.5043e-05 0.00011133 -0.00015498 -4.772e-05 -0.00012427

1455 7.3674e-05 4.9252e-05 4.7164e-05 3.3348e-05 3.1051e-05 8.7502e-06 -9.3921e-06 -2.831e-05 -5.4554e-05 -4.6177e-05 -3.4171e-05 -6.5856e-05 -0.00011495 -9.7649e-05 -9.5673e-05 -3.8811e-05 -1.5711e-06 2.8204e-05 1.7535e-05 1.736e-07 -2.7076e-05 -1.8237e-05 -4.762e-05 -4.7263e-05 -1.8696e-06 -3.9025e-06 -4.2241e-05 -1.0172e-05 -8.8724e-05 -5.6284e-05 -0.00015242 -0.00013155 0.00010833 -1.3543e-05 5.0641e-05 0.00017233 0.00019527 0.00016154 0.00016107 0.00010688 0.00014044 0.00011003 0.00012453 0.00016514 0.00017164 0.00025725 0.00017887 8.7094e-05 6.6954e-05 4.5137e-05 0.00010843 0.00014477 0.00018553 0.00020176 -1.7263e-05 1.6379e-05 0.0001562 0.00012949 0.00016416 -5.7109e-05 -3.5048e-05 -0.00012239

1462.5 3.0674e-05 5.3733e-05 4.4068e-05 3.475e-05 2.342e-05 9.5641e-06 -7.3404e-06 -1.1291e-05 -4.8566e-05 -2.9822e-05 -4.2289e-05 -3.6008e-05 -6.4132e-05 -5.648e-05 -6.7171e-05 -4.196e-05 -1.2308e-06 3.4979e-05 4.9248e-06 5.9008e-06 -6.6941e-06 -1.6085e-05 -7.7537e-06 7.3704e-06 4.4479e-05 5.643e-06 -1.1421e-05 -2.1087e-05 -8.7182e-05 -7.2987e-05 -0.00016404 -1.7339e-06 0.00017749 0.00010979 0.0001556 0.00012242 0.00016616 0.00010881 0.00010739 7.6391e-05 0.00017465 0.0001051 0.00014626 0.00020062 8.0374e-05 0.00019643 9.7945e-05 6.1117e-05 -9.4467e-06 -5.632e-05 0.00011019 4.8259e-05 0.00014914 0.00026674 -5.8845e-06 0.00013948 0.00033601 2.7804e-05 -1.8952e-05 -3.8092e-05 -8.5004e-05 -0.00030943

1470 0.0001153 0.00015202 0.00013662 0.0001151 0.00012578 9.7857e-05 9.2555e-05 9.2702e-05 6.7181e-05 7.87e-05 6.2246e-05 5.6308e-05 5.5664e-05 5.0203e-05 1.4512e-05 3.8553e-05 8.3452e-05 8.1262e-05 8.14e-05 9.3843e-05 6.9778e-05 7.0769e-05 5.9757e-05 8.0449e-05 0.00013485 6.442e-05 1.1357e-05 2.0832e-06 1.9674e-06 2.5809e-05 -3.4146e-05 0.0001624 0.00012562 0.00017538 0.00018837 5.9358e-05 2.9925e-05 1.3695e-06 -3.4407e-05 -4.3077e-05 0.00012342 7.6235e-05 0.0001276 0.0001004 -4.973e-06 5.6117e-05 3.5567e-05 -6.1043e-05 -7.0484e-05 -0.00010484 -5.2252e-06 9.3006e-05 8.9991e-06 0.00025715 -4.2316e-05 5.9261e-05 0.00012366 -0.00011285 -0.00014374 -0.00015104 -5.2597e-05 -0.0003702

1477.5 0.000129 0.00015099 0.0001403 0.00012084 0.00014804 0.00011401 0.00011445 0.00013907 0.00012653 0.00011996 0.00011733 9.1084e-05 9.3169e-05 0.00010374 5.6341e-05 7.502e-05 0.00012681 9.7927e-05 0.00012349 0.00011678 9.6492e-05 0.00010143 8.4304e-05 9.4325e-05 0.00013648 4.9415e-05 -6.8342e-06 -2.511e-05 -3.864e-05 5.7442e-05 6.2947e-05 0.00013623 7.2559e-05 0.00018153 0.00015859 8.049e-05 7.896e-06 -3.098e-05 -5.7666e-05 -9.7768e-06 7.6641e-05 0.00010306 5.4646e-05 7.2376e-05 0.00010962 4.9974e-05 7.8631e-05 6.2486e-06 0.00010018 1.6517e-05 0.00015655 0.00018457 0.00014623 0.00035388 0.00011426 0.00011004 -8.7305e-05 -6.8596e-05 -0.00013418 1.9115e-05 2.7829e-05 -0.00032372

1485 9.8946e-05 8.7339e-05 0.0001029 9.7079e-05 9.1947e-05 9.927e-05 9.3231e-05 9.9612e-05 0.00013071 0.00012111 0.0001319 0.00010077 9.2182e-05 0.00012293 7.8029e-05 8.994e-05 0.00013985 9.4721e-05 0.00012406 9.4406e-05 9.2614e-05 7.4456e-05 8.7108e-05 4.5161e-05 6.7179e-05 -6.9495e-06 -7.2517e-05 -5.5383e-05 -7.1957e-05 -4.5325e-06 -1.9962e-05 3.9663e-05 2.9319e-05 0.00013409 0.00015126 8.9281e-05 -1.2665e-05 -4.5959e-05 -7.0584e-05 -4.1807e-05 -1.1386e-05 9.2588e-05 -1.5663e-05 9.8884e-05 0.00010089 4.305e-05 7.3638e-05 0.00020757 0.00024803 0.00018798 0.00037423 0.0002366 0.00034603 0.00041113 0.00021567 0.00026919 -0.00014598 0.00011824 2.2113e-05 0.00015435 5.8449e-05 -0.00016346

1492.5 8.5631e-05 6.0051e-05 8.2264e-05 5.5888e-05 4.9028e-05 9.2874e-05 9.6224e-05 6.0633e-05 0.00011757 0.00012066 0.00010039 9.6086e-05 8.6527e-05 0.00011495 8.8304e-05 0.00012981 0.00014281 9.2678e-05 9.2851e-05 6.8322e-05 7.4826e-05 5.774e-05 6.2512e-05 -1.2781e-05 1.7751e-05 -4.8071e-05 -9.9411e-05 -0.00010729 -0.00012851 -0.00011053 -0.00022516 -0.00010514 -1.272e-05 0.0001319 0.00015278 4.1786e-05 -3.245e-05 -0.00013008 -0.0001623 -0.00012439 -0.00011088 -6.219e-05 -0.00015288 -3.7069e-05 -5.2372e-05 -6.4471e-06 7.1325e-05 0.0003146 0.00038856 0.00032642 0.00032412 0.00029298 0.00034468 0.00033028 8.9606e-05 0.00031797 0.0001867 0.00016478 0.00011164 2.1519e-05 -7.1624e-05 -0.00014898

1499.9 5.4493e-05 3.5479e-05 3.9193e-05 2.1743e-05 2.8655e-05 6.6093e-05 6.4657e-05 2.8355e-05 8.8048e-05 8.4747e-05 5.8924e-05 7.7773e-05 4.5825e-05 0.00012471 7.7685e-05 0.00011999 0.00011465 5.4935e-05 6.6367e-05 4.4459e-05 5.7819e-05 6.6013e-05 4.5357e-05 -3.0723e-05 4.0757e-07 -3.4723e-05 -8.3697e-05 -9.2596e-05 -0.00011828 -0.0001751 -0.00024333 -0.00018425 -0.00014228 6.859e-05 0.00013983 -8.4093e-05 -5.5774e-05 -0.00025233 -0.00030076 -0.00025255 -0.00030146 -0.00026137 -0.0002445 -9.0484e-05 -5.8174e-05 3.48e-05 0.00017083 0.00031161 0.00039096 0.00036997 0.00036676 0.00035142 0.00042352 0.00036925 4.406e-05 0.00030907 0.0005733 8.6347e-05 0.00015725 -6.2916e-06 -0.00012916 -2.3111e-05

1507.4 -4.2885e-05 -3.6189e-05 -5.0973e-05 -9.3579e-06 -2.2738e-05 1.8853e-05 4.7115e-06 -2.6588e-05 2.5082e-05 3.1408e-05 3.2799e-05 2.9753e-05 4.0969e-06 0.00010851 5.7293e-05 4.9835e-05 7.5629e-05 -3.4181e-06 3.8768e-05 3.6976e-05 4.8328e-05 5.544e-05 7.6019e-05 -3.0349e-05 7.0125e-06 6.9944e-06 1.7905e-05 -2.7284e-05 -6.8112e-05 -4.7542e-05 -0.00016845 -0.00027374 -0.00025355 -0.00014367 -7.6352e-05 -0.00025098 -0.00020493 -0.00036891 -0.00041974 -0.00040936 -0.00048724 -0.00041037 -0.00025254 -9.5555e-05 3.3703e-06 0.00013059 0.00022233 0.00024859 0.00016892 0.00020013 0.00043022 0.00034881 0.00043041 0.00040007 0.00023992 0.00034447 0.00053919 0.00014968 0.00023804 0.00012974 -4.7385e-05 0.00016495

1514.8 -8.2124e-05 -4.9467e-05 -7.7384e-05 -1.2394e-05 -5.8966e-05 9.3654e-06 6.9567e-06 -4.0572e-05 2.3022e-05 3.057e-05 4.8571e-05 3.2995e-06 1.383e-05 0.00013856 7.0842e-05 4.6913e-05 0.00010666 8.3634e-06 7.5615e-05 8.7843e-05 0.00010049 9.5847e-05 0.00013498 2.9399e-05 9.0565e-05 6.6041e-05 0.00013879 0.00010205 0.00010298 7.1538e-05 -8.027e-05 -0.00023099 -0.00032393 -0.00025464 -0.00027404 -0.00039484 -0.0005128 -0.00055143 -0.00054312 -0.00059821 -0.00060476 -0.00046752 -0.00028713 -0.00018722 -0.00010876 1.9506e-05 1.9252e-05 2.1639e-05 -0.0001122 -5.1535e-05 0.00017252 0.00019589 0.00027661 0.00025482 0.00029982 0.00035438 0.00043174 0.00048119 0.00025471 0.0003682 0.0001511 0.00027488

1522.3 -0.00010407 -8.7867e-05 -9.8798e-05 -6.4277e-05 -0.00011577 -4.0202e-05 -1.8412e-05 -8.3481e-05 -5.5095e-06 1.643e-05 9.0497e-06 -5.0449e-05 1.5204e-05 0.00015436 7.4736e-05 7.028e-05 0.00016124 6.6352e-05 0.00014472 0.00016583 0.00018076 0.00015095 0.00018536 0.00011106 0.00017706 0.0001369 0.00017338 0.00012609 0.0001392 9.7314e-05 -0.00011932 -0.00024983 -0.00026105 -0.00021657 -0.00032159 -0.00041878 -0.0005929 -0.00066202 -0.00066107 -0.00066253 -0.0005857 -0.00042033 -0.00031422 -0.0002383 -0.00027202 -0.00025513 -0.00027672 -0.00035248 -0.00031907 -0.0003167 -8.5174e-05 -0.00013891 5.36e-05 0.00020034 0.00036586 0.00037458 0.00059946 0.00063363 0.00038796 0.0003936 0.00058388 0.00033846

1529.7 -0.00016178 -0.00017773 -0.00016089 -0.00015497 -0.00020621 -0.00014717 -9.8608e-05 -0.00019762 -9.6334e-05 -8.4815e-05 -0.00014303 -0.00016427 -9.5756e-05 9.3906e-05 1.8537e-05 6.0392e-05 0.00014241 3.574e-05 0.00013547 0.00014637 0.00019078 0.00016177 0.00018862 0.000103 0.00015395 9.627e-05 1.7546e-05 -6.7129e-05 -0.00014256 -0.00022928 -0.00036941 -0.00037621 -0.00030109 -0.00019311 -0.00018086 -0.00023886 -0.00042232 -0.00049884 -0.00055541 -0.00047689 -0.00033146 -0.00022996 -0.00017131 -0.00028938 -0.00037538 -0.00055804 -0.00052064 -0.00064742 -0.00058287 -0.00052425 -0.0001912 -0.00016968 2.0645e-05 0.00023074 0.00037674 0.00037171 0.00089932 0.00041668 0.00041123 0.00046148 0.00075827 0.0004672

1537.1 -0.00026963 -0.0002896 -0.00028358 -0.00028368 -0.0003418 -0.00028003 -0.00023663 -0.00035083 -0.00024666 -0.00024949 -0.00036551 -0.00037052 -0.00027659 -5.0851e-05 -5.2648e-05 -3.1302e-05 3.3482e-05 -7.5147e-05 2.4422e-05 2.9268e-05 9.6656e-05 8.4299e-05 0.00011823 -2.2865e-05 -1.0132e-05 -9.423e-05 -0.00022249 -0.00044775 -0.00065452 -0.00079492 -0.00091697 -0.00065727 -0.0004849 -0.00021701 -3.1061e-05 0.00019706 0.0001155 0.00012619 0.00016087 0.00032172 0.00038124 0.0003334 0.0001445 -0.00019543 -0.00035865 -0.00046239 -0.00046497 -0.00050524 -0.00038957 -0.000252 0.00010312 5.6051e-05 7.8318e-05 0.00019218 0.00010894 0.00013853 0.00087479 0.00014231 0.00031583 0.00049705 0.00053257 0.00045467

1544.5 -0.00029102 -0.00029055 -0.00030437 -0.00030502 -0.00034503 -0.0002893 -0.00027301 -0.00035824 -0.00029506 -0.00032237 -0.00044607 -0.00048504 -0.00039097 -0.00013321 -8.2541e-05 -0.00010928 -5.7714e-05 -0.00018875 -8.9689e-05 -0.00010505 -5.7773e-05 -6.8373e-05 -3.5717e-05 -0.00024994 -0.00021711 -0.00033508 -0.0005216 -0.00084349 -0.0011218 -0.0014206 -0.0015929 -0.0011877 -0.0010066 -0.00032604 0.00027971 0.00088232 0.001103 0.0013375 0.0015333 0.0015993 0.0013867 0.0011719 0.00075065 0.00041371 0.00024778 0.00033486 0.00029303 0.00031658 0.00044029 0.00026491 0.00032557 0.00017011 -3.2709e-05 1.2033e-05 -0.0001637 -0.00010529 0.00026376 -0.00017942 0.00012453 0.00035206 0.00016878 0.00030179

1551.9 -0.00019264 -0.00016991 -0.000179 -0.00018005 -0.00018882 -0.00017372 -0.00017253 -0.00024822 -0.00022006 -0.00026284 -0.00038612 -0.00047556 -0.00037952 -0.00013346 -5.5104e-05 -0.00010118 -0.00011674 -0.00027147 -0.00021599 -0.00024233 -0.00023806 -0.00025459 -0.00025133 -0.00046957 -0.00046067 -0.00056917 -0.00078928 -0.0011735 -0.0015077 -0.0018791 -0.00207 -0.001668 -0.0011594 -0.00012451 0.00083754 0.0018845 0.0025377 0.0029075 0.0029558 0.0028311 0.0023386 0.0019945 0.0015722 0.0013401 0.0012394 0.0012934 0.0012252 0.0010352 0.00091589 0.00038868 0.00018985 0.00019511 -0.0001523 -8.0175e-05 -0.0002108 -0.00024785 0.00010302 -0.00049337 -0.00016299 0.00027121 -3.446e-05 0.00010475

1559.3 9.853e-06 2.0793e-05 2.6379e-05 1.7335e-05 1.8328e-05 -3.6006e-06 -8.1882e-06 -8.3396e-05 -8.8623e-05 -0.00015431 -0.00029138 -0.00041474 -0.00030378 -2.2158e-05 1.8864e-05 -1.7763e-05 -8.9943e-05 -0.00025785 -0.00026371 -0.00031137 -0.00035737 -0.00035394 -0.00038168 -0.00056364 -0.00058718 -0.00068658 -0.00092009 -0.0012555 -0.0016005 -0.0019115 -0.0018416 -0.0010596 -0.00026945 0.0008584 0.0019699 0.0031344 0.0037243 0.0038744 0.0034601 0.0030143 0.0023768 0.0020314 0.0016412 0.0014581 0.0012924 0.0012344 0.0011083 0.00083371 0.00070654 0.00038704 0.00019043 0.0002494 1.6544e-05 1.7101e-05 1.123e-05 -0.00022163 0.00028241 -0.0005239 -0.00022163 8.1696e-05 -0.00011422 -0.00020232

1566.7 0.00021499 0.00022105 0.00021203 0.00018331 0.00016108 0.00013721 0.0001041 4.8485e-05 3.3298e-05 -8.5607e-05 -0.000265 -0.00041667 -0.00027379 5.8759e-05 0.00012309 9.6046e-05 2.3343e-05 -0.0001285 -0.00015024 -0.00021055 -0.00026262 -0.00025423 -0.00027307 -0.00043764 -0.00044523 -0.00055402 -0.0007726 -0.001156 -0.0014841 -0.0014553 -0.0008599 0.00058081 0.0017908 0.0028888 0.0036973 0.004268 0.0041646 0.0034266 0.0023747 0.0014255 0.00053284 0.00015542 -3.0401e-05 2.9217e-05 0.00022796 0.000323 0.00043464 0.00039881 0.00051574 0.0005513 0.00043245 0.00045814 0.00035487 0.00024906 0.00030626 4.7702e-05 0.00055373 -0.00013947 2.4957e-05 3.9852e-05 -6.8443e-05 -0.00032745

1574 0.00026721 0.00028811 0.00024985 0.00020727 0.00014162 0.00015781 0.00010231 2.4752e-05 7.3642e-06 -0.00016753 -0.0004383 -0.00063622 -0.00039234 9.6193e-05 0.00025165 0.00024965 0.00015813 3.0311e-05 1.7336e-05 -1.4602e-05 -3.0817e-05 -1.2345e-05 1.9076e-05 -0.00011122 -6.4487e-05 -0.00023638 -0.00046422 -0.0009493 -0.0012805 -0.0010613 0.00011781 0.002232 0.0039192 0.00498 0.0052152 0.0043669 0.0029339 0.00097212 -0.00075613 -0.0020175 -0.0026316 -0.00235 -0.0016746 -0.0010827 -0.00026832 -5.8788e-05 0.00025963 0.00031765 0.00045176 0.00063827 0.0006627 0.00072883 0.0005483 0.00057984 0.00060166 0.00033527 0.00068918 0.00020661 0.00027477 8.5633e-05 0.00010682 -0.00023503

1581.4 0.00023307 0.0002349 0.0002248 0.00017572 0.00010394 0.00014412 9.8782e-05 -2.8784e-05 -3.1143e-05 -0.00024808 -0.00068765 -0.00099053 -0.000574 0.00024199 0.00057016 0.00051983 0.00040138 0.00024846 0.0002328 0.00019766 0.00019461 0.00021647 0.00028514 0.00023102 0.0002925 0.00010389 -5.1954e-05 -0.00063775 -0.0011141 -0.00102 8.891e-05 0.0021404 0.0039891 0.0045019 0.003757 0.0013914 -0.001076 -0.0032569 -0.0040847 -0.0039063 -0.0027176 -0.001593 -0.00049326 -4.6548e-05 0.00046163 0.00057842 0.00071077 0.00070559 0.00066623 0.00072809 0.00083909 0.00088623 0.00061336 0.00072647 0.00081633 0.000558 0.0004965 0.00027482 0.00041524 0.00025533 0.00029184 0.00015423

1588.8 0.00023847 0.00022695 0.00025076 0.00024567 0.00017927 0.00022274 0.00022018 7.0316e-05 0.00010882 -0.00015297 -0.00078826 -0.001411 -0.00086063 0.00054196 0.001137 0.0010166 0.00080828 0.00048866 0.00045038 0.00033614 0.00027498 0.00023866 0.00028479 0.00017876 0.00021786 4.3633e-05 -0.00015522 -0.0007952 -0.0014802 -0.0021797 -0.0021406 -0.0016581 -0.0014132 -0.0022287 -0.0034167 -0.0046668 -0.0045825 -0.0029033 -0.00072164 0.0010999 0.0024273 0.0028001 0.0028477 0.0024571 0.0019326 0.0017177 0.0015209 0.0013025 0.0011004 0.00088199 0.00087562 0.00080143 0.00067851 0.00064093 0.00061754 0.00055731 0.00048471 0.00034543 0.00047781 0.00041876 0.00036738 0.00037734

1596.1 0.00016784 0.00019256 0.00024749 0.00028924 0.00025039 0.00033975 0.00037001 0.00021824 0.00030298 4.5201e-05 -0.00075471 -0.0018175 -0.0013144 0.00078064 0.0017964 0.0015909 0.0012514 0.0006631 0.00057996 0.00034126 0.0001888 3.9568e-05 -3.7219e-05 -0.00048901 -0.00058289 -0.00096439 -0.0015293 -0.0027526 -0.004161 -0.0062294 -0.0082811 -0.011194 -0.013561 -0.014048 -0.011123 -0.0041612 0.002382 0.007713 0.0096338 0.0098932 0.0086277 0.0074482 0.0060424 0.0049589 0.0035148 0.0029191 0.002431 0.0019821 0.0015554 0.001165 0.00099282 0.00085037 0.00086649 0.00055683 0.00058387 0.00063539 0.00075857 0.0004809 0.00056528 0.0007113 0.00057561 0.00052058

1603.4 5.3634e-05 0.00013437 0.00021819 0.00030285 0.00029393 0.0004486 0.00048336 0.00035933 0.00044497 0.00018698 -0.00068397 -0.0019796 -0.0016928 0.00079127 0.0022158 0.0019991 0.0015672 0.00072602 0.00062912 0.00028401 6.5566e-05 -0.00020571 -0.00043715 -0.0015178 -0.0018452 -0.0027773 -0.0042416 -0.007261 -0.010584 -0.015442 -0.020218 -0.025104 -0.025523 -0.01739 -0.004413 0.010026 0.016924 0.019247 0.017527 0.015265 0.011938 0.0098047 0.0075884 0.0062178 0.0043972 0.0036596 0.0029696 0.0025769 0.0019622 0.0013982 0.0012617 0.0012361 0.00106 0.00071595 0.00075838 0.00088655 0.00097517 0.00059815 0.00080984 0.00094533 0.00087596 0.00063946

1610.7 0.00010805 0.00019675 0.00028803 0.00034341 0.00034389 0.00049922 0.00052893 0.00043557 0.00048877 0.00014991 -0.00073993 -0.0019567 -0.0017353 0.00055794 0.002095 0.0019812 0.0015618 0.00066482 0.00058385 0.00021505 -1.4412e-05 -0.00036567 -0.00066863 -0.0023338 -0.0029539 -0.0045574 -0.0072298 -0.013345 -0.020416 -0.029589 -0.035301 -0.032063 -0.020101 -0.00097999 0.013765 0.02351 0.024454 0.022311 0.018403 0.015173 0.011527 0.0094015 0.007212 0.0059073 0.0041872 0.0034751 0.002875 0.0025881 0.0019431 0.0014183 0.0013503 0.00141 0.0010554 0.00089969 0.00073331 0.0010213 0.000656 0.00054487 0.00092625 0.00097884 0.00090704 0.00081322

1618 0.0002585 0.00031327 0.00038629 0.00035183 0.00032862 0.00042315 0.00043007 0.00034587 0.00035807 -4.8821e-05 -0.00098388 -0.0019768 -0.0015423 0.00029172 0.0014572 0.0014805 0.0012145 0.00046464 0.00043415 0.00011627 -0.00011691 -0.00045869 -0.00073861 -0.002736 -0.0035707 -0.0057891 -0.0097424 -0.019425 -0.03098 -0.041762 -0.039573 -0.01904 0.0010046 0.017036 0.023412 0.025284 0.022414 0.018575 0.014641 0.011744 0.0088929 0.0073146 0.0056743 0.0047102 0.0033066 0.0028098 0.002423 0.0021112 0.0015512 0.0012467 0.0011987 0.0012425 0.0010021 0.00089929 0.00070408 0.00091279 0.00060161 0.00048082 0.00078298 0.00084755 0.00069613 0.00079032

1625.3 0.00035875 0.0003684 0.00037212 0.00028939 0.00020959 0.00022058 0.00018131 8.2459e-05 6.7959e-05 -0.00031834 -0.0012993 -0.0021401 -0.0014608 -6.4664e-05 0.00069136 0.00077481 0.00069115 0.00016185 0.00015256 -0.00012914 -0.00036753 -0.00067657 -0.00091111 -0.0030004 -0.0041037 -0.0070061 -0.0123 -0.024383 -0.035971 -0.038703 -0.024907 -0.00050232 0.013471 0.020667 0.021419 0.019862 0.016381 0.012902 0.0099184 0.0078277 0.0059003 0.0049659 0.0039399 0.0034097 0.0023172 0.0020326 0.0018082 0.0014716 0.0011653 0.0010175 0.00098133 0.00099744 0.00079575 0.00067335 0.00056113 0.00061067 0.00047127 0.00035553 0.00052814 0.00050971 0.00037901 0.00033699

1632.6 0.00023336 0.0002107 0.00015986 6.3699e-05 -4.0628e-05 -8.6929e-05 -0.00015085 -0.00025794 -0.00028801 -0.00057391 -0.0014325 -0.0021464 -0.0015634 -0.00051387 2.5517e-05 0.000124 0.00014348 -0.00021199 -0.00024613 -0.00050355 -0.00076672 -0.0010391 -0.0013005 -0.0033051 -0.0047841 -0.0080686 -0.013582 -0.022224 -0.026186 -0.020913 -0.0084043 0.0057371 0.012239 0.014893 0.013986 0.012162 0.0097766 0.0075453 0.0057366 0.0045635 0.0033713 0.0028938 0.0024464 0.0022178 0.0015353 0.0013183 0.0012083 0.0010177 0.0010624 0.00078009 0.00075519 0.00074082 0.00060902 0.00042471 0.00032595 0.00039111 0.00041507 0.00013341 0.00028706 0.00018271 0.0001021 -4.5307e-05

1639.9 -5.8049e-05 -9.367e-05 -0.00016562 -0.00022752 -0.00032386 -0.00035752 -0.0003944 -0.00046312 -0.00048035 -0.00063372 -0.0012129 -0.0017357 -0.0014932 -0.00082479 -0.00038102 -0.00029534 -0.00028045 -0.0005048 -0.00060219 -0.00084501 -0.001102 -0.0013723 -0.0017317 -0.0034196 -0.0048612 -0.0069169 -0.0092325 -0.010594 -0.0098459 -0.0065862 -0.0016284 0.0037739 0.0061448 0.006846 0.0060381 0.0050239 0.00411 0.0031267 0.0024405 0.0019717 0.0014118 0.0012714 0.0012166 0.001164 0.0008638 0.00083983 0.00068586 0.00070606 0.00075123 0.00060194 0.00056607 0.00056983 0.00049549 0.00036677 0.00025269 0.00029271 0.0002377 5.9651e-06 0.00010632 3.8663e-05 -0.00013779 -0.00017736

1647.2 -0.00038166 -0.00043025 -0.00048706 -0.0004993 -0.00054908 -0.0005462 -0.00053371 -0.00052179 -0.00050658 -0.00054791 -0.00081925 -0.0011272 -0.0011481 -0.00082574 -0.00050217 -0.00044746 -0.00049655 -0.00070316 -0.00084444 -0.0010825 -0.0013149 -0.001534 -0.0018774 -0.0025535 -0.0028056 -0.0024309 -0.0017533 -0.00104 -0.0011563 -0.0011548 -0.00041385 0.00053121 0.00096693 0.00092692 0.00060113 0.00032247 0.00048668 0.00030496 0.00023681 0.0001815 0.00014834 0.00024251 0.00031775 0.00033605 0.00028891 0.00039817 0.00028701 0.00040491 0.00028966 0.00042082 0.00041993 0.00035593 0.0004345 0.00026769 0.00031392 0.00017713 0.00015716 7.2327e-05 7.9837e-05 0.00011009 -9.7783e-05 -7.7551e-05

1654.4 -0.00063523 -0.00065909 -0.00068686 -0.00065762 -0.00065 -0.00063572 -0.00058947 -0.00050386 -0.00046369 -0.00045911 -0.00051292 -0.00063309 -0.00065203 -0.00054718 -0.00032678 -0.00038617 -0.00051359 -0.0007652 -0.00098224 -0.0011596 -0.0013241 -0.001306 -0.0011513 -0.00034441 0.00032781 0.001107 0.0015833 0.001268 0.00066436 -0.00017502 -0.00072743 -0.0013507 -0.0016686 -0.0017868 -0.0015848 -0.0013909 -0.00094547 -0.00072668 -0.00063078 -0.00052212 -0.0003749 -0.00023332 -0.00017609 -0.00017758 -0.00011856 4.1046e-05 4.3178e-05 5.8103e-05 1.21e-05 0.00011708 0.0002649 0.00016338 0.00027653 0.00010002 0.0001476 3.2908e-05 0.00011809 2.0744e-05 -2.2661e-05 0.00024838 -5.7269e-05 0.00026754

1661.7 -0.00068467 -0.00064244 -0.00063194 -0.00058448 -0.00055433 -0.00052593 -0.00048658 -0.00041992 -0.00036167 -0.00035759 -0.00035703 -0.00033105 -0.00024537 -8.898e-05 4.8654e-05 -0.00012573 -0.00037631 -0.00071327 -0.00092106 -0.00096427 -0.00079903 -0.0002461 0.00035206 0.0011808 0.0014357 0.0013535 0.001166 0.00051591 6.2086e-05 -0.00057579 -0.0011992 -0.0019598 -0.0022098 -0.0020167 -0.0015194 -0.0010189 -0.00066897 -0.00042514 -0.00039299 -0.00034941 -0.00029373 -0.00026506 -0.00030353 -0.00033495 -0.00035114 -0.00022691 -0.00017772 -0.00022663 -0.00019547 -0.00020939 -5.0953e-05 -7.5407e-05 2.8267e-06 -7.6847e-05 -0.00016641 -9.7724e-05 -0.00028509 -0.00016145 -0.00014175 0.00018196 -4.4392e-05 0.00053197

1668.9 -0.00045511 -0.00038268 -0.00035865 -0.0002869 -0.00028191 -0.00025561 -0.00024194 -0.00023148 -0.00020834 -0.00023032 -0.00029544 -0.00031209 -0.00013351 0.00013824 0.00033085 8.4057e-05 -0.00030997 -0.00060031 -0.000568 -0.00015443 0.00043802 0.00090674 0.0010494 0.0010327 0.00089173 0.00044452 3.1501e-05 -0.00038216 -0.00067447 -0.0011311 -0.0016147 -0.0018391 -0.0017733 -0.0012059 -0.00053169 5.8147e-05 0.0002066 0.00022854 0.00017799 6.009e-05 -5.4976e-05 -0.00015175 -0.0002679 -0.00027936 -0.00046804 -0.00045269 -0.00049638 -0.00050756 -0.00034672 -0.00049562 -0.00039188 -0.00039492 -0.00032615 -0.00033066 -0.00036376 -0.00021961 -0.00029697 -0.00019889 -6.1542e-05 4.4752e-05 5.0862e-05 0.00044447

1676.1 -7.0903e-05 -3.3376e-05 -1.6333e-05 4.9303e-05 3.5864e-05 5.1692e-05 3.882e-05 2.3181e-06 -4.1858e-05 -0.00011434 -0.00031531 -0.00051031 -0.00044266 -0.00014165 0.00010478 -0.00015391 -0.00047209 -8.3954e-05 0.00057649 0.0011974 0.0013008 0.0010644 0.00079926 0.00046759 0.00019762 -0.00025774 -0.00053605 -0.00078005 -0.0011309 -0.0015247 -0.0018353 -0.0015053 -0.00099361 -0.00011202 0.0004845 0.00092817 0.00094622 0.0007644 0.00055697 0.00029266 0.00012118 -4.7582e-05 -0.00016664 -0.00020255 -0.00043075 -0.00052034 -0.00056603 -0.00063571 -0.0004283 -0.0005991 -0.00050664 -0.00048574 -0.00055977 -0.00038942 -0.00048022 -0.0003439 -0.00033404 -0.00017696 1.4452e-05 0.00013641 0.0001004 0.00033087

1683.4 0.00026709 0.00027521 0.00030172 0.00032172 0.0003194 0.00031661 0.0002877 0.00022316 0.00016322 3.9014e-05 -0.00030982 -0.0007529 -0.0010147 -0.0010088 -0.00090057 -0.00080105 -0.0001491 0.0015272 0.0020487 0.0018062 0.0012701 0.00081519 0.00052776 0.00011638 -0.00011281 -0.00037877 -0.00053056 -0.0008093 -0.0012544 -0.0016888 -0.001837 -0.0009621 -0.00020228 0.00065985 0.0010458 0.0013394 0.0011891 0.0008804 0.00063607 0.00031151 0.00019549 2.8648e-05 -0.00012258 -0.00017231 -0.0004066 -0.00055163 -0.00050431 -0.00051204 -0.0004766 -0.00045761 -0.00049051 -0.00044164 -0.00052887 -0.00026596 -0.00044912 -0.00033865 -0.00047737 -0.00022028 3.2246e-07 8.6603e-05 -3.588e-05 0.00032682

1690.6 0.00041424 0.00042245 0.00046086 0.00043044 0.0004215 0.00043171 0.00041111 0.00034614 0.00033628 0.00019107 -0.00024124 -0.00091222 -0.0018164 -0.00252 -0.0023287 -0.00042195 0.0015658 0.0028077 0.0023521 0.0015725 0.0010297 0.00061733 0.00034656 2.7135e-05 -7.0413e-05 -0.0001898 -0.00033901 -0.0007443 -0.0012752 -0.001614 -0.0015165 -0.00057674 0.00021818 0.00089047 0.0010167 0.0012177 0.001043 0.0007369 0.00051076 0.00022796 0.00013309 5.8703e-05 -0.00012404 -0.0001704 -0.00031165 -0.00043148 -0.00046559 -0.00039894 -0.00042515 -0.00027353 -0.0002804 -0.00035269 -0.00036773 -0.00027624 -0.0002241 -0.0002508 -0.00035843 -0.00021164 -3.4449e-05 -4.3165e-05 -0.00015076 0.00030405

1697.8 0.00059843 0.00059028 0.00061371 0.00058167 0.0005291 0.00057094 0.00056974 0.00052612 0.0005552 0.00044539 2.1928e-05 -0.00099652 -0.00307 -0.0043782 -0.0020463 0.0016132 0.0030363 0.0027893 0.0020915 0.0014319 0.00095131 0.00060896 0.00038121 0.0002338 0.00025365 0.00012568 -9.2532e-05 -0.00057407 -0.0010215 -0.0012505 -0.0010362 -0.00034322 0.00018828 0.00066786 0.00078947 0.00094873 0.00092205 0.00070864 0.0004701 0.00025997 0.00012835 9.74e-05 -1.6662e-05 -0.00011052 -0.0001043 -0.00028827 -0.00034142 -0.00026808 -0.00035079 -0.0001017 -1.0379e-05 -0.00018496 -0.00022492 -0.00026076 -0.00013464 -0.00012894 -4.6866e-05 -1.9258e-05 -1.5678e-05 -0.00018506 -8.1578e-05 0.00013323

1704.9 0.00076588 0.00074089 0.00074713 0.00072316 0.00064159 0.00070322 0.00069983 0.00069673 0.00075021 0.00069023 0.00020717 -0.0016157 -0.0045479 -0.0041766 6.9705e-05 0.0026174 0.0028992 0.0023446 0.0018548 0.0013607 0.00094787 0.000689 0.00055129 0.00053221 0.00055634 0.00037076 0.00015371 -0.00028705 -0.00068231 -0.00083184 -0.00061961 -0.00034827 4.0807e-05 0.00041873 0.00061924 0.00077185 0.00091003 0.00072847 0.00054014 0.00037967 0.00022722 0.00017278 0.00010343 3.6982e-05 3.4428e-05 -7.8021e-05 -8.922e-05 -5.5342e-05 -0.00015991 7.9213e-05 0.00016167 1.4187e-05 -0.00010158 -9.4588e-05 -4.9177e-05 6.0521e-05 0.00031684 0.00010456 0.00013627 4.9326e-06 5.3063e-05 2.2621e-05

1712.1 0.00078808 0.00074382 0.00074981 0.00071299 0.00065546 0.00071507 0.00070897 0.00072622 0.00080508 0.0006708 -0.00028994 -0.0027208 -0.0038761 -0.0016284 0.0010999 0.0020549 0.0021326 0.0018024 0.001514 0.00116 0.00085591 0.00070013 0.00068228 0.00063165 0.00064592 0.00047461 0.0002743 -8.1188e-05 -0.00038565 -0.00059235 -0.00052182 -0.00031755 -0.00012042 0.00020113 0.00039045 0.00064747 0.00078715 0.00067761 0.00054627 0.00040386 0.00028124 0.00021871 0.00022191 0.00014232 7.9278e-05 0.00010337 5.1098e-05 8.902e-05 5.3884e-05 0.00011101 0.00023999 0.00017473 0.00014633 6.007e-05 0.00016179 0.00015996 0.00044856 0.00016562 0.00016528 0.00032146 3.3829e-05 0.00032903

1719.3 0.00075914 0.00072741 0.0007484 0.00070117 0.00067952 0.0007228 0.00071923 0.00069361 0.00069941 0.00023404 -0.0011022 -0.0023124 -0.0015276 -4.2249e-05 0.0010013 0.0014002 0.0015107 0.0013983 0.0012126 0.00097664 0.00082293 0.00075601 0.00078375 0.00069298 0.00070564 0.00052294 0.00039231 0.00011651 -0.00022567 -0.00046554 -0.00041865 -0.00020564 -0.00011908 0.00015193 0.00034121 0.00054837 0.00067699 0.00062677 0.00051233 0.00043912 0.0003488 0.00033603 0.00034455 0.0002277 0.00023864 0.00013645 0.00012138 0.00016746 0.00030864 0.00017993 0.00042262 0.00028925 0.00039124 0.00018482 0.00029603 0.00028693 0.00017665 0.00029884 0.00025378 0.00039033 5.6377e-05 0.00043409

1726.4 0.00067301 0.00065885 0.00068008 0.00064245 0.0006266 0.00064744 0.00064092 0.00047877 0.00026665 -0.00030526 -0.00088547 -0.00082575 -0.00025467 0.00032445 0.00074712 0.00096494 0.0010674 0.00099799 0.00089886 0.00080137 0.00075277 0.00073811 0.0007381 0.00064733 0.00066413 0.00053245 0.00044472 0.00025604 -1.8087e-05 -0.00025174 -0.00023794 -5.6426e-06 6.965e-06 7.2901e-05 0.00028486 0.00031824 0.00046274 0.00041472 0.00035788 0.00041084 0.00030801 0.00036405 0.00038838 0.00024394 0.00037431 0.00018601 0.00023209 0.00020762 0.00042504 0.00028462 0.00049755 0.0003386 0.00043512 0.0003094 0.00037988 0.00026706 3.5246e-05 0.00036668 0.00036484 0.00038726 0.00018725 0.00027693

1733.6 0.00050492 0.00050567 0.00053069 0.00050156 0.0004726 0.00045741 0.0004022 0.00011981 -7.9439e-05 -0.00020911 -0.00015857 -7.8794e-05 7.7034e-05 0.00034226 0.00053724 0.00064855 0.0007064 0.00062147 0.00060902 0.00060922 0.0006025 0.00058726 0.00055447 0.00048274 0.00050307 0.00045552 0.0004106 0.00030937 0.00010939 -4.6656e-05 -8.4417e-05 7.1241e-05 1.3212e-05 2.9202e-05 0.00011762 0.00012067 0.00025558 0.00025551 0.00022888 0.00031715 0.0001939 0.00029552 0.0002984 0.00024137 0.00040856 0.00025067 0.00033455 0.00023712 0.00041971 0.00027863 0.00042273 0.00040126 0.00040331 0.00033975 0.00042952 0.00023481 0.00026806 0.00046393 0.00031112 0.00038078 0.00024366 0.00017185

1740.7 0.0003927 0.00039474 0.0004166 0.00038726 0.00034693 0.00027265 0.00016955 2.8116e-05 3.0389e-05 0.00013757 0.00016491 0.00012186 0.00013376 0.00028352 0.00039648 0.00042194 0.00046649 0.00037283 0.00042756 0.00045058 0.00044657 0.00041615 0.00039662 0.00030165 0.00034672 0.00036204 0.00037099 0.00030428 0.00021577 4.8298e-05 -6.2645e-05 8.1365e-05 -1.1983e-05 1.7057e-05 -6.0872e-06 7.1374e-05 0.00011956 0.00015825 0.00016235 0.0002428 0.00015647 0.00027098 0.00022573 0.00023728 0.00033972 0.00025791 0.00029716 0.00028581 0.00037283 0.00020989 0.00031306 0.0004149 0.00038074 0.00037152 0.00033983 0.00033277 0.00047846 0.00043761 0.0002541 0.00019864 0.00017076 0.00012196

1747.8 0.00031512 0.00028932 0.00030325 0.00027778 0.00021738 0.00014431 0.00011669 0.00015052 0.00019081 0.00023695 0.00020829 0.0001377 0.00013999 0.00020762 0.00027994 0.00026963 0.00030288 0.00026942 0.00033185 0.00031555 0.00029903 0.00026986 0.00027924 0.00018159 0.00024608 0.00025409 0.00033426 0.00029036 0.00022543 9.5271e-05 -2.8622e-06 3.2356e-05 -4.863e-06 9.3468e-05 -6.4066e-05 -2.4357e-05 6.5151e-05 8.2602e-05 9.2816e-05 0.0002114 0.00014841 0.00023685 0.00017591 0.00024169 0.00026672 0.00019586 0.00015189 0.00022727 0.0002979 0.00015993 0.00019652 0.00031363 0.00023608 0.00029316 0.00019438 0.00024654 0.00023349 0.00026024 0.00014377 8.1674e-05 0.00012947 7.63e-05

1755 0.00024465 0.00022677 0.00022667 0.00020742 0.00017715 0.00017974 0.00019574 0.00023412 0.00025438 0.00024108 0.00021631 0.00016129 0.00014985 0.00021045 0.00020058 0.00021446 0.00025614 0.00025221 0.00029225 0.00024664 0.00021716 0.00020835 0.00023139 0.00018949 0.00024096 0.00023208 0.00031072 0.00026247 0.00024533 0.0002302 0.00015168 7.6236e-05 1.9972e-05 3.9149e-05 -7.7577e-05 -5.0228e-05 3.451e-05 6.3075e-05 9.2572e-05 0.00023884 0.00010439 0.00020939 0.00017757 0.00023166 0.00022527 0.00011177 7.5144e-05 0.00015038 0.00025192 0.0002113 0.0002587 0.00025839 0.000213 0.00012213 0.0001528 0.00018 -6.4928e-05 0.00024932 -1.0507e-05 5.0733e-05 0.0002028 0.00010788

1762.1 0.00014488 0.00014685 0.00016208 0.0001698 0.0001833 0.00018999 0.00020055 0.00021576 0.00021708 0.00019032 0.00019195 0.00012889 0.00014385 0.00016863 0.00013922 0.00018105 0.00021645 0.00021941 0.00021194 0.00017799 0.0001624 0.00015408 0.00017616 0.00016623 0.00020149 0.00015702 0.00023223 0.00017434 0.00014391 0.00024458 0.00022565 2.6797e-05 2.7592e-06 -4.1586e-05 -7.2817e-05 -1.6283e-05 3.8939e-05 9.9639e-05 0.00012975 0.00020378 4.4661e-05 0.00017452 0.00017118 0.00012837 0.00011918 0.00010811 7.8649e-05 0.00016261 0.00020598 0.00017945 0.00019673 0.00019464 0.00019101 9.2036e-05 0.00020539 0.00019671 0.0001466 0.00018087 -2.471e-05 0.00024397 0.0001425 0.00010969

1769.2 8.0928e-05 8.9097e-05 0.00012347 0.0001476 0.0001693 0.00014967 0.00016561 0.0001568 0.00015005 0.0001196 0.00014815 9.8582e-05 9.4109e-05 9.5083e-05 0.00014106 0.00015504 0.00019023 0.00015026 0.00012345 0.00012353 0.00013212 0.00011644 0.00013048 8.7373e-05 9.5554e-05 6.9482e-05 0.00013507 8.3161e-05 0.00014795 0.00019059 0.00015916 -6.5048e-05 -0.00011609 -6.4487e-05 1.5872e-05 6.0155e-05 8.7389e-05 4.5168e-05 0.00012795 0.00010022 4.2072e-06 0.00012105 0.00014326 4.7049e-05 3.1985e-05 9.1978e-05 0.0001289 0.00016604 0.00013582 0.00012175 8.8596e-06 0.00015606 9.1718e-05 0.00014295 0.00015461 0.00015564 0.00039853 0.00019065 -3.8265e-06 0.000329 9.9941e-05 8.6636e-05

1776.3 3.3688e-05 8.7364e-05 0.00011024 0.00011507 0.0001353 0.00013293 0.00012838 0.00010966 0.0001018 7.3406e-05 0.00012316 5.262e-05 5.2169e-05 3.756e-05 0.000154 0.00015031 0.00014186 0.00010194 8.9362e-05 9.2976e-05 0.00011865 9.203e-05 9.0416e-05 4.4158e-05 4.7363e-05 2.0674e-05 6.9508e-05 6.6273e-05 0.00017183 0.0001427 5.566e-05 -0.00012377 -0.00010739 6.4959e-05 0.00013778 0.00014736 0.00012382 4.5175e-05 7.9425e-05 5.4533e-05 2.2449e-05 4.7959e-05 7.6045e-05 6.6689e-05 -1.6418e-05 8.3067e-05 7.7539e-05 0.00017786 0.00015522 6.735e-05 -2.6326e-05 4.4664e-05 -3.6995e-05 0.00011103 3.0277e-05 0.00011619 0.0003703 1.5381e-05 -0.00011308 0.00014984 4.176e-05 -1.7848e-05

1783.3 -3.0258e-05 4.279e-05 5.4948e-05 3.5096e-05 5.1317e-05 6.2375e-05 5.2141e-05 3.4604e-05 2.2169e-05 9.1467e-06 4.4559e-05 2.7852e-06 -1.1277e-05 1.255e-05 9.7155e-05 7.7305e-05 4.6502e-05 2.481e-05 3.5071e-05 3.2024e-05 5.042e-05 2.7946e-05 2.2587e-05 -1.1633e-05 2.0326e-05 -7.6138e-07 3.8872e-05 3.606e-05 0.00013872 2.3397e-05 -8.0204e-05 -4.7251e-05 2.3608e-05 0.00013079 0.00017764 0.00015747 3.3654e-05 9.9715e-06 -4.3636e-05 -1.889e-05 1.8621e-05 -2.537e-05 -2.1187e-05 0.00013794 -3.5525e-05 9.1564e-05 1.0758e-05 0.00011108 0.00010931 -2.4891e-05 -1.196e-05 -6.042e-05 3.1359e-05 0.00013729 -8.0967e-05 0.00014174 0.00022776 -0.00013029 -0.00017307 -3.9872e-05 -7.0663e-08 -0.00014928

1790.4 -3.7451e-05 8.9191e-07 7.7283e-06 8.7925e-06 1.5474e-05 2.5419e-05 8.4338e-06 6.3428e-06 1.1861e-06 7.2458e-06 3.325e-05 3.0291e-05 1.7185e-05 5.5202e-05 5.6734e-05 4.2594e-05 1.4739e-05 1.2649e-05 2.7298e-06 1.3618e-05 2.2964e-05 9.8869e-06 -1.1761e-06 2.7392e-05 5.9255e-05 2.7308e-05 8.4495e-05 8.1779e-05 8.3931e-05 -4.161e-05 -2.7355e-05 1.4935e-05 0.00012309 0.00015368 0.00014609 6.2097e-05 -1.3629e-06 -3.1382e-05 -0.00012312 -3.8869e-05 2.9088e-05 2.0289e-05 -1.1283e-05 0.00019477 -1.0194e-05 9.7725e-05 8.6748e-05 -1.0281e-05 9.625e-05 3.4199e-05 4.5142e-05 8.944e-06 0.00013847 0.00010818 -7.8164e-05 0.00013375 0.00026976 -5.5717e-05 -8.8109e-05 -9.411e-05 5.4354e-05 -0.00018395

1797.4 1.3573e-05 9.9197e-06 3.596e-05 4.5685e-05 4.1986e-05 4.8037e-05 2.8539e-05 5.143e-05 4.9656e-05 5.1771e-05 7.9377e-05 9.6693e-05 8.8066e-05 0.00012138 5.691e-05 3.6761e-05 4.6672e-05 3.8334e-05 1.7784e-05 3.2988e-05 2.7017e-05 2.0589e-05 3.4201e-05 0.00011009 0.00011829 8.4594e-05 0.00014742 0.0001245 4.9902e-05 9.8184e-06 6.8044e-05 4.7941e-05 0.00013316 0.00012214 0.00010565 1.728e-05 -9.5516e-06 -3.845e-05 -8.532e-05 -8.6119e-06 8.4403e-05 9.5611e-05 7.5012e-05 0.00018105 2.5006e-05 0.00011379 0.00012565 -7.9564e-05 0.00018015 7.9583e-05 4.5278e-05 3.7925e-05 0.00015943 1.606e-06 7.9703e-05 0.00011374 5.7153e-05 5.7346e-05 4.99e-05 -4.7442e-05 0.00021177 -0.00011358

1804.5 9.2082e-05 5.5557e-05 8.6965e-05 7.7289e-05 7.7152e-05 6.5218e-05 7.5809e-05 9.638e-05 8.6944e-05 8.9101e-05 0.00013569 9.3173e-05 0.00011615 0.00012448 4.7793e-05 4.672e-05 5.9873e-05 6.2099e-05 4.7606e-05 3.7441e-05 3.3862e-05 2.3897e-05 3.3864e-05 0.00011936 0.00012028 0.00011132 0.00014224 0.0001135 -3.9546e-06 3.4991e-05 5.7881e-05 -3.2002e-05 5.3011e-05 0.0001575 0.00014455 4.284e-05 6.0849e-05 5.2635e-05 3.4385e-05 2.5499e-05 0.0001309 0.00013728 0.00014386 7.9556e-05 0.00010698 7.4679e-05 8.2181e-05 -2.1099e-05 0.00023382 3.7358e-05 5.7499e-05 -7.8916e-06 0.00013138 9.2358e-06 0.0001481 7.9763e-05 -0.00034446 4.1793e-05 -1.663e-05 -2.4058e-05 0.00018318 -0.00026129

1811.5 9.6819e-05 5.7626e-05 8.7143e-05 6.1924e-05 7.9136e-05 4.1405e-05 7.9104e-05 7.1561e-05 7.1738e-05 0.00010464 0.00012698 7.2463e-05 8.3029e-05 7.4027e-05 2.0786e-05 4.1091e-05 3.7472e-05 3.9474e-05 2.6629e-05 1.9109e-05 2.6145e-05 2.8676e-05 3.4383e-05 5.562e-05 7.2277e-05 0.00011426 8.7811e-05 6.0509e-05 -8.3851e-06 -1.3754e-05 -1.5095e-05 -5.4769e-05 3.9623e-05 0.00017478 0.00016881 0.00010667 0.00014301 0.00014827 9.6946e-05 7.986e-05 0.00011278 0.00011995 0.00018095 4.784e-05 0.00015484 5.8775e-05 9.0113e-05 5.9102e-05 0.00018176 6.0473e-05 -1.2878e-05 -2.0656e-05 0.00020849 -5.0569e-05 0.00010124 7.1021e-05 -0.0003207 6.8558e-05 -0.00015842 8.098e-05 4.4229e-05 -0.00033458

1818.6 5.3997e-05 4.3287e-05 4.831e-05 3.209e-05 5.3323e-05 2.0744e-05 4.2435e-05 2.4705e-05 3.4417e-05 8.8103e-05 6.7366e-05 3.6593e-05 4.4977e-05 3.6783e-05 -2.2501e-05 2.1653e-05 -1.3924e-05 1.6536e-06 -5.8992e-06 -1.4569e-05 1.8573e-05 2.9245e-05 4.342e-05 2.2192e-05 2.4254e-05 6.0359e-05 2.6316e-05 -1.5069e-05 -4.0619e-05 -5.9133e-05 -1.0632e-05 -7.2598e-05 -1.9409e-05 0.0001619 0.00016371 0.0001505 0.00021056 0.00022172 0.00014692 0.00017967 0.00013965 0.00016187 0.00020134 0.00010134 0.00014967 0.00013768 0.00013738 1.9188e-05 9.2329e-05 0.00019516 -4.0394e-05 2.6025e-05 0.00019579 3.9757e-05 8.104e-05 0.00014225 0.00013299 0.00022988 7.6123e-06 0.00010433 -2.4192e-05 -0.00024444

1825.6 7.204e-05 7.4064e-05 5.1338e-05 4.8639e-05 5.8055e-05 5.6442e-05 5.0779e-05 4.9294e-05 7.0185e-05 8.795e-05 5.7036e-05 4.7213e-05 4.2217e-05 6.3168e-05 -1.6895e-05 2.3507e-05 -4.931e-06 8.728e-06 1.6189e-05 1.5021e-05 2.9721e-05 3.3747e-05 5.3428e-05 3.0933e-05 1.7772e-05 1.1872e-05 -1.2796e-05 -4.6583e-05 4.9197e-05 9.5491e-05 0.00017362 7.7998e-05 -1.0076e-05 6.9671e-05 0.00013144 0.00016939 0.00022115 0.00021416 9.8297e-05 0.00016422 0.00013745 0.00016677 0.00016438 0.00011709 0.00016009 0.00018905 0.00015054 4.4824e-05 5.0307e-05 0.00025854 -7.0754e-05 7.2061e-05 8.3068e-05 0.00016592 8.8972e-05 8.342e-05 0.0003009 0.00026631 8.5265e-05 9.9426e-05 -0.00018526 -3.5088e-05

1832.6 8.4588e-05 8.9733e-05 6.3732e-05 4.4935e-05 5.1316e-05 6.8286e-05 4.5203e-05 7.9547e-05 8.5159e-05 7.6129e-05 7.3929e-05 4.596e-05 5.7804e-05 6.7113e-05 1.7431e-05 2.1831e-05 8.769e-06 2.8405e-05 3.7383e-05 3.9728e-05 4.6977e-05 3.1033e-05 4.0648e-05 3.9466e-05 1.4885e-05 -1.083e-05 -3.4064e-05 -1.932e-05 0.00010415 0.00017124 0.00028284 0.00016393 1.5587e-05 -2.6455e-05 7.5778e-05 0.00018032 0.00019905 0.0002039 8.6401e-05 0.00011565 1.8611e-05 7.1502e-05 7.0871e-05 0.00012752 0.00014519 0.00015894 0.00012829 7.3775e-05 0.00015051 0.00020238 2.1647e-05 7.9308e-05 9.2476e-05 0.00015194 0.00024614 1.1769e-05 -8.981e-05 8.468e-05 6.6237e-05 9.0228e-05 -0.00013809 6.3292e-05

1839.6 5.7958e-05 6.4249e-05 4.6405e-05 2.5047e-05 2.6832e-05 4.0704e-05 2.4634e-05 5.1728e-05 5.8004e-05 4.1473e-05 6.1286e-05 2.7571e-05 2.7643e-05 2.7288e-05 1.2857e-05 7.0664e-06 -1.0114e-05 2.2691e-05 2.1687e-05 3.2073e-05 3.6885e-05 1.9171e-06 9.4153e-06 9.9364e-06 -1.7266e-05 -3.5948e-05 -4.9927e-05 -4.3734e-05 8.797e-05 0.00011508 0.00016508 3.216e-05 -6.9344e-05 -3.262e-05 0.00010079 0.00017876 0.0001566 0.000154 0.00010918 6.9652e-05 -4.2198e-05 4.1245e-06 5.1126e-05 0.00013786 6.3618e-05 0.00011643 0.00011095 5.324e-05 6.9767e-05 1.5026e-05 0.00011225 0.00011271 0.00014178 3.0312e-05 0.00020952 5.4691e-05 7.6238e-05 8.8666e-05 0.00015688 -1.1283e-06 -2.1468e-06 -8.2802e-05

1846.6 5.9621e-05 4.7601e-05 3.3112e-05 3.0046e-05 4.0513e-05 2.5615e-05 2.7415e-05 2.6003e-05 3.7683e-05 2.7643e-05 5.7672e-05 1.3745e-05 1.4758e-05 8.5347e-06 1.1317e-05 3.2508e-05 -7.6744e-06 2.0223e-05 4.7778e-06 2.2394e-05 1.7983e-05 1.7478e-05 -4.9594e-06 -1.0508e-05 -2.8838e-05 -1.3235e-05 2.0526e-05 -3.8696e-06 6.7733e-05 4.4297e-05 7.2755e-05 -4.7809e-05 -2.2857e-06 0.00010247 0.0001464 0.00011287 9.4248e-05 8.7355e-05 8.2761e-05 5.2793e-05 3.0179e-06 9.1458e-05 9.3695e-05 0.00015259 -8.7104e-06 8.8921e-05 0.00011512 6.0617e-05 3.9727e-05 -8.8004e-05 4.5101e-05 1.0648e-05 0.00015201 -0.00013373 7.8129e-05 3.427e-05 0.00034176 6.7701e-06 8.3192e-05 -0.00024464 -5.2955e-05 -0.00013338

1853.5 8.2219e-05 6.2125e-05 5.2006e-05 5.8473e-05 7.6776e-05 4.025e-05 3.9741e-05 2.6383e-05 3.0217e-05 4.869e-05 6.8682e-05 3.185e-05 2.4605e-05 3.8593e-05 3.2181e-05 3.5755e-05 3.1251e-05 2.7409e-05 3.1197e-05 4.2985e-05 3.5961e-05 4.9524e-05 9.618e-06 3.7721e-05 3.1525e-05 0.00010382 0.00012675 0.0001169 0.000103 7.4067e-05 -1.5744e-05 0.00012937 0.00018827 0.00015043 9.1881e-05 3.8394e-05 3.2905e-05 2.7998e-05 1.8762e-05 6.1915e-05 6.7924e-05 0.00014154 0.00010949 0.00017773 6.501e-05 0.00014865 0.00019242 2.0427e-05 0.00015497 2.7363e-05 -4.2257e-05 -7.3447e-05 0.00013084 -1.7193e-05 0.00019106 0.00010598 0.0002909 -8.4555e-05 7.7712e-05 -0.00021941 -0.00010666 -0.00016782

1860.5 8.5152e-05 7.5403e-05 6.9373e-05 7.3086e-05 9.8938e-05 5.786e-05 4.1383e-05 4.0726e-05 2.1746e-05 5.5051e-05 4.4074e-05 8.8678e-06 4.7266e-05 5.702e-05 2.0773e-05 1.7698e-05 2.8767e-05 3.2216e-05 4.1883e-05 2.2457e-05 2.725e-05 1.6154e-05 1.6629e-05 5.0758e-05 5.9691e-05 0.00012153 0.00012064 0.00013207 -1.0438e-05 5.3004e-06 -7.4174e-05 0.00012915 0.00015792 0.0001012 1.3927e-05 -5.8059e-05 -1.6854e-05 -1.7999e-05 2.9725e-05 7.6005e-05 0.00013643 0.00014602 8.7132e-05 0.00012843 5.7614e-05 0.0001839 0.00017747 2.1666e-05 0.00012464 0.00012174 -0.00010524 -7.5362e-05 5.8659e-05 5.1665e-05 0.00014703 0.00019011 -2.0983e-05 -6.8944e-05 0.000111 -4.2837e-05 -0.00012493 -0.00026166

Fig. 3c.

0 1772.8 1767.2 1761.6 1756 1750.5 1745 1739.6 1734.2 1728.8 1723.5 1718.2 1712.9 1707.7 1702.5 1697.4 1692.3 1687.2 1682.2 1677.2 1672.2 1667.3 1662.4 1657.5 1652.7 1647.9 1643.1 1638.4 1633.7 1629 1624.3 1619.7 1615.2 1610.6 1606.1 1601.6 1597.1 1592.7 1588.3 1583.9 1579.5 1575.2 1570.9 1566.7 1562.4 1558.2 1554 1549.9 1545.7 1541.6 1537.5 1533.5 1529.5 1525.4 1521.5 1517.5 1513.6 1509.7 1505.8 1501.9 1498.1 1494.3 1490.5

1402.1 -7.4909e-06 2.6394e-06 4.9432e-05 4.5064e-05 6.677e-05 4.2413e-05 2.6249e-05 3.3307e-05 3.4245e-05 7.5432e-06 -2.8778e-05 -9.1528e-05 -4.0631e-05 1.4564e-05 4.3154e-06 2.6936e-05 8.8858e-06 6.1515e-06 3.6106e-05 2.9648e-05 -1.7169e-07 8.785e-06 -2.2906e-05 -0.00011921 -9.3934e-05 -6.3314e-05 -3.1964e-05 -2.9467e-05 -5.9428e-05 -5.4222e-05 -0.00011992 -6.8493e-05 -0.00015754 -0.00016035 -8.0702e-05 -7.2254e-05 -0.00013571 -6.9107e-05 -0.00010361 -0.00021196 -0.00014002 -0.00014392 -8.0822e-05 -0.00012997 -8.8807e-05 -2.2576e-05 7.6502e-06 -9.9597e-05 -4.8935e-05 0.00010301 0.00014576 -6.8644e-05 -9.3864e-06 0.00019137 -0.00027859 -0.00018709 -6.4286e-05 -0.00042918 -0.0001171 0.00018064 -0.00013559 -6.3351e-05

1409.6 5.1277e-05 4.5208e-05 7.2708e-05 6.7899e-05 7.4592e-05 5.63e-05 5.8726e-05 5.3862e-05 5.5946e-05 3.17e-05 -1.7046e-05 -6.3601e-05 -3.152e-05 5.9864e-06 1.7236e-05 5.1211e-05 5.2532e-05 3.9222e-05 5.6444e-05 4.1086e-05 2.6126e-05 2.6996e-05 -6.9396e-06 -6.6021e-05 -3.5598e-05 -3.5464e-05 -5.2633e-06 -9.1769e-06 -1.2939e-05 -2.4066e-05 -4.6951e-05 -8.3172e-05 -0.0001708 -0.00015812 -6.0288e-05 -5.2043e-05 -0.00011511 -0.00010134 -7.3146e-05 -0.00015691 -0.00011969 -0.00014738 -7.484e-05 -0.00015347 -7.0873e-05 1.0027e-05 -3.4911e-05 -6.5934e-05 -8.6137e-05 3.9336e-05 0.00010617 -4.0813e-05 -8.3637e-05 9.7108e-05 -0.00030388 -0.00022662 -0.00019744 -0.00030836 4.6459e-05 0.00012953 -0.00017147 5.3666e-06

1417.2 7.8514e-05 4.711e-05 4.4783e-05 3.966e-05 2.2662e-05 1.7966e-05 5.2136e-05 4.9929e-05 3.7129e-05 2.3776e-05 -3.135e-05 -7.7463e-05 -7.1942e-05 -6.5663e-05 1.0912e-05 6.5763e-05 8.0243e-05 6.4078e-05 4.97e-05 2.4418e-05 3.8096e-05 1.3871e-05 -2.1652e-07 2.2642e-05 3.4217e-05 1.2217e-05 3.7008e-05 -2.0565e-05 2.7083e-05 2.0607e-05 1.049e-05 -4.1017e-05 -9.1644e-05 -3.8509e-05 -4.3222e-05 -2.3219e-05 -6.3637e-05 -6.6904e-05 -1.8882e-05 -1.7382e-05 -4.2577e-05 -7.5107e-05 -2.857e-05 -0.00011451 -3.8891e-05 1.5991e-05 -3.3177e-05 -3.0097e-05 -6.0417e-05 -2.6481e-05 -5.5564e-06 4.6086e-05 -0.00011762 2.6823e-05 -0.00017991 -0.00011578 -0.00026556 3.0461e-05 0.00010795 -3.2394e-05 -0.00015719 5.9588e-05

1424.8 -9.9001e-08 -1.7863e-05 -4.0576e-05 -3.3247e-05 -4.489e-05 -4.7737e-05 -4.8465e-06 -1.3036e-05 -2.5746e-05 -2.9907e-05 -6.4861e-05 -0.00011504 -0.00013935 -0.00011809 -3.6183e-05 3.1932e-05 4.1778e-05 3.4335e-05 4.2775e-06 -8.4028e-06 1.1067e-05 -2.605e-05 -3.1576e-05 2.1584e-05 1.4364e-05 1.9887e-05 3.4809e-05 -3.5414e-05 -1.4402e-05 1.943e-05 3.5032e-05 3.5693e-05 -3.2175e-05 5.42e-05 -4.2e-05 -5.667e-05 -4.0077e-05 -3.8184e-05 -1.5583e-05 6.675e-05 -5.5644e-06 -1.4325e-05 -3.8162e-06 -7.2318e-05 -1.7736e-05 2.0571e-05 -1.6246e-05 3.2152e-05 1.4907e-05 2.9582e-05 -4.5821e-05 0.00012469 6.2471e-05 9.6316e-05 -1.9545e-06 1.323e-06 -9.0912e-05 0.00024929 3.2641e-05 -8.94e-05 -8.5095e-05 0.00010829

1432.4 -3.0757e-05 -2.1794e-05 -6.1639e-05 -4.8173e-05 -5.5986e-05 -6.2116e-05 -2.8172e-05 -3.892e-05 -4.0749e-05 -4.622e-05 -7.1941e-05 -9.904e-05 -0.00015969 -0.00012968 -6.3732e-05 1.0967e-05 1.8841e-05 1.3153e-05 -2.2224e-05 -1.7421e-05 -1.1417e-05 -3.5462e-05 -4.5485e-05 6.0372e-06 -3.0379e-05 -1.19e-05 -4.4693e-05 -0.00012335 -0.00011589 -4.2081e-05 2.8416e-05 3.0958e-05 8.0388e-05 9.5974e-05 -3.0557e-05 -7.682e-05 9.9602e-06 -5.0436e-05 -2.8839e-05 9.0882e-05 -1.3895e-05 2.4046e-05 -7.1343e-05 -7.072e-05 -2.9559e-05 4.8618e-05 3.531e-05 8.7826e-05 4.6337e-06 2.182e-05 3.6654e-05 8.3891e-05 6.1683e-05 8.0112e-05 6.8382e-05 6.2772e-05 -0.00013959 0.00022399 -5.6585e-05 5.7712e-05 0.00011499 0.00011924

1439.9 -3.7087e-07 2.907e-06 -2.6009e-05 -2.4927e-05 -2.7352e-05 -3.6491e-05 -2.1407e-05 -2.5657e-05 -1.9497e-05 -1.6583e-05 -5.4529e-05 -6.5555e-05 -0.0001471 -0.0001073 -5.4262e-05 -2.9212e-06 2.2153e-05 1.2173e-05 6.9762e-06 7.2163e-06 1.3349e-05 -8.3561e-06 -5.7701e-06 2.5317e-05 -5.5557e-06 -3.6018e-06 -3.265e-05 -0.00011447 -9.9589e-05 -9.0742e-05 -1.2332e-06 2.4803e-05 0.00011178 0.00010885 -2.5628e-06 1.4825e-05 3.5107e-05 3.6996e-05 8.9965e-06 7.2615e-05 -1.3728e-05 -2.9858e-05 -6.4068e-05 -7.0556e-05 -6.9225e-05 1.9819e-05 4.7351e-05 -5.0773e-05 -0.00016111 -6.5709e-05 2.2454e-05 5.7614e-05 -5.0712e-06 -5.3131e-05 7.5181e-06 4.5268e-05 -0.00045246 0.00021413 -7.5934e-05 0.00013594 0.00032747 8.7997e-05

1447.4 2.8029e-05 2.6004e-05 3.3761e-05 1.7862e-05 2.3532e-05 1.1849e-05 1.8352e-05 2.7778e-06 1.4896e-05 2.8231e-05 -3.2588e-06 -1.557e-05 -6.6449e-05 -4.4298e-05 -1.4683e-05 1.9118e-05 3.3849e-05 4.5254e-05 5.8863e-05 5.0597e-05 6.5695e-05 4.0361e-05 4.3729e-05 5.0451e-05 3.5808e-05 4.7594e-05 3.9849e-05 -9.2928e-06 -4.7565e-05 -2.8106e-05 -2.1991e-05 3.6835e-05 2.8971e-06 0.00011454 -7.3111e-06 2.4874e-05 -2.0127e-05 9.8991e-05 5.9978e-05 1.9416e-05 -2.0666e-05 -9.4431e-05 1.7974e-05 -6.6664e-06 -9.0009e-05 1.1414e-05 1.0832e-05 -0.00015152 -0.00021011 -6.9505e-05 2.2296e-05 3.8467e-05 -3.9712e-06 -0.00013847 -9.1954e-05 -0.00012971 -0.00028312 4.5851e-05 -0.00013454 3.3921e-05 0.0001672 -6.1554e-05

1455 3.4169e-05 5.1508e-05 6.9667e-05 5.8494e-05 4.8722e-05 4.4234e-05 5.2533e-05 3.7246e-05 5.1221e-05 5.4667e-05 4.1991e-05 3.4137e-05 -3.1826e-06 1.2104e-05 3.0182e-06 1.8832e-05 3.546e-05 6.7993e-05 6.3889e-05 5.448e-05 7.2898e-05 5.7434e-05 4.738e-05 6.1645e-05 2.613e-05 2.7539e-05 4.7209e-05 3.2173e-05 -3.2606e-05 1.6175e-06 -3.8629e-05 4.0269e-05 -4.6176e-05 5.4726e-05 -7.553e-06 -4.0021e-05 -3.8868e-05 8.9918e-05 7.3038e-05 -2.0732e-05 2.0611e-05 -2.8164e-05 3.1668e-05 3.9558e-05 -3.1252e-05 5.9201e-05 -6.9728e-06 -4.4398e-05 -3.4521e-05 3.7661e-05 7.7716e-05 3.2989e-05 -3.467e-05 -0.00015737 -0.00010354 -0.00016226 -0.00011923 -0.00013449 -0.00017669 -8.3894e-05 -5.3096e-05 -5.1392e-05

1462.5 4.4287e-05 6.5322e-05 6.1905e-05 7.3601e-05 4.5225e-05 3.833e-05 4.1442e-05 5.1323e-05 4.3554e-05 4.607e-05 3.0105e-05 3.169e-05 -2.9929e-06 2.0199e-05 -1.058e-06 -1.0514e-05 1.8908e-05 3.7092e-05 2.9764e-05 1.779e-05 1.3758e-05 1.5398e-05 1.3163e-05 3.1126e-05 1.4521e-05 -1.5897e-05 1.473e-05 -2.0827e-05 -6.3564e-05 -5.3289e-05 -0.00012161 6.445e-06 -2.4072e-05 3.5569e-05 8.4717e-05 1.78e-05 7.1921e-05 5.9002e-05 6.7328e-05 1.6672e-05 5.9531e-05 4.3446e-05 3.9987e-06 8.7318e-05 8.0373e-05 4.148e-05 -1.1481e-05 8.8078e-05 9.6758e-05 0.00015862 0.00011696 9.1502e-05 3.0638e-05 -5.7497e-05 6.8323e-06 -2.9577e-05 -0.00012577 -0.00011223 2.9804e-06 -4.4452e-05 -0.00010701 0.00015833

1470 2.4545e-05 2.2302e-05 2.8535e-05 4.7144e-05 1.8579e-05 1.5988e-05 7.4709e-06 2.1942e-05 1.9365e-06 1.446e-05 8.6438e-07 -2.4834e-06 -3.0021e-05 -1.5444e-05 -3.2028e-05 -3.8189e-05 -1.9717e-06 -1.7528e-06 6.1928e-06 -2.3265e-05 -2.9391e-05 -2.5956e-05 -7.1266e-06 -1.9994e-05 -1.7731e-05 -4.7923e-05 -4.1094e-05 -8.2136e-05 -0.00012856 -0.00010874 -0.00015029 1.4163e-05 -1.0245e-05 4.2458e-06 0.00014147 9.5274e-05 0.0001304 5.8138e-05 8.5108e-05 3.9167e-05 4.8417e-05 1.2092e-05 1.5073e-05 0.00017938 2.5332e-05 1.4592e-06 1.5115e-05 5.643e-05 2.3854e-05 0.00011762 9.5365e-05 0.00015038 0.00012747 1.1814e-06 8.2709e-05 6.4887e-05 0.00016968 1.9708e-05 8.4076e-05 8.5577e-05 7.97e-05 0.0001426

1477.5 -1.2905e-05 -2.3208e-05 4.9606e-06 -1.4738e-06 -2.6624e-06 1.1593e-05 -9.3322e-06 -1.4368e-05 -6.0969e-06 5.0703e-06 -2.6006e-06 -2.4239e-05 -3.8608e-05 -1.824e-05 -2.4714e-05 2.0664e-06 4.1351e-06 1.5084e-05 1.2582e-05 -1.7245e-05 -2.8728e-05 -2.1226e-05 -1.5479e-05 -1.3542e-05 -1.7742e-05 -1.0507e-05 -6.3938e-05 -8.5783e-05 -0.00011959 -6.9362e-05 -2.6517e-05 3.3972e-05 5.8397e-05 -2.6181e-05 6.4936e-05 7.4205e-06 5.2104e-05 2.8264e-05 3.81e-05 4.652e-05 4.0162e-05 3.6321e-05 3.3052e-05 0.00014084 -7.9076e-06 -1.9432e-06 8.1115e-05 -2.6097e-05 -4.9356e-05 6.2601e-05 0.00011923 0.00014397 0.00013767 -3.4452e-05 7.7778e-05 0.00012661 0.00041471 0.00010008 1.2732e-06 0.00015581 0.00022914 -9.8013e-05

1485 1.4721e-05 4.5941e-06 3.0182e-05 7.1364e-06 9.9613e-06 3.1632e-05 2.0833e-05 -1.0858e-05 1.9151e-05 3.1223e-05 3.8008e-05 1.0011e-05 -1.3935e-06 5.1388e-05 2.2131e-05 4.8545e-05 3.8137e-05 4.373e-05 3.4952e-05 2.8356e-05 1.7526e-06 1.1198e-05 3.5196e-06 2.6203e-06 4.6227e-06 5.3877e-05 3.6336e-06 -3.1776e-06 4.4375e-05 1.2381e-05 1.2571e-05 2.1304e-05 0.00013973 -3.3374e-05 -3.6424e-05 -0.00013337 -2.609e-05 -4.6051e-05 -5.0063e-05 -4.8971e-05 -4.0538e-05 -4.5068e-05 -7.2463e-05 -4.6048e-05 -3.3594e-05 -4.442e-05 3.3722e-05 1.3066e-05 1.0805e-05 5.7513e-05 0.00014786 0.00013498 4.7115e-05 -2.2699e-05 -1.8919e-05 0.00010746 0.00025677 0.00010469 -6.814e-05 0.0001865 0.00019541 -0.00015502

1492.5 -3.491e-06 -8.985e-06 1.1047e-05 -6.9504e-06 -2.1976e-05 4.4794e-06 -2.8623e-06 -4.7146e-05 -4.1012e-06 5.3637e-06 3.2184e-06 -1.3587e-05 -3.4892e-05 1.3459e-08 7.0357e-06 1.8577e-05 1.5426e-05 -6.9366e-06 -2.751e-06 -5.0948e-06 -1.1763e-05 -1.6144e-05 -1.2071e-05 -8.9311e-06 -1.3111e-05 7.2593e-06 1.6905e-05 8.2854e-06 6.3059e-05 2.8889e-05 -9.2966e-05 -5.0709e-05 2.4857e-05 -6.1185e-05 -2.2175e-05 -0.00013832 -3.473e-05 -6.6036e-05 -6.8155e-05 -0.00010259 -9.347e-05 -0.00012047 -0.00014881 -0.00016973 -0.00013008 -9.6515e-05 -5.2938e-07 5.9497e-05 0.00011785 1.4715e-05 0.00012078 8.3353e-05 1.4487e-05 -2.8821e-05 -8.6577e-05 -1.5767e-06 0.0001103 -8.5065e-05 -2.3711e-05 7.9615e-05 0.00011469 -8.1906e-06

1499.9 -3.3112e-05 -4.6637e-05 -5.8079e-06 -3.7665e-05 -5.1098e-05 -2.5342e-05 -3.6931e-05 -8.0408e-05 -3.9964e-05 -4.3349e-05 -5.4563e-05 -6.909e-05 -9.2511e-05 -8.2952e-05 -1.9118e-05 -3.0055e-05 -1.5149e-05 -5.4207e-05 -4.1125e-05 -2.9661e-05 -3.0892e-05 -3.4677e-05 -1.4178e-05 -4.6717e-05 -2.9425e-05 -4.5957e-05 -8.9476e-06 -3.5385e-05 -2.6595e-05 -1.5046e-05 -0.00014889 -0.00013569 -0.000183 -0.00012023 6.6033e-05 -3.0297e-05 8.3579e-06 4.0765e-05 -1.0323e-05 -3.8488e-06 3.1795e-05 -2.9097e-05 -0.00012075 1.1173e-06 -4.8271e-05 2.3935e-05 8.5482e-05 1.4777e-05 0.00015183 9.0798e-06 6.3744e-05 -1.5151e-05 4.2626e-06 -0.00013404 -2.0294e-06 -5.4867e-05 6.3963e-05 -0.00022311 8.5681e-05 0.00010722 0.00010866 0.00020692

1507.4 -5.9779e-06 -2.3829e-05 9.6375e-06 -2.1106e-06 -1.1728e-05 1.3111e-05 -1.53e-05 -4.1122e-05 -1.2094e-05 -2.6943e-05 -3.5673e-05 -3.4951e-05 -5.847e-05 -2.1714e-05 2.6714e-05 2.08e-05 1.8983e-05 1.3312e-05 1.4006e-05 3.3883e-05 1.8952e-05 2.8636e-05 4.3368e-05 -1.1775e-06 9.41e-06 8.7417e-06 1.6178e-05 -4.0807e-05 -4.383e-05 -4.9387e-05 -0.00013465 -0.00025333 -0.00022137 -4.2626e-05 0.00012693 9.2263e-05 0.00016586 0.00016797 0.00015005 7.8041e-05 0.00016338 0.00017553 7.4305e-05 0.00025218 9.3993e-05 6.7687e-05 8.3296e-05 -2.7087e-05 9.4473e-05 2.6662e-06 4.8229e-05 -1.4063e-05 6.5916e-05 -0.00013975 -1.1425e-05 -4.5862e-05 -2.3899e-05 -6.8308e-05 4.5562e-05 4.2966e-05 4.3144e-05 0.00021355

1514.8 -1.494e-05 -1.4367e-05 -9.9109e-06 -4.4193e-06 -5.8589e-06 1.5209e-05 -2.6069e-05 -1.8172e-05 9.3127e-06 -1.7147e-05 -8.8882e-06 -5.598e-06 -2.7631e-05 3.2905e-05 2.1607e-05 6.2967e-05 3.376e-05 5.1122e-05 3.7745e-05 5.7471e-05 4.163e-05 4.3841e-05 4.2225e-05 7.747e-05 2.7708e-05 5.3131e-05 8.5105e-06 -2.9194e-06 -1.9591e-05 -6.9623e-05 -7.9762e-05 -0.00019435 -0.00019649 6.1478e-05 8.958e-05 0.00017109 0.00024858 0.00019542 0.00019129 3.1487e-05 0.00014938 0.00025857 0.00022142 0.0002108 1.5891e-05 -6.5952e-05 -4.4015e-05 -6.0512e-05 -2.0384e-05 4.9306e-05 0.0001519 6.0399e-05 -2.7602e-05 -8.2969e-05 -3.6407e-05 -5.7044e-05 4.5206e-05 -0.00022321 6.6795e-05 -0.00011986 -3.2915e-05 5.9236e-05

1522.3 -3.1801e-05 -1.4432e-05 -3.2197e-05 -3.8481e-05 -1.8502e-05 -3.1752e-05 -4.671e-05 -4.5701e-05 -9.7402e-06 -3.4138e-05 -3.3393e-05 -6.6488e-05 -4.5018e-05 1.0521e-06 -2.3304e-05 2.1835e-05 6.5464e-06 -9.6627e-06 -5.857e-06 7.7965e-06 -1.0096e-05 -1.1638e-05 -3.0656e-05 -2.7007e-06 -3.7812e-05 -2.1009e-05 -4.2536e-05 -5.537e-05 -1.8997e-05 -3.0575e-05 -2.3447e-05 -4.1802e-06 -0.00011792 0.00012321 0.00011185 0.00016878 0.00016183 0.00017652 0.00010212 3.6167e-05 0.00018023 0.0001807 0.0001198 5.3041e-05 -2.493e-05 -0.0001218 3.819e-05 -1.4183e-05 5.6654e-05 9.4074e-05 0.00023245 0.00014789 -1.2864e-05 0.00013409 0.00010144 -7.2417e-06 0.00026229 -0.00024549 0.0001845 -6.8293e-05 2.4775e-05 5.2275e-05

1529.7 -2.6449e-05 -3.9786e-06 -3.0191e-05 -3.688e-05 -2.0854e-05 -3.6426e-05 -2.5367e-05 -5.4782e-05 -1.5609e-05 -4.3478e-05 -7.8015e-05 -0.00013713 -8.5288e-05 3.6392e-05 1.3307e-06 -1.0073e-07 3.3167e-05 -1.9199e-05 -1.2498e-05 -8.6159e-06 -1.6361e-05 -2.4614e-05 -3.5527e-05 -9.0923e-05 -4.0873e-05 -9.0407e-05 -0.00013392 -0.00018184 -0.00018639 -0.00015028 -3.2346e-05 0.00013609 0.00014208 0.00021852 0.00032453 0.00031923 0.00027857 0.00029192 0.00029661 0.00027162 0.00023374 8.8908e-05 -2.408e-05 1.0773e-05 0.00013802 -2.9934e-06 0.0001843 0.00014033 0.0001076 7.7035e-05 0.00015405 0.00021555 0.00010114 0.00026453 0.00022677 4.9009e-05 0.00034814 6.0408e-05 0.00022996 3.9797e-05 0.00022119 0.00012391

1537.1 -5.874e-05 -2.9871e-05 -3.0622e-05 -2.7975e-05 -3.3516e-05 -5.399e-06 -6.6811e-06 -4.1996e-05 -1.8822e-05 -5.74e-05 -0.00012619 -0.00017293 -9.5416e-05 0.00010918 9.1409e-05 5.7881e-05 0.00011159 4.6278e-05 5.6814e-05 6.4569e-05 6.0657e-05 4.5166e-05 6.2036e-05 -8.0769e-06 4.1732e-05 -4.5472e-05 -0.00013496 -0.00025164 -0.00040192 -0.00031969 -0.00021947 7.4411e-05 0.00028243 0.00037192 0.00059556 0.00064519 0.00062535 0.00055103 0.00055265 0.00041641 0.00018113 1.2336e-05 2.4566e-05 0.0002015 0.00027112 0.00016554 0.00024034 0.00023003 0.00011604 9.8436e-05 0.00018867 0.00026098 0.00019641 0.00027507 0.00040313 5.1816e-05 0.00058895 2.3106e-05 0.00023327 0.00031731 0.00028657 0.0002238

1544.5 -7.9996e-05 -6.1316e-05 -3.8859e-05 -2.481e-05 -4.8399e-05 -1.3193e-05 -2.2002e-05 -4.3144e-05 -3.3479e-05 -6.9534e-05 -0.00015791 -0.00021392 -8.753e-05 0.00016777 0.00015841 0.00011894 0.00015492 8.6937e-05 8.4372e-05 0.00010844 8.3062e-05 8.0637e-05 9.588e-05 0.00011915 9.776e-05 4.607e-05 -5.8971e-05 -0.00019685 -0.00035945 -0.00033208 -0.00030024 3.9009e-05 0.00025759 0.00042223 0.00060761 0.0007064 0.00066321 0.00047196 0.00026117 8.9819e-05 3.781e-05 1.9378e-05 0.00017865 0.00033661 0.00022776 0.00025431 0.00021091 0.00016405 0.00021778 0.00032794 0.00031439 0.00031387 0.0002227 0.00045778 0.00043638 0.00013897 0.00064674 5.5873e-05 0.00015306 0.00043579 0.00024841 0.00017614

1551.9 -4.603e-05 -5.2732e-05 -2.2668e-05 -1.8491e-05 -3.2459e-05 -2.2787e-05 -3.1181e-05 -3.8878e-05 -5.0045e-05 -8.82e-05 -0.00019563 -0.0002724 -0.00010925 0.00013476 0.00015913 0.00012462 0.00011187 2.6181e-05 1.3761e-05 8.8572e-06 -2.132e-05 -2.9949e-05 -1.4906e-05 2.1253e-05 -4.1425e-05 -5.7482e-05 -0.00013926 -0.00022288 -0.00031284 -0.00034065 -0.00026429 -3.8458e-05 0.00014187 0.00034554 0.00036994 0.0003795 0.0001921 -7.6573e-05 -0.00020565 -0.0001857 2.1681e-05 0.00014392 0.00028101 0.00028563 0.00018738 0.00023745 0.00026488 0.00028257 0.00045331 0.00048492 0.00038979 0.00034848 0.00021878 0.00054017 0.00037799 0.0002414 0.00053297 0.00023966 0.00023741 0.00020044 0.00027478 0.00024387

1559.3 -8.9473e-06 -1.227e-05 1.0369e-05 -1.0245e-05 -2.0935e-05 -4.9736e-06 -1.1683e-05 -2.8908e-05 -7.3376e-05 -0.00012791 -0.00023673 -0.00033953 -0.00016579 6.7924e-05 0.00013693 0.00010671 8.861e-05 -3.6027e-05 -5.633e-05 -7.9161e-05 -9.3105e-05 -0.00013865 -0.00013003 -0.00018696 -0.00023007 -0.000286 -0.00037086 -0.00046695 -0.00062704 -0.00065312 -0.00051633 -0.00021393 5.7047e-05 0.00025762 0.00021531 0.00013579 -6.4102e-05 -0.00015775 8.7572e-06 0.00017183 0.00025897 0.000289 0.00030594 0.00026887 0.00034599 0.00035028 0.00040125 0.00048169 0.00062 0.00047515 0.00050684 0.00036721 0.00038531 0.00042393 0.00030673 0.00030975 0.00052142 0.00039226 0.00031031 0.00021286 0.00031446 0.00037665

1566.7 -7.0715e-05 -6.7338e-05 -7.0917e-05 -7.0835e-05 -0.00011509 -7.3215e-05 -8.623e-05 -0.00013688 -0.00014764 -0.0002156 -0.00035485 -0.00047693 -0.00025621 4.2299e-05 0.00013564 0.00011652 9.359e-05 -2.8926e-05 -1.552e-05 -5.814e-05 -6.4985e-05 -0.00010644 -0.00010769 -0.00024598 -0.00023812 -0.00034606 -0.00047662 -0.00076208 -0.001096 -0.0011208 -0.00088496 -0.00047663 -0.00015813 2.5622e-05 9.5527e-05 0.00020454 0.00042961 0.000604 0.00070012 0.00062385 0.0003495 0.00029881 0.00031139 0.00037283 0.00052312 0.00046938 0.00052911 0.00046836 0.00062171 0.00051139 0.00056776 0.00036433 0.00041813 0.00027695 0.00023474 0.0003549 0.00042379 0.00046933 0.0002987 0.00024374 0.00033005 0.00027805

1574 -0.00016956 -0.00016786 -0.00018501 -0.00016307 -0.00018297 -0.00013809 -0.0001582 -0.00021133 -0.00020773 -0.00028186 -0.00049631 -0.0006422 -0.00037172 8.8204e-05 0.00023751 0.0002254 0.00015827 5.8025e-05 4.3509e-05 3.4675e-06 8.0971e-07 -1.4429e-05 -3.7541e-05 -0.00016256 -0.000131 -0.00026748 -0.00046527 -0.00086105 -0.0012252 -0.0012985 -0.001061 -0.00077755 -0.00055905 -0.00036415 2.8305e-05 0.00064339 0.0011421 0.0011862 0.00085088 0.00054805 0.00028031 0.00034419 0.00041855 0.00049268 0.00058292 0.00053681 0.00059822 0.00040682 0.00049165 0.00042212 0.00042587 0.0002941 0.00023079 0.00022253 0.00025087 0.00036423 0.00027561 0.00032691 0.00029453 0.00020807 0.00020965 0.00018646

1581.4 -9.2694e-05 -8.6205e-05 -9.5191e-05 -9.4668e-05 -8.2686e-05 -4.117e-05 -6.2509e-05 -9.5242e-05 -0.00011755 -0.00022838 -0.00055624 -0.00080588 -0.00049922 0.0001655 0.00045403 0.00041184 0.00027448 0.0001501 8.3281e-05 2.3649e-05 -6.8806e-06 -2.5616e-05 -8.6949e-05 -0.0002197 -0.00023684 -0.00040387 -0.00069096 -0.0011548 -0.0015325 -0.0017217 -0.0015225 -0.0013067 -0.00085964 -5.286e-05 0.00086993 0.0016518 0.0017417 0.0012793 0.00069529 0.00050588 0.00039848 0.00043663 0.00049202 0.00043669 0.00056486 0.00044384 0.00049445 0.00029428 0.00032218 0.00027489 0.00021818 0.00013508 0.0001649 0.00014487 0.00025683 0.00021275 6.746e-05 0.00021916 0.00026556 0.00021069 4.812e-06 0.00018174

1588.8 8.7657e-05 8.2852e-05 8.5133e-05 5.9506e-05 4.3405e-05 7.0432e-05 4.4856e-05 1.9943e-06 -4.6785e-05 -0.00023592 -0.00072456 -0.0011755 -0.00082359 0.00019792 0.00072713 0.00062046 0.00044523 0.00021528 0.00010327 2.2829e-05 -3.8042e-05 -9.9301e-05 -0.00016061 -0.00036489 -0.00046935 -0.00073439 -0.0011716 -0.0019772 -0.0027592 -0.0033635 -0.0031782 -0.0022629 -0.00087295 0.00099394 0.0022741 0.0027737 0.0023772 0.0018066 0.0013429 0.0011599 0.00089441 0.00072948 0.00067788 0.0005284 0.00058267 0.00047145 0.00044001 0.00044911 0.00031174 0.00034059 0.00027657 0.00028217 0.00031402 0.00012271 0.00032866 0.00023406 0.00035762 0.00038256 0.00031374 0.00030558 6.1132e-05 0.00038208

1596.1 0.00012529 8.7658e-05 0.0001206 8.2497e-05 3.1612e-05 4.9343e-05 1.947e-05 -7.8938e-05 -0.00013371 -0.00043644 -0.001115 -0.0018785 -0.0014396 0.00010907 0.0010753 0.00094761 0.00069907 0.00033412 0.00021561 9.4599e-05 3.5858e-06 -9.6263e-05 -0.00013777 -0.00042591 -0.00058363 -0.00092257 -0.0015038 -0.0028267 -0.0041898 -0.005377 -0.0053321 -0.0037304 -0.0017118 0.00031929 0.0015504 0.0024767 0.0028186 0.0030416 0.0028849 0.0026976 0.0022907 0.0018766 0.0015296 0.0012227 0.0010424 0.0008359 0.00068787 0.00078255 0.00060918 0.00047083 0.00045496 0.00061627 0.00046059 0.00030557 0.00032959 0.00035823 0.00048101 0.00063415 0.00020051 0.0003801 0.00027658 0.00047746

1603.4 1.5197e-05 -1.4285e-05 2.5264e-05 8.9129e-08 -6.1077e-05 -5.4843e-06 -3.339e-05 -0.00015232 -0.00019922 -0.00057349 -0.001454 -0.0025813 -0.0021135 -5.4095e-05 0.0013992 0.0013354 0.0009939 0.00049193 0.00035241 0.00016546 4.973e-05 -0.00010404 -0.00016718 -0.00059627 -0.00078936 -0.0012752 -0.0020082 -0.0037334 -0.005582 -0.0073849 -0.0077426 -0.006683 -0.0052117 -0.002917 -0.00022443 0.003086 0.0048202 0.0054678 0.0050948 0.0045672 0.0038768 0.0032609 0.0026076 0.0022011 0.0017216 0.0013189 0.0010115 0.00097336 0.00066936 0.00045507 0.00049334 0.00070021 0.00050536 0.00042641 0.00030887 0.00044207 0.00045726 0.00052133 0.00012616 0.00029152 0.0002313 0.00028732

1610.7 2.7032e-05 2.3139e-05 5.6472e-05 4.8276e-05 1.1183e-05 7.006e-05 6.5089e-05 -5.6968e-05 -8.2392e-05 -0.00044768 -0.0014567 -0.0027163 -0.002305 -9.9372e-05 0.0015099 0.0015125 0.0011332 0.0005377 0.00035249 0.00010928 -3.8663e-05 -0.00022027 -0.00034453 -0.00096094 -0.0013105 -0.0021394 -0.0034939 -0.0061941 -0.0091868 -0.012021 -0.012471 -0.010524 -0.0070154 -0.0016036 0.0034692 0.0076733 0.008722 0.0082313 0.0069337 0.0057008 0.004407 0.0036493 0.0028762 0.0024767 0.0019344 0.0014951 0.0012267 0.00098827 0.00066716 0.00054056 0.00049322 0.00061989 0.00044248 0.00037699 0.0002724 0.00045786 0.00024321 0.00023469 0.00026213 0.00024702 0.00017332 6.4521e-06

1618 0.00019546 0.00020094 0.00022962 0.0002128 0.00018447 0.00021547 0.00020807 8.4954e-05 6.536e-05 -0.0002761 -0.0012081 -0.0022517 -0.0018477 4.3222e-05 0.0013243 0.0013255 0.0010217 0.00043298 0.00024826 -2.9841e-05 -0.00018765 -0.00039682 -0.00058378 -0.0014075 -0.0019563 -0.0032304 -0.0055611 -0.010248 -0.015179 -0.018567 -0.016415 -0.0087874 -0.0010545 0.0060637 0.00993 0.011634 0.010768 0.0090722 0.0071612 0.0056358 0.004067 0.0032208 0.0024689 0.0020661 0.0016011 0.001208 0.0011608 0.00089608 0.00078884 0.00070545 0.00054643 0.00052617 0.00047234 0.00032302 0.00019083 0.00039429 0.00018894 7.4522e-05 0.00038227 0.00018361 0.00019338 -5.7107e-05

1625.3 0.00028513 0.00028664 0.00029925 0.00025053 0.00019842 0.0002257 0.00020019 8.4125e-05 4.1484e-05 -0.00026462 -0.0010318 -0.0017031 -0.0011745 0.00017132 0.00097993 0.00097823 0.000771 0.00030946 0.00018637 -6.728e-05 -0.00019703 -0.00043355 -0.00070297 -0.0016816 -0.002405 -0.0039954 -0.0069693 -0.012676 -0.01761 -0.018638 -0.012738 -0.002025 0.0053812 0.0099411 0.010998 0.010633 0.0091254 0.007385 0.0057105 0.0045017 0.003306 0.0026539 0.002057 0.0017141 0.0011922 0.00084896 0.0009251 0.00076733 0.00063942 0.0006166 0.00052646 0.00045056 0.00056684 0.00041469 8.3788e-05 0.00033169 0.00033357 0.00016913 0.00030213 0.00011391 0.00011003 6.5681e-05

1632.6 8.1286e-05 8.7268e-05 9.1861e-05 4.9787e-05 1.0562e-05 4.5106e-05 2.1644e-05 -7.3268e-05 -9.9534e-05 -0.0003536 -0.00087927 -0.0013051 -0.00078571 0.00013561 0.00060762 0.00061736 0.00049003 0.00020515 0.00012692 -1.2402e-05 -0.00010389 -0.00029886 -0.00056633 -0.0014524 -0.0021848 -0.0035562 -0.005855 -0.0092709 -0.01138 -0.010366 -0.0056142 0.0011015 0.0047963 0.0065746 0.0065475 0.0060717 0.005143 0.004174 0.0032783 0.0025589 0.002034 0.0017581 0.0014207 0.001169 0.00085553 0.0006003 0.00056865 0.00053886 0.00029844 0.00039572 0.00037452 0.00023111 0.00041819 0.00028304 -8.6939e-05 0.00024274 0.00020906 0.00010433 0.00010594 7.2283e-05 -2.7522e-05 0.00019707

1639.9 -0.00018558 -0.0001484 -0.00013425 -0.00014268 -0.0001499 -0.00010011 -0.00010279 -0.00016237 -0.00015004 -0.00030744 -0.00059928 -0.00090987 -0.00060754 -1.7295e-05 0.00030453 0.00032947 0.00024747 9.3882e-05 6.3599e-05 3.3701e-05 -3.703e-05 -0.00015745 -0.00025738 -0.00070609 -0.0010634 -0.0015895 -0.0022313 -0.0028889 -0.0035686 -0.003397 -0.0018413 0.00028198 0.0011645 0.0017183 0.0018743 0.0018328 0.0016529 0.0013504 0.0011292 0.00075469 0.00067246 0.00061104 0.000495 0.00036956 0.00030073 0.00029892 0.0002037 0.00017108 7.8527e-05 0.00015412 0.00017408 4.9287e-05 0.00018145 -0.00012423 -0.00022116 7.9228e-05 1.5461e-05 -3.2017e-05 -1.5628e-05 0.000143 -3.5811e-05 6.5632e-05

1647.2 -0.00013232 -9.6802e-05 -7.831e-05 -6.495e-05 -7.0032e-05 -2.8006e-05 -3.2622e-05 -7.0648e-05 -4.1858e-05 -0.0001251 -0.00028884 -0.00051648 -0.00041186 -6.8222e-05 0.00014977 0.00015045 0.00011241 5.1309e-05 7.1988e-05 5.7964e-05 1.858e-05 -2.1161e-05 1.1711e-05 -1.2765e-05 5.1596e-05 8.698e-05 4.4804e-05 -0.00035469 -0.0010166 -0.0011564 -0.00081731 -0.00052529 -0.00041272 -0.00016812 0.00015129 0.00017401 0.0002802 0.0001972 0.00022375 4.527e-05 4.4792e-05 2.2359e-06 -3.0708e-05 -5.9427e-05 -7.1718e-05 1.9075e-05 3.8603e-05 1.5735e-06 9.4224e-05 8.8615e-05 7.5718e-05 -4.1927e-06 4.7149e-05 -9.8103e-05 -8.0259e-05 2.5531e-05 0.00023109 2.1723e-05 0.00019552 0.00033969 9.3465e-05 5.8786e-05

1654.4 8.6703e-05 8.3775e-05 7.4831e-05 6.0136e-05 5.904e-05 5.2094e-05 3.7481e-05 3.8892e-06 5.317e-06 -5.3136e-05 -0.0001919 -0.00035065 -0.00030646 -0.00012998 1.8587e-05 3.3112e-05 2.716e-05 9.9392e-05 0.00012254 8.1043e-05 6.1202e-05 6.6084e-05 8.6647e-05 7.0096e-05 0.00012606 -2.2613e-05 -0.0004117 -0.00093031 -0.0010567 -0.00094896 -0.00072027 -0.00035221 -0.00010007 9.4973e-05 0.00024685 0.00027316 0.00033075 0.00026098 0.00020158 0.00015557 5.8271e-05 5.2597e-05 3.8859e-05 -4.8026e-05 -8.9176e-05 2.4762e-05 2.5125e-05 8.3588e-05 0.00026108 8.8811e-05 4.9567e-05 0.00013701 6.017e-05 0.00013148 3.5493e-05 0.0001689 0.00032781 0.00015059 0.00032623 0.00043256 0.0001829 0.00016416

1661.7 0.00014765 0.00011632 0.0001104 7.9591e-05 6.0121e-05 3.8725e-05 1.8516e-05 -1.5818e-05 -2.401e-05 -8.4827e-05 -0.00022967 -0.00037166 -0.0004099 -0.000288 -0.00012652 -1.0219e-05 6.8834e-05 0.00021851 0.00019074 0.00012285 4.9549e-05 9.7232e-07 -5.5002e-05 -0.00020524 -0.00033974 -0.00063903 -0.00093518 -0.0011203 -0.0010372 -0.0011189 -0.00075887 -0.00016436 0.0001465 0.00034121 0.0004292 0.0005248 0.00051776 0.00042465 0.00026879 0.0002692 0.00015488 0.00020677 0.00016562 5.2709e-05 -1.3996e-06 0.00017546 0.00012475 0.00016381 0.00027554 0.00014526 7.475e-05 0.00025221 0.00011301 0.00020798 9.0124e-05 0.00018711 0.00033104 0.00021767 0.00023703 0.0003955 0.0001133 2.7241e-05

1668.9 3.262e-05 2.5283e-05 1.9984e-05 2.6141e-05 -1.8391e-06 4.3082e-06 -1.3134e-05 -1.6818e-05 -1.6766e-05 -6.7064e-05 -0.00019122 -0.00035597 -0.00043292 -0.0003219 -6.5941e-05 0.00022412 0.00033272 0.00034683 0.00020074 -7.9169e-06 -0.00015633 -0.00019475 -0.0001992 -0.00029893 -0.00039222 -0.00049065 -0.00044039 -0.00052073 -0.00067834 -0.00099171 -0.00079244 -0.00034936 -0.00012949 0.00013605 0.00027965 0.00047832 0.00047071 0.00037187 0.00024144 0.00019364 0.00017219 0.00016251 0.00016022 0.00012071 8.444e-05 0.00015905 9.5692e-05 6.1325e-05 5.719e-05 0.00014397 1.7735e-05 0.00016515 5.884e-05 0.00015334 -1.8362e-05 4.2596e-05 0.00025375 0.00019675 5.8104e-05 0.00017668 3.7524e-05 -9.9085e-05

1676.1 -5.4059e-05 -2.7401e-05 -4.1552e-05 -2.5255e-05 -2.126e-05 -8.0922e-06 -1.2104e-05 4.3605e-06 9.8969e-06 -1.1063e-05 -8.1784e-05 -0.00026875 -0.00027899 -1.9133e-05 0.00037927 0.00060027 0.00047441 6.2699e-05 -0.00025297 -0.00039919 -0.00034482 -0.00022226 -0.00015164 -0.00015111 -3.7689e-05 4.419e-05 0.00013053 -0.0001469 -0.0004103 -0.00074528 -0.00085319 -0.00076477 -0.00051471 -7.667e-05 0.00012235 0.00035378 0.00039123 0.00037877 0.00025518 0.00023256 0.00016746 8.3504e-05 0.0001142 0.00013401 -5.3562e-06 -3.4539e-05 -3.6635e-05 -6.7355e-05 -3.8353e-05 -3.667e-05 -4.9935e-05 -2.4537e-05 -1.8469e-05 3.6798e-05 -0.00014869 -7.6044e-05 0.00021623 3.5388e-05 -0.00013551 7.3887e-05 -3.3448e-05 5.8588e-05

1683.4 -4.9322e-05 -3.0215e-05 -3.5929e-05 -3.4631e-05 -2.9678e-05 -2.3405e-05 -3.6945e-05 -2.7692e-05 -1.8519e-05 -3.8071e-05 -0.00012631 -0.00031046 -0.00024763 0.00015999 0.00055873 0.00032561 -0.00014887 -0.00052884 -0.00048105 -0.00023277 -7.4233e-05 -4.3453e-06 2.6295e-05 0.00010717 0.00028061 0.00021942 0.00015878 -0.00020241 -0.0004815 -0.00088389 -0.0011102 -0.00097532 -0.00055423 -0.00013354 0.00017561 0.00038779 0.00046301 0.00049275 0.00029146 0.00027267 0.00014481 7.2689e-06 6.0638e-05 5.7147e-05 -7.9225e-05 -0.00011767 -0.00012185 -0.00012498 -4.4472e-05 -0.00015244 -1.2481e-05 -3.8145e-06 9.5321e-06 -1.9569e-05 -7.416e-05 -3.0104e-05 0.00022603 1.8371e-05 -3.0464e-05 0.00014327 -3.5647e-05 0.00011112

1690.6 3.0757e-05 2.7499e-05 2.9002e-05 3.2058e-05 2.3646e-05 1.4252e-05 -3.4721e-06 -1.6316e-05 -1.6667e-05 -7.9892e-05 -0.0002366 -0.00055287 -0.00071661 -0.00057075 -0.00057125 -0.00069509 -0.00044189 0.00018402 0.00048322 0.00057355 0.00045576 0.00034864 0.00034029 0.00033908 0.00035307 0.00011937 -2.9039e-05 -0.00034466 -0.00077924 -0.001208 -0.0012974 -0.001006 -0.00052294 -3.5827e-05 0.00032148 0.00060115 0.00069026 0.00067291 0.00042686 0.0003785 0.00022264 7.3016e-05 7.5568e-05 0.00013032 -9.6623e-06 -3.5238e-08 -5.2781e-05 -6.1368e-05 7.2553e-05 -7.3271e-05 4.6113e-05 0.00025646 6.528e-05 0.0001533 4.4359e-05 0.00013122 0.00016758 0.00015999 0.00016488 0.00034705 6.621e-05 0.00011501

1697.8 6.8635e-05 6.6453e-05 7.0259e-05 7.6316e-05 8.8258e-05 7.0442e-05 6.8466e-05 4.6033e-05 4.426e-05 -6.8465e-05 -0.0003973 -0.0011121 -0.0021904 -0.0028254 -0.0020811 -0.00041259 0.00088412 0.0016526 0.0015389 0.0012186 0.00088674 0.00066564 0.00059132 0.00037038 0.00026402 6.7148e-05 -7.9136e-05 -0.00046006 -0.00098365 -0.001426 -0.0014022 -0.0011295 -0.00061511 -7.7179e-05 0.00033494 0.00078653 0.00087996 0.0008175 0.00060219 0.00050131 0.000397 0.00026461 0.00019239 0.00030401 0.00012518 0.00011534 6.6044e-05 5.2626e-05 0.00016639 6.6354e-05 9.8228e-05 0.00027427 6.7364e-05 0.00017834 6.7596e-05 0.00015205 7.9155e-05 9.9758e-05 0.00019488 0.00036747 9.6575e-05 6.671e-05

1704.9 7.1627e-05 8.0276e-05 9.8872e-05 9.3311e-05 0.00010607 0.00011314 0.000125 0.00010097 9.2232e-05 -4.4538e-05 -0.00066371 -0.002194 -0.0042541 -0.0042578 -0.0014316 0.0010361 0.0020206 0.0021901 0.0018161 0.0013707 0.0010142 0.00073068 0.00056714 0.0003197 0.00022107 0.00010671 -2.592e-05 -0.00050622 -0.00095524 -0.001294 -0.0012496 -0.0011379 -0.00054171 -6.8995e-05 0.00031431 0.00074703 0.00085387 0.00083832 0.00062252 0.00054162 0.00042452 0.00035802 0.00030463 0.00035731 0.00023706 0.00018272 0.00016503 0.00015951 0.00025024 0.00016011 0.00017222 0.00023174 4.6976e-05 -3.2745e-05 0.00011024 8.0098e-05 4.6509e-05 -4.8695e-05 0.00017081 0.00012619 2.9943e-06 8.7321e-06

1712.1 8.3768e-05 8.5587e-05 0.00011063 0.00010901 9.4034e-05 0.0001194 0.00012865 9.2714e-05 3.6517e-05 -0.00020627 -0.0012234 -0.0030976 -0.004139 -0.0027228 2.8685e-05 0.0014722 0.0018371 0.0017442 0.001391 0.0010267 0.00074838 0.00048654 0.0003474 0.00024377 0.00020893 8.4376e-05 -5.5511e-05 -0.00045947 -0.00078038 -0.00094463 -0.00093923 -0.00076392 -0.00031131 4.5678e-05 0.00026767 0.00050385 0.00065395 0.0006668 0.00048438 0.00046367 0.00034823 0.00028904 0.00027112 0.00026416 0.00025393 0.00023002 0.00016773 0.00023967 0.00018727 0.00019494 0.00013324 0.00015712 5.6243e-05 -3.0276e-05 3.5004e-05 1.6867e-05 0.00024104 -5.6861e-05 0.00012941 -0.0001103 -0.00010309 0.000111

1719.3 7.1416e-05 5.4777e-05 6.6935e-05 8.0395e-05 5.3643e-05 5.3717e-05 3.9165e-05 -3.089e-05 -0.00016388 -0.00053304 -0.0013957 -0.0022639 -0.0020071 -0.00090745 0.00042955 0.001013 0.0010646 0.00092068 0.00071287 0.00048268 0.00030281 0.00018547 0.00014863 0.00012917 0.00011506 -4.6843e-06 -0.00013498 -0.00037493 -0.00064827 -0.00074059 -0.00071247 -0.00033267 -8.8692e-05 0.00015573 0.00015801 0.00032638 0.00048226 0.0004684 0.00035095 0.00032765 0.00024125 0.00019221 0.00017865 0.00014692 0.00013251 0.00017607 8.5815e-05 0.00023179 8.5915e-05 0.00011073 -6.5306e-06 6.7969e-05 -4.6503e-05 2.4498e-05 -7.1839e-05 -9.5622e-05 0.00040133 6.2275e-05 -3.2762e-05 -0.0001038 -2.4126e-05 -0.00010202

1726.4 5.3542e-05 5.1322e-05 5.0721e-05 4.9461e-05 1.2869e-05 -1.1289e-05 -4.9686e-05 -0.0001467 -0.0002625 -0.00040881 -0.00060955 -0.00073615 -0.00057076 -0.00014476 0.00031631 0.00046111 0.00041257 0.00029391 0.0001921 7.4185e-05 1.6723e-05 4.5703e-05 5.7607e-05 4.2259e-05 1.5648e-05 -2.2129e-05 -0.00010252 -0.00025692 -0.00048786 -0.00063774 -0.0004625 -0.00010927 3.1063e-05 0.00013167 0.00011955 0.00028061 0.00037262 0.00033094 0.00026889 0.00019998 0.0001265 6.4915e-05 0.00012175 3.2959e-05 -3.1806e-05 6.5887e-05 -1.9118e-05 5.7124e-05 3.8931e-05 -5.9981e-05 1.5024e-06 5.7511e-06 -9.2274e-05 -5.4595e-05 -0.00016996 -0.00012805 -9.534e-05 0.00017249 -0.00018143 -3.6647e-05 0.00010317 -0.00013041

1733.6 8.949e-05 0.00011671 0.00010359 8.4066e-05 5.3237e-05 2.5912e-05 9.4137e-07 -1.7035e-05 -6.8317e-06 7.805e-05 5.9364e-05 -2.9928e-05 8.4876e-06 0.00013563 0.00021514 0.00018664 0.00013232 4.7589e-05 2.0703e-05 3.4747e-06 1.4098e-05 7.7337e-05 6.9861e-05 7.2788e-05 5.7458e-05 6.0298e-05 4.1427e-06 -0.00012572 -0.00022791 -0.00036181 -0.00015523 1.3026e-05 8.7122e-05 0.00012573 0.00017842 0.00026193 0.00026065 0.00020224 0.0001655 0.00011459 7.6722e-05 4.9109e-05 9.1106e-05 -3.3336e-05 -0.00011586 -4.6416e-06 -9.4132e-05 -6.6369e-05 -2.3672e-05 -0.00014743 -1.8427e-06 -1.6529e-05 -8.8067e-05 -5.7079e-05 -4.8204e-05 -0.00012133 -0.00030528 0.00013412 -0.00010837 -0.00018095 0.00021912 0.00011588

1740.7 9.7607e-05 0.00011031 0.00010396 8.4317e-05 7.466e-05 7.1428e-05 9.2782e-05 0.00014418 0.00017983 0.00020418 0.0001359 9.2684e-05 0.0001232 0.0001322 0.0001221 4.8454e-05 4.6535e-05 8.6491e-06 4.0383e-05 6.3932e-05 9.4452e-05 0.00010434 7.358e-05 0.00010321 0.00014161 9.4138e-05 2.0213e-05 2.3097e-05 -7.1979e-06 -0.00011282 1.6304e-05 7.8716e-05 0.00016439 0.0002077 0.00015822 0.00010003 6.5653e-05 7.7317e-05 4.1068e-05 -1.7697e-06 1.2258e-05 9.894e-06 -3.9556e-06 -5.9434e-05 -0.00012921 -3.3827e-05 -0.00011229 2.44e-05 4.0488e-05 -1.1428e-05 -3.2979e-05 2.3349e-05 -0.00011279 8.2372e-05 0.00017163 -0.00010037 -0.00016007 -7.9932e-06 -9.5837e-05 -0.00020585 0.00014237 0.00015984

1747.8 6.2774e-05 5.1293e-05 7.7132e-05 5.0148e-05 7.5712e-05 7.8633e-05 0.00010742 0.00011663 0.00011587 7.0857e-05 1.856e-05 3.6364e-05 3.8787e-05 4.1242e-05 3.3475e-05 5.2437e-06 1.1452e-05 3.935e-05 8.6675e-05 8.9485e-05 0.00010271 8.6273e-05 6.1853e-05 0.00010906 0.00015623 7.9366e-05 3.2759e-05 7.656e-05 5.7419e-06 -5.7121e-05 1.5735e-05 8.7429e-05 0.00020925 0.0002667 0.00012478 -1.5941e-05 -2.4565e-05 -4.4029e-06 -2.4293e-05 -3.9139e-05 1.816e-05 -7.9438e-06 -5.7081e-05 -6.42e-05 -2.6111e-05 -9.7697e-05 -2.5109e-05 6.3119e-05 0.00018483 9.7982e-05 3.2206e-05 6.1962e-05 -7.7462e-05 0.00014815 0.00018014 -3.2603e-05 -0.00030426 -2.8173e-05 -5.1554e-05 6.3724e-05 3.5808e-05 2.7989e-05

1755 3.8495e-05 3.9705e-05 6.5731e-05 4.1663e-05 7.4225e-05 6.9296e-05 6.1677e-05 5.3426e-05 2.4484e-05 -2.5475e-05 -2.2904e-05 1.5018e-06 -3.0344e-05 1.0438e-05 2.318e-05 2.4526e-05 3.5831e-05 0.00011255 0.00010682 8.4336e-05 7.9962e-05 9.5307e-05 5.8114e-05 0.0001007 0.00010601 4.7756e-05 5.8831e-05 4.8877e-05 -2.6541e-05 -5.4583e-05 3.9119e-05 9.7244e-05 0.00012921 0.00012742 2.1096e-05 -6.9779e-06 -1.0476e-05 5.7094e-06 1.3338e-05 -1.825e-06 5.7137e-05 2.2425e-05 -2.7289e-05 1.347e-08 0.00011362 -0.00010687 2.5091e-05 -3.2055e-05 0.00013996 0.00010923 0.00010378 7.1277e-05 2.4902e-05 3.7594e-05 5.3505e-05 -9.0221e-05 -0.00013693 -8.7133e-05 -1.51e-05 0.00023735 3.3498e-07 -9.3896e-05

1762.1 6.5623e-05 7.1689e-05 6.8043e-05 5.5687e-05 8.1809e-05 6.9409e-05 5.6801e-05 3.8935e-05 6.3641e-06 5.0908e-06 6.5919e-06 1.8678e-05 -3.4292e-06 2.4154e-05 3.2953e-05 0.00010395 0.00010174 0.00014191 0.000108 9.0638e-05 7.8123e-05 9.156e-05 6.0184e-05 6.6126e-05 4.0873e-05 2.9564e-05 5.3006e-05 4.2157e-05 3.681e-05 2.8629e-05 0.0001378 8.6772e-05 -6.3568e-05 -9.4095e-05 -3.2942e-05 -6.235e-07 1.7321e-05 5.8713e-05 6.7261e-05 2.5253e-05 9.6858e-05 6.3594e-05 2.9896e-05 1.0812e-05 9.2845e-05 -1.9942e-06 4.4593e-05 -9.2704e-05 0.00016354 7.7765e-05 0.00012035 -2.2355e-05 0.00013306 -1.1923e-05 -0.00010635 -0.00016386 -0.00021166 -9.6382e-05 -1.8594e-05 0.00024019 -4.5128e-05 -0.00010478

1769.2 7.7284e-05 9.9259e-05 9.7267e-05 7.4369e-05 9.4696e-05 8.5721e-05 6.7047e-05 5.119e-05 3.9568e-05 6.1231e-05 4.9252e-05 7.4989e-05 2.8985e-05 1.801e-05 6.0742e-05 0.00015872 0.00013885 0.00011625 0.00010914 8.0435e-05 7.6326e-05 5.8337e-05 4.5477e-05 3.7261e-05 3.4311e-05 6.2385e-05 6.8966e-05 0.00010002 0.00015149 0.00016092 0.00015985 -2.5447e-05 -9.5887e-05 -0.00013597 -1.9851e-05 -1.4323e-05 1.2456e-05 3.7003e-05 4.9309e-05 4.9815e-05 8.1886e-05 9.5872e-05 6.976e-05 -1.263e-05 -6.3426e-06 9.2192e-05 7.7097e-05 -1.2152e-05 0.00023083 6.8466e-05 0.00010267 -8.3647e-07 0.00013718 9.0526e-05 -8.7103e-05 -0.00017795 -0.00023853 -8.0287e-05 2.6245e-05 0.00010706 3.1504e-05 -5.5285e-05

1776.3 5.2692e-05 8.4025e-05 8.5467e-05 7.133e-05 7.7427e-05 6.1592e-05 5.3477e-05 4.1411e-05 4.4441e-05 5.8038e-05 5.7639e-05 6.4459e-05 2.8792e-05 9.2011e-06 5.0109e-05 0.00012033 9.0554e-05 5.793e-05 7.0642e-05 3.1935e-05 2.858e-05 9.8109e-06 2.598e-06 1.572e-05 2.1557e-05 4.9316e-05 6.034e-05 0.00010829 0.00013126 0.00012464 6.1717e-05 -0.00015641 -9.4786e-05 -0.00010358 -2.9856e-05 -3.4217e-05 2.5464e-05 -4.099e-06 5.6999e-07 0.0001015 1.8991e-05 2.8774e-05 2.3167e-05 -8.1836e-06 1.1815e-05 -2.5138e-05 5.3778e-05 1.4857e-05 6.403e-05 -2.3558e-05 1.0259e-05 -4.9128e-05 -6.281e-06 -1.1099e-05 -0.0001795 -6.8441e-05 -9.9768e-05 3.6557e-05 0.00012798 -2.2339e-05 7.9903e-05 1.1525e-05

1783.3 6.6369e-05 6.4062e-05 7.4663e-05 8.1171e-05 7.8358e-05 5.6622e-05 6.649e-05 5.8551e-05 7.1094e-05 6.7279e-05 9.2907e-05 5.5102e-05 3.8162e-05 4.3053e-05 5.7571e-05 6.2641e-05 6.062e-05 5.4275e-05 4.6048e-05 2.7492e-05 3.117e-05 1.1886e-05 2.9515e-05 2.4192e-05 2.8639e-05 2.1759e-05 6.2356e-05 8.6197e-05 8.4918e-05 1.229e-05 -4.01e-05 -0.00010246 2.655e-05 4.1221e-05 2.4861e-05 -1.5311e-05 9.4541e-05 0.00010417 5.8979e-05 0.00015939 -5.3918e-06 -3.3392e-05 -2.3667e-05 -2.9911e-05 -5.4474e-06 -0.00010235 -4.7045e-06 -4.9411e-05 -4.9499e-05 -3.4352e-05 -3.5883e-05 -7.2386e-05 -0.00010998 -0.00020312 -0.00015776 7.1003e-05 3.925e-05 0.00017267 5.894e-05 -1.1585e-05 8.7017e-05 -0.00012555

1790.4 8.6416e-05 8.4405e-05 9.6794e-05 9.8512e-05 9.4135e-05 8.6944e-05 8.9085e-05 8.9901e-05 0.00010662 9.2486e-05 0.00010171 5.4817e-05 9.2781e-05 9.3075e-05 5.4705e-05 6.6561e-05 8.32e-05 6.9037e-05 6.2792e-05 5.4931e-05 5.1265e-05 5.4577e-05 6.5569e-05 2.4867e-05 5.6936e-05 3.1342e-05 5.8313e-05 3.6157e-05 -7.6853e-06 -0.00010816 -4.634e-05 4.7632e-05 0.00011869 0.00018118 0.00014908 7.8898e-05 0.00013278 0.00016136 0.00011961 0.00012861 -9.4605e-06 -2.4505e-05 2.6884e-05 -3.6657e-05 4.8909e-05 -1.1783e-05 -1.068e-05 -0.00015686 4.4572e-05 4.635e-06 8.0968e-05 7.987e-06 -6.5338e-05 -0.00015343 2.0099e-05 9.6624e-05 -3.4285e-06 0.00022935 -5.7789e-05 4.1382e-06 5.8806e-05 -0.00014714

1797.4 0.00010966 0.00011526 0.00011216 0.00010427 0.0001114 0.00010762 0.00011632 0.0001219 0.00012456 0.00010932 7.7395e-05 8.9199e-05 0.00011411 0.00012399 5.6633e-05 9.187e-05 8.7897e-05 8.5917e-05 0.00011228 8.9611e-05 7.4389e-05 0.00011755 6.5087e-05 5.7314e-05 5.8854e-05 6.1123e-05 4.6655e-05 5.3407e-07 -8.5187e-05 -8.0186e-05 9.241e-05 9.0408e-05 0.00010192 0.00015058 0.00019281 0.00017938 0.00011598 0.00018471 0.00017373 0.0001274 6.3145e-05 2.6231e-05 2.0201e-05 5.8417e-06 8.847e-05 3.8804e-05 -7.8732e-06 -0.00014429 7.0686e-05 8.0756e-06 0.0001077 2.8915e-05 6.292e-05 0.00013218 5.8895e-05 7.3982e-05 4.8101e-05 0.00017139 -4.3203e-06 5.3078e-05 -4.3027e-05 -8.4356e-05

1804.5 0.00012128 0.0001162 9.5939e-05 9.4046e-05 9.9599e-05 8.5951e-05 9.9246e-05 0.00011685 0.00010422 7.9553e-05 6.3049e-05 8.7136e-05 7.2617e-05 9.7089e-05 3.9934e-05 7.6238e-05 8.168e-05 7.8363e-05 0.00012464 8.8645e-05 8.4728e-05 0.0001142 4.9854e-05 8.1902e-05 4.2925e-05 6.238e-05 3.8129e-05 3.4957e-06 -6.8011e-05 1.1175e-05 0.00013487 5.067e-05 6.9268e-05 0.00012442 0.00015189 0.00017087 7.5503e-05 0.00013009 0.00011705 8.4656e-05 5.6568e-05 2.702e-05 1.3443e-05 6.5937e-05 4.8817e-05 -4.8381e-05 7.1427e-06 -0.00014455 -3.2841e-05 9.1361e-05 6.6024e-05 1.8122e-05 8.829e-05 0.00014072 -2.3644e-05 -7.1031e-05 2.2715e-05 4.7838e-05 -4.5988e-05 5.0495e-06 -0.00010996 -0.00024908

1811.5 8.6236e-05 8.9253e-05 7.0601e-05 8.9644e-05 7.3538e-05 5.1151e-05 7.4318e-05 8.155e-05 6.7492e-05 4.7554e-05 5.1846e-05 7.2372e-05 3.9491e-05 6.0018e-05 3.9066e-05 4.7967e-05 6.6904e-05 6.8355e-05 0.00010353 5.8697e-05 7.0138e-05 5.5812e-05 4.9557e-05 8.3084e-05 5.3761e-05 5.7696e-05 3.9731e-05 2.996e-05 2.5117e-05 9.9083e-05 9.4439e-05 7.6748e-05 0.00012027 0.0002328 0.00015004 8.9671e-05 4.5777e-06 1.5696e-05 -2.9385e-07 -3.5782e-05 -5.2389e-05 5.784e-05 5.6746e-05 7.4412e-05 7.0604e-05 -3.7378e-05 1.7953e-05 -0.000167 -0.00012219 9.2786e-05 7.3995e-05 0.00016413 4.3768e-05 -3.9748e-05 -3.7708e-05 -0.00018971 7.0037e-05 7.8697e-05 7.5712e-05 1.9852e-05 -5.2305e-05 -5.0237e-06

1818.6 7.364e-05 7.5925e-05 8.6187e-05 9.5759e-05 6.3236e-05 5.1634e-05 6.7908e-05 6.8091e-05 3.9209e-05 3.6639e-05 5.1089e-05 6.8833e-05 6.4799e-05 6.4988e-05 2.1633e-05 8.2043e-05 4.6178e-05 4.6492e-05 6.7023e-05 4.7228e-05 5.0472e-05 1.367e-05 3.2107e-05 3.6437e-05 4.6549e-05 3.6854e-05 3.6355e-05 3.4467e-05 6.9761e-05 0.00010007 4.5395e-05 0.00011396 0.00010262 0.00013183 0.00011325 -1.8367e-05 -5.6497e-05 -7.5625e-05 -2.6817e-05 -7.2428e-05 -0.00010503 0.00014756 8.5521e-05 -2.053e-05 8.8681e-05 2.8479e-06 -3.2097e-05 -0.00011511 -0.00013734 2.5951e-05 -4.6033e-05 0.00017931 8.3459e-06 -0.00017007 -7.7496e-05 -0.00015004 -7.0771e-05 -1.5155e-05 7.7961e-05 -2.6643e-05 -0.00012497 0.00016022

1825.6 9.6759e-05 8.4747e-05 0.0001034 0.00010276 7.8615e-05 8.7325e-05 8.1415e-05 8.8748e-05 5.8211e-05 4.9645e-05 0.00011182 0.00010751 9.7189e-05 8.2626e-05 4.3198e-05 0.00010152 5.3456e-05 5.5931e-05 5.4722e-05 6.413e-05 5.4428e-05 3.7049e-05 5.1637e-05 6.2944e-06 5.3816e-05 4.415e-05 7.4286e-05 8.3124e-05 0.00013077 0.0001422 3.8065e-05 0.00015408 1.3023e-05 -6.684e-05 -1.8949e-05 -1.8317e-05 1.3453e-05 -3.3817e-05 -1.1995e-05 3.1667e-05 -6.1008e-05 0.0001227 0.00010239 -3.3162e-05 9.9686e-05 6.3794e-05 -4.4226e-05 -3.6707e-05 4.6076e-07 2.5204e-05 -0.0001057 -4.0421e-05 -8.7694e-05 -0.00010748 -3.1454e-05 1.1291e-06 -5.5522e-05 -3.6007e-05 5.9205e-06 9.4993e-05 -0.00018163 9.4487e-05

1832.6 5.821e-05 7.4049e-05 8.4024e-05 6.9021e-05 7.0513e-05 7.1979e-05 6.593e-05 7.5067e-05 6.735e-05 6.3342e-05 0.00011803 8.852e-05 0.00010833 7.103e-05 5.386e-05 7.5343e-05 4.8407e-05 5.5607e-05 3.7844e-05 6.4987e-05 6.2637e-05 5.2203e-05 5.7979e-05 2.3455e-05 8.5866e-05 9.1767e-05 0.00013464 0.00012427 0.00015225 0.00020777 0.00014837 0.00010321 -8.3227e-05 -0.00011559 -7.3807e-05 3.7459e-06 7.4454e-05 3.0535e-05 2.5807e-05 0.00014038 2.7485e-05 -4.7505e-06 0.00011795 1.7447e-05 6.8034e-05 0.00010531 -1.1961e-06 5.1759e-05 3.4282e-05 -1.2169e-05 -3.4095e-05 -0.00017076 -5.8084e-05 -3.0741e-05 3.9137e-05 7.2996e-05 0.00016146 0.000113 7.6307e-05 0.00010378 -5.4776e-05 -6.3511e-05

1839.6 5.4827e-05 7.4799e-05 7.4761e-05 5.4396e-05 6.6432e-05 5.516e-05 5.4571e-05 4.2508e-05 6.9579e-05 6.541e-05 9.21e-05 5.651e-05 7.4858e-05 4.5838e-05 5.6487e-05 5.1892e-05 2.2777e-05 5.2196e-05 2.447e-05 4.0032e-05 5.0827e-05 4.7183e-05 5.7853e-05 2.9214e-05 9.9431e-05 0.00010061 0.00012922 0.0001453 0.00021732 0.00028542 0.00019028 1.7499e-05 -4.3018e-05 -0.000133 -0.00012344 -4.511e-07 -1.0233e-06 3.6377e-05 8.4289e-05 0.00011593 2.1522e-05 -8.5976e-05 8.1766e-05 -3.3614e-06 5.4977e-05 9.2375e-05 2.9478e-05 9.4958e-05 -0.00011226 -4.3497e-05 -4.2017e-05 -0.00010635 -8.0785e-05 -0.00014979 -2.8183e-05 7.1778e-05 0.00020935 0.00011029 0.00013982 1.68e-05 -7.0413e-05 -8.1223e-05

1846.6 5.3027e-05 6.8417e-05 3.6147e-05 4.8036e-05 5.313e-05 4.1417e-05 3.8582e-05 2.8201e-05 6.1199e-05 5.5443e-05 6.7266e-05 4.8537e-05 5.2347e-05 1.4755e-05 4.8639e-05 4.1335e-05 1.9616e-05 2.9962e-05 2.6132e-05 2.3213e-05 4.6127e-05 3.6505e-05 5.0533e-05 2.5086e-05 7.364e-05 4.8891e-05 6.1379e-05 0.00013082 0.00019426 0.00026731 8.1313e-05 -1.1849e-05 4.1947e-05 -4.8128e-05 -8.768e-05 3.7325e-05 -1.8293e-05 3.6835e-05 6.8098e-05 5.7163e-05 1.4287e-05 -3.0238e-05 3.35e-05 -4.8688e-06 4.7258e-05 3.4723e-05 3.068e-05 9.2352e-05 -0.00015005 1.5617e-05 -4.7737e-05 -5.7937e-05 -5.6876e-05 -9.4752e-05 -0.0001159 0.00010497 0.00022207 6.559e-05 4.195e-05 -6.7131e-07 -9.4455e-05 -9.529e-05

1853.5 7.8271e-05 0.00010125 6.3029e-05 7.4394e-05 8.5664e-05 5.3548e-05 5.2575e-05 6.3687e-05 8.0619e-05 6.3208e-05 8.8046e-05 8.1903e-05 9.0486e-05 3.6661e-05 8.2487e-05 4.2588e-05 3.523e-05 4.706e-05 6.0456e-05 5.3762e-05 8.5942e-05 7.458e-05 7.5087e-05 5.3179e-05 9.5213e-05 6.0313e-05 5.1857e-05 5.3768e-05 8.0679e-05 0.00012613 2.128e-05 2.8055e-05 0.00013527 0.00013276 9.0517e-05 7.2173e-05 1.1956e-05 7.7751e-05 4.8391e-05 3.1313e-05 7.9547e-06 4.6011e-05 6.8349e-05 1.8709e-05 4.3398e-05 6.1472e-05 -3.7422e-06 7.283e-05 -6.7452e-05 7.1227e-05 1.4797e-05 -6.171e-05 -2.3122e-05 1.907e-05 -4.7561e-05 0.00015227 0.00023903 6.6624e-06 3.9221e-05 6.3043e-05 -5.215e-05 -0.00020491

1860.5 0.00013061 0.00011352 0.00010189 9.9913e-05 0.00010447 6.9659e-05 6.8419e-05 7.3985e-05 9.0248e-05 6.465e-05 7.5608e-05 9.7428e-05 0.00012185 0.0001004 9.8223e-05 4.7003e-05 4.4863e-05 4.9736e-05 6.8106e-05 8.2744e-05 9.4455e-05 8.6407e-05 9.3657e-05 7.9028e-05 0.00011185 7.1475e-05 2.2489e-05 -3.8762e-05 -5.9263e-05 -8.3146e-05 2.8799e-06 0.00010454 0.00015705 0.00019054 0.00027425 0.00018814 0.00011055 0.00010566 8.9327e-05 7.4279e-05 4.3953e-05 3.4436e-05 9.2572e-05 4.1784e-05 5.5148e-05 0.00014667 3.4292e-05 8.5583e-06 1.2355e-05 7.5795e-05 2.1855e-05 2.2546e-05 1.9748e-05 7.8223e-05 0.00012008 0.00010303 0.00034963 1.4098e-07 -4.0913e-07 3.3525e-05 -0.00018268 -8.9592e-05

Fig. 3d

0 1772.8 1767.2 1761.6 1756 1750.5 1745 1739.6 1734.2 1728.8 1723.5 1718.2 1712.9 1707.7 1702.5 1697.4 1692.3 1687.2 1682.2 1677.2 1672.2 1667.3 1662.4 1657.5 1652.7 1647.9 1643.1 1638.4 1633.7 1629 1624.3 1619.7 1615.2 1610.6 1606.1 1601.6 1597.1 1592.7 1588.3 1583.9 1579.5 1575.2 1570.9 1566.7 1562.4 1558.2 1554 1549.9 1545.7 1541.6 1537.5 1533.5 1529.5 1525.4 1521.5 1517.5 1513.6 1509.7 1505.8 1501.9 1498.1 1494.3 1490.5

1402.1 -8.29e-05 -5.4709e-05 -6.3185e-05 -8.6039e-05 -8.0918e-05 -0.00011524 -0.00010153 -0.00010407 -8.2527e-05 -7.9435e-05 -9.5881e-05 -0.00017874 -0.0001388 -0.00012237 -0.00010538 -8.8718e-05 -6.4817e-05 -6.878e-05 -0.00011704 -7.0828e-05 -7.7436e-05 -7.4256e-05 -0.000106 -3.0005e-05 -3.4309e-05 -4.2233e-05 -0.00010492 -7.0622e-05 -0.00010401 -0.00014547 -5.8447e-05 -1.0697e-05 -0.0001085 -7.5522e-05 -8.9587e-05 -3.7321e-05 -3.8028e-05 -6.8842e-05 3.0156e-05 1.8618e-05 9.1718e-05 0.00014272 -3.9861e-05 -9.9473e-05 -5.9573e-05 0.00013558 4.7126e-05 0.00012331 -1.7678e-05 -8.6724e-05 0.00017397 -4.2807e-05 4.2324e-05 0.00011528 7.4909e-05 0.00026714 0.00060754 9.8589e-05 4.4609e-05 0.00024714 7.6432e-05 -0.00010844

1409.6 -5.6804e-05 -4.1324e-05 -4.1732e-05 -6.5948e-05 -5.776e-05 -7.7294e-05 -6.457e-05 -6.7135e-05 -6.5471e-05 -5.948e-05 -8.0325e-05 -0.00013973 -0.00011904 -8.704e-05 -6.5022e-05 -4.3038e-05 -1.1809e-05 -2.1182e-05 -5.9804e-05 -3.4674e-05 -4.0079e-05 -4.0186e-05 -5.8051e-05 -1.3925e-05 -1.8241e-05 -1.9422e-05 -5.6173e-05 -1.6169e-05 -3.0174e-05 -5.5354e-05 1.2494e-05 2.3313e-05 -0.00011278 -6.0273e-05 -5.5867e-05 -6.8466e-05 -8.0126e-05 -9.5148e-05 -6.8466e-05 -4.6386e-05 -5.5712e-06 0.00010112 -4.6204e-05 -0.0001085 -4.9342e-05 7.0467e-05 2.2101e-05 8.6132e-05 -4.3563e-05 -5.2191e-05 9.8409e-05 -6.5779e-05 3.6129e-05 7.7504e-05 -8.2366e-07 0.00018367 0.00062053 -5.9141e-05 4.8669e-05 0.00018437 2.6566e-05 -4.9184e-05

1417.2 -3.0586e-05 -2.6902e-05 -1.5538e-05 -4.0565e-05 -1.9461e-05 -1.6753e-05 -7.8504e-06 -2.0589e-05 -2.5119e-05 -2.4816e-05 -5.4253e-05 -9.3807e-05 -8.8043e-05 -4.8026e-05 -2.6248e-05 2.4526e-05 3.5632e-05 2.38e-05 1.0929e-05 2.0843e-05 4.7781e-06 1.7806e-06 -1.9266e-06 -1.7392e-05 -4.7587e-06 -8.8848e-06 -1.4161e-05 9.8193e-06 2.7139e-05 -2.0394e-05 2.2271e-05 3.4509e-08 -0.00010149 -4.494e-05 4.0865e-06 1.5205e-05 -1.6646e-05 -1.6435e-05 -9.0277e-05 -6.157e-05 -6.3566e-05 1.3813e-05 7.3693e-06 -5.2871e-05 2.7032e-05 1.313e-05 2.6388e-05 2.5868e-07 2.098e-05 -6.0334e-07 6.9829e-05 -3.9933e-05 9.2153e-05 7.1396e-05 3.5957e-06 5.154e-05 0.00044552 -0.00013618 -1.9299e-05 0.00026633 0.00012702 0.00012969

1424.8 -1.4301e-06 1.3006e-05 3.9614e-05 1.8289e-05 3.8185e-05 4.6825e-05 4.1802e-05 1.0418e-05 3.6224e-05 2.1585e-05 -2.4419e-06 -3.7758e-05 -4.6452e-05 -1.7974e-05 -3.1624e-06 4.9243e-05 6.0747e-05 6.0306e-05 5.7021e-05 6.1661e-05 3.5659e-05 3.4984e-05 3.3547e-05 -1.4979e-05 6.682e-06 4.9596e-06 2.8465e-07 -1.2772e-06 -1.2598e-05 -8.0158e-05 -3.0167e-05 -2.1707e-05 -8.2716e-06 -1.3184e-05 4.2531e-05 0.00010785 4.4337e-05 7.2019e-05 -1.2624e-05 -3.59e-05 -3.9712e-05 -5.7397e-05 4.1515e-05 -2.0818e-05 8.6123e-05 -1.1587e-05 -1.0826e-05 -0.00011343 4.9336e-05 -2.0333e-05 0.00015844 4.4103e-05 5.8774e-05 2.1229e-05 1.89e-05 -4.8899e-05 0.00018336 -3.5403e-05 -7.8915e-05 0.0002056 0.00017046 0.00025029

1432.4 9.0543e-06 2.626e-05 4.0436e-05 3.7774e-05 4.4334e-05 6.2274e-05 3.9546e-05 2.335e-05 3.9693e-05 2.5772e-05 1.8095e-05 -1.0197e-05 -3.4538e-05 -2.5678e-05 -1.576e-05 1.891e-05 6.7954e-05 6.523e-05 6.0026e-05 6.1596e-05 3.7946e-05 4.3432e-05 4.0428e-05 -2.8924e-05 2.8554e-05 2.0163e-05 -1.8325e-05 -2.9755e-05 -1.7315e-05 -4.2706e-05 2.863e-05 3.2436e-05 0.00010734 5.9477e-05 7.3789e-05 4.7859e-05 -4.4144e-06 4.0197e-05 1.0237e-05 -5.8854e-05 -3.0076e-05 -7.9278e-05 6.4822e-05 -2.6816e-05 5.0964e-06 -4.8405e-05 -4.9148e-05 -9.3336e-05 -6.1032e-05 -7.9825e-05 0.00017249 7.3579e-05 -3.6464e-05 -0.00014087 -5.6253e-05 -6.6866e-05 0.00019887 -2.5564e-05 -2.458e-05 -6.5757e-05 9.3509e-05 0.00023026

1439.9 -2.5656e-05 -1.469e-05 -2.4049e-05 -1.2841e-05 5.4518e-06 8.0907e-06 -7.3838e-06 -2.1995e-06 -1.3804e-05 -4.1908e-06 -1.6841e-05 -4.3834e-05 -6.5401e-05 -4.4887e-05 -2.7973e-05 -8.7681e-06 2.2856e-05 2.7245e-05 2.5761e-05 1.6843e-05 -5.63e-06 7.282e-06 -1.1113e-05 -1.9531e-05 1.519e-05 -1.0542e-06 -4.2264e-05 -8.6186e-05 -5.5219e-05 -0.00011955 -1.7756e-05 -1.7032e-05 3.9525e-05 2.3689e-05 1.7815e-05 -1.3762e-06 1.351e-05 5.0387e-05 4.755e-05 -2.9671e-05 5.1609e-05 -5.8246e-06 5.8289e-05 1.7353e-05 -2.3219e-05 -3.2044e-05 -4.2912e-07 3.4524e-05 -0.00010451 -1.638e-05 0.00016128 3.1692e-05 -8.8164e-06 3.079e-05 -0.00018042 6.778e-05 0.00039382 -3.1675e-05 0.00013431 -9.8204e-05 0.00017177 9.1679e-05

1447.4 -2.8068e-06 -5.8886e-06 -1.6684e-05 -9.5356e-06 1.3313e-05 -1.6088e-06 -5.1643e-06 -1.2609e-06 -7.9673e-06 1.908e-06 -3.0816e-05 -4.5271e-05 -4.817e-05 -3.3209e-05 -4.2009e-06 1.4082e-05 2.6916e-05 1.9102e-05 2.2417e-05 1.076e-07 -4.0979e-06 -8.2056e-06 -1.432e-05 4.9471e-05 2.4195e-05 -1.3691e-05 -3.6574e-05 -7.1565e-05 -6.0495e-05 -0.00015434 -9.5124e-05 -7.065e-05 -5.7295e-05 2.9637e-05 2.1246e-05 1.1307e-05 3.2466e-05 4.5427e-05 7.5714e-05 4.6619e-05 6.2706e-05 5.0227e-05 1.8773e-05 -4.5292e-05 -4.1659e-05 -6.0473e-05 -4.9445e-05 1.6669e-05 -6.5662e-05 9.9232e-06 0.00013611 2.5558e-05 3.1357e-05 4.4733e-06 -0.00028208 0.00016841 0.00037638 -3.0531e-05 4.5405e-05 -4.0754e-05 3.8833e-05 -0.0001829

1455 1.7868e-05 1.0607e-05 -5.3824e-06 3.7492e-06 5.3401e-06 6.6236e-06 1.5889e-06 -2.2386e-05 -1.4264e-05 -8.2301e-06 -2.8029e-05 -2.7876e-05 -1.7492e-05 -3.0978e-05 -1.5707e-05 -1.3224e-05 8.6618e-06 2.1688e-05 2.4425e-05 5.0216e-06 2.0451e-05 1.4002e-06 -6.1603e-06 4.2493e-05 2.9376e-05 -6.6439e-06 -7.905e-06 -9.9182e-06 2.7021e-05 -3.4116e-05 -9.6725e-05 -7.1228e-05 -8.8943e-05 4.5432e-05 4.2148e-05 -3.0112e-05 5.3894e-07 -1.7837e-05 -1.1346e-05 2.225e-05 -1.6969e-05 1.5714e-05 5.2292e-05 -6.8558e-05 -1.482e-05 -2.2937e-05 -4.9367e-05 5.3989e-06 -5.7301e-05 -2.0236e-05 0.00013463 -2.5755e-05 4.226e-06 -9.0965e-05 -0.00026466 0.00013052 9.2633e-05 -4.1768e-05 -0.00010079 -2.2066e-06 -6.2994e-05 -0.00032142

1462.5 1.911e-05 2.6815e-05 1.1911e-05 2.5493e-05 1.8752e-05 3.1421e-05 3.2127e-05 -3.9129e-06 2.0995e-06 3.1893e-06 4.5429e-06 4.4256e-06 1.0242e-05 -1.7514e-05 1.6217e-06 -1.3473e-05 -2.137e-06 2.6233e-05 1.4682e-05 2.3087e-05 4.2348e-05 1.2463e-05 -4.9998e-06 -2.2325e-07 1.0306e-05 7.1076e-06 -2.7436e-05 -1.3265e-06 2.7406e-05 -2.0061e-05 -6.2439e-05 -6.4012e-05 -8.828e-05 -4.315e-05 -2.4627e-06 1.6894e-05 4.2363e-05 4.323e-05 -2.9264e-05 5.2765e-05 -3.0082e-05 2.2217e-05 5.1702e-05 1.7541e-05 -8.6388e-05 -2.5961e-06 -2.2724e-05 4.5537e-06 -9.0544e-05 2.5176e-05 0.00011397 -1.5267e-05 -3.5151e-05 -2.8442e-05 -3.7813e-05 0.00015277 -9.1719e-05 -6.3904e-05 9.7196e-05 3.3877e-05 1.6571e-05 -0.00018541

1470 5.8354e-06 4.9355e-05 3.3846e-05 3.5597e-05 3.943e-05 3.6218e-05 3.6687e-05 1.8372e-05 3.0998e-05 1.5991e-05 2.1442e-05 -1.2597e-06 -2.6641e-06 -2.3245e-05 1.2972e-05 8.4102e-06 6.2003e-06 1.5605e-05 7.5051e-06 3.3975e-05 3.5318e-05 2.9463e-06 -1.4547e-06 -6.2401e-06 -2.8856e-05 5.9643e-06 -4.8518e-05 6.8271e-06 1.545e-06 -6.2962e-05 -5.728e-05 -5.2352e-05 1.347e-05 -3.0799e-05 -1.3462e-05 7.5272e-05 6.5779e-05 7.906e-05 -2.6637e-05 3.9883e-05 1.3927e-05 2.3506e-05 4.2272e-05 8.867e-05 -5.3945e-05 4.667e-05 -5.362e-05 -7.0223e-05 -0.00011092 7.5171e-05 3.8157e-05 5.7641e-05 -0.00012422 -3.5385e-05 7.9635e-05 7.078e-05 -8.0503e-05 -5.7363e-05 0.00021122 3.5782e-05 2.9911e-05 2.6467e-05

1477.5 -4.9382e-06 5.5762e-05 4.4325e-05 3.0261e-05 3.7886e-05 2.4179e-05 1.2399e-05 8.1487e-06 3.2203e-05 3.2338e-05 5.5287e-06 1.4878e-05 -4.9465e-07 -1.6744e-05 8.0373e-06 2.8643e-05 2.5368e-05 1.2013e-05 1.7301e-05 3.802e-05 3.4807e-05 1.6566e-05 3.7335e-06 2.9456e-05 -2.8264e-05 -1.9737e-06 -2.6432e-05 1.6886e-05 7.3698e-07 2.2276e-06 2.957e-05 7.5667e-06 8.1772e-05 5.9523e-05 2.6127e-05 7.9036e-05 1.4531e-05 4.7339e-05 -1.342e-05 -9.6155e-06 6.9225e-05 1.8112e-05 8.3508e-05 6.0977e-05 6.006e-05 5.1857e-05 -3.8664e-05 -0.00012067 2.4901e-06 9.5361e-05 -1.2678e-05 9.4e-05 -0.00012448 -9.6832e-05 -3.7857e-05 3.2671e-05 0.00025846 3.6732e-05 0.00012692 9.0072e-05 4.3995e-05 8.5249e-05

1485 4.4779e-05 7.9018e-05 7.6001e-05 6.082e-05 5.5952e-05 5.22e-05 2.856e-05 3.5906e-05 6.0599e-05 5.1691e-05 3.6413e-05 5.2626e-05 3.0475e-05 4.0563e-05 2.8953e-05 4.8802e-05 6.586e-05 3.685e-05 4.0107e-05 4.8384e-05 3.9633e-05 4.5331e-05 2.513e-05 2.3351e-05 3.3421e-05 3.3922e-05 3.196e-05 2.8081e-05 9.0081e-06 -1.7048e-05 3.1022e-05 -9.6991e-06 7.6853e-05 0.00010922 7.2618e-05 0.00011338 7.0605e-05 -3.888e-06 1.1478e-05 -1.5438e-06 7.2325e-05 -3.045e-06 9.63e-05 -1.8793e-05 3.1739e-05 7.4524e-05 2.7228e-05 -6.0565e-05 7.2017e-05 6.5921e-05 5.8484e-05 1.8239e-05 -5.0814e-05 -0.00010936 -6.6135e-05 0.00014402 0.000392 3.1453e-05 5.1598e-05 9.5342e-06 0.00024618 6.1831e-05

1492.5 8.3575e-05 9.2724e-05 9.6228e-05 0.00010136 8.8659e-05 8.9865e-05 6.8589e-05 8.7219e-05 0.00010014 8.4072e-05 6.182e-05 6.1409e-05 7.1746e-05 9.2117e-05 5.253e-05 9.0006e-05 0.0001045 7.0707e-05 9.1748e-05 8.8563e-05 8.0226e-05 7.6716e-05 8.2017e-05 1.4946e-05 0.00010099 7.7721e-05 0.00010381 5.523e-05 4.2175e-05 2.0431e-06 -3.6234e-06 -0.00013706 2.7219e-05 4.2277e-05 3.3927e-05 0.00012997 8.5044e-05 1.6787e-05 3.5671e-05 -2.1175e-06 4.0461e-05 -5.4032e-05 5.6487e-05 -5.9461e-05 -2.2678e-05 8.3577e-05 9.0846e-05 -6.9041e-06 4.8123e-05 4.3696e-05 4.2249e-05 -3.5257e-05 -9.9622e-05 -0.00014084 -3.3371e-05 9.3155e-05 2.7463e-05 -8.6981e-05 -9.2672e-05 -0.00016558 0.0001418 -0.00019877

1499.9 6.0934e-05 3.7946e-05 6.6155e-05 6.7639e-05 6.9438e-05 7.4832e-05 3.5559e-05 6.3087e-05 6.1349e-05 5.312e-05 2.8873e-05 1.3103e-05 3.9482e-05 8.4932e-05 4.8952e-05 7.5355e-05 7.275e-05 6.4257e-05 8.8082e-05 8.6716e-05 7.6383e-05 4.8902e-05 7.487e-05 4.5716e-06 8.3063e-05 4.2629e-05 0.00010831 4.5355e-05 2.079e-05 4.6343e-06 -2.7137e-05 -0.00012129 1.6523e-05 -9.2571e-05 3.5633e-06 5.8597e-05 1.652e-05 7.3377e-05 5.2801e-06 -2.5575e-06 2.6588e-05 -1.6301e-05 9.7541e-05 4.2957e-05 5.2825e-05 2.6536e-05 7.9761e-05 -3.6681e-05 -2.0087e-05 4.6886e-06 2.1112e-05 -8.7678e-05 -0.0001501 -6.2764e-05 -5.2079e-05 -5.7166e-05 -0.00045223 -0.00017861 -8.4252e-05 -0.00011732 5.1155e-06 -0.00021711

1507.4 -3.9462e-05 -5.3298e-05 -4.6654e-05 -3.7068e-05 -5.2356e-05 -2.5363e-05 -7.4878e-05 -4.3934e-05 -3.7271e-05 -4.9378e-05 -5.2877e-05 -8.2662e-05 -7.3135e-05 -2.3928e-05 -2.6011e-05 -2.5716e-05 -2.723e-05 -9.0667e-06 -6.0562e-06 -1.0111e-05 -1.971e-05 -4.223e-05 -4.6064e-06 -4.4415e-05 -1.949e-05 -4.8471e-05 -1.2518e-05 -6.758e-05 -5.9401e-05 -3.9231e-05 -4.3506e-05 -3.7929e-05 2.1668e-05 -0.00010356 2.0906e-05 -1.0981e-05 1.6227e-05 3.793e-05 -2.0648e-05 -2.0694e-07 3.7775e-05 1.7126e-05 8.0057e-05 0.00013761 4.3523e-05 -7.3646e-06 1.5806e-05 -6.5239e-05 -7.1807e-05 -2.6421e-06 2.4052e-05 -9.5767e-05 -5.073e-05 -6.57e-05 -0.00015082 -9.3397e-05 -0.00038808 -0.00018073 6.5842e-06 -1.3097e-05 2.6642e-05 -8.4633e-06

1514.8 -9.5698e-05 -8.2019e-05 -8.9273e-05 -6.7025e-05 -0.00011219 -7.7553e-05 -8.6508e-05 -5.3956e-05 -5.8661e-05 -5.8852e-05 -6.8639e-05 -9.2627e-05 -0.00010583 -3.9843e-05 -4.792e-05 -3.5537e-05 -3.2053e-05 -2.3528e-05 -2.594e-05 -4.99e-05 -3.2421e-05 -5.3111e-05 -2.8697e-05 -4.7178e-05 -3.8956e-05 -4.8703e-05 -7.6674e-05 -9.8768e-05 -4.9264e-05 -5.1921e-05 -8.7257e-06 6.7027e-06 2.7973e-05 5.21e-06 8.3696e-05 2.6389e-06 5.6243e-05 3.1196e-06 2.8747e-05 4.3796e-05 8.8106e-05 0.000134 4.2366e-05 7.7111e-05 6.037e-05 7.4992e-05 7.711e-05 -9.6294e-06 4.699e-05 1.7192e-05 6.5539e-05 -1.5321e-06 7.1831e-05 -0.00011501 -0.00014971 -1.8348e-05 9.4077e-05 1.811e-06 5.9011e-05 0.00013338 -5.1944e-05 0.00010685

1522.3 -7.7485e-05 -8.3968e-05 -7.5726e-05 -5.3958e-05 -9.3041e-05 -6.1053e-05 -4.7565e-05 -3.5356e-05 -3.6966e-05 -3.9799e-05 -6.2571e-05 -6.7987e-05 -7.3842e-05 1.3419e-05 -9.565e-06 2.1599e-05 3.4442e-05 4.2871e-06 1.3606e-05 -4.9676e-06 2.5622e-05 9.3096e-06 1.7036e-05 -3.0198e-05 -2.6292e-06 -1.826e-05 -3.3615e-05 -9.1178e-05 -3.7561e-05 -1.0251e-05 1.3965e-05 4.855e-05 5.9293e-05 8.7152e-05 0.00016877 0.00010966 0.0001449 7.8673e-05 9.1936e-05 0.00011949 0.00012113 0.0001502 4.7848e-05 6.1823e-05 9.3593e-05 0.00015902 8.841e-05 1.6928e-05 0.00015296 4.2236e-05 9.0188e-05 1.8456e-06 0.00011819 -7.7276e-05 -2.0636e-05 -1.243e-05 0.00028831 -1.6837e-05 4.1059e-05 0.00016368 6.3479e-06 8.6079e-05

1529.7 -8.6739e-05 -0.00010249 -9.0088e-05 -7.3436e-05 -9.0193e-05 -5.5549e-05 -6.6594e-05 -8.3993e-05 -5.2416e-05 -7.3771e-05 -0.00011886 -0.00013367 -0.00010861 6.4307e-06 -1.1726e-05 1.1259e-05 7.4622e-06 -2.0674e-05 4.6777e-06 8.3773e-06 2.2941e-05 1.4285e-05 1.4915e-05 -3.7196e-05 7.0914e-06 -2.3854e-05 -1.9092e-05 -8.1966e-05 -4.5417e-05 -3.8304e-05 -5.3941e-06 5.9267e-05 0.00012501 0.00017338 0.00020557 0.0002394 0.00017536 0.00012361 6.7966e-05 3.4036e-05 6.2469e-05 7.3883e-05 7.6227e-05 0.00012176 0.00014659 0.00015035 6.9599e-05 4.9863e-05 0.00027834 0.00014635 0.00016058 8.4184e-05 8.1791e-05 6.4706e-06 3.51e-05 3.5711e-05 0.00021552 -9.156e-05 4.8001e-05 8.4968e-05 0.00019841 0.00015127

1537.1 -8.456e-05 -8.9869e-05 -8.2768e-05 -6.1878e-05 -8.5475e-05 -5.3238e-05 -6.4583e-05 -9.6978e-05 -5.6496e-05 -9.1587e-05 -0.00016132 -0.00018854 -0.00011699 2.7857e-05 3.175e-05 2.1915e-05 1.2445e-05 -7.5577e-06 1.8913e-06 9.9299e-07 3.6233e-06 -1.8704e-06 -1.638e-05 -2.6098e-05 1.1635e-05 -1.9146e-05 -6.0688e-05 -0.00011677 -0.00013829 -0.00012361 -5.5859e-05 -3.6147e-06 0.00017008 0.00020438 0.00016757 0.00024558 0.00022036 0.00013091 7.2701e-05 9.1389e-05 0.00021497 0.00024309 0.00022576 0.00024589 0.00022603 0.0002666 0.00017605 0.00017857 0.00031714 0.00030945 0.00033169 0.00025284 0.00016053 0.00019939 9.5298e-05 0.00021997 0.00036266 0.00024759 0.00018822 0.00028013 0.00028793 0.00029216

1544.5 -7.3821e-05 -7.3556e-05 -6.7989e-05 -5.1381e-05 -7.3487e-05 -6.1287e-05 -5.3618e-05 -9.4186e-05 -4.9565e-05 -9.8555e-05 -0.00017821 -0.00020945 -0.00010394 0.00010396 0.00011444 7.0048e-05 6.3308e-05 2.6206e-05 1.8501e-05 -5.4117e-07 3.8003e-08 -3.7124e-06 -1.446e-05 -2.7517e-05 -5.4915e-05 -9.842e-05 -0.00016878 -0.00027325 -0.00036619 -0.00036204 -0.00023847 -0.00016484 7.7753e-05 0.00018295 0.00016171 0.00017714 0.00027789 0.00020117 0.00022587 0.00034053 0.00035204 0.00033465 0.00024708 0.00022872 0.00020447 0.00030084 0.00019046 0.00019473 0.0002031 0.00034718 0.00034737 0.00023056 0.00030461 0.00020909 0.00010746 0.00026813 0.00044682 0.00037413 0.00015115 0.00030628 0.00019055 0.00015417

1551.9 -5.086e-05 -4.6447e-05 -3.8739e-05 -4.5578e-05 -6.1485e-05 -4.429e-05 -4.5667e-05 -8.3617e-05 -5.5289e-05 -0.00011778 -0.00019681 -0.00023958 -0.000122 0.0001009 0.00013276 7.3741e-05 5.7638e-05 -3.8001e-06 -1.9722e-05 -3.8751e-05 -3.8562e-05 -2.9601e-05 -2.5546e-05 -5.4552e-05 -0.0001419 -0.00018551 -0.00025146 -0.00039736 -0.00048867 -0.00044803 -0.00033279 -0.00023549 -3.2341e-05 0.00021811 0.00019872 0.00020305 0.00037052 0.00035775 0.00033609 0.00030655 0.00016601 0.00012987 0.00012359 9.6282e-05 0.00015261 0.0001809 0.00019087 0.00030535 0.00015974 0.00032275 0.00026865 0.00021032 0.00039598 0.00031005 0.00022737 0.00026973 0.00039191 0.00046294 0.00019533 0.00022278 0.00032662 6.9311e-06

1559.3 -4.3259e-05 -5.0272e-05 -4.3069e-05 -5.5388e-05 -6.4549e-05 -5.2689e-05 -4.824e-05 -8.6397e-05 -8.7521e-05 -0.00014426 -0.00023231 -0.00028863 -0.00016139 6.5456e-05 0.00012302 7.7731e-05 4.0937e-05 -1.1183e-05 -3.5232e-05 -5.4869e-05 -6.8924e-05 -5.0819e-05 -5.4346e-05 -7.9928e-05 -0.0001661 -0.0002022 -0.00025568 -0.00044511 -0.0005648 -0.00053097 -0.00035779 -0.00013873 -2.6618e-05 0.00038349 0.00031699 0.00039751 0.00045111 0.00030148 0.00012409 6.126e-05 5.3041e-05 8.7815e-05 7.1869e-05 9.3676e-05 0.00016742 0.00017689 0.00026391 0.0003803 0.00012807 0.00035613 0.0002885 0.00021603 0.00027851 0.00040831 0.00028932 0.00029054 0.00044646 0.0004696 0.00042921 0.0003227 0.00044888 0.0002511

1566.7 -7.2584e-05 -6.8608e-05 -4.3513e-05 -6.4412e-05 -8.6551e-05 -6.0959e-05 -5.6116e-05 -9.751e-05 -9.2972e-05 -0.00015081 -0.00028139 -0.00034845 -0.0001845 0.00010828 0.00018066 0.00016221 9.9368e-05 2.573e-05 1.906e-05 1.0136e-05 -1.2141e-05 6.9012e-06 -6.5675e-06 -6.9916e-05 -0.00012296 -0.0001797 -0.0002536 -0.00055943 -0.00076757 -0.00078641 -0.00058017 -0.00022906 -5.3814e-05 0.00044333 0.00053495 0.00059768 0.00038263 0.0001077 6.8336e-06 1.3433e-05 3.0812e-05 1.0796e-05 0.00010101 0.00014656 0.00024128 0.00026928 0.0002274 0.00023217 0.00015173 0.00026615 0.00029168 0.00023171 0.00010649 0.00030083 0.00022983 9.4286e-05 0.00028543 0.00017941 0.0003968 0.00027192 0.0002668 0.00025931

1574 -7.8309e-05 -6.1936e-05 -4.6434e-05 -5.6253e-05 -8.2107e-05 -6.0207e-05 -5.6948e-05 -0.00012257 -0.00011452 -0.00019225 -0.00038161 -0.00049942 -0.0002934 9.1942e-05 0.00020788 0.00018641 0.00010696 7.404e-07 -9.1843e-06 -3.353e-05 -5.2742e-05 -6.2808e-05 -7.4532e-05 -0.0001325 -0.00016227 -0.00024957 -0.00037353 -0.00064784 -0.00089077 -0.00092023 -0.00070295 -0.00035134 -0.00011088 0.00021721 0.00026634 0.00014435 4.0058e-05 0.00010145 0.00010373 0.00013712 8.7172e-05 4.9857e-05 0.00020625 0.00021372 0.00030723 0.00036683 0.00030219 0.0002404 0.00030567 0.0002246 0.00026616 0.00027881 0.00015554 0.00027047 0.00038295 0.00012404 0.00040252 0.00019236 0.0003744 0.00014906 0.00015934 0.00023988

1581.4 -9.25e-05 -8.6132e-05 -9.4163e-05 -7.611e-05 -0.00010939 -9.7459e-05 -9.7705e-05 -0.00018123 -0.00018698 -0.00028969 -0.00054873 -0.00075165 -0.00048519 3.9611e-05 0.0002805 0.00021737 0.00013529 1.1085e-06 -4.3755e-05 -7.4249e-05 -9.7407e-05 -0.00012767 -0.00012607 -0.00021445 -0.00021703 -0.00035289 -0.00052441 -0.00087067 -0.0011352 -0.0012239 -0.00097574 -0.0006654 -0.00043534 -0.00016841 1.7058e-05 0.00037916 0.00065818 0.0007374 0.00053167 0.00041656 0.00028835 0.00028783 0.00023976 0.00026534 0.00023339 0.0002968 0.00027872 0.0001733 0.00028767 0.00023851 0.00019489 0.0001874 0.00022047 0.00017155 0.00032693 0.0002021 0.00030511 0.00019898 0.00036034 0.00014472 0.00027491 0.0002758

1588.8 -0.00016985 -0.00015988 -0.00017123 -0.0001421 -0.00018002 -0.00015938 -0.0001729 -0.00022668 -0.00025317 -0.00039871 -0.00076307 -0.0010976 -0.00074362 6.7967e-05 0.00050828 0.00040345 0.00026021 9.923e-05 2.2949e-05 -2.1036e-05 -7.2232e-05 -0.00010774 -0.00013088 -0.00029592 -0.00033108 -0.00050431 -0.00076965 -0.0013416 -0.0018119 -0.0021842 -0.0019798 -0.0015418 -0.0011515 -0.00021368 0.00062075 0.0014797 0.0016005 0.0013756 0.0011002 0.00085242 0.00074238 0.00064914 0.00044006 0.00044039 0.00031876 0.0003276 0.00026828 0.00018837 0.00023887 0.00018275 0.00013058 0.00020039 0.00016933 5.0163e-05 8.3021e-05 0.00012646 0.00021641 0.00016261 0.00022429 0.00022026 0.00035599 0.00020524

1596.1 -0.00010852 -8.5014e-05 -0.00010002 -7.2238e-05 -0.00011053 -9.662e-05 -0.00012427 -0.00016847 -0.00022803 -0.00045185 -0.00098381 -0.0015843 -0.0011475 9.7997e-05 0.00082959 0.00072692 0.0004892 0.00023036 0.00011922 1.9295e-05 -7.458e-05 -0.00013738 -0.00022198 -0.00046034 -0.00054246 -0.00079011 -0.0012318 -0.002097 -0.002938 -0.0036246 -0.0034833 -0.0026357 -0.0015673 0.0003279 0.001551 0.0022187 0.0021809 0.0019668 0.0016853 0.001394 0.0011443 0.0009484 0.00073545 0.00060806 0.00057274 0.00049595 0.0003858 0.00041417 0.00039386 0.00031853 0.00026698 0.00031355 0.00016315 0.00013051 0.00017102 0.00017834 0.00023534 0.00013986 0.00012322 0.00025435 0.00031649 4.1883e-05

1603.4 -6.0442e-06 3.2403e-06 -9.253e-07 7.7769e-06 -4.6875e-05 -3.5753e-05 -7.3281e-05 -0.00014739 -0.00021448 -0.00052186 -0.0012219 -0.0021091 -0.0016538 1.4078e-05 0.0011497 0.0010514 0.00074285 0.00038759 0.00024526 0.00010087 -3.3529e-05 -9.8092e-05 -0.00018048 -0.00043517 -0.00054482 -0.00092031 -0.0015801 -0.002844 -0.0041789 -0.0051533 -0.0048658 -0.0030999 -0.0013232 0.00064847 0.0019567 0.002917 0.0032338 0.0030276 0.0025314 0.0020694 0.0015052 0.0011842 0.00089814 0.00068677 0.0005165 0.00042591 0.00038643 0.00033831 0.0004558 0.00032196 0.00032113 0.00029719 0.00016094 0.00013793 0.00016225 0.00015528 3.0775e-05 -3.4648e-06 5.4375e-05 0.00015361 0.00016059 -3.7527e-05

1610.7 -7.5382e-05 -3.9999e-05 -4.7939e-05 -3.621e-05 -6.681e-05 -5.0451e-05 -8.0344e-05 -0.00018385 -0.00025914 -0.00059927 -0.0014086 -0.0023547 -0.0018928 -9.9968e-05 0.0012353 0.0011791 0.00087652 0.00046857 0.00030025 0.00011174 -3.1555e-05 -0.00011306 -0.00019837 -0.00052598 -0.00070992 -0.0012173 -0.00208 -0.0037624 -0.0055682 -0.0066327 -0.0058451 -0.0035505 -0.0016019 0.00051863 0.0024299 0.0038674 0.0041772 0.0038607 0.00318 0.002622 0.0020775 0.0016912 0.0013469 0.0010549 0.00073083 0.00053132 0.00054549 0.00035318 0.00049278 0.00023219 0.00031043 0.00030613 0.00025179 0.00011495 0.00010991 0.00011731 0.00011127 2.1247e-05 0.00016239 0.00025837 9.5894e-05 8.3178e-05

1618 -6.7849e-05 -3.3438e-05 -4.1783e-05 -1.6072e-05 -4.2275e-05 -2.8722e-05 -5.0772e-05 -0.00014857 -0.00024314 -0.00056866 -0.001374 -0.0021714 -0.0016504 -1.9423e-05 0.0010826 0.00099976 0.00075695 0.00038615 0.0002342 4.1417e-05 -6.8799e-05 -0.00017686 -0.00033623 -0.0008597 -0.0011545 -0.0019076 -0.0031814 -0.0055814 -0.0079563 -0.0090581 -0.0073279 -0.0038191 -0.00069385 0.0024235 0.0044882 0.0054389 0.005127 0.0044303 0.0034925 0.002783 0.0021759 0.0017473 0.0014215 0.0012531 0.00093845 0.0007226 0.00070812 0.00055467 0.00061486 0.00038052 0.00029019 0.00045652 0.00046065 0.0002406 0.00021157 0.00031856 0.00016483 0.00019491 0.00033284 0.00045925 0.00021684 0.00033246

1625.3 -4.9864e-05 -3.8778e-05 -3.9322e-05 -3.3269e-05 -5.7712e-05 -5.2484e-05 -7.6028e-05 -0.00012154 -0.00022734 -0.00049879 -0.0011509 -0.001746 -0.00118 8.4758e-05 0.00080331 0.00070588 0.00055289 0.00025803 0.0001545 1.7923e-05 -5.6608e-05 -0.00018754 -0.00034742 -0.00091296 -0.0012955 -0.002208 -0.0038455 -0.0066813 -0.008957 -0.0094844 -0.0068407 -0.0018818 0.0018245 0.0043686 0.0053803 0.0054893 0.0048639 0.0039899 0.0031068 0.0023688 0.0017602 0.0013578 0.0010767 0.00089504 0.00062217 0.00055964 0.00044839 0.00036422 0.00047365 0.00032675 0.00024315 0.00031892 0.00038282 0.00018043 7.7219e-05 0.00028311 1.4788e-05 0.00018914 0.00026639 0.00033109 0.00021469 0.00035086

1632.6 -5.2648e-05 -3.7477e-05 -4.7897e-05 -5.6085e-05 -5.1453e-05 -4.3528e-05 -5.451e-05 -8.5843e-05 -0.00016087 -0.00034426 -0.00079319 -0.0011689 -0.00070633 8.4736e-05 0.00052136 0.0004657 0.00040101 0.00021282 0.0001447 3.5289e-05 -6.6604e-05 -0.00018642 -0.00029102 -0.00065839 -0.00095739 -0.001603 -0.0027124 -0.004385 -0.0056202 -0.0056486 -0.0036811 -0.00024449 0.0018099 0.0029256 0.003127 0.0031117 0.0027981 0.0022329 0.0017599 0.0013176 0.0010256 0.00078955 0.00064964 0.00049175 0.0003335 0.00031701 0.00024164 0.0001257 0.00028251 0.00018666 0.00023074 0.00011699 9.5002e-05 -1.5329e-05 5.877e-05 0.00020225 -2.8008e-05 2.4103e-05 0.00011833 0.00012264 0.00015652 0.00021357

1639.9 -4.379e-05 -8.5032e-06 -2.6835e-05 -1.6708e-05 -2.1238e-05 -1.6653e-05 -1.4147e-05 -4.8323e-05 -8.4007e-05 -0.00018062 -0.00047064 -0.00069267 -0.0004198 -1.0426e-05 0.00026837 0.00028131 0.000244 0.0001277 5.2397e-05 -2.6962e-05 -9.4401e-05 -0.00013073 -0.00017036 -0.00034921 -0.00046353 -0.00074445 -0.0011515 -0.0017979 -0.0024859 -0.0023944 -0.0014578 -0.00026602 0.00049661 0.00094805 0.0010687 0.0011608 0.0010897 0.00088415 0.00065583 0.00042189 0.00040545 0.00030351 0.000238 0.00024635 0.00015683 0.00014842 0.00012682 1.0446e-05 0.00020404 0.00016289 0.00018126 0.00010763 1.4867e-05 -1.5032e-05 0.00014675 0.0001753 -0.00013979 -5.9264e-05 5.2159e-05 0.0001591 0.00012003 0.00020136

1647.2 -4.3923e-05 -3.0524e-05 -4.0895e-05 -3.3013e-05 -4.5735e-05 -5.0127e-05 -4.6707e-05 -7.3161e-05 -8.7462e-05 -0.00012104 -0.00030059 -0.00043797 -0.00032181 -7.4951e-05 0.00012667 0.00013802 0.00010171 1.1998e-06 -7.2501e-05 -0.00010178 -8.1112e-05 -8.2055e-05 -8.7131e-05 -0.00020465 -0.00026684 -0.00043548 -0.00072529 -0.0012242 -0.0014711 -0.0011397 -0.00077224 -0.00029865 0.00017183 0.00042681 0.00052856 0.00056517 0.00048472 0.0004072 0.00021673 0.00010304 0.00014934 0.00013544 2.9036e-05 8.4255e-05 -7.253e-05 -1.3513e-05 -3.0259e-05 -9.2565e-05 0.00010315 4.9756e-05 1.5196e-05 -8.8157e-05 -2.1855e-05 -4.6848e-05 0.00011414 -1.8566e-05 -0.000325 -0.0001029 1.2206e-05 5.3232e-05 7.903e-05 5.5066e-05

1654.4 -8.1778e-05 -8.2119e-05 -9.0578e-05 -8.9716e-05 -9.5332e-05 -9.9972e-05 -9.9297e-05 -0.00012295 -0.00011823 -0.00014073 -0.00026262 -0.00034789 -0.00031995 -0.00012682 1.6996e-05 3.038e-06 -8.5719e-06 -9.533e-05 -0.00010596 -9.7816e-05 -7.9455e-05 -0.00010219 -0.00013731 -0.00018926 -0.00026174 -0.00040384 -0.00054966 -0.00058555 -0.0006494 -0.0006109 -0.00054738 -0.0002812 2.2783e-05 0.00019427 0.00017366 0.00017621 0.0001372 0.00014188 -3.4734e-05 -2.6668e-05 -1.1524e-05 2.515e-05 -4.7685e-05 -2.0056e-05 -9.9101e-05 -2.8742e-05 9.3568e-06 -6.5766e-05 7.7342e-05 -4.705e-05 -2.2487e-05 -0.00011575 -5.127e-06 -0.00011034 7.8733e-05 -7.8571e-05 9.8875e-05 9.6377e-05 7.2957e-05 -3.7154e-05 7.6258e-05 -3.3693e-05

1661.7 -6.3347e-05 -6.1313e-05 -7.007e-05 -5.9689e-05 -7.672e-05 -7.5382e-05 -7.8038e-05 -0.00011807 -8.3895e-05 -0.00012549 -0.0002504 -0.00032302 -0.00031909 -0.00014172 -1.328e-05 -2.6753e-05 -1.2674e-05 -2.9342e-05 -4.1766e-06 -5.6882e-05 -8.5253e-05 -0.00011253 -0.00012797 -0.00011284 -0.00012198 -0.00016965 -0.00014415 -0.00014333 -0.00041374 -0.00052837 -0.00045397 -0.00033871 -0.00016006 -7.048e-05 -3.3399e-05 4.8214e-05 5.895e-05 0.00014738 -1.3047e-05 3.2163e-05 -5.0901e-06 1.7263e-05 -5.6169e-05 1.2139e-05 -6.336e-06 1.6957e-06 3.1051e-05 4.2834e-05 0.00011101 -7.4836e-05 -1.7014e-05 5.701e-05 0.00014505 6.1278e-05 0.00020731 3.6522e-05 0.00032926 0.00027333 0.00020447 -3.2936e-05 0.00014492 -8.3362e-05

1668.9 -0.0001089 -9.2529e-05 -0.00010096 -8.8604e-05 -0.00011792 -0.00010409 -9.5499e-05 -0.00013446 -0.00010033 -0.000149 -0.00025635 -0.00034775 -0.0003334 -0.00021089 -9.6543e-05 -6.4726e-05 -9.7569e-06 1.8562e-05 -1.0133e-05 -0.00010708 -0.00011024 -0.00010471 -8.3958e-05 -3.9304e-05 4.4553e-06 9.1464e-06 -5.6011e-05 -0.00030205 -0.00052183 -0.00063016 -0.00059083 -0.00049924 -0.00029869 -0.00017793 -1.5273e-05 9.3132e-05 0.00014465 0.00019375 7.7719e-05 0.00011785 2.0442e-05 5.2169e-05 -7.5421e-05 4.2176e-05 -7.9815e-05 -1.8399e-05 -7.1342e-05 7.3001e-05 0.00010733 -3.732e-05 -3.6888e-05 2.6501e-05 7.8395e-05 6.8011e-05 0.00020875 1.6819e-05 -1.9045e-05 0.00015456 0.00016173 -0.00014245 0.00010689 -0.00013929

1676.1 -0.00014821 -0.00011671 -0.00013288 -0.00010027 -0.00014948 -0.00012411 -0.0001049 -0.00011757 -0.00011856 -0.0001607 -0.00024319 -0.00038323 -0.0004257 -0.00037165 -0.00021492 -5.1512e-05 5.2503e-05 5.3489e-05 -2.2436e-06 -7.2323e-05 -7.9979e-05 -6.9455e-05 -4.1875e-05 1.6596e-05 -1.1817e-05 -9.1373e-05 -0.00021602 -0.00038801 -0.0005288 -0.00062041 -0.00069554 -0.00060108 -0.00034505 -0.00015215 4.1673e-05 0.0001011 0.00017508 0.00014458 4.3118e-05 2.9727e-05 7.8621e-06 3.8354e-06 -8.2753e-05 -5.3886e-05 -0.00011219 -6.3497e-05 -0.00012399 -2.8201e-05 5.7378e-05 -3.5474e-05 -6.3243e-05 -0.00012359 -3.6611e-05 -8.8985e-05 0.00013124 -3.578e-05 -0.0001146 1.9299e-05 2.4876e-06 -2.1801e-05 4.6662e-06 -3.044e-05

1683.4 -1.5217e-05 1.7175e-05 -6.2366e-06 3.3697e-05 -6.3159e-06 1.3721e-05 2.7395e-06 -5.489e-06 1.3346e-06 -4.5632e-05 -0.00016228 -0.00035177 -0.00043025 -0.00031996 6.6888e-05 0.00033724 0.00033435 0.00017769 7.4656e-05 1.3524e-05 9.7453e-06 2.6708e-05 5.4322e-05 1.9518e-05 -6.8938e-05 -0.00012328 -0.00016312 -0.00034458 -0.00050093 -0.0006181 -0.00074591 -0.00057656 -0.00025101 9.2658e-06 0.00023729 0.00028168 0.00029913 0.00029433 0.00017438 6.5813e-05 0.00010508 6.1009e-05 3.4505e-05 -4.7711e-07 2.1135e-05 2.3643e-05 6.3247e-06 -5.5577e-05 8.0599e-05 1.8482e-06 -3.4654e-05 -0.00011383 3.4724e-05 6.6936e-06 0.00022352 9.4561e-06 0.0002735 1.0898e-05 -8.7788e-05 0.00013613 -3.1901e-06 8.6221e-05

1690.6 7.2436e-05 8.5471e-05 8.4975e-05 9.2581e-05 7.4368e-05 9.3128e-05 6.2834e-05 6.2075e-05 6.3565e-05 2.6294e-05 -0.00014823 -0.00042257 -0.00056674 -0.00030773 0.00019616 0.00035114 0.00027036 0.00019782 0.00019179 0.00019193 0.00019591 0.00018967 0.00012499 3.7718e-05 1.0522e-06 -1.4219e-05 -0.00011826 -0.00039181 -0.00063774 -0.0008781 -0.00091791 -0.00070111 -0.00033914 4.586e-05 0.00036956 0.00048467 0.00051392 0.00051494 0.00037891 0.00024536 0.00018294 0.00014151 0.00013843 9.818e-05 8.4541e-05 7.1549e-05 9.7312e-05 2.0708e-05 0.00019411 4.6553e-05 -3.7566e-05 1.8992e-05 0.00014237 0.00013645 0.00013873 3.5356e-05 0.00022463 6.8014e-05 -4.0795e-05 9.4127e-05 1.2834e-05 1.4145e-05

1697.8 4.5597e-05 4.6494e-05 6.6353e-05 6.7722e-05 6.8428e-05 8.5382e-05 6.056e-05 4.7808e-05 4.8225e-05 -3.1774e-05 -0.00034274 -0.00088777 -0.0014107 -0.0013617 -0.00071239 -4.3245e-05 0.00039374 0.00075865 0.00076768 0.00062407 0.0004651 0.00030929 0.00020077 0.0001404 0.00013116 3.5303e-05 -0.00012986 -0.00046474 -0.00081977 -0.0012647 -0.001239 -0.0010248 -0.00061881 -0.00013283 0.00027836 0.00058986 0.00077719 0.00063561 0.00049168 0.00039453 0.00028198 0.00019309 0.00015871 0.00013969 0.00012743 0.00012281 0.00010894 3.8105e-05 0.00018014 5.2769e-05 4.5358e-05 6.3871e-05 0.00013189 4.86e-05 1.9304e-05 7.0911e-05 -0.00020333 1.5547e-05 -5.7009e-05 4.5268e-05 7.98e-05 -9.7598e-05

1704.9 3.1451e-05 2.62e-05 4.8476e-05 5.4875e-05 5.1016e-05 6.6595e-05 5.1534e-05 2.0788e-05 1.2039e-05 -0.00014812 -0.00071724 -0.0018391 -0.0029657 -0.0027262 -0.00097859 0.00049478 0.0011532 0.001428 0.0012433 0.00091201 0.00062004 0.00039442 0.00034115 0.00020625 0.00018662 4.3661e-05 -0.00012418 -0.00053544 -0.00098074 -0.0014114 -0.0013758 -0.0011573 -0.0007789 -0.00028451 0.00021931 0.00068981 0.0008975 0.00073416 0.00064167 0.00048357 0.00035311 0.00031226 0.00017603 0.00018311 0.00014396 0.0001408 0.00012839 -3.8111e-05 8.8432e-05 5.0192e-05 0.00016713 4.7697e-05 2.4608e-05 -0.00013167 2.9821e-05 5.2944e-05 -0.00024798 -6.8575e-05 -6.1223e-05 -8.3822e-05 1.9903e-05 -0.0001034

1712.1 4.3024e-05 1.7594e-05 3.6997e-05 3.8497e-05 1.4853e-05 1.9974e-05 2.4776e-05 -1.0943e-05 -6.5661e-05 -0.00029241 -0.0010658 -0.0023318 -0.0030684 -0.0021285 -8.2867e-05 0.0010679 0.00135 0.0012772 0.001043 0.00074919 0.00055285 0.0004073 0.00033952 0.00021721 0.0001857 5.9242e-05 -0.00013178 -0.00044821 -0.0009378 -0.0012259 -0.0011848 -0.00086083 -0.00050872 -9.2036e-05 0.00026139 0.00066854 0.000714 0.00061834 0.00057245 0.0003723 0.00027908 0.0002799 0.00016273 0.0001662 0.00013842 0.00015822 0.00014158 -7.9004e-05 9.723e-05 4.0648e-05 0.00010369 0.00011305 -4.5153e-05 -2.3897e-05 5.9731e-05 9.3319e-05 -0.00025703 -7.3999e-06 -2.2702e-05 -9.881e-05 7.5103e-05 9.5304e-05

1719.3 4.6218e-05 3.8311e-05 2.8719e-05 3.0796e-05 2.7709e-05 1.965e-05 2.1034e-05 -2.7199e-05 -0.00010364 -0.00029996 -0.00080741 -0.0013733 -0.0014422 -0.00069635 0.00031125 0.00076745 0.00081741 0.00065538 0.00051932 0.00038266 0.00031774 0.00023514 0.00016947 0.00016958 0.00010733 3.147e-05 -0.00011081 -0.00032039 -0.00066192 -0.00081942 -0.00075442 -0.00037943 -2.2938e-06 0.00012244 0.00021721 0.00038024 0.00037249 0.00031121 0.00027548 0.00015865 0.00017316 0.00013003 0.00011694 0.00013081 0.0001339 9.602e-05 0.0001078 -4.7186e-05 0.00014036 3.4043e-05 -2.4874e-07 0.00017573 9.505e-06 0.00014873 0.00017325 8.593e-05 -8.3017e-07 0.00022249 4.0299e-05 0.00014516 0.00021032 0.00021889

1726.4 3.7896e-05 6.7205e-05 4.3571e-05 4.0332e-05 5.7372e-05 4.0974e-05 4.2493e-05 1.4763e-05 -3.2082e-05 -6.2871e-05 -0.00018552 -0.00035772 -0.00034963 -6.5662e-05 0.00018285 0.00027531 0.00027774 0.00020738 0.00018777 0.00012846 9.8039e-05 5.4274e-05 4.191e-05 4.8238e-05 -2.8845e-06 -1.6761e-05 -0.00010219 -0.00027817 -0.00038617 -0.00052656 -0.00036064 -0.00016099 0.00014327 0.00015612 0.0001765 0.00024541 0.0001815 0.00014343 0.00015865 8.2331e-05 0.00012014 8.2071e-05 9.7153e-05 8.6968e-05 -9.1965e-07 3.5582e-05 5.5132e-05 5.611e-05 6.9724e-05 2.2151e-05 1.8865e-05 0.00013303 6.2058e-05 0.00014516 0.00023366 7.2264e-05 0.00016583 0.00029491 2.0605e-06 -8.5638e-05 0.00012554 0.00020126

1733.6 9.124e-06 4.02e-05 3.5335e-05 1.2587e-05 4.155e-05 3.0368e-05 3.1712e-05 3.877e-05 2.0511e-05 3.9108e-05 -5.6151e-05 -0.00013961 -9.0529e-05 -2.0808e-05 1.6247e-05 7.2635e-05 6.3255e-05 5.5716e-05 6.9273e-05 1.6059e-05 1.3584e-05 1.3021e-05 3.7498e-06 -2.7485e-05 -3.3091e-05 -4.4246e-05 -9.3436e-05 -0.00021648 -0.00025104 -0.00031435 -0.0001327 -9.876e-05 3.4649e-05 6.1022e-05 0.00011124 0.00022482 0.00011823 0.00014076 0.00015505 4.1002e-05 6.0862e-05 6.3778e-05 6.424e-05 2.7782e-05 -7.9601e-05 0.00011694 7.8824e-06 9.4535e-05 -0.00010572 7.0817e-06 2.3569e-05 3.5043e-05 3.5645e-05 0.00015166 6.5398e-05 6.4185e-05 0.00012153 0.00025073 -0.0001812 -0.00031539 -1.3715e-05 -4.5748e-05

1740.7 2.1799e-05 3.5603e-05 4.2741e-05 3.0531e-06 4.192e-05 3.6722e-05 2.2978e-05 3.1168e-05 3.9994e-06 -7.6769e-06 -9.8062e-05 -6.4616e-05 -3.7967e-05 -3.8427e-05 1.1303e-06 6.6365e-05 2.6075e-05 3.4947e-05 5.2401e-05 2.6606e-05 2.277e-05 3.6935e-05 2.2517e-05 -1.2673e-05 1.0053e-05 -2.7006e-05 -5.5018e-05 -7.3853e-05 -8.8003e-05 -4.8728e-05 -4.7266e-05 2.0191e-05 2.6702e-05 7.8585e-05 6.2202e-05 8.7179e-05 7.404e-05 7.2742e-05 3.8994e-05 -4.1159e-05 1.5393e-05 3.6871e-05 4.2085e-06 -3.2547e-05 3.6778e-06 9.4955e-05 1.445e-05 1.6246e-05 -3.6153e-05 2.3388e-05 -9.3047e-05 -8.4999e-05 -4.8102e-05 2.7459e-05 -0.00011401 1.4478e-05 0.00012675 0.00013838 -0.00023409 -0.00019165 -8.514e-05 8.106e-05

1747.8 6.2536e-05 6.5455e-05 7.4959e-05 4.179e-05 7.5435e-05 6.1069e-05 6.2735e-05 4.5311e-05 3.827e-05 3.7544e-05 2.8361e-06 2.7219e-05 4.1793e-05 4.526e-05 5.7487e-05 0.00010336 5.496e-05 6.6597e-05 8.8094e-05 7.7497e-05 5.5521e-05 6.3018e-05 7.0696e-05 4.4566e-05 5.9778e-05 4.0067e-05 2.7736e-06 5.4971e-06 -2.2682e-06 2.074e-05 -4.3054e-05 0.00013336 6.0933e-05 0.00015404 9.5562e-05 9.437e-05 0.00012224 9.7608e-05 1.7597e-05 -1.9319e-05 5.3192e-05 2.8647e-05 3.1076e-05 -4.9002e-05 0.00011954 8.9284e-05 5.0236e-05 -4.8285e-05 8.9235e-05 5.7167e-05 -7.3415e-05 -9.9754e-06 -0.00012034 -1.0883e-05 -2.2945e-05 0.00011805 0.00010295 -8.882e-06 -0.00010033 -9.5449e-05 -3.0429e-05 0.00018844

1755 4.9152e-05 5.1707e-05 8.2362e-05 5.8424e-05 7.618e-05 5.2563e-05 5.4035e-05 2.9014e-05 5.1101e-05 5.7448e-05 5.7357e-05 4.2934e-05 6.2865e-05 7.9603e-05 5.3658e-05 6.4645e-05 3.1813e-05 6.1661e-05 7.4899e-05 6.6689e-05 3.2005e-05 3.6432e-05 3.9205e-05 4.348e-05 5.6787e-05 6.6735e-05 2.3644e-05 3.668e-05 6.2733e-06 -5.7059e-06 -1.8175e-05 0.00011593 5.8953e-05 5.0802e-05 -1.4997e-05 7.6906e-05 7.4907e-05 9.0831e-05 4.8666e-06 -9.4176e-06 7.5098e-05 4.3748e-05 3.1731e-05 -3.0349e-05 0.00016445 7.716e-05 2.6373e-05 6.6107e-06 6.2182e-05 7.7887e-05 1.1002e-05 3.2208e-05 -9.3049e-05 4.5448e-05 1.2841e-05 0.00019336 -0.00029066 4.9303e-05 7.3998e-05 -3.6454e-06 2.8786e-05 0.00011734

1762.1 4.1199e-05 3.5699e-05 5.4942e-05 4.1874e-05 5.3127e-05 1.6807e-05 3.4125e-05 3.213e-05 3.7463e-05 5.1983e-05 5.3191e-05 3.0061e-05 5.8087e-05 7.9466e-05 3.0647e-05 3.5809e-05 2.3636e-05 5.4061e-05 5.1752e-05 4.5119e-05 2.626e-05 2.0097e-05 1.6992e-05 2.0511e-05 3.7987e-05 5.7967e-05 3.3567e-05 4.1557e-05 1.3206e-05 4.2677e-05 5.3418e-05 6.4556e-05 4.8466e-05 -3.384e-05 -0.00013294 -4.1412e-05 -3.478e-05 -1.6309e-05 -4.3615e-07 -1.325e-05 6.64e-05 2.1758e-05 -7.4728e-06 -1.8703e-05 0.00010735 3.1414e-05 2.2018e-05 0.00011254 -7.3684e-05 0.0001168 2.3008e-05 -9.7181e-06 -6.3268e-05 8.8747e-05 -2.6391e-05 0.00014055 -0.00024696 2.6359e-05 7.5051e-05 2.5835e-05 3.8969e-05 0.00013576

1769.2 3.1566e-05 1.9028e-05 3.6752e-05 1.6696e-05 2.8177e-05 7.1242e-06 4.3901e-05 5.2637e-05 3.9091e-05 5.1384e-05 3.7205e-05 3.8736e-05 4.3018e-05 3.6861e-05 3.1377e-05 4.2468e-05 3.2482e-05 5.6879e-05 4.9719e-05 2.9351e-05 3.6265e-05 2.2921e-05 2.3599e-05 -3.0166e-07 2.0787e-05 2.3574e-05 -2.7205e-06 4.5841e-05 8.1447e-06 0.00010228 -1.3391e-06 -6.6017e-06 -5.9506e-05 -2.7929e-05 -0.00013578 -4.1787e-05 -2.7792e-05 -1.7668e-05 7.7896e-06 -8.6698e-06 4.3647e-05 1.4154e-06 -1.8236e-05 -2.2046e-05 2.2761e-05 -3.9134e-06 5.2287e-05 2.4892e-05 6.4682e-06 3.3236e-05 -5.3392e-06 -1.3348e-07 -6.7193e-05 0.00016046 1.5311e-05 5.3625e-05 -9.8804e-05 -4.4227e-05 0.00010052 0.00012422 -1.5901e-05 0.0001357

1776.3 3.8005e-05 1.4203e-05 7.0038e-05 2.7995e-05 4.0621e-05 4.193e-05 6.5097e-05 6.6874e-05 5.696e-05 5.2258e-05 2.4966e-05 2.797e-05 1.3218e-05 -8.4367e-06 3.0265e-05 3.9778e-05 4.5105e-05 5.8815e-05 4.9202e-05 3.4154e-05 4.057e-05 3.6972e-05 5.4098e-05 3.0106e-05 3.7445e-05 3.0791e-05 2.8988e-05 0.00010183 6.2514e-05 0.00011491 -2.263e-05 -2.2364e-05 -2.9452e-05 1.2277e-05 -4.6458e-05 3.6648e-05 3.8963e-05 7.7672e-05 1.9223e-05 -6.0523e-06 6.4415e-05 3.0586e-05 3.5333e-05 1.0991e-05 -4.2821e-05 4.0234e-05 -2.2158e-05 -7.3059e-05 0.00020473 -4.9183e-05 -6.5858e-05 0.00010777 -4.6823e-06 3.3794e-05 5.1447e-05 7.2908e-05 -0.00019166 8.0732e-06 0.0001944 0.00024994 -2.4247e-05 4.6334e-05

1783.3 5.3375e-05 2.0843e-05 6.921e-05 5.1846e-05 5.3507e-05 4.7594e-05 4.8331e-05 5.3168e-05 3.9941e-05 3.106e-05 3.551e-05 1.5555e-05 -7.437e-06 1.2996e-06 3.0247e-05 4.0303e-05 3.7628e-05 5.9172e-05 4.6117e-05 5.7441e-05 4.7915e-05 2.7203e-05 5.4392e-05 3.4375e-05 4.6801e-05 5.53e-05 6.0873e-05 8.8474e-05 9.1075e-05 6.8051e-05 -4.0003e-05 1.1886e-05 0.00013032 0.00010434 0.00010519 7.404e-05 7.677e-05 0.00012475 2.8623e-05 1.2997e-05 6.6903e-05 7.8014e-05 4.926e-05 7.4078e-05 -1.4145e-05 0.00014848 -5.3304e-05 1.6453e-05 0.00025063 -4.9433e-05 -1.484e-06 8.5855e-05 6.5753e-05 -6.6325e-05 0.00012617 7.4366e-05 0.00026813 7.8951e-05 0.00016969 0.00010523 1.1911e-05 -2.1458e-05

1790.4 3.5241e-05 2.3085e-05 4.7531e-05 4.5608e-05 4.8341e-05 2.7059e-05 1.8029e-05 3.8132e-05 3.0208e-05 8.9752e-06 3.1279e-05 1.6495e-05 2.9496e-05 1.6951e-05 2.755e-05 2.1538e-05 2.0936e-05 4.087e-05 3.5432e-05 4.6188e-05 2.8848e-05 1.1349e-05 1.8204e-05 -1.158e-05 5.2809e-05 2.5789e-05 -3.1841e-06 -5.3663e-06 1.5122e-05 -7.1053e-06 -6.1769e-05 5.5352e-05 0.00017206 0.0001351 0.00017165 4.3893e-05 0.00010078 0.00010893 7.4577e-05 3.568e-05 1.5698e-05 3.6665e-05 2.1137e-05 6.2888e-05 4.0173e-06 0.0001171 1.3456e-05 4.614e-06 0.00018864 3.2816e-05 -3.2087e-05 3.954e-05 7.5732e-05 -0.00014229 0.00011806 -4.3138e-05 0.00049655 5.0214e-05 -5.6637e-05 -8.3795e-05 4.9106e-05 2.0803e-05

1797.4 3.115e-05 5.278e-05 5.6346e-05 4.9145e-05 7.4665e-05 3.9322e-05 4.6035e-05 8.0127e-05 5.069e-05 3.85e-05 4.8893e-05 5.2493e-05 5.648e-05 5.5057e-05 4.4522e-05 2.9224e-05 1.223e-05 3.3429e-05 3.2318e-05 3.7708e-05 2.2728e-05 2.9926e-05 -6.1163e-07 1.5076e-05 6.6197e-05 1.9995e-05 1.3902e-05 1.6914e-05 -4.8815e-06 1.9687e-05 -5.4228e-06 9.2075e-05 0.00011593 4.9087e-05 6.0018e-05 1.2395e-05 8.6489e-05 5.6821e-05 6.6162e-05 -3.1557e-05 -4.0393e-05 -4.8246e-05 -9.5879e-06 1.4092e-05 -7.1655e-05 3.4029e-05 7.2659e-05 1.8885e-05 7.2045e-05 3.8553e-05 -8.8144e-05 5.2562e-05 3.6284e-05 -0.00017018 7.7106e-05 -5.2339e-05 9.9632e-05 0.00013867 -0.00015807 -0.00013937 -4.3178e-05 2.5327e-05

1804.5 6.9293e-05 0.00010311 9.0057e-05 8.4703e-05 9.6125e-05 7.8065e-05 8.3048e-05 0.00010369 7.3331e-05 7.8792e-05 7.6829e-05 7.8412e-05 8.699e-05 8.6298e-05 6.9565e-05 5.0603e-05 5.7238e-05 5.9763e-05 3.6008e-05 6.2142e-05 4.7631e-05 4.6016e-05 3.3752e-05 5.0082e-05 4.7328e-05 5.1217e-05 3.4515e-05 2.914e-05 4.0721e-05 5.8807e-05 5.051e-05 6.2196e-05 8.988e-05 8.5836e-06 2.4244e-05 3.7219e-05 9.5217e-05 3.955e-05 3.7459e-05 -1.6627e-05 1.8124e-05 9.9345e-07 8.6911e-06 5.0282e-05 -8.1961e-05 -3.1805e-05 9.9147e-05 8.5788e-05 7.9748e-05 2.6552e-05 2.6435e-05 -6.6657e-05 -3.8959e-06 -0.00010041 -3.0682e-06 5.3506e-05 -5.0095e-05 0.00013439 -0.00013242 -9.6479e-05 -0.00014419 -0.00013113

1811.5 0.00010966 0.0001137 0.00011119 9.942e-05 8.0632e-05 8.5738e-05 6.8776e-05 6.4212e-05 7.131e-05 7.9602e-05 7.393e-05 7.7441e-05 6.8242e-05 4.6747e-05 6.8743e-05 4.6908e-05 7.6098e-05 7.1834e-05 4.1055e-05 5.7248e-05 5.0148e-05 3.0102e-05 4.7221e-05 3.2203e-05 9.8438e-06 4.0382e-05 2.3245e-05 1.2488e-05 6.5275e-05 1.5907e-05 -4.3547e-05 3.6318e-05 7.2912e-05 5.8781e-05 0.00012657 8.0048e-05 0.00013174 6.2525e-05 3.6271e-05 3.7818e-05 3.2597e-05 2.2344e-05 4.1902e-05 9.1219e-05 -2.0941e-06 -2.9312e-07 7.0753e-05 8.686e-05 3.8388e-05 4.7238e-05 4.7579e-05 -9.3025e-05 5.2016e-05 7.2982e-05 -4.4869e-05 9.4677e-05 0.00028282 5.8027e-06 -9.6306e-05 9.2538e-06 -2.3131e-05 -0.00023488

1818.6 6.9507e-05 7.5166e-05 7.1676e-05 6.9108e-05 5.1352e-05 6.0632e-05 4.9457e-05 3.5535e-05 5.8311e-05 8.5417e-05 5.8071e-05 5.847e-05 3.3292e-05 2.5569e-05 3.827e-05 2.9117e-05 4.4877e-05 5.7333e-05 4.546e-05 3.9572e-05 4.9751e-05 2.6899e-05 2.6434e-05 4.3058e-05 3.4788e-05 4.4969e-05 5.2664e-05 3.424e-05 6.1912e-05 2.738e-05 -2.3772e-05 5.3799e-05 9.1171e-05 0.00011273 0.00017719 0.00012722 0.0001204 5.8629e-05 2.4717e-05 1.606e-05 1.9956e-05 -1.7494e-05 6.8418e-05 8.8988e-05 6.3614e-06 8.9089e-05 2.7966e-05 0.00010204 -2.2012e-05 0.00010982 6.992e-05 -5.764e-06 0.00011898 0.00013828 2.0455e-05 0.00013438 0.00031016 1.3364e-05 3.8315e-05 2.706e-05 5.1237e-05 1.8709e-05

1825.6 5.8137e-05 6.3714e-05 6.2979e-05 6.2806e-05 5.286e-05 6.5384e-05 5.2556e-05 6.5067e-05 6.7217e-05 9.3285e-05 6.4659e-05 6.0548e-05 4.478e-05 5.7678e-05 4.5331e-05 4.4321e-05 5.0824e-05 6.0714e-05 5.0435e-05 6.1948e-05 6.4183e-05 3.2044e-05 3.542e-05 7.5209e-05 7.9927e-05 8.059e-05 9.4886e-05 7.0951e-05 5.8947e-05 4.9278e-05 4.5092e-05 7.81e-05 8.3013e-05 6.677e-05 0.0001253 4.5518e-05 7.942e-05 4.953e-05 2.7761e-05 9.8883e-06 2.2869e-05 2.2444e-05 2.4654e-05 9.4428e-05 -2.5439e-06 0.00014837 3.3289e-05 6.5515e-05 -0.00010898 2.2808e-05 0.00014579 5.0813e-05 2.8463e-05 7.6136e-05 0.00012455 0.00012156 0.00024443 9.2326e-05 0.00010818 2.5349e-06 1.1151e-05 0.00033193

1832.6 5.2878e-05 6.4565e-05 7.7649e-05 6.113e-05 6.0305e-05 7.2263e-05 6.0761e-05 9.0586e-05 7.0846e-05 7.55e-05 6.4134e-05 4.8149e-05 7.8664e-05 9.5594e-05 4.0697e-05 3.6002e-05 6.8426e-05 6.8385e-05 4.1897e-05 6.1244e-05 6.067e-05 4.2111e-05 4.4391e-05 7.5302e-05 7.3865e-05 6.6999e-05 7.0787e-05 5.1154e-05 3.4587e-05 8.4386e-05 7.8131e-05 6.0508e-05 8.054e-05 -3.9187e-05 1.3143e-05 -4.8607e-05 7.1942e-05 5.397e-05 5.0093e-05 5.2227e-05 3.0208e-05 9.9178e-05 -2.0345e-05 0.00012125 5.0323e-05 0.00015068 8.9519e-05 -1.3783e-05 -0.00017859 -9.7001e-05 0.00015162 2.731e-05 -7.0941e-05 -1.7557e-05 9.5172e-05 1.088e-05 0.00010709 0.00023385 -6.8798e-05 8.4868e-05 2.3015e-05 0.00026411

1839.6 4.9626e-05 3.3917e-05 6.8941e-05 4.8143e-05 5.2208e-05 5.4739e-05 5.6413e-05 7.4677e-05 4.7646e-05 5.5671e-05 6.1539e-05 4.9918e-05 6.4351e-05 7.4497e-05 3.2753e-05 2.0749e-05 5.5667e-05 6.3405e-05 4.5244e-05 4.141e-05 5.119e-05 4.3355e-05 3.1912e-05 8.6184e-05 6.8114e-05 5.9575e-05 6.6756e-05 8.2629e-05 6.4945e-05 0.00019881 0.00015052 8.938e-06 9.3327e-05 2.3414e-07 7.598e-05 -8.3797e-06 8.2094e-05 2.4329e-05 7.279e-06 5.0771e-05 3.5353e-05 0.00011041 -8.8098e-06 8.1863e-05 0.00013046 0.00012455 0.00013667 -6.8511e-06 -7.0148e-05 -7.3936e-05 8.569e-05 8.0317e-06 -0.00010232 3.0719e-05 3.0941e-05 -2.0975e-05 -0.00015209 0.00015771 -0.00013927 0.0002106 6.6334e-05 0.00014686

1846.6 5.6538e-05 3.1424e-05 7.3987e-05 5.8888e-05 5.6237e-05 6.4511e-05 5.2399e-05 6.643e-05 4.669e-05 6.3447e-05 7.3388e-05 6.7845e-05 6.1771e-05 6.7464e-05 3.7113e-05 4.8022e-05 6.4498e-05 7.1406e-05 7.3742e-05 4.9931e-05 5.9745e-05 4.6422e-05 4.1444e-05 7.7808e-05 7.655e-05 7.1992e-05 0.00010319 0.00012527 0.00010919 0.00025861 0.00013219 4.1816e-05 4.8705e-05 0.00010802 0.00012117 6.8238e-05 0.00013566 3.7611e-05 8.8668e-06 7.0843e-05 4.0078e-05 8.3838e-05 2.1577e-05 1.1334e-05 9.6978e-05 0.00018748 0.00011316 3.2333e-05 8.0503e-05 -6.3579e-06 3.2019e-05 9.4533e-05 -2.5983e-05 8.8805e-05 6.3375e-05 -5.0073e-05 -0.00024428 7.7592e-06 -1.1051e-05 0.00017511 2.8326e-05 4.8209e-05

1853.5 0.00010239 8.108e-05 9.9854e-05 8.8711e-05 7.7568e-05 7.7242e-05 8.4736e-05 7.1081e-05 8.6112e-05 9.5801e-05 8.7334e-05 9.0424e-05 7.7509e-05 9.1359e-05 5.3551e-05 7.2541e-05 8.0865e-05 8.1452e-05 9.3242e-05 6.8849e-05 6.7888e-05 6.153e-05 5.8853e-05 7.8617e-05 7.2976e-05 6.0607e-05 0.00010419 0.00010425 8.4649e-05 0.00012235 8.8527e-06 6.2442e-05 0.0001002 0.0002455 0.0001183 0.0001139 0.00014301 8.6487e-05 1.2334e-05 3.8615e-05 5.0441e-05 7.988e-05 8.9579e-06 -6.2624e-07 1.3527e-05 0.00014494 0.00014015 4.6186e-05 5.2729e-05 7.6142e-05 6.396e-05 0.00012614 4.5469e-05 0.00016692 9.6825e-05 -5.5776e-05 -7.8026e-05 -1.8146e-05 9.1955e-05 0.00014674 2.8749e-05 2.2465e-06

1860.5 0.00011545 0.00010274 9.9856e-05 9.9618e-05 8.7625e-05 8.1169e-05

Fig. 4a

0.3 -0.92291 -0.90332

0.35 -0.88344 -0.8666

0.401 -0.8305 -0.82257

0.45 -0.77718 -0.77548

0.5 -0.71123 -0.71306

0.6 -0.58165 -0.58206

0.7 -0.49077 -0.48708

0.8 -0.41912 -0.41851

0.9 -0.34808 -0.35113

1 -0.28468 -0.29042

1.1 -0.24169 -0.24743

1.2 -0.2073 -0.21026

1.3 -0.17568 -0.17926

1.4 -0.15062 -0.15387

1.5 -0.1312 -0.13346

1.75 -0.09596 -0.09839

2 -0.07251 -0.07426

2.25 -0.05799 -0.05951

2.5 -0.04707 -0.04916

2.75 -0.03943 -0.04053

3 -0.03421 -0.03569

3.5 -0.02635 -0.02716

4 -0.02101 -0.02109

4.5 -0.0177 -0.01753

5 -0.01549 -0.01541

6 -0.01093 -0.01299

7 -0.00962 -0.0092

8.5 -0.00679 -0.00524

10 -0.00605 -0.00445

Fig. 4b

1 -0.89479 -0.8445

1.1 -0.85223 -0.83411

1.2 -0.73398 -0.76246

1.3 -0.69955 -0.66906

1.4 -0.67698 -0.62633

1.5 -0.60132 -0.5719

1.75 -0.49538 -0.43903

2 -0.42174 -0.38484

2.25 -0.35848 -0.32938

2.5 -0.25375 -0.26857

2.75 -0.25331 -0.26221

3 -0.21608 -0.18902

3.5 -0.119 -0.1212

4 -0.12669 -0.14892

4.5 -0.0807 -0.1244

5 -0.09422 -0.06773

6 -0.04368 -0.01803

7 -0.01049 -0.02896

8.5 -0.0383 -0.02655

10 -0.05177 -0.05471

Fig. 5a

1 -0.49149 1 -0.4492

1.08842 -0.48576 1.2 -0.45151

1.17684 -0.48011 1.4 -0.45446

1.26526 -0.47454 1.6 -0.44873

1.35369 -0.46904 1.85 -0.44568

1.44211 -0.46361 2.1 -0.44057

1.53053 -0.45826 2.35 -0.43301

1.61895 -0.45298 2.6 -0.42441

1.70737 -0.44777 2.85 -0.41096

1.79579 -0.44263 3.1 -0.39815

1.88421 -0.43756 3.35 -0.38079

1.97264 -0.43256 3.75 -0.36112

2.06106 -0.42763 4.15 -0.33559

2.14948 -0.42276 4.55 -0.3138

2.2379 -0.41796 4.95 -0.29518

2.32632 -0.41322 5.35 -0.28

2.41474 -0.40854 5.75 -0.26228

2.50316 -0.40393 6.15 -0.24704

2.59159 -0.39938 6.55 -0.22563

2.68001 -0.39489 7.638 -0.20733

2.76843 -0.39047 8.907 -0.18263

2.85685 -0.3861 10.387 -0.15856

2.94527 -0.38179 12.113 -0.13804

3.03369 -0.37754 14.125 -0.11639

3.12211 -0.37334 16.472 -0.10254

3.21054 -0.3692 19.209 -0.09159

3.29896 -0.36512 22.4 -0.08659

3.38738 -0.36109 26.122 -0.08196

3.4758 -0.35712 30.462 -0.07437

3.56422 -0.3532 35.523 -0.071

3.65264 -0.34933 41.425 -0.06743

3.74106 -0.34552 48.307 -0.06771

3.82949 -0.34175 56.333 -0.06549

3.91791 -0.33804 65.692 -0.06486

4.00633 -0.33437 76.606 -0.06633

4.09475 -0.33076 89.333 -0.06726

4.18317 -0.32719

4.27159 -0.32368

4.36001 -0.32021

4.44844 -0.31678

4.53686 -0.3134

4.62528 -0.31007

4.7137 -0.30678

4.80212 -0.30354

4.89054 -0.30034

4.97896 -0.29718

5.06739 -0.29407

5.15581 -0.291

5.24423 -0.28797

5.33265 -0.28498

5.42107 -0.28203

5.50949 -0.27911

5.59791 -0.27624

5.68634 -0.27341

5.77476 -0.27062

5.86318 -0.26786

5.9516 -0.26514

6.04002 -0.26245

6.12844 -0.25981

6.21686 -0.2572

6.30529 -0.25462

6.39371 -0.25208

6.48213 -0.24957

6.57055 -0.24709

6.65897 -0.24465

6.74739 -0.24225

6.83581 -0.23987

6.92424 -0.23753

7.01266 -0.23521

7.10108 -0.23293

7.1895 -0.23068

7.27792 -0.22846

7.36634 -0.22627

7.45476 -0.22411

7.54319 -0.22198

7.63161 -0.21987

7.72003 -0.2178

7.80845 -0.21575

7.89687 -0.21373

7.98529 -0.21174

8.07371 -0.20977

8.16214 -0.20784

8.25056 -0.20592

8.33898 -0.20404

8.4274 -0.20217

8.51582 -0.20034

8.60424 -0.19852

8.69266 -0.19674

8.78109 -0.19497

8.86951 -0.19323

8.95793 -0.19152

9.04635 -0.18982

9.13477 -0.18815

9.22319 -0.1865

9.31161 -0.18488

9.40004 -0.18327

9.48846 -0.18169

9.57688 -0.18013

9.6653 -0.17859

9.75372 -0.17707

9.84214 -0.17557

9.93056 -0.17409

10.01898 -0.17263

10.10741 -0.17119

10.19583 -0.16977

10.28425 -0.16837

10.37267 -0.16698

10.46109 -0.16562

10.54951 -0.16427

10.63793 -0.16295

10.72636 -0.16164

10.81478 -0.16035

10.9032 -0.15907

10.99162 -0.15781

11.08004 -0.15657

11.16846 -0.15535

11.25688 -0.15414

11.34531 -0.15295

11.43373 -0.15178

11.52215 -0.15062

11.61057 -0.14947

11.69899 -0.14834

11.78741 -0.14723

11.87583 -0.14613

11.96426 -0.14505

12.05268 -0.14398

12.1411 -0.14292

12.22952 -0.14188

12.31794 -0.14086

12.40636 -0.13985

12.49478 -0.13885

12.58321 -0.13786

12.67163 -0.13689

12.76005 -0.13593

12.84847 -0.13498

12.93689 -0.13405

13.02531 -0.13313

13.11373 -0.13222

13.20216 -0.13132

13.29058 -0.13044

13.379 -0.12957

13.46742 -0.1287

13.55584 -0.12786

13.64426 -0.12702

13.73268 -0.12619

13.82111 -0.12538

13.90953 -0.12457

13.99795 -0.12378

14.08637 -0.12299

14.17479 -0.12222

14.26321 -0.12146

14.35163 -0.12071

14.44006 -0.11997

14.52848 -0.11923

14.6169 -0.11851

14.70532 -0.1178

14.79374 -0.1171

14.88216 -0.1164

14.97058 -0.11572

15.05901 -0.11505

15.14743 -0.11438

15.23585 -0.11372

15.32427 -0.11308

15.41269 -0.11244

15.50111 -0.11181

15.58953 -0.11118

15.67796 -0.11057

15.76638 -0.10997

15.8548 -0.10937

15.94322 -0.10878

16.03164 -0.1082

16.12006 -0.10762

16.20848 -0.10706

16.29691 -0.1065

16.38533 -0.10595

16.47375 -0.10541

16.56217 -0.10487

16.65059 -0.10434

16.73901 -0.10382

16.82743 -0.1033

16.91586 -0.1028

17.00428 -0.1023

17.0927 -0.1018

17.18112 -0.10131

17.26954 -0.10083

17.35796 -0.10036

17.44638 -0.09989

17.53481 -0.09943

17.62323 -0.09897

17.71165 -0.09852

17.80007 -0.09808

17.88849 -0.09764

17.97691 -0.09721

18.06533 -0.09679

18.15376 -0.09637

18.24218 -0.09595

18.3306 -0.09554

18.41902 -0.09514

18.50744 -0.09474

18.59586 -0.09435

18.68428 -0.09396

18.77271 -0.09358

18.86113 -0.0932

18.94955 -0.09283

19.03797 -0.09246

19.12639 -0.0921

19.21481 -0.09174

19.30323 -0.09139

19.39166 -0.09104

19.48008 -0.0907

19.5685 -0.09036

19.65692 -0.09003

19.74534 -0.0897

19.83376 -0.08937

19.92218 -0.08905

20.01061 -0.08874

20.09903 -0.08843

20.18745 -0.08812

20.27587 -0.08782

20.36429 -0.08752

20.45271 -0.08722

20.54113 -0.08693

20.62956 -0.08664

20.71798 -0.08636

20.8064 -0.08608

20.89482 -0.0858

20.98324 -0.08553

21.07166 -0.08526

21.16008 -0.085

21.24851 -0.08473

21.33693 -0.08448

21.42535 -0.08422

21.51377 -0.08397

21.60219 -0.08372

21.69061 -0.08348

21.77903 -0.08324

21.86746 -0.083

21.95588 -0.08276

22.0443 -0.08253

22.13272 -0.0823

22.22114 -0.08208

22.30956 -0.08186

22.39798 -0.08164

22.48641 -0.08142

22.57483 -0.08121

22.66325 -0.081

22.75167 -0.08079

22.84009 -0.08058

22.92851 -0.08038

23.01693 -0.08018

23.10536 -0.07998

23.19378 -0.07979

23.2822 -0.0796

23.37062 -0.07941

23.45904 -0.07922

23.54746 -0.07904

23.63588 -0.07886

23.72431 -0.07868

23.81273 -0.0785

23.90115 -0.07833

23.98957 -0.07816

24.07799 -0.07799

24.16641 -0.07782

24.25483 -0.07765

24.34326 -0.07749

24.43168 -0.07733

24.5201 -0.07717

24.60852 -0.07702

24.69694 -0.07686

24.78536 -0.07671

24.87378 -0.07656

24.96221 -0.07641

25.05063 -0.07626

25.13905 -0.07612

25.22747 -0.07598

25.31589 -0.07584

25.40431 -0.0757

25.49273 -0.07556

25.58116 -0.07543

25.66958 -0.07529

25.758 -0.07516

25.84642 -0.07503

25.93484 -0.07491

26.02326 -0.07478

26.11168 -0.07466

26.20011 -0.07453

26.28853 -0.07441

26.37695 -0.07429

26.46537 -0.07418

26.55379 -0.07406

26.64221 -0.07395

26.73063 -0.07383

26.81906 -0.07372

26.90748 -0.07361

26.9959 -0.0735

27.08432 -0.0734

27.17274 -0.07329

27.26116 -0.07319

27.34958 -0.07308

27.43801 -0.07298

27.52643 -0.07288

27.61485 -0.07278

27.70327 -0.07268

27.79169 -0.07259

27.88011 -0.07249

27.96853 -0.0724

28.05695 -0.07231

28.14538 -0.07222

28.2338 -0.07213

28.32222 -0.07204

28.41064 -0.07195

28.49906 -0.07187

28.58748 -0.07178

28.6759 -0.0717

28.76433 -0.07161

28.85275 -0.07153

28.94117 -0.07145

29.02959 -0.07137

29.11801 -0.07129

29.20643 -0.07122

29.29485 -0.07114

29.38328 -0.07106

29.4717 -0.07099

29.56012 -0.07092

29.64854 -0.07084

29.73696 -0.07077

29.82538 -0.0707

29.9138 -0.07063

30.00223 -0.07057

30.09065 -0.0705

30.17907 -0.07043

30.26749 -0.07037

30.35591 -0.0703

30.44433 -0.07024

30.53275 -0.07017

30.62118 -0.07011

30.7096 -0.07005

30.79802 -0.06999

30.88644 -0.06993

30.97486 -0.06987

31.06328 -0.06981

31.1517 -0.06975

31.24013 -0.0697

31.32855 -0.06964

31.41697 -0.06959

31.50539 -0.06953

31.59381 -0.06948

31.68223 -0.06943

31.77065 -0.06937

31.85908 -0.06932

31.9475 -0.06927

32.03592 -0.06922

32.12434 -0.06917

32.21276 -0.06912

32.30118 -0.06907

32.3896 -0.06903

32.47803 -0.06898

32.56645 -0.06893

32.65487 -0.06889

32.74329 -0.06884

32.83171 -0.0688

32.92013 -0.06875

33.00855 -0.06871

33.09698 -0.06867

33.1854 -0.06863

33.27382 -0.06859

33.36224 -0.06854

33.45066 -0.0685

33.53908 -0.06846

33.6275 -0.06842

33.71593 -0.06839

33.80435 -0.06835

33.89277 -0.06831

33.98119 -0.06827

34.06961 -0.06824

34.15803 -0.0682

34.24645 -0.06816

34.33488 -0.06813

34.4233 -0.06809

34.51172 -0.06806

34.60014 -0.06803

34.68856 -0.06799

34.77698 -0.06796

34.8654 -0.06793

34.95383 -0.06789

35.04225 -0.06786

35.13067 -0.06783

35.21909 -0.0678

35.30751 -0.06777

35.39593 -0.06774

35.48435 -0.06771

35.57278 -0.06768

35.6612 -0.06765

35.74962 -0.06762

35.83804 -0.0676

35.92646 -0.06757

36.01488 -0.06754

36.1033 -0.06751

36.19173 -0.06749

36.28015 -0.06746

36.36857 -0.06744

36.45699 -0.06741

36.54541 -0.06739

36.63383 -0.06736

36.72225 -0.06734

36.81068 -0.06731

36.8991 -0.06729

36.98752 -0.06726

37.07594 -0.06724

37.16436 -0.06722

37.25278 -0.0672

37.3412 -0.06717

37.42963 -0.06715

37.51805 -0.06713

37.60647 -0.06711

37.69489 -0.06709

37.78331 -0.06707

37.87173 -0.06705

37.96015 -0.06703

38.04858 -0.06701

38.137 -0.06699

38.22542 -0.06697

38.31384 -0.06695

38.40226 -0.06693

38.49068 -0.06691

38.5791 -0.06689

38.66753 -0.06687

38.75595 -0.06686

38.84437 -0.06684

38.93279 -0.06682

39.02121 -0.0668

39.10963 -0.06679

39.19805 -0.06677

39.28648 -0.06675

39.3749 -0.06674

39.46332 -0.06672

39.55174 -0.0667

39.64016 -0.06669

39.72858 -0.06667

39.817 -0.06666

39.90543 -0.06664

39.99385 -0.06663

40.08227 -0.06661

40.17069 -0.0666

40.25911 -0.06658

40.34753 -0.06657

40.43595 -0.06656

40.52438 -0.06654

40.6128 -0.06653

40.70122 -0.06652

40.78964 -0.0665

40.87806 -0.06649

40.96648 -0.06648

41.0549 -0.06646

41.14333 -0.06645

41.23175 -0.06644

41.32017 -0.06643

41.40859 -0.06642

41.49701 -0.0664

41.58543 -0.06639

41.67385 -0.06638

41.76228 -0.06637

41.8507 -0.06636

41.93912 -0.06635

42.02754 -0.06634

42.11596 -0.06633

42.20438 -0.06632

42.2928 -0.06631

42.38123 -0.06629

42.46965 -0.06628

42.55807 -0.06627

42.64649 -0.06626

42.73491 -0.06626

42.82333 -0.06625

42.91175 -0.06624

43.00018 -0.06623

43.0886 -0.06622

43.17702 -0.06621

43.26544 -0.0662

43.35386 -0.06619

43.44228 -0.06618

43.5307 -0.06617

43.61913 -0.06616

43.70755 -0.06616

43.79597 -0.06615

43.88439 -0.06614

43.97281 -0.06613

44.06123 -0.06612

44.14965 -0.06612

44.23808 -0.06611

44.3265 -0.0661

44.41492 -0.06609

44.50334 -0.06609

44.59176 -0.06608

44.68018 -0.06607

44.7686 -0.06606

44.85703 -0.06606

44.94545 -0.06605

45.03387 -0.06604

45.12229 -0.06604

45.21071 -0.06603

45.29913 -0.06602

45.38755 -0.06602

45.47597 -0.06601

45.5644 -0.066

45.65282 -0.066

45.74124 -0.06599

45.82966 -0.06599

45.91808 -0.06598

46.0065 -0.06597

46.09492 -0.06597

46.18335 -0.06596

46.27177 -0.06596

46.36019 -0.06595

46.44861 -0.06594

46.53703 -0.06594

46.62545 -0.06593

46.71387 -0.06593

46.8023 -0.06592

46.89072 -0.06592

46.97914 -0.06591

47.06756 -0.06591

47.15598 -0.0659

47.2444 -0.0659

47.33282 -0.06589

47.42125 -0.06589

47.50967 -0.06588

47.59809 -0.06588

47.68651 -0.06587

47.77493 -0.06587

47.86335 -0.06587

47.95177 -0.06586

48.0402 -0.06586

48.12862 -0.06585

48.21704 -0.06585

48.30546 -0.06584

48.39388 -0.06584

48.4823 -0.06584

48.57072 -0.06583

48.65915 -0.06583

48.74757 -0.06582

48.83599 -0.06582

48.92441 -0.06582

49.01283 -0.06581

49.10125 -0.06581

49.18967 -0.06581

49.2781 -0.0658

49.36652 -0.0658

49.45494 -0.0658

49.54336 -0.06579

49.63178 -0.06579

49.7202 -0.06579

49.80862 -0.06578

49.89705 -0.06578

49.98547 -0.06578

50.07389 -0.06577

50.16231 -0.06577

50.25073 -0.06577

50.33915 -0.06576

50.42757 -0.06576

50.516 -0.06576

50.60442 -0.06575

50.69284 -0.06575

50.78126 -0.06575

50.86968 -0.06575

50.9581 -0.06574

51.04652 -0.06574

51.13495 -0.06574

51.22337 -0.06573

51.31179 -0.06573

51.40021 -0.06573

51.48863 -0.06573

51.57705 -0.06572

51.66547 -0.06572

51.7539 -0.06572

51.84232 -0.06572

51.93074 -0.06571

52.01916 -0.06571

52.10758 -0.06571

52.196 -0.06571

52.28442 -0.06571

52.37285 -0.0657

52.46127 -0.0657

52.54969 -0.0657

52.63811 -0.0657

52.72653 -0.06569

52.81495 -0.06569

52.90337 -0.06569

52.9918 -0.06569

53.08022 -0.06569

53.16864 -0.06568

53.25706 -0.06568

53.34548 -0.06568

53.4339 -0.06568

53.52232 -0.06568

53.61075 -0.06567

53.69917 -0.06567

53.78759 -0.06567

53.87601 -0.06567

53.96443 -0.06567

54.05285 -0.06567

54.14127 -0.06566

54.2297 -0.06566

54.31812 -0.06566

54.40654 -0.06566

54.49496 -0.06566

54.58338 -0.06566

54.6718 -0.06565

54.76022 -0.06565

54.84865 -0.06565

54.93707 -0.06565

55.02549 -0.06565

55.11391 -0.06565

55.20233 -0.06565

55.29075 -0.06564

55.37917 -0.06564

55.4676 -0.06564

55.55602 -0.06564

55.64444 -0.06564

55.73286 -0.06564

55.82128 -0.06564

55.9097 -0.06563

55.99812 -0.06563

56.08655 -0.06563

56.17497 -0.06563

56.26339 -0.06563

56.35181 -0.06563

56.44023 -0.06563

56.52865 -0.06563

56.61707 -0.06562

56.7055 -0.06562

56.79392 -0.06562

56.88234 -0.06562

56.97076 -0.06562

57.05918 -0.06562

57.1476 -0.06562

57.23602 -0.06562

57.32445 -0.06562

57.41287 -0.06562

57.50129 -0.06561

57.58971 -0.06561

57.67813 -0.06561

57.76655 -0.06561

57.85497 -0.06561

57.9434 -0.06561

58.03182 -0.06561

58.12024 -0.06561

58.20866 -0.06561

58.29708 -0.06561

58.3855 -0.0656

58.47392 -0.0656

58.56235 -0.0656

58.65077 -0.0656

58.73919 -0.0656

58.82761 -0.0656

58.91603 -0.0656

59.00445 -0.0656

59.09287 -0.0656

59.1813 -0.0656

59.26972 -0.0656

59.35814 -0.0656

59.44656 -0.06559

59.53498 -0.06559

59.6234 -0.06559

59.71182 -0.06559

59.80025 -0.06559

59.88867 -0.06559

59.97709 -0.06559

60.06551 -0.06559

60.15393 -0.06559

60.24235 -0.06559

60.33077 -0.06559

60.4192 -0.06559

60.50762 -0.06559

60.59604 -0.06559

60.68446 -0.06559

60.77288 -0.06558

60.8613 -0.06558

60.94972 -0.06558

61.03815 -0.06558

61.12657 -0.06558

61.21499 -0.06558

61.30341 -0.06558

61.39183 -0.06558

61.48025 -0.06558

61.56867 -0.06558

61.6571 -0.06558

61.74552 -0.06558

61.83394 -0.06558

61.92236 -0.06558

62.01078 -0.06558

62.0992 -0.06558

62.18762 -0.06558

62.27605 -0.06558

62.36447 -0.06557

62.45289 -0.06557

62.54131 -0.06557

62.62973 -0.06557

62.71815 -0.06557

62.80657 -0.06557

62.89499 -0.06557

62.98342 -0.06557

63.07184 -0.06557

63.16026 -0.06557

63.24868 -0.06557

63.3371 -0.06557

63.42552 -0.06557

63.51394 -0.06557

63.60237 -0.06557

63.69079 -0.06557

63.77921 -0.06557

63.86763 -0.06557

63.95605 -0.06557

64.04447 -0.06557

64.13289 -0.06557

64.22132 -0.06557

64.30974 -0.06557

64.39816 -0.06557

64.48658 -0.06557

64.575 -0.06556

64.66342 -0.06556

64.75184 -0.06556

64.84027 -0.06556

64.92869 -0.06556

65.01711 -0.06556

65.10553 -0.06556

65.19395 -0.06556

65.28237 -0.06556

65.37079 -0.06556

65.45922 -0.06556

65.54764 -0.06556

65.63606 -0.06556

65.72448 -0.06556

65.8129 -0.06556

65.90132 -0.06556

65.98974 -0.06556

66.07817 -0.06556

66.16659 -0.06556

66.25501 -0.06556

66.34343 -0.06556

66.43185 -0.06556

66.52027 -0.06556

66.60869 -0.06556

66.69712 -0.06556

66.78554 -0.06556

66.87396 -0.06556

66.96238 -0.06556

67.0508 -0.06556

67.13922 -0.06556

67.22764 -0.06556

67.31607 -0.06556

67.40449 -0.06556

67.49291 -0.06556

67.58133 -0.06556

67.66975 -0.06556

67.75817 -0.06556

67.84659 -0.06555

67.93502 -0.06555

68.02344 -0.06555

68.11186 -0.06555

68.20028 -0.06555

68.2887 -0.06555

68.37712 -0.06555

68.46554 -0.06555

68.55397 -0.06555

68.64239 -0.06555

68.73081 -0.06555

68.81923 -0.06555

68.90765 -0.06555

68.99607 -0.06555

69.08449 -0.06555

69.17292 -0.06555

69.26134 -0.06555

69.34976 -0.06555

69.43818 -0.06555

69.5266 -0.06555

69.61502 -0.06555

69.70344 -0.06555

69.79187 -0.06555

69.88029 -0.06555

69.96871 -0.06555

70.05713 -0.06555

70.14555 -0.06555

70.23397 -0.06555

70.32239 -0.06555

70.41082 -0.06555

70.49924 -0.06555

70.58766 -0.06555

70.67608 -0.06555

70.7645 -0.06555

70.85292 -0.06555

70.94134 -0.06555

71.02977 -0.06555

71.11819 -0.06555

71.20661 -0.06555

71.29503 -0.06555

71.38345 -0.06555

71.47187 -0.06555

71.56029 -0.06555

71.64872 -0.06555

71.73714 -0.06555

71.82556 -0.06555

71.91398 -0.06555

72.0024 -0.06555

72.09082 -0.06555

72.17924 -0.06555

72.26767 -0.06555

72.35609 -0.06555

72.44451 -0.06555

72.53293 -0.06555

72.62135 -0.06555

72.70977 -0.06555

72.79819 -0.06555

72.88662 -0.06555

72.97504 -0.06555

73.06346 -0.06555

73.15188 -0.06555

73.2403 -0.06555

73.32872 -0.06555

73.41714 -0.06555

73.50557 -0.06555

73.59399 -0.06555

73.68241 -0.06555

73.77083 -0.06555

73.85925 -0.06555

73.94767 -0.06555

74.03609 -0.06555

74.12452 -0.06555

74.21294 -0.06555

74.30136 -0.06555

74.38978 -0.06555

74.4782 -0.06555

74.56662 -0.06554

74.65504 -0.06554

74.74347 -0.06554

74.83189 -0.06554

74.92031 -0.06554

75.00873 -0.06554

75.09715 -0.06554

75.18557 -0.06554

75.27399 -0.06554

75.36242 -0.06554

75.45084 -0.06554

75.53926 -0.06554

75.62768 -0.06554

75.7161 -0.06554

75.80452 -0.06554

75.89294 -0.06554

75.98137 -0.06554

76.06979 -0.06554

76.15821 -0.06554

76.24663 -0.06554

76.33505 -0.06554

76.42347 -0.06554

76.51189 -0.06554

76.60032 -0.06554

76.68874 -0.06554

76.77716 -0.06554

76.86558 -0.06554

76.954 -0.06554

77.04242 -0.06554

77.13084 -0.06554

77.21927 -0.06554

77.30769 -0.06554

77.39611 -0.06554

77.48453 -0.06554

77.57295 -0.06554

77.66137 -0.06554

77.74979 -0.06554

77.83822 -0.06554

77.92664 -0.06554

78.01506 -0.06554

78.10348 -0.06554

78.1919 -0.06554

78.28032 -0.06554

78.36874 -0.06554

78.45717 -0.06554

78.54559 -0.06554

78.63401 -0.06554

78.72243 -0.06554

78.81085 -0.06554

78.89927 -0.06554

78.98769 -0.06554

79.07612 -0.06554

79.16454 -0.06554

79.25296 -0.06554

79.34138 -0.06554

79.4298 -0.06554

79.51822 -0.06554

79.60664 -0.06554

79.69507 -0.06554

79.78349 -0.06554

79.87191 -0.06554

79.96033 -0.06554

80.04875 -0.06554

80.13717 -0.06554

80.22559 -0.06554

80.31402 -0.06554

80.40244 -0.06554

80.49086 -0.06554

80.57928 -0.06554

80.6677 -0.06554

80.75612 -0.06554

80.84454 -0.06554

80.93296 -0.06554

81.02139 -0.06554

81.10981 -0.06554

81.19823 -0.06554

81.28665 -0.06554

81.37507 -0.06554

81.46349 -0.06554

81.55191 -0.06554

81.64034 -0.06554

81.72876 -0.06554

81.81718 -0.06554

81.9056 -0.06554

81.99402 -0.06554

82.08244 -0.06554

82.17086 -0.06554

82.25929 -0.06554

82.34771 -0.06554

82.43613 -0.06554

82.52455 -0.06554

82.61297 -0.06554

82.70139 -0.06554

82.78981 -0.06554

82.87824 -0.06554

82.96666 -0.06554

83.05508 -0.06554

83.1435 -0.06554

83.23192 -0.06554

83.32034 -0.06554

83.40876 -0.06554

83.49719 -0.06554

83.58561 -0.06554

83.67403 -0.06554

83.76245 -0.06554

83.85087 -0.06554

83.93929 -0.06554

84.02771 -0.06554

84.11614 -0.06554

84.20456 -0.06554

84.29298 -0.06554

84.3814 -0.06554

84.46982 -0.06554

84.55824 -0.06554

84.64666 -0.06554

84.73509 -0.06554

84.82351 -0.06554

84.91193 -0.06554

85.00035 -0.06554

85.08877 -0.06554

85.17719 -0.06554

85.26561 -0.06554

85.35404 -0.06554

85.44246 -0.06554

85.53088 -0.06554

85.6193 -0.06554

85.70772 -0.06554

85.79614 -0.06554

85.88456 -0.06554

85.97299 -0.06554

86.06141 -0.06554

86.14983 -0.06554

86.23825 -0.06554

86.32667 -0.06554

86.41509 -0.06554

86.50351 -0.06554

86.59194 -0.06554

86.68036 -0.06554

86.76878 -0.06554

86.8572 -0.06554

86.94562 -0.06554

87.03404 -0.06554

87.12246 -0.06554

87.21089 -0.06554

87.29931 -0.06554

87.38773 -0.06554

87.47615 -0.06554

87.56457 -0.06554

87.65299 -0.06554

87.74141 -0.06554

87.82984 -0.06554

87.91826 -0.06554

88.00668 -0.06554

88.0951 -0.06554

88.18352 -0.06554

88.27194 -0.06554

88.36036 -0.06554

88.44879 -0.06554

88.53721 -0.06554

88.62563 -0.06554

88.71405 -0.06554

88.80247 -0.06554

88.89089 -0.06554

88.97931 -0.06554

89.06774 -0.06554

89.15616 -0.06554

89.24458 -0.06554

89.333 -0.06554

Fig. 5b.

1 0.14809 1 0.14858

1.08842 0.14679 1.2 0.13804

1.17684 0.14551 1.4 0.13399

1.26526 0.14423 1.6 0.13333

1.35369 0.14297 1.85 0.13456

1.44211 0.14171 2.1 0.13432

1.53053 0.14048 2.35 0.13042

1.61895 0.13925 2.6 0.1273

1.70737 0.13803 2.85 0.12793

1.79579 0.13683 3.1 0.1244

1.88421 0.13563 3.35 0.12428

1.97264 0.13445 3.75 0.11975

2.06106 0.13328 4.15 0.11361

2.14948 0.13212 4.55 0.10843

2.2379 0.13097 4.95 0.10431

2.32632 0.12984 5.35 0.09819

2.41474 0.12871 5.75 0.09182

2.50316 0.12759 6.15 0.0871

2.59159 0.12649 6.55 0.08341

2.68001 0.12539 7.638 0.07947

2.76843 0.12431 8.907 0.06948

2.85685 0.12324 10.387 0.05683

2.94527 0.12217 12.113 0.04769

3.03369 0.12112 14.125 0.03853

3.12211 0.12008 16.472 0.03441

3.21054 0.11904 19.209 0.02945

3.29896 0.11802 22.4 0.02604

3.38738 0.117 26.122 0.0214

3.4758 0.116 30.462 0.0193

3.56422 0.115 35.523 0.01473

3.65264 0.11402 41.425 0.01458

3.74106 0.11304 48.307 0.01367

3.82949 0.11208 56.333 0.01266

3.91791 0.11112 65.692 0.01113

4.00633 0.11017 76.606 0.0113

4.09475 0.10923 89.333 0.01147

4.18317 0.1083

4.27159 0.10738

4.36001 0.10647

4.44844 0.10556

4.53686 0.10467

4.62528 0.10378

4.7137 0.1029

4.80212 0.10203

4.89054 0.10117

4.97896 0.10031

5.06739 0.09947

5.15581 0.09863

5.24423 0.0978

5.33265 0.09698

5.42107 0.09617

5.50949 0.09536

5.59791 0.09456

5.68634 0.09377

5.77476 0.09299

5.86318 0.09221

5.9516 0.09144

6.04002 0.09068

6.12844 0.08993

6.21686 0.08918

6.30529 0.08844

6.39371 0.08771

6.48213 0.08698

6.57055 0.08626

6.65897 0.08555

6.74739 0.08485

6.83581 0.08415

6.92424 0.08346

7.01266 0.08277

7.10108 0.08209

7.1895 0.08142

7.27792 0.08075

7.36634 0.08009

7.45476 0.07944

7.54319 0.07879

7.63161 0.07815

7.72003 0.07752

7.80845 0.07689

7.89687 0.07627

7.98529 0.07565

8.07371 0.07504

8.16214 0.07443

8.25056 0.07383

8.33898 0.07324

8.4274 0.07265

8.51582 0.07207

8.60424 0.07149

8.69266 0.07092

8.78109 0.07035

8.86951 0.06979

8.95793 0.06924

9.04635 0.06869

9.13477 0.06814

9.22319 0.0676

9.31161 0.06707

9.40004 0.06654

9.48846 0.06602

9.57688 0.0655

9.6653 0.06498

9.75372 0.06447

9.84214 0.06397

9.93056 0.06347

10.01898 0.06297

10.10741 0.06248

10.19583 0.062

10.28425 0.06152

10.37267 0.06104

10.46109 0.06057

10.54951 0.0601

10.63793 0.05964

10.72636 0.05918

10.81478 0.05872

10.9032 0.05827

10.99162 0.05783

11.08004 0.05739

11.16846 0.05695

11.25688 0.05652

11.34531 0.05609

11.43373 0.05566

11.52215 0.05524

11.61057 0.05482

11.69899 0.05441

11.78741 0.054

11.87583 0.0536

11.96426 0.0532

12.05268 0.0528

12.1411 0.05241

12.22952 0.05202

12.31794 0.05163

12.40636 0.05125

12.49478 0.05087

12.58321 0.05049

12.67163 0.05012

12.76005 0.04975

12.84847 0.04939

12.93689 0.04903

13.02531 0.04867

13.11373 0.04832

13.20216 0.04796

13.29058 0.04762

13.379 0.04727

13.46742 0.04693

13.55584 0.04659

13.64426 0.04626

13.73268 0.04593

13.82111 0.0456

13.90953 0.04527

13.99795 0.04495

14.08637 0.04463

14.17479 0.04432

14.26321 0.044

14.35163 0.04369

14.44006 0.04339

14.52848 0.04308

14.6169 0.04278

14.70532 0.04248

14.79374 0.04219

14.88216 0.0419

14.97058 0.04161

15.05901 0.04132

15.14743 0.04103

15.23585 0.04075

15.32427 0.04047

15.41269 0.0402

15.50111 0.03992

15.58953 0.03965

15.67796 0.03938

15.76638 0.03912

15.8548 0.03885

15.94322 0.03859

16.03164 0.03834

16.12006 0.03808

16.20848 0.03783

16.29691 0.03757

16.38533 0.03733

16.47375 0.03708

16.56217 0.03684

16.65059 0.03659

16.73901 0.03635

16.82743 0.03612

16.91586 0.03588

17.00428 0.03565

17.0927 0.03542

17.18112 0.03519

17.26954 0.03496

17.35796 0.03474

17.44638 0.03452

17.53481 0.0343

17.62323 0.03408

17.71165 0.03387

17.80007 0.03365

17.88849 0.03344

17.97691 0.03323

18.06533 0.03302

18.15376 0.03282

18.24218 0.03262

18.3306 0.03241

18.41902 0.03221

18.50744 0.03202

18.59586 0.03182

18.68428 0.03163

18.77271 0.03143

18.86113 0.03124

18.94955 0.03106

19.03797 0.03087

19.12639 0.03068

19.21481 0.0305

19.30323 0.03032

19.39166 0.03014

19.48008 0.02996

19.5685 0.02978

19.65692 0.02961

19.74534 0.02944

19.83376 0.02927

19.92218 0.0291

20.01061 0.02893

20.09903 0.02876

20.18745 0.0286

20.27587 0.02843

20.36429 0.02827

20.45271 0.02811

20.54113 0.02795

20.62956 0.0278

20.71798 0.02764

20.8064 0.02749

20.89482 0.02733

20.98324 0.02718

21.07166 0.02703

21.16008 0.02688

21.24851 0.02674

21.33693 0.02659

21.42535 0.02645

21.51377 0.0263

21.60219 0.02616

21.69061 0.02602

21.77903 0.02588

21.86746 0.02575

21.95588 0.02561

22.0443 0.02547

22.13272 0.02534

22.22114 0.02521

22.30956 0.02508

22.39798 0.02495

22.48641 0.02482

22.57483 0.02469

22.66325 0.02456

22.75167 0.02444

22.84009 0.02432

22.92851 0.02419

23.01693 0.02407

23.10536 0.02395

23.19378 0.02383

23.2822 0.02371

23.37062 0.0236

23.45904 0.02348

23.54746 0.02337

23.63588 0.02325

23.72431 0.02314

23.81273 0.02303

23.90115 0.02292

23.98957 0.02281

24.07799 0.0227

24.16641 0.02259

24.25483 0.02249

24.34326 0.02238

24.43168 0.02228

24.5201 0.02218

24.60852 0.02207

24.69694 0.02197

24.78536 0.02187

24.87378 0.02177

24.96221 0.02167

25.05063 0.02158

25.13905 0.02148

25.22747 0.02138

25.31589 0.02129

25.40431 0.0212

25.49273 0.0211

25.58116 0.02101

25.66958 0.02092

25.758 0.02083

25.84642 0.02074

25.93484 0.02065

26.02326 0.02056

26.11168 0.02048

26.20011 0.02039

26.28853 0.0203

26.37695 0.02022

26.46537 0.02014

26.55379 0.02005

26.64221 0.01997

26.73063 0.01989

26.81906 0.01981

26.90748 0.01973

26.9959 0.01965

27.08432 0.01957

27.17274 0.0195

27.26116 0.01942

27.34958 0.01934

27.43801 0.01927

27.52643 0.01919

27.61485 0.01912

27.70327 0.01905

27.79169 0.01897

27.88011 0.0189

27.96853 0.01883

28.05695 0.01876

28.14538 0.01869

28.2338 0.01862

28.32222 0.01855

28.41064 0.01848

28.49906 0.01842

28.58748 0.01835

28.6759 0.01829

28.76433 0.01822

28.85275 0.01816

28.94117 0.01809

29.02959 0.01803

29.11801 0.01797

29.20643 0.0179

29.29485 0.01784

29.38328 0.01778

29.4717 0.01772

29.56012 0.01766

29.64854 0.0176

29.73696 0.01754

29.82538 0.01748

29.9138 0.01743

30.00223 0.01737

30.09065 0.01731

30.17907 0.01726

30.26749 0.0172

30.35591 0.01715

30.44433 0.01709

30.53275 0.01704

30.62118 0.01698

30.7096 0.01693

30.79802 0.01688

30.88644 0.01683

30.97486 0.01678

31.06328 0.01673

31.1517 0.01667

31.24013 0.01663

31.32855 0.01658

31.41697 0.01653

31.50539 0.01648

31.59381 0.01643

31.68223 0.01638

31.77065 0.01634

31.85908 0.01629

31.9475 0.01624

32.03592 0.0162

32.12434 0.01615

32.21276 0.01611

32.30118 0.01606

32.3896 0.01602

32.47803 0.01597

32.56645 0.01593

32.65487 0.01589

32.74329 0.01585

32.83171 0.0158

32.92013 0.01576

33.00855 0.01572

33.09698 0.01568

33.1854 0.01564

33.27382 0.0156

33.36224 0.01556

33.45066 0.01552

33.53908 0.01548

33.6275 0.01544

33.71593 0.0154

33.80435 0.01537

33.89277 0.01533

33.98119 0.01529

34.06961 0.01526

34.15803 0.01522

34.24645 0.01518

34.33488 0.01515

34.4233 0.01511

34.51172 0.01508

34.60014 0.01504

34.68856 0.01501

34.77698 0.01497

34.8654 0.01494

34.95383 0.01491

35.04225 0.01487

35.13067 0.01484

35.21909 0.01481

35.30751 0.01478

35.39593 0.01474

35.48435 0.01471

35.57278 0.01468

35.6612 0.01465

35.74962 0.01462

35.83804 0.01459

35.92646 0.01456

36.01488 0.01453

36.1033 0.0145

36.19173 0.01447

36.28015 0.01444

36.36857 0.01441

36.45699 0.01438

36.54541 0.01436

36.63383 0.01433

36.72225 0.0143

36.81068 0.01427

36.8991 0.01425

36.98752 0.01422

37.07594 0.01419

37.16436 0.01417

37.25278 0.01414

37.3412 0.01411

37.42963 0.01409

37.51805 0.01406

37.60647 0.01404

37.69489 0.01401

37.78331 0.01399

37.87173 0.01397

37.96015 0.01394

38.04858 0.01392

38.137 0.01389

38.22542 0.01387

38.31384 0.01385

38.40226 0.01382

38.49068 0.0138

38.5791 0.01378

38.66753 0.01376

38.75595 0.01373

38.84437 0.01371

38.93279 0.01369

39.02121 0.01367

39.10963 0.01365

39.19805 0.01363

39.28648 0.01361

39.3749 0.01358

39.46332 0.01356

39.55174 0.01354

39.64016 0.01352

39.72858 0.0135

39.817 0.01348

39.90543 0.01346

39.99385 0.01344

40.08227 0.01343

40.17069 0.01341

40.25911 0.01339

40.34753 0.01337

40.43595 0.01335

40.52438 0.01333

40.6128 0.01331

40.70122 0.0133

40.78964 0.01328

40.87806 0.01326

40.96648 0.01324

41.0549 0.01323

41.14333 0.01321

41.23175 0.01319

41.32017 0.01318

41.40859 0.01316

41.49701 0.01314

41.58543 0.01313

41.67385 0.01311

41.76228 0.01309

41.8507 0.01308

41.93912 0.01306

42.02754 0.01305

42.11596 0.01303

42.20438 0.01302

42.2928 0.013

42.38123 0.01299

42.46965 0.01297

42.55807 0.01296

42.64649 0.01294

42.73491 0.01293

42.82333 0.01291

42.91175 0.0129

43.00018 0.01289

43.0886 0.01287

43.17702 0.01286

43.26544 0.01284

43.35386 0.01283

43.44228 0.01282

43.5307 0.0128

43.61913 0.01279

43.70755 0.01278

43.79597 0.01276

43.88439 0.01275

43.97281 0.01274

44.06123 0.01273

44.14965 0.01271

44.23808 0.0127

44.3265 0.01269

44.41492 0.01268

44.50334 0.01267

44.59176 0.01265

44.68018 0.01264

44.7686 0.01263

44.85703 0.01262

44.94545 0.01261

45.03387 0.0126

45.12229 0.01259

45.21071 0.01258

45.29913 0.01256

45.38755 0.01255

45.47597 0.01254

45.5644 0.01253

45.65282 0.01252

45.74124 0.01251

45.82966 0.0125

45.91808 0.01249

46.0065 0.01248

46.09492 0.01247

46.18335 0.01246

46.27177 0.01245

46.36019 0.01244

46.44861 0.01243

46.53703 0.01242

46.62545 0.01241

46.71387 0.0124

46.8023 0.01239

46.89072 0.01238

46.97914 0.01238

47.06756 0.01237

47.15598 0.01236

47.2444 0.01235

47.33282 0.01234

47.42125 0.01233

47.50967 0.01232

47.59809 0.01231

47.68651 0.01231

47.77493 0.0123

47.86335 0.01229

47.95177 0.01228

48.0402 0.01227

48.12862 0.01226

48.21704 0.01226

48.30546 0.01225

48.39388 0.01224

48.4823 0.01223

48.57072 0.01223

48.65915 0.01222

48.74757 0.01221

48.83599 0.0122

48.92441 0.0122

49.01283 0.01219

49.10125 0.01218

49.18967 0.01217

49.2781 0.01217

49.36652 0.01216

49.45494 0.01215

49.54336 0.01215

49.63178 0.01214

49.7202 0.01213

49.80862 0.01213

49.89705 0.01212

49.98547 0.01211

50.07389 0.01211

50.16231 0.0121

50.25073 0.01209

50.33915 0.01209

50.42757 0.01208

50.516 0.01207

50.60442 0.01207

50.69284 0.01206

50.78126 0.01206

50.86968 0.01205

50.9581 0.01204

51.04652 0.01204

51.13495 0.01203

51.22337 0.01203

51.31179 0.01202

51.40021 0.01202

51.48863 0.01201

51.57705 0.012

51.66547 0.012

51.7539 0.01199

51.84232 0.01199

51.93074 0.01198

52.01916 0.01198

52.10758 0.01197

52.196 0.01197

52.28442 0.01196

52.37285 0.01196

52.46127 0.01195

52.54969 0.01195

52.63811 0.01194

52.72653 0.01194

52.81495 0.01193

52.90337 0.01193

52.9918 0.01192

53.08022 0.01192

53.16864 0.01191

53.25706 0.01191

53.34548 0.0119

53.4339 0.0119

53.52232 0.01189

53.61075 0.01189

53.69917 0.01189

53.78759 0.01188

53.87601 0.01188

53.96443 0.01187

54.05285 0.01187

54.14127 0.01186

54.2297 0.01186

54.31812 0.01186

54.40654 0.01185

54.49496 0.01185

54.58338 0.01184

54.6718 0.01184

54.76022 0.01184

54.84865 0.01183

54.93707 0.01183

55.02549 0.01182

55.11391 0.01182

55.20233 0.01182

55.29075 0.01181

55.37917 0.01181

55.4676 0.01181

55.55602 0.0118

55.64444 0.0118

55.73286 0.0118

55.82128 0.01179

55.9097 0.01179

55.99812 0.01179

56.08655 0.01178

56.17497 0.01178

56.26339 0.01178

56.35181 0.01177

56.44023 0.01177

56.52865 0.01177

56.61707 0.01176

56.7055 0.01176

56.79392 0.01176

56.88234 0.01175

56.97076 0.01175

57.05918 0.01175

57.1476 0.01174

57.23602 0.01174

57.32445 0.01174

57.41287 0.01173

57.50129 0.01173

57.58971 0.01173

57.67813 0.01173

57.76655 0.01172

57.85497 0.01172

57.9434 0.01172

58.03182 0.01171

58.12024 0.01171

58.20866 0.01171

58.29708 0.01171

58.3855 0.0117

58.47392 0.0117

58.56235 0.0117

58.65077 0.0117

58.73919 0.01169

58.82761 0.01169

58.91603 0.01169

59.00445 0.01169

59.09287 0.01168

59.1813 0.01168

59.26972 0.01168

59.35814 0.01168

59.44656 0.01167

59.53498 0.01167

59.6234 0.01167

59.71182 0.01167

59.80025 0.01166

59.88867 0.01166

59.97709 0.01166

60.06551 0.01166

60.15393 0.01166

60.24235 0.01165

60.33077 0.01165

60.4192 0.01165

60.50762 0.01165

60.59604 0.01165

60.68446 0.01164

60.77288 0.01164

60.8613 0.01164

60.94972 0.01164

61.03815 0.01163

61.12657 0.01163

61.21499 0.01163

61.30341 0.01163

61.39183 0.01163

61.48025 0.01163

61.56867 0.01162

61.6571 0.01162

61.74552 0.01162

61.83394 0.01162

61.92236 0.01162

62.01078 0.01161

62.0992 0.01161

62.18762 0.01161

62.27605 0.01161

62.36447 0.01161

62.45289 0.01161

62.54131 0.0116

62.62973 0.0116

62.71815 0.0116

62.80657 0.0116

62.89499 0.0116

62.98342 0.0116

63.07184 0.01159

63.16026 0.01159

63.24868 0.01159

63.3371 0.01159

63.42552 0.01159

63.51394 0.01159

63.60237 0.01158

63.69079 0.01158

63.77921 0.01158

63.86763 0.01158

63.95605 0.01158

64.04447 0.01158

64.13289 0.01158

64.22132 0.01157

64.30974 0.01157

64.39816 0.01157

64.48658 0.01157

64.575 0.01157

64.66342 0.01157

64.75184 0.01157

64.84027 0.01157

64.92869 0.01156

65.01711 0.01156

65.10553 0.01156

65.19395 0.01156

65.28237 0.01156

65.37079 0.01156

65.45922 0.01156

65.54764 0.01155

65.63606 0.01155

65.72448 0.01155

65.8129 0.01155

65.90132 0.01155

65.98974 0.01155

66.07817 0.01155

66.16659 0.01155

66.25501 0.01155

66.34343 0.01154

66.43185 0.01154

66.52027 0.01154

66.60869 0.01154

66.69712 0.01154

66.78554 0.01154

66.87396 0.01154

66.96238 0.01154

67.0508 0.01154

67.13922 0.01153

67.22764 0.01153

67.31607 0.01153

67.40449 0.01153

67.49291 0.01153

67.58133 0.01153

67.66975 0.01153

67.75817 0.01153

67.84659 0.01153

67.93502 0.01153

68.02344 0.01153

68.11186 0.01152

68.20028 0.01152

68.2887 0.01152

68.37712 0.01152

68.46554 0.01152

68.55397 0.01152

68.64239 0.01152

68.73081 0.01152

68.81923 0.01152

68.90765 0.01152

68.99607 0.01152

69.08449 0.01151

69.17292 0.01151

69.26134 0.01151

69.34976 0.01151

69.43818 0.01151

69.5266 0.01151

69.61502 0.01151

69.70344 0.01151

69.79187 0.01151

69.88029 0.01151

69.96871 0.01151

70.05713 0.01151

70.14555 0.01151

70.23397 0.0115

70.32239 0.0115

70.41082 0.0115

70.49924 0.0115

70.58766 0.0115

70.67608 0.0115

70.7645 0.0115

70.85292 0.0115

70.94134 0.0115

71.02977 0.0115

71.11819 0.0115

71.20661 0.0115

71.29503 0.0115

71.38345 0.0115

71.47187 0.01149

71.56029 0.01149

71.64872 0.01149

71.73714 0.01149

71.82556 0.01149

71.91398 0.01149

72.0024 0.01149

72.09082 0.01149

72.17924 0.01149

72.26767 0.01149

72.35609 0.01149

72.44451 0.01149

72.53293 0.01149

72.62135 0.01149

72.70977 0.01149

72.79819 0.01149

72.88662 0.01149

72.97504 0.01148

73.06346 0.01148

73.15188 0.01148

73.2403 0.01148

73.32872 0.01148

73.41714 0.01148

73.50557 0.01148

73.59399 0.01148

73.68241 0.01148

73.77083 0.01148

73.85925 0.01148

73.94767 0.01148

74.03609 0.01148

74.12452 0.01148

74.21294 0.01148

74.30136 0.01148

74.38978 0.01148

74.4782 0.01148

74.56662 0.01148

74.65504 0.01148

74.74347 0.01147

74.83189 0.01147

74.92031 0.01147

75.00873 0.01147

75.09715 0.01147

75.18557 0.01147

75.27399 0.01147

75.36242 0.01147

75.45084 0.01147

75.53926 0.01147

75.62768 0.01147

75.7161 0.01147

75.80452 0.01147

75.89294 0.01147

75.98137 0.01147

76.06979 0.01147

76.15821 0.01147

76.24663 0.01147

76.33505 0.01147

76.42347 0.01147

76.51189 0.01147

76.60032 0.01147

76.68874 0.01147

76.77716 0.01147

76.86558 0.01147

76.954 0.01146

77.04242 0.01146

77.13084 0.01146

77.21927 0.01146

77.30769 0.01146

77.39611 0.01146

77.48453 0.01146

77.57295 0.01146

77.66137 0.01146

77.74979 0.01146

77.83822 0.01146

77.92664 0.01146

78.01506 0.01146

78.10348 0.01146

78.1919 0.01146

78.28032 0.01146

78.36874 0.01146

78.45717 0.01146

78.54559 0.01146

78.63401 0.01146

78.72243 0.01146

78.81085 0.01146

78.89927 0.01146

78.98769 0.01146

79.07612 0.01146

79.16454 0.01146

79.25296 0.01146

79.34138 0.01146

79.4298 0.01146

79.51822 0.01146

79.60664 0.01146

79.69507 0.01146

79.78349 0.01145

79.87191 0.01145

79.96033 0.01145

80.04875 0.01145

80.13717 0.01145

80.22559 0.01145

80.31402 0.01145

80.40244 0.01145

80.49086 0.01145

80.57928 0.01145

80.6677 0.01145

80.75612 0.01145

80.84454 0.01145

80.93296 0.01145

81.02139 0.01145

81.10981 0.01145

81.19823 0.01145

81.28665 0.01145

81.37507 0.01145

81.46349 0.01145

81.55191 0.01145

81.64034 0.01145

81.72876 0.01145

81.81718 0.01145

81.9056 0.01145

81.99402 0.01145

82.08244 0.01145

82.17086 0.01145

82.25929 0.01145

82.34771 0.01145

82.43613 0.01145

82.52455 0.01145

82.61297 0.01145

82.70139 0.01145

82.78981 0.01145

82.87824 0.01145

82.96666 0.01145

83.05508 0.01145

83.1435 0.01145

83.23192 0.01145

83.32034 0.01145

83.40876 0.01145

83.49719 0.01145

83.58561 0.01145

83.67403 0.01145

83.76245 0.01145

83.85087 0.01145

83.93929 0.01145

84.02771 0.01144

84.11614 0.01144

84.20456 0.01144

84.29298 0.01144

84.3814 0.01144

84.46982 0.01144

84.55824 0.01144

84.64666 0.01144

84.73509 0.01144

84.82351 0.01144

84.91193 0.01144

85.00035 0.01144

85.08877 0.01144

85.17719 0.01144

85.26561 0.01144

85.35404 0.01144

85.44246 0.01144

85.53088 0.01144

85.6193 0.01144

85.70772 0.01144

85.79614 0.01144

85.88456 0.01144

85.97299 0.01144

86.06141 0.01144

86.14983 0.01144

86.23825 0.01144

86.32667 0.01144

86.41509 0.01144

86.50351 0.01144

86.59194 0.01144

86.68036 0.01144

86.76878 0.01144

86.8572 0.01144

86.94562 0.01144

87.03404 0.01144

87.12246 0.01144

87.21089 0.01144

87.29931 0.01144

87.38773 0.01144

87.47615 0.01144

87.56457 0.01144

87.65299 0.01144

87.74141 0.01144

87.82984 0.01144

87.91826 0.01144

88.00668 0.01144

88.0951 0.01144

88.18352 0.01144

88.27194 0.01144

88.36036 0.01144

88.44879 0.01144

88.53721 0.01144

88.62563 0.01144

88.71405 0.01144

88.80247 0.01144

88.89089 0.01144

88.97931 0.01144

89.06774 0.01144

89.15616 0.01144

89.24458 0.01144

89.333 0.01144

Fig. 6a

1002 0.72615

1004 0.83527

1006 0.92312

1008 0.9802

1010 1

1012 0.9802

1014 0.92312

1016 0.83527

1018 0.72615

1020 0.60653

1022 0.48676

1024 0.37533

1026 0.27808

1028 0.19803

1030 0.1357

1032 0.08989

1034 0.05863

1036 0.04017

1038 0.03432

1040 0.04398

1042 0.07769

1044 0.1534

1046 0.30423

1048 0.58638

1050 1.08902

1052 1.94458

1054 3.33671

1056 5.50123

1058 8.71433

1060 13.26286

1062 19.39407

1064 27.24766

1066 36.78049

1068 47.70172

1070 59.44

1072 71.16261

1074 81.85648

1076 90.4654

1078 96.05947

1080 98

1082 96.05947

1084 90.4654

1086 81.85648

1088 71.16261

1090 59.44

1092 47.70172

1094 36.78049

1096 27.24766

1098 19.39407

1100 13.26286

1102 8.71432

1104 5.50121

1106 3.33665

1108 1.94443

1110 1.08868

1112 0.58565

1114 0.30269

1116 0.15031

1118 0.07172

1120 0.03288

1122 0.01448

1124 0.00614

1126 0.00253

1128 0.00108

1130 6.68914E-4

1132 9.73917E-4

1134 0.00229

1136 0.00576

1138 0.01413

1140 0.03339

1142 0.07581

1144 0.16539

1146 0.34664

1148 0.69805

1150 1.35058

1152 2.51063

1154 4.48409

1156 7.69472

1158 12.68646

1160 20.09629

1162 30.58577

1164 44.72511

1166 62.83643

1168 84.82031

1170 110.00601

1172 137.07593

1174 164.10968

1176 188.77107

1178 208.62429

1180 221.5249

1182 226

1184 221.5249

1186 208.62429

1188 188.77107

1190 164.10968

1192 137.07593

1194 110.00601

1196 84.82031

1198 62.83643

1200 44.72511

1202 30.58577

1204 20.09629

1206 12.68646

1208 7.69472

1210 4.48409

1212 2.51063

1214 1.35058

1216 0.69805

1218 0.34664

1220 0.16539

1222 0.07581

1224 0.03339

1226 0.01413

1228 0.00574

1230 0.00224

1232 8.42224E-4

1234 3.03702E-4

1236 1.05219E-4

1238 3.50244E-5

1240 1.12015E-5

1242 3.44198E-6

1244 1.01617E-6

1246 2.88242E-7

1248 7.85555E-8

1250 2.0571E-8

1252 5.18254E-9

1254 1.28553E-9

1256 4.39921E-10

1258 6.83045E-10

1260 2.47129E-9

1262 9.38777E-9

1264 3.44366E-8

1266 1.21402E-7

1268 4.11209E-7

1270 1.33823E-6

1272 4.18433E-6

1274 1.25704E-5

1276 3.62829E-5

1278 1.0062E-4

1280 2.68097E-4

1282 6.86322E-4

1284 0.00169

1286 0.00399

1288 0.00906

1290 0.01976

1292 0.04141

1294 0.0834

1296 0.16135

1298 0.29994

1300 0.53571

1302 0.91928

1304 1.51564

1306 2.40088

1308 3.65405

1310 5.34326

1312 7.50701

1314 10.1334

1316 13.14231

1318 16.37633

1320 19.60602

1322 22.5523

1324 24.92414

1326 26.46536

1328 27

1330 26.46536

1332 24.92414

1334 22.5523

1336 19.60602

1338 16.37633

1340 13.14231

1342 10.1334

1344 7.50701

1346 5.34326

1348 3.65405

1350 2.40088

1352 1.51564

1354 0.91928

1356 0.53571

1358 0.29994

1360 0.16135

1362 0.0834

1364 0.04141

1366 0.01976

1368 0.00906

1370 0.00399

1372 0.00169

1374 6.95267E-4

1376 2.91928E-4

1378 1.6163E-4

1380 1.86348E-4

1382 3.67214E-4

1384 8.09459E-4

1386 0.00176

1388 0.00368

1390 0.00742

1392 0.01436

1394 0.02671

1396 0.04774

1398 0.08203

1400 0.13551

1402 0.21532

1404 0.32922

1406 0.48479

1408 0.68832

1410 0.94395

1412 1.25348

1414 1.6174

1416 2.03744

1418 2.52055

1420 3.08323

1422 3.75483

1424 4.57745

1426 5.60085

1428 6.87147

1430 8.41655

1432 10.22655

1434 12.24072

1436 14.34119

1438 16.35961

1440 18.0976

1442 19.35804

1444 19.98068

1446 19.87354

1448 19.03205

1450 17.54087

1452 15.55828

1454 13.28733

1456 10.94132

1458 8.71168

1460 6.74453

1462 5.12928

1464 3.89862

1466 3.0373

1468 2.49569

1470 2.20448

1472 2.08815

1474 2.07612

1476 2.1118

1478 2.16008

1480 2.21459

1482 2.30589

1484 2.51156

1486 2.96896

1488 3.8901

1490 5.57627

1492 8.4276

1494 12.94043

1496 19.68475

1498 29.25649

1500 42.20582

1502 58.95234

1504 79.70761

1506 104.43056

1508 132.83602

1510 164.46052

1512 198.76384

1514 235.22166

1516 273.35402

1518 312.64951

1520 352.38437

1522 391.38956

1524 427.8636

1526 459.3415

1528 482.8963

1530 495.57564

1532 494.98644

1534 479.87235

1536 450.51154

1538 408.80622

1540 358.02632

1542 302.27303

1544 245.80554

1546 192.40089

1548 144.88847

1550 104.9338

1552 73.06958

1554 48.91174

1556 31.46902

1558 19.45816

1560 11.56208

1562 6.6018

1564 3.62212

1566 1.90952

1568 0.96725

1570 0.47076

1572 0.22014

1574 0.09891

1576 0.0427

1578 0.01771

1580 0.00706

1582 0.0027

1584 9.94113E-4

1586 3.51371E-4

1588 1.19323E-4

1590 3.89326E-5

1592 1.22048E-5

1594 3.67601E-6

1596 1.06378E-6

1598 2.95769E-7

1600 7.90103E-8

1602 2.02788E-8

1604 5.0007E-9

1606 1.1848E-9

1608 2.69706E-10

1610 5.89878E-11

1612 1.23955E-11

1614 2.5026E-12

1616 4.85455E-13

1618 9.04762E-14

1620 1.62012E-14

1622 2.78734E-15

1624 4.60744E-16

1626 7.31741E-17

1628 1.11656E-17

1630 1.63696E-18

1632 2.3058E-19

1634 3.12056E-20

1636 4.05762E-21

1638 5.06919E-22

1640 6.08464E-23

1642 7.01711E-24

1644 7.77518E-25

1646 8.27735E-26

1648 8.46642E-27

1650 8.32025E-28

1652 7.856E-29

1654 7.1268E-30

1656 6.21178E-31

1658 5.20195E-32

1660 4.18547E-33

1662 3.23556E-34

1664 2.40317E-35

1666 1.71493E-36

1668 1.17581E-37

1670 7.74563E-39

1672 4.90234E-40

1674 2.98112E-41

1676 1.74174E-42

1678 9.77721E-44

1680 5.27321E-45

1682 2.73252E-46

1684 1.36044E-47

1686 6.50766E-49

1688 2.99087E-50

1690 1.32068E-51

1692 5.6031E-53

1694 2.28395E-54

1696 8.94482E-56

1698 3.36578E-57

1700 1.21683E-58

1702 4.22667E-60

1704 1.41058E-61

1706 4.52298E-63

1708 1.39341E-64

1710 4.12442E-66

1712 1.17294E-67

1714 3.2049E-69

1716 8.41362E-71

1718 2.12216E-72

1720 5.14285E-74

1722 1.19745E-75

1724 2.67878E-77

1726 5.75767E-79

1728 1.18901E-80

1730 2.35912E-82

1732 4.49721E-84

1734 8.23693E-86

1736 1.44949E-87

1738 2.45072E-89

1740 3.98108E-91

1742 6.21349E-93

1744 9.31749E-95

1746 1.34243E-96

1748 1.85828E-98

1750 2.47148E-100

1752 3.15816E-102

1754 3.87738E-104

1756 4.57373E-106

1758 5.1836E-108

1760 5.64444E-110

1762 5.90524E-112

1764 5.93585E-114

1766 5.73267E-116

1768 5.31935E-118

1770 4.74229E-120

1772 4.06206E-122

1774 3.34298E-124

1776 2.64331E-126

1778 2.00813E-128

1780 1.46576E-130

1782 1.02792E-132

1784 6.9261E-135

1786 4.48379E-137

1788 2.78887E-139

1790 1.66664E-141

1792 9.56933E-144

1794 5.27898E-146

1796 2.798E-148

1798 1.42486E-150

1800 6.97149E-153

1802 3.27723E-155

1804 1.48019E-157

1806 6.42325E-160

1808 2.67807E-162

1810 1.07279E-164

1812 4.12894E-167

1814 1.52683E-169

1816 5.42461E-172

1818 1.85172E-174

1820 6.07311E-177

1822 1.91371E-179

1824 5.79385E-182

1826 1.68534E-184

1828 4.71016E-187

1830 1.26477E-189

1832 3.263E-192

1834 8.08817E-195

1836 1.92625E-197

1838 4.40759E-200

1840 9.6899E-203

1842 2.04675E-205

1844 4.15374E-208

1846 8.09919E-211

1848 1.5173E-213

1850 2.73106E-216

1852 4.723E-219

1854 7.84754E-222

1856 1.25279E-224

1858 1.92154E-227

1860 2.83171E-230

1862 4.00938E-233

1864 5.45423E-236

1866 7.12883E-239

1868 8.95223E-242

1870 1.08012E-244

1872 1.25211E-247

1874 1.39457E-250

1876 1.49233E-253

1878 1.53433E-256

1880 1.51566E-259

1882 1.4385E-262

1884 1.31175E-265

1886 1.14926E-268

1888 9.67413E-272

1890 7.82411E-275

1892 6.07976E-278

1894 4.53906E-281

1896 3.25592E-284

1898 0

1900 0

1902 0

1904 0

1906 0

1908 0

1910 0

1912 0

1914 0

1916 0

1918 0

1920 0

1922 0

1924 0

1926 0

1928 0

1930 0

1932 0

1934 0

1936 0

1938 0

1940 0

1942 0

1944 0

1946 0

1948 0

1950 0

1952 0

1954 0

1956 0

1958 0

1960 0

1962 0

1964 0

1966 0

1968 0

1970 0

1972 0

1974 0

1976 0

1978 0

1980 0

1982 0

1984 0

1986 0

1988 0

1990 0

1992 0

1994 0

1996 0

1998 0

2000 0

Fig. 6b

1002 0.29816

1004 0.5698

1006 1.06094

1008 1.92064

1010 3.37374

1012 5.73975

1014 9.44268

1016 15.00144

1018 22.98935

1020 33.95389

1022 48.29641

1024 66.12451

1026 87.10549

1028 110.36208

1030 134.45436

1032 157.48015

1034 177.29975

1036 191.85479

1038 199.51796

1040 199.39208

1042 191.48239

1044 176.69675

1046 156.67321

1048 133.48057

1050 109.26761

1052 85.94282

1054 64.9484

1056 47.15904

1058 32.90007

1060 22.05273

1062 14.20235

1064 8.78798

1066 5.22455

1068 2.98427

1070 1.63779

1072 0.86359

1074 0.43751

1076 0.21297

1078 0.09965

1080 0.0449

1082 0.01972

1084 0.00903

1086 0.00569

1088 0.00713

1090 0.01387

1092 0.02943

1094 0.06141

1096 0.12357

1098 0.23905

1100 0.44436

1102 0.79364

1104 1.3619

1106 2.24539

1108 3.55686

1110 5.41341

1112 7.91595

1114 11.12149

1116 15.01244

1118 19.47009

1120 24.26123

1122 29.04596

1124 33.41081

1126 36.92465

1128 39.20795

1130 40

1132 39.20795

1134 36.92465

1136 33.41081

1138 29.04596

1140 24.26123

1142 19.47009

1144 15.01244

1146 11.12149

1148 7.91595

1150 5.41341

1152 3.55687

1154 2.24539

1156 1.3619

1158 0.79366

1160 0.44442

1162 0.23923

1164 0.12408

1166 0.06284

1168 0.03323

1170 0.02355

1172 0.03083

1174 0.0614

1176 0.13478

1178 0.29229
[truncated: 354,761 more chars]
